# Post-functionalization of dibenzothiophene to functionalized biphenyls via a photoinduced thia-Baeyer-Villiger oxidation

**DOI:** 10.1038/s41467-020-14522-7

**Published:** 2020-02-14

**Authors:** Xiaofeng Ma, Yazhou Liu, Le Du, Jingwei Zhou, István E. Markó

**Affiliations:** 1 Natural Products Research Centre, Chengdu Institute of Biology, Chinese Academy of Sciences, 610041 Chengdu, People’s Republic of China; 2 Laboratory of Organic and Medicinal Chemistry, Université Catholique de Louvain, Place Louis Pasteur 1 bte L4. 01. 02, 1348 Louvain-la-Neuve, Belgium; 3 Antibiotics Research and Re-evaluation Key Laboratory of Sichuan Province, Sichuan Industrial Institute of Antibiotics, Chengdu University, 610052 Chengdu, People’s Republic of China; 4 Institute of Clinical Pharmacology, Guangzhou University of Chinese Medicine, 510405 Guangzhou, People’s Republic of China

**Keywords:** Photocatalysis, Synthetic chemistry methodology, Synthetic chemistry methodology

## Abstract

The Baeyer-Villiger reaction is used extensively in organic chemistry. Sila- and bora-variants have also been documented widely, with these processes underpinning, for example, the Fleming-Tamao oxidation and hydroborative alkene hydration, respectively. By contrast, the development of thia-Baeyer-Villiger reactions involving sulfoxides has long been considered unlikely because competitive oxidation to the sulfone occurs exclusively. Here, we disclose a photoinduced thia-Baeyer-Villiger-type oxidations; specifically, we find that exposure of dibenzothiophene (DBT) derivatives to an iron porphyrin catalyst under Ultraviolet irradiation in the presence of *t*-BuOOH generates sulfinic esters in up to 87% yield. The produced sulfinic esters are transformed to a variety of biphenyl substrates including biphenyl sulfoxides, sulfones and sulfonamides in 1-2 steps. These results provide a mild process for the selective functionalization of sulfur compounds, and offer a biomimetic approach to convert DBT into 2-hydroxybiphenyl under controllable stepwise pathway. Based upon experimental evidences and DFT calculation, a mechanism is proposed.

## Abstract (web summary)

The development of thia-Baeyer-Villiger reactions has been elusive so far due to competitive oxidation of sulfoxides to sulfones. Here, the authors show a thia-Baeyer-Villiger-type oxidations converting dibenzothiophene derivatives into sulfinic esters with *t*-BuOOH and an iron catalyst under UV irradiation.

## Introduction

**Fig. 1:**
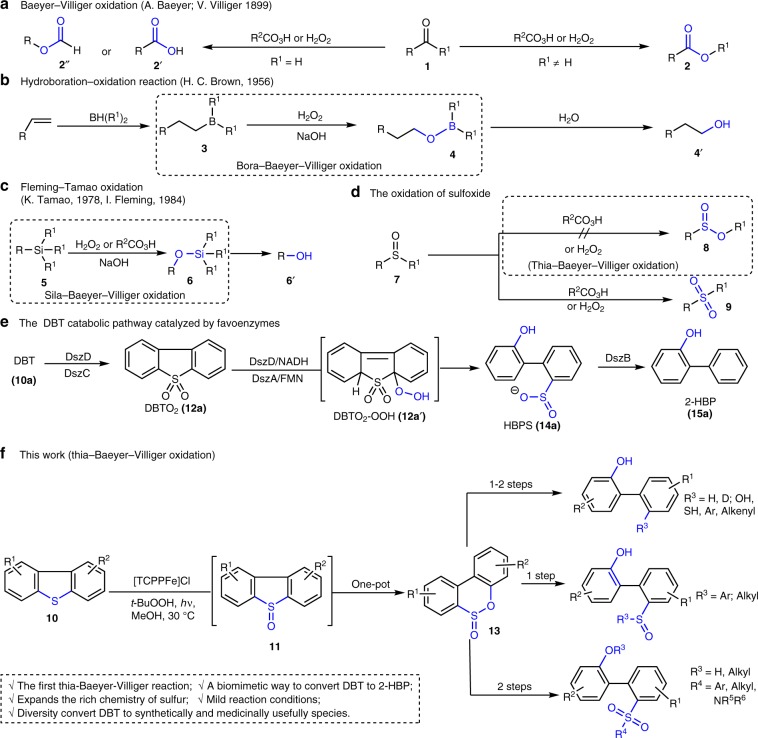
Examples of oxygen insertion reactions. **a** Hydroperoxides or peracids promoted Baeyer–Villiger oxidation of ketone or aldehyde, which via oxygen insert to C–C or C–H bond. **b** Hydroboration oxidation of alkene, in which the last step via oxygen from hydroperoxides insert to C–B bond. **c** Fleming-Tamao oxidation through oxygen from hydroperoxides or peracids oxygen insert to C–Si bond. **d** In the presence of hydroperoxides or peracids, sulfoxide–carbon bond cannot take place oxygen insert reaction to produce sulfinic esters **8**, but formed sulfone **9** via direct oxidation of sulfoxide. **e** DBT (**10a)** catabolic pathway catalyzed by Favoenzymes, in which oxygen from flavin hydroperoxide nucleophilic addition to DBTO_2_ formed DBTO_2_-OOH. **f** Photoinduced thia-Baeyer–Villiger reaction, in which oxygen from *t*-BuOOH insert to sulfoxide–carbon bonds (This work). **DszA:** DBT-5,5´-dioxide (DBTO_2_) monooxygenase, **DszB:** 2-(2´-hydroxyphenyl) benzenesulfinate (HBPS) desulfinase, **DszC:** DBT monooxygenase, **DszD:** flavin reductase, **NADH:** nicotinamide adenine dinucleotide, **FMN:** flavin mononucleotide.

The Baeyer–Villiger oxidation involves the insertion of an oxygen atom into either the C–C or C–H bond of a carbonyl compound (i.e., aldehydes or ketones **1**). The process is a particularly important transformation for the synthesis of esters **2** or carboxylic acids **2**′, and can be promoted by a wide variety of oxidants, including peracids and hydroperoxides (Fig. 1a)^1–4^. The regiochemical outcome of such reactions rests upon stringent stereo-electronic factors, and these have been elucidated by detailed mechanistic studies, which, in turn, allows regioselectivity to be predicted on a case-by-case basis^2–4^. In a related transformation, Brown reported that treatment of boranes **3** with basic hydrogen peroxide affords the corresponding borate esters **4** via a bora-analog of the Baeyer–Villiger rearrangement; subsequent hydrolysis generates the corresponding alcohols. This process is the final step of the alkenes hydroboration reaction, which effects the net anti-Markovnikov hydration of alkenes (Fig. 1b)^5–7^. Tamao and Fleming^8–11^ subsequently described the sila-version of the Baeyer–Villiger reaction. Depending upon the structure of the silane **5**, either hydroperoxides or peracids can be employed to promote oxidative rearrangement to the corresponding silyl ether **6** (Fig. 1c). The versatility, predictability, and mildness of these transformations accounts for their popularity in numerous synthetic ventures. In stark contrast, related oxygen atom insertion processes involving carbon sulfoxide bonds, to generate the corresponding sulfinic esters **8**, have not been reported, and, indeed, have long been considered a hopeless endeavor. Here, a lower energy pathway is available where the sulfoxide **7** is converted to the corresponding sulfone **9** (Fig. 1d)^12,13^. Even if such a thia-Baeyer–Villliger reaction could be achieved, it has been shown in certain cases that sulfinic esters **8** will rearrange to sulfones **9**, which highlights a further challenge that must be overcome^12^.

Dibenzothiophene (DBT, **10a**) and its methyl substituted derivatives, such as 4-methyl-dibenzothiophene and dimethyl-dibenzothiophenes (generally, 4,6-, 3,7-, and 2,8-dimethyl-DBTs), present as the major sulfur-containing heterocycles in crude petroleum. These are among the most recalcitrant compounds in deep desulfurization processes and can only be partially removed by conventional metal-catalyzed hydrodesulfurization (HDS) under high temperature and very high H_2_ pressure^14,15^. As a potential substitution for HDS, the biological desulfurization catalyzed by enzymes has drawn much attention in recent years due to its high selectivity, milder conditions, and lower CO_2_ emissions^16–19^. Among the developed biological desulfurization processes, the DBT catabolic pathway in Rhodococcus erythropolis has been well studied. In this process, DBT is initially oxidized to sulfone DBTO_2_ **12a** via sulfoxide DBTO **11a**. The resulting DBTO_2_ is subsequently converted to 2-(2-hydroxybiphenyl)-benzenesulfinate (HBPS, **14a**) through a flavin hydroperoxide mediated rearrangement. Then, desulfurization of HBPS by desulfinase (DszB) forms 2-hydroxybiphenyl (2-HBP, **15a**) (Fig. 1e)^18^. Despite the great success in catabolization of DBT, the transformation still presents challenge of finding a microbial strain with high efficiency and broad scope of sulfur compounds, including substituted DBT derivatives. A biomimetic approach that can imitate the DBT catabolic pathway to efficiently convert DBT, especially its derivatives, to 2-HBP under mild conditions is thus highly desired.

In this article, we report our preliminary results on the discovery of a thia-Baeyer–Villiger reaction of DBT derivatives (**10**), leading to the corresponding sulfinic esters (**13**) via corresponding sulfoxides (**11**) (Fig. 1f). This demonstrates the feasibility of an oxygen insertion process into a sulfoxide-carbon linkage. Most notably, our observations expand the rich chemistry of sulfoxides and provide a biomimetic way to convert DBT and their derivatives to HBPS and 2-HBP under controllable condition, which offer an easy and efficient way for their conversion into other synthetically and medicinally useful products.

## Results

### Discovery and optimization of thia-Baeyer–Villiger oxidation

During studies on the biomimetic oxidation of DBT **10a** catalyzed by the iron porphyrin complex [TCPPFe]Cl (5,10,15,20-Tetrakis(2-chlorophenyl)porphyrinato) iron (III) chloride)^20–32^, we observed that the initial oxidation product, sulfoxide DBTO **11a**, underwent facile conversion to the corresponding sulfone DBTO_2_ **12a**^20^. When this reaction was run on larger scale, we were able to isolate small amounts of an unknown compound possessing the same molecular formula (C_12_H_12_SO_2_) as sulfone DBTO_2_ **12a**. Spectroscopic analysis indicated that this product was non-symmetrical and it was assigned as sulfinic ester (BPS, **13a**) by comparison to data reported by Crich^33^, who prepared this compound via a different synthetic route. Finally, single crystal X-ray diffraction analysis of related sulfinic ester **13e**, which was prepared in a manner analogous to BPS, unambiguously established the structures of these thia-Baeyer–Villiger products (see Table 2).

That such an oxidative insertion process took place under mild conditions was truly exciting. Various attempts to improve the yield of BPS **13a** by varying different reaction parameters met with failure. Ultimately, a key observation was made when the reaction was run in the dark, as, under these conditions, only sulfide DBT **10a** was recovered in 92% yield (Table 1, entry 2). It was thus evident that light is necessary for the formation of BPS **13a**. Indeed, irradiation of a methanol solution containing the iron porphyrin catalyst and DBT **10a** using a 250 Watt high-pressure Hg lamp in the presence of 6.0 equiv. of *t*-BuOOH provided sulfinic ester BPS **13a** in 86% yield (Table 1, entry 1, see Supplementary Figs. 3–9 for reaction progress monitored by HPLC). No product was formed when the reaction was performed under omission of the catalyst TCPPFeCl (Table 1, entry 3). Similarly, the corresponding manganese porphyrin catalyst was not effective and gave only trace amount of desired product (Table 1, entry 4). The other iron porphyrin catalysts, including [T(*o*-NO_2_)PPFe]Cl, [T(*o*-NH_2_)PPFe]Cl, and [TPPFe]Cl were also tested, which afforded the target product in 15%-62% yield. (Table 1, entries 5-7). Increasing *t*-BuOOH did not improve yield (Table 1, entry 1 vs entry 10), but decreasing it resulted in reducing the yield dramatically (Table 1, entry 1 vs. entries 8, 9). Lastly, changing the power of light only increased the yield of oxidized product DBTO_2_ (Table 1, entries 11 and12). Furthermore, the reaction can start from DBTO directly in the presence of 4.0 equiv. of *t*-BuOOH, under those condition, the target product was isolated in 84% yield (Table 1, entry 13, see Supplementary Figs. 10–14 for reaction progress monitored by HPLC).

**Table 1:** Optimization of the thia-Baeyer–Villiger reaction.


Entry	Variations from standard conditions	Yield of BPS^a^
1	None	86%
2	No light	0^b^
3	No [TCPPFe]Cl	0^c^
4	[TCPPMn]Cl instead of [TCPPFe]Cl	trace
5	[TPPFe]Cl instead of [TCPPFe]Cl	39%
6	[T(*o*-NH_2_)PPFe]Cl instead of [TCPPFe]Cl	15%
7	[T(*o*-NO_2_)PPFe]Cl instead of [TCPPFe]Cl	62%
8	*t*-BuOOH (2.0 eq)	33%
9	*t*-BuOOH (4.0 eq)	63%
10	*t*-BuOOH (8.0 eq)	87%
11	500 W Hg Lamp	63%^d^
12	150 W Hg Lamp	69%^e^
13	From DBTO **11a** and *t*-BuOOH (4.0 eq)	84%


^*a*^Isolated yield.

^*b*^DBT was recovered in 92% yield.

^*c*^No reaction.

^*d*^DBTO_2_ was isolated in 30% yield.

^*e*^DBTO_2_ was isolated in 21% yield. **[TPPFe]Cl**: meso-tetraphenylporphinatoiron (III) chloride; **[TCPPFe]Cl**: (5,10,15,20-Tetrakis(2-chlorophenyl) porphyrinato) iron (III) chloride); **[T(*o*-NH**_**2**_**)PPFe]Cl**: (5,10,15,20-Tetrakis(2-amino)porphyrinato) iron (III) chloride); **[T(*o*-NO**_**2**_**)PPFe]Cl**: (5,10,15,20-Tetrakis(2-nitro)porphyrinato) iron (III) chloride); **[TCPPMn]Cl**: (5,10,15,20-Tetrakis(2-chlorophenyl) porphyrinato)manganese (III) chloride.

### Substrate scope of thia-Baeyer–Villiger oxidation

At this stage, the new protocol was applied to a range of aromatic sulfides and salient results are displayed in Table 2. As can be seen, the reaction tolerates a variety of substituents, ranging from alkyl groups to benzylic alcohols (**10f**), ethers (**10h**, **10i**) and alkenes (**10i**). Placement of a methyl substituent at the 4-position (**10b**) had little impact on the yield of the reaction; however, the non-symmetrical nature of the starting material **10b** resulted in the formation of two isomeric sulfinic esters, **13b** and **13b’** (Table 2, entry 3). Sulfide **10c**, which possesses methyl groups at the C4 and C6 positions, provided a decreased in the yield of **13c**, indicating that the process is sensitive to sterics (Table 2, entry 5). However, it is worth noting that **10c** is a particularly inert substrate, and its desulfurization, even under stringent conditions, proceeds in only marginal yields^14^. That adduct **13c** could be formed in 55% yield is a clear testimony to the power of this oxidative rearrangement process. Systems with methyl groups at other positions delivered the desired sulfinic esters in good to excellent yields (Table 2, entries 7 and 9). Notably, when corresponding sulfoxides (**11a–11d**) were used as the starting materials, the thia-Baeyer–Villiger oxidation maintained the same efficiency (Table 2, entry 1 vs. entry 2; entry 3 vs. entry 4; entry 5 vs. entry 6). Sulfide **10f**, which contains an unprotected benzylic alcohol, underwent thia-Baeyer–Villiger rearrangement to provide regioisomers **13f** and **13f’** in a 1:1 ratio (Table 2, entry 10). Benzylic ether containing sulfides **10h** and **10i** afforded oxidized products **13h** and **13i** in 70% and 62% yield, respectively (Table 2, entries 12 and 13). Highly electron-rich sulfide **10j**, could also be oxidized to provide sulfinic ester **13j**, albeit in modest yield (Table 2, entry 14). A current limitation of the method involves electron-deficient sulfides, as evidenced by substrate **10k**, which did not provide thia-Baeyer–Villiger product **13k** (Table 1, entry 15), while the corresponding sulfoxide **11k** was subjected to the standard conditions, deoxygenation of the sulfoxide occurred in 89% yield (Table 1, entry 16).

**Table 2:**
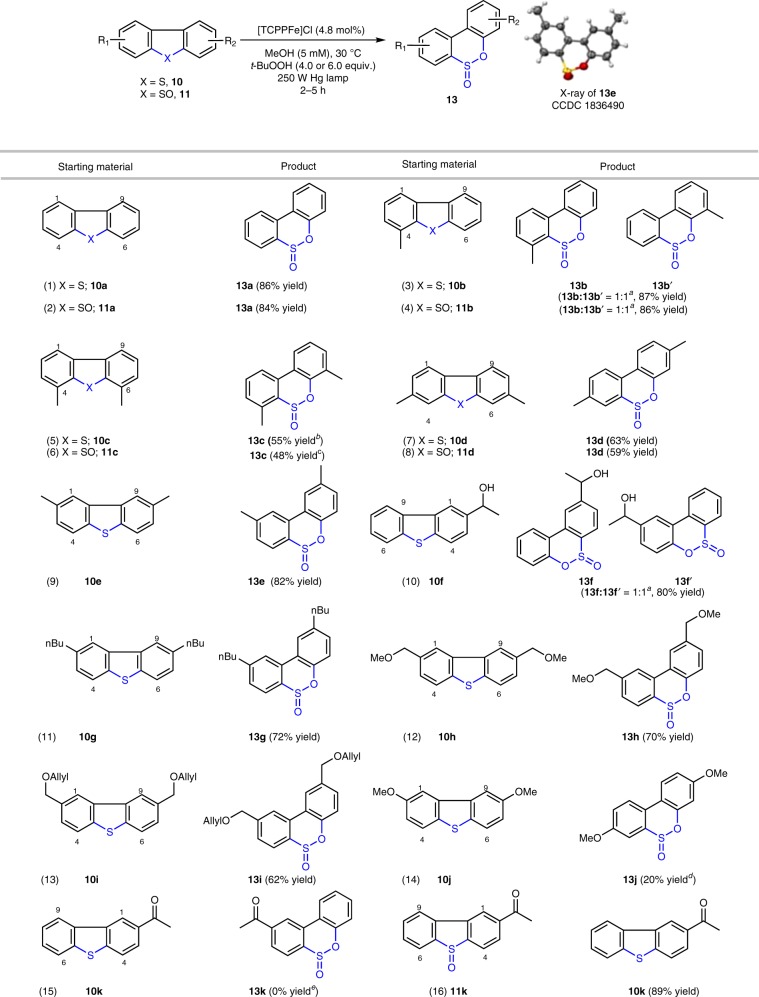
Scope of the Thia-Baeyer–Villiger Reaction.

^*a*^The ratio of two regioisomers.

^*b*^12.0 equiv. of *t*-BuOOH and 5.7 mol% [TCPPFe]Cl was used.

^*c*^8.0 equiv. of *t*-BuOOH and 5.7 mol% [TCPPFe]Cl was used.

^*d*^150 W Hg Lamp was used.

^*e*^No reaction occurred and only starting material was recovered.

### Mechanistic investigations of thia-Baeyer–Villiger oxidation

The photochemical behavior of DBTO and the corresponding sulfone DBTO_2_ has been investigated extensively^34^. The seminal work of Jenks^35–39^, Greer^40,41^, Nakai^42^, Shiraishi^43,44^, and others^45^ has provided important mechanistic insights and enabled the establishment of a general reaction framework that rationalizes the formation of various products observed during photochemical processes involving these compounds. In all cases, it has been established that irradiation of DBTO results in cleavage of the sulfoxide S–O bond to release sulfide DBT and atomic oxygen O(^3^P), which oxidizes the solvent. In certain cases, sulfinic ester has been observed, but only in trace amounts^46,47^. It was found that biphenylsultone, an overoxidation product of **13a** was formed in less than 5% yield in a catalytic oxidation of DBT by metal-sulfophthalocyanines catalysts in the presence of H_2_O_2_ or monopersulfate^48^. Hence, it appears that the porphyrin catalyst [TCPPFe]Cl is critical for the diversion of the reaction pathway towards thia-Baeyer–Villiger product sulfinic ester. Indeed, UV–Vis (Ultraviolet–visible) spectroscopy (Supplementary Fig. 15a) showed that upon addition of sulfoxide DBTO to [TCPPFe]Cl in the presence of *t*-BuOOH, a [DBTO·TCPPFe∙*t*-BuOOH]Cl complex was formed. When this complex was irradiated, a mixture of sulfinic ester and sulfide DBT was generated. Addition of imidazole to the same reaction solution, followed by irradiation, provided the desired product BPS in only 44% yield (HPLC yield) (Supplementary Table 1, entry 2) (See Supplementary Fig. 15b for UV–Vis spectroscopy). Imidazole is a good axial ligand for [TCPPFe]Cl^24,49,50^ and would be expected to displace rapidly DBTO from the metal complex. Consequently, the decrease of reactivity observed in the presence of imidazole indicates that the formation of a [DBTO·TCPPFe∙*t*-BuOOH]Cl complex is a compulsory step in the successful generation of BPS (see Supplementary Figs. 17–21 for reaction progress monitored by HPLC).

### Control experiments

A range of experiments were then performed to clarify the mechanism of the thia-Baeyer–Villiger reaction (Supplementary Table 1). Started from DBTO, in the absence of light (under air atmosphere, with PhIO as oxidant), only DBTO_2_ from the oxidation of DBTO was formed in 89% yield (Supplementary Table 1, entry 3), and lack of *t*-BuOOH and (or) catalyst led to reduce of DBTO to DBT (Supplementary Table 1, entries 4–6). The reaction was inhibited dramatically by radical quenchers such as (2,2,6,6-tetramethylpiperidin-1- yl)oxyl (TEMPO) and butylated hydroxytoluene (BHT) suggested a radical pathway (Supplementary Table 1, entries 7–10)^51–53^. Furthermore, addition of 1.0 equiv. of CuCl_2_ to the reaction mixture resulted in the formation of BPS in 59% yield (HPLC yield) (Supplementary Table 1, entry 11), which revealed a single-electron processes may be involved in this photochemical process^51–53^.

### Mechanism and DFT calculations

**Fig. 2:**
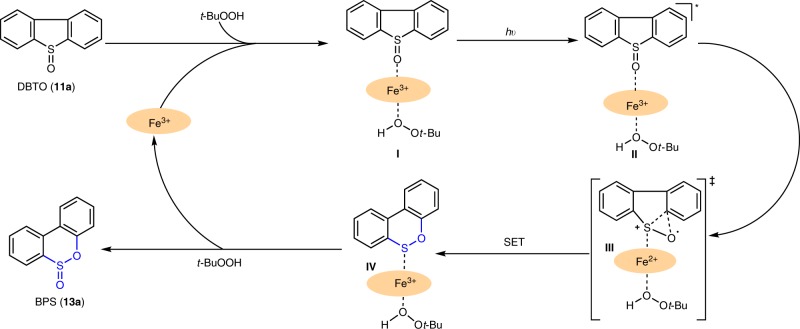
A plausible mechanism for the thia-Baeyer–Villiger reaction. **SET:** single-electron transfer.

Based on the observations outlined above, we propose a plausible reaction mechanism for the thia-Baeyer–Villiger reaction (Fig. 2). Starting from sulfide DBT, the Fe porphyrin complex and *t*-BuOOH effect initial oxidation to sulfoxide DBTO^20^. Ligation of DBTO to the Fe center of [TCPPFe]Cl forms complex **I**. At this stage, photochemical irradiation of **I** forms excited state **II**^52,54,55^, which undergoes C–S bond cleavage with concomitant C–O bond formation, leading to iron-coordinated sulfenic ester species **IV** via transition state **III**. Computational studies (Supplementary Fig. 72 and Supplementary Table 2) revealed that DBTO could directly isomerize to sulfenic ester **IV** without the Fe porphyrin via high-energy transition state (47.0 kcal/mol). However, the energy barrier was significantly lower (15.7 kcal/mol), in the presence of the Fe porphyrin complex (see **III** in Supplementary Fig. 72 and Supplementary Table 2). Thus, the process from **II** to **IV** is facilitated by the porphyrin Fe catalyst, via either a weakening of the C–S bond of **III** or stabilization of radical cation **III**. Either way, deviation of the process from established pathways (vide supra) requires smooth formation of intermediate **IV**^56,57^ rather than the formation of O(^3^P)^35–41,47^. From sulfenic ester **IV**, oxidation, presumably by an Fe/*t*-BuOOH complex, affords sulfinic ester and releases the iron catalyst to re-enter the catalytic cycle.

### A biomimetic desulfurization processes

**Fig. 3:**
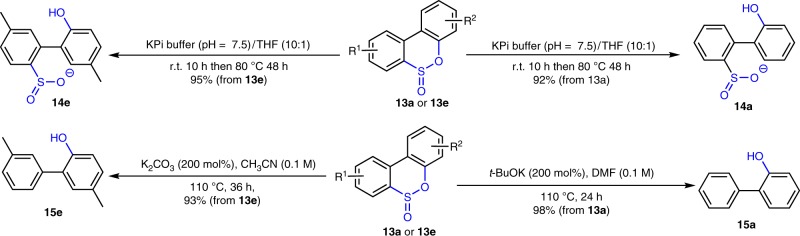
Biomimetic desulfurization. In KPi buffer or in the presence of bases, sulfinic esters (**13a** and **13e**) were smoothly converted to HBPS or 2-HBP derivatives. **KPi buffer**: potassium phosphate buffer.

In the biocatalytic desulfurization process depicted in Fig. 1e, DBT (**10a**) was oxidized to DBTO_2_ (**12a**) by DBT monooxygenase (DszC), which was further transformed to HBPS (**14a**) by DBTO_2_-monozygenase (DszA), the produced **14a** was then desulfurized by desulfinase (DszB) forms 2-hydroxybiphenyl (2-HBP) (**15a**)^18^. An analogous transformation can be achieved using the developed thia-Baeyer–Villiger methodology, as the rearrangement products sulfinic esters (for example, **13a** and **13e**) can easily hydrolyze into HBPS derivatives **14a** and **14e** in 92% and 95% yield when exposed to KPi buffer (Fig. 3)^18^. Alternatively, when **13a** and **13e** were heated in the presence of base in polar solvents, the corresponding desulfurized products 2-HBPs were formed in excellent yields (**15a** and **15e**, 98% and 93% yield, respectively).

### Diverse transformation of sulfinic esters

**Fig. 4:**
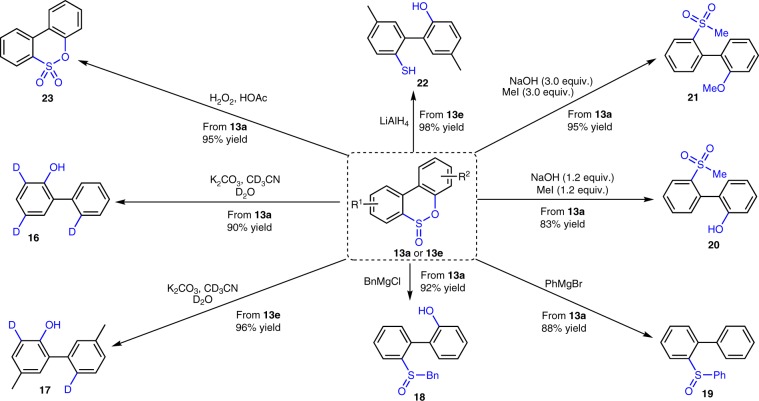
Diverse transform of sulfinic esters. Sulfinic esters **13a** and **13e** were transformed to medicinally and synthetically useful products **16**–**23**.

By combination of the developed thia-Baeyer–Villiger rearrangement with the base-induced desulfurization process, we successfully imitated the biocatalytic desulfurization process and convert DBT into 2-HBP in an efficient way, however, different from biocatalytic desulfurization, our method proceeded under a controllable stepwise conditions, thus, if necessary, the product from thia-Baeyer–Villiger reaction can be convert to a vast array of medicinally and synthetically useful intermediates (Fig. 4). Indeed, when sulfinic esters were treated with base in a deuterated solvent in presence of D_2_O, the corresponding deuterated products (**16** and **17**) were isolated in excellent yields (90% and 96%, respectively). Otherwise, when BPS **13a** was reacted with alkyl and aryl Grignard reagents, the biphenyl sulfoxides (**18** and **19**) were formed in 88% and 92% yields, respectively. Furthermore, the sulfinic ester is a dual electrophilic and nucleophilic functional group, it can react with nucleophiles and electrophiles under controllable conditions. For example, when BPS was first treated with nucleophilic NaOH, which was followed by the addition of electrophilic MeI, the biphenyl sulfone was generated in good yield, by control amount of NaOH and MeI, one can control the distribution of the product from 2-hydroxybiphenyl **20** (83% yield) to 2-methoxybiphenyl **21** (95% yield). Besides, the sulfur (IV) in sulfinic ester can take place facilely oxidation and reduction reaction, thus, when treated BPS with LiAlH_4_, the reduced product thiol-biphenol **22** was obtained in 98% yield, while H_2_O_2_ in HOAc efficiently oxidized BPS to biphenylsultone **23** in 95% yield^58^.

### Transformation of **23** to biphenyl sulfones and sulfonamides

Considering the prevalence of sulfones and sulfonamides in medicinal chemistry, the potential application of the current methodology was further demonstrated by transformation of biphenylsultone to a range of biphenyl sulfones and sulfonamides (Table 3). For example, when biphenylsultone **23** was submitted to aryl and alkyl lithium reagents, the corresponding biphenyl sulfones were obtained in good yields (**24a** and **24b**, 62% and 65% yield, respectively), while subjected sultone **23** to a solution lithium amide, the sulfonamides (**24c**–**24f**) were produced in 85% to 99% yields.

**Table 3:**
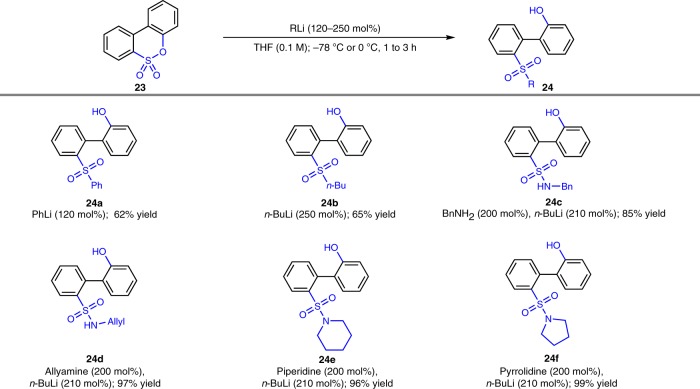
Synthesis of biphenyl sulfones and sulfonamides.^*a*^

^*a*^Isolated yield.

### Transformation of 2-HBPs to substituted biphenyl

Lastly, the desulfurized product 2-HBPs **15** are also useful building blocks for hydroxyl-directed C–H bond activation as exemplified in Table 4^59^. As described by Fan and co-workers^60^, in the presence of *t*-BuOOH, 2,2-biphenol was obtained in 76% yield from 2-HBP via a Pd(OAc)_2_ catalyzed C–H bond hydroxylation. Similarly, **15e** was converted to **25b** and **25c** in 62% and 48% yield, respectively, by Pd(OAc)_2_ catalyzed C–H bond arylation and alkenylation.

**Table 4:**
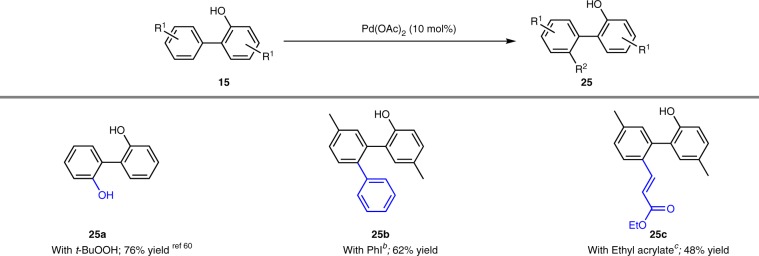
Hydroxyl-directed C–H bond activation.^*a*^

^*a*^Isolated yield.

^*b*^Reaction conditions: Pd(OAc)_2_ (10 mol%), PhI (120 mol%), Cs_2_CO_3_ (120 mol%), DMF (0.2 M), 100 °C, 24 h.

^*c*^Reaction conditions: Pd(OAc)_2_ (10 mol%), ethyl acrylate (200 mol%), 1,4-benzoquinone (100 mol%), HOAc (0.5 M), 80 °C, 24 h.

## Discussion

We have discovered an efficient thia-Baeyer–Villiger oxidation that enables the conversion of sulfides to sulfinic esters via the corresponding sulfoxides by an iron porphyrin catalyst under UV irradiation. The process provides a mild way for the selective functionalization of sulfur. Our methodology successfully imitated the DBT catabolic pathway in Rhodococcus erythropolis to convert DBT into 2-HBP under a controllable and efficient stepwise pathway, which was beneficial to a wide range of further transformation. By maximum three steps, DBT and their derivatives were transformed to a wide range of biphenyl substrates, such as biphenyl thiol, sulfoxides, sulfones, sulfonamides, and 2,2-biphenols. Mechanism research shows that the photoinduced thia-Baeyer–Villiger oxidation may take place via a radical cation pathway.

## Methods

### General procedures for thia-Baeyer–Villiger reaction of DBT

To a stirring solution of DBT or their derivatives (0.15 mmol) and [TCPPFe]Cl (6 mg, 4.8 mol%) in MeOH (30 mL) was added 6.0 equiv. of *t-*BuOOH (160 μL, 5.5 M in decane) over 5 min at 30 °C (bath temperature) (the bath temperature may rise to 35 °C during reaction), the resulted mixture was exposed to 250 W high-pressure mercury lamp, after the reaction was complete (2–3 h) as judged by thin-layer chromatography analysis, the solvent was removed under reduced pressure to give the crude product, which was purified by flash SiO_2_ gel column chromatography to obtain the product.

## Supplementary information

Supplementary Information

Description of Additional Supplementary Files

Supplementary Data 1

## Data Availability

The authors declare that full experimental details, mechanistic studies, UV–Vis spectra and characterization of compounds are available in the Supplementary Information. The X-ray crystallographic data for compound **13e** reported in this study has been deposited at the Cambridge Crystallographic Data Center (CCDC), under deposition number 1836490. These data can be obtained free of charge from The Cambridge Crystallographic Data Center via www.ccdc.cam.ac.uk/data_request/cif. All other data are available from the authors upon reasonable request.
