# Supplementary material for: Post-functionalization of dibenzothiophene to functionalized biphenyls via a photoinduced thia-Baeyer-Villiger oxidation
Source: Nat Commun. 2020 Feb 14;11:914. doi: 10.1038/s41467-020-14522-7 (PMC7021910; doi:10.1038/s41467-020-14522-7)
Supplement: Supplementary file 1 — Supplementary Information [file 41467_2020_14522_MOESM1_ESM.pdf]

Supplementary Information

Post-functionalization of Dibenzothiophene to Functionalized Biphenyls via a Photoinduced Thia-Baeyer-Villiger Oxidation

Ma et al

Supplementary Methods

General Information. Unless stated otherwise, all materials were purchased from commercial sources (Acros, Aldrich, Alfa Aesar, Fluorochem and Strem) and used without any further treatment. All reactions were conducted in commercial grade solvents were dried and purified by standard procedures as specified in Purification of Laboratory Chemicals, 4th Ed (Armarego, W. L. F.; Perrin, D. D. Butterworth Heinemann: 1997).

These are quartz immersion well reactors. They have a 400 mL filled volume and are suited for use with a water (or glycol/water circulator) cooled 500 W, 250 W, 150 W high-pressure Hg lamp (Shanghai Jiguang Special Illumination Instrument Factory). The batch reactors were wrapped in aluminum foil to reflect back light.

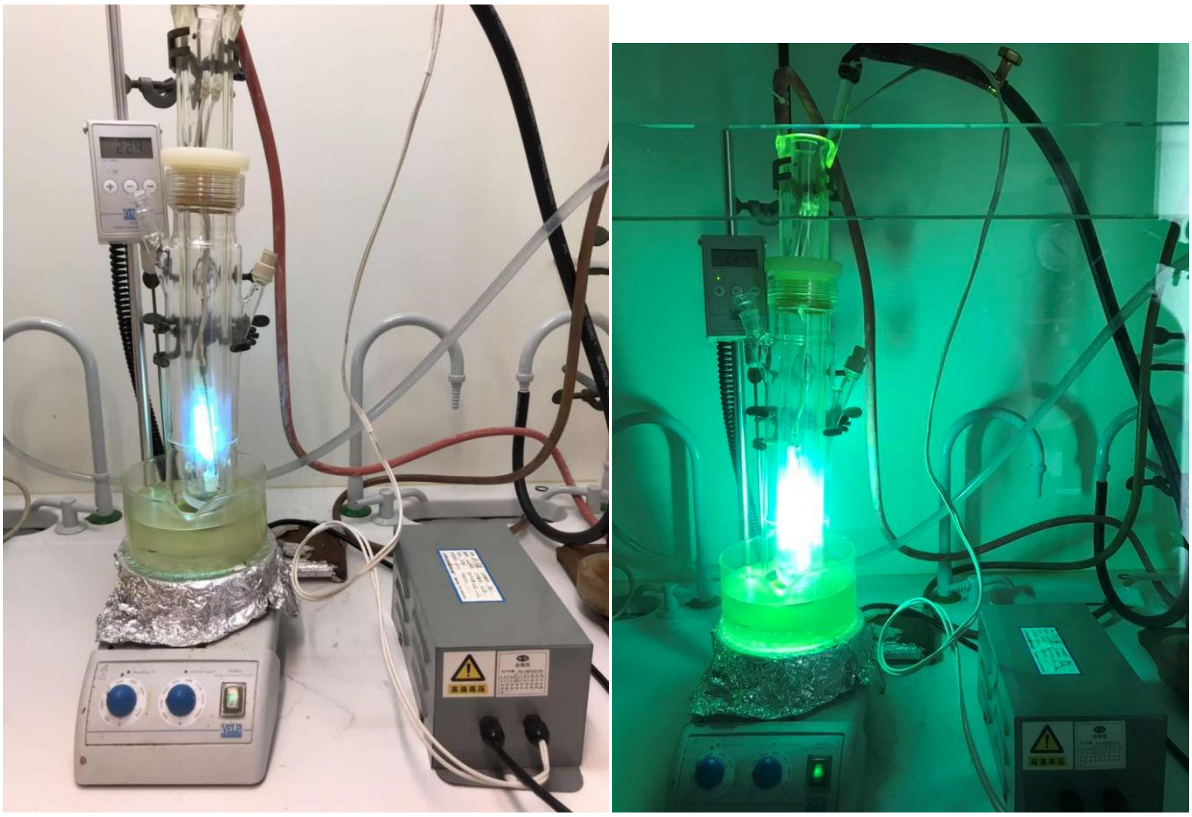

a. **b.**

Supplementary Figure 1. The photochemical equipment used in this study. a. At the beginning of the reaction**; b.** After 10 min. Please note the aluminum foil for reflect back light was removed during taking the photographies.

Proton nuclear magnetic resonances (^1H NMR) spectra were recorded at 300 MHz, 400 MHz or 500 MHz. Carbon-13 nuclear magnetic resonances (^{13}C NMR) spectra were recorded at 75 MHz, 101 MHz or 126 MHz. Chemical shifts (δ) are reported in ppm from $CDCl_3$ (δ = 7.26 ppm), DMSO-

d_6 (δ = 2.50 ppm) or CD_3OD (δ = 3.31 ppm) for ^1H NMR and relative to $CDCl_3$ (δ = 77.2 ppm), DMSO-d_6 (δ = 39.5 ppm), CD_3OD (δ = 49.0 ppm) for ^{13}C NMR. Chemical shifts (δ) are given in parts per million (ppm). Peaks are described as singlet (s), doublet (d), triplet (t), quartet (q), multiplet (m) and broad (br). Coupling constants (J) are quoted to the nearest 0.5 Hz. Flash column chromatography was performed over ROCC Silica gel 60 (40-63 μmesh) eluting with ethyl acetate (EA) (or dichloromethane (DCM)) and petroleum ether (PE). Thin layer chromatography (TLC) was performed using aluminium backed $60F_{254}$ silica plates. Visualization was achieved by UV fluorescence or a basic $KMnO_4$ solution and heat. High-resolution mass data (HRMS) were obtained with a Kratos MS50TC instrument. Infrared (IR) spectra were recorded by transmittance on a Shimadzu FTIR-8400S spectrometer as neat films and reported in wave number (cm^{-1}). Melting points were measured on a Buchı B-545 melting point apparatus and are uncorrected. Single-crystal X-ray diffractions were measured and the crystal structures determined by Dr. Koen Robeyns from the Crystallography Service, Université catholique de Louvain. UV-Vis spectra were achieved with VWR UV-1600 PC spectrophotometer (λ = 190-900 nm). All HPLC spectra were recorded on a Shimadzu Essentia LC-16 equipped with a WR5-5085-C18 (4.6×250 mm, 5μm) and a DAD detector.

General procedure A for preparation of DBTO and their derivatives

Dibenzo[*b*,*d*]thiophene 5-oxide (DBTO) and their derivatives were synthesized according to a literature procedure.[1] Dibenzothiophene (DBT) is suspended in trifluoroacetic acid (TFA) (0.74 mL of TFA per mmol of DBT) at room temperature. To the suspension is slowly dropped 35% aqueous hydrogen peroxide (0.11 mL of hydrogen peroxide per mmol of DBT) while cooling with ice so as to maintain the temperature around 60 °C. After completion of dropping, the reaction solution is stirred at room temperature for 30 minutes. Then, the reaction solution is added to 100 mL of water to precipitate crystals. The crystals are recovered by filtration and washed with water. The obtained crystals are further purified by recrystallization or flash chromatography.

General procedure B for Thia-Baeyer-Villiger Reaction starting from DBT and their derivatives

To a stirring solution of DBT or their derivatives (0.15 mmol) and [TCPPFe]Cl (6.0 mg, 4.8 mol%) in 30 mL of MeOH was added 6.0 equiv. of *t*-BuOOH (5.5 M in decane) over 5 min at 30 ˚C (Bath temperature) (Please note, the bath temperature may rise to 35 ˚C during reaction), the resulted mixture was exposed to 250 W high pressure mercury lamp for a given time, the solvent was removed under reduced pressure to give the crude product, which was purified by flash SiO_2 gel column chromatography to achieve the product.

General procedure C for Thia-Baeyer-Villiger Reaction starting from DBTO and their derivatives

To a stirring solution of DBTO or their derivatives (0.15 mmol) and [TCPPFe]Cl (6.0 mg, 4.8 mol%) in 30 mL of MeOH was added 4.0 equiv. of *t*-BuOOH (5.5 M in decane) over 5 min at 30 °C (Bath temperature) (Please note, the bath temperature may rise to 35 °C during reaction), the resulted mixture was exposed to 250 W high pressure mercury lamp for a given time, the solvent was removed under reduced pressure to give the crude product, which was purified by flash SiO_2 gel column chromatography to achieve the product.

Reaction progress monitored by HPLC

Area normalization method was used for the calculation of HPLC yield.
Preparation of the sample: sample (100 μL) was taken from the reaction mixture and diluted to 1.0 mL with MeOH for HPLC analyses.
HPLC Conditions: gradient elution: 70% to 80% CH_3CN in H_2O from 0 min to 15 min; 80% to 90% CH_3CN in H_2O from 15 min to 20 min; 90% CH_3CN in H_2O after 20 min; *detector*: 254 nm, *flow rate*: 0.5 mL/min.

Synthesis of 4-methyl-dibenzo[*b*,*d*]thiophene (10b)

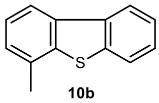

10b

10b was prepared according to a literature procedure. The spectroscopic properties of **10b** were consistent with the data available in the literature.[2]

Synthesis of 4,6-dimethyldibenzo[*b*,*d*]thiophene (10c)

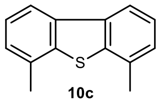

10c

10c was prepared according to a literature procedure. The spectroscopic properties of **10c** were consistent with the data available in the literature.[2]

Synthesis of 3,7-dimethyl-dibenzo[*b*,*d*]thiophene (10d)

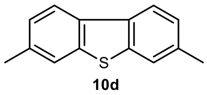

10d

To a flame dried flask was quickly added 3,7-dibromo-dibenzo[*b,d*]thiophene[3] (1.00 g, 2.93 mmol) under argon atmosphere. Then, 30 mL of anhydrous Et_2O was added and the solution was cooled to -78 °C. *n*-BuLi (2.5 M, 2.50 mL) was added dropwise. The resulted mixture was stirred at -78 °C

for 0.5 h and then at room temperature for 20 min, the mixture was cooled to -78 °C again and MeI (0.73 mL, 11.7 mmol) was added dropwise. The resulting mixture was stirred at reflux overnight. The reaction was cooled to room temperature then quenched by the addition of brine and diethyl ether. The resulting layers were separated and the aqueous layer was extracted with diethyl ether (3 × 30 mL). The combined organic layers were washed with brine, dried over Na_2SO_4, filtered, and the solvent was evaporated under reduced pressure to give the crude product, which was purified by flash SiO_2 gel column chromatography (PE) to afford the product **10d** (0.34 g, 55%) as a white solid. ^{1}H NMR (300 MHz, $CDCl_3$) δ 7.99 (d, *J* = 2.0 Hz, 2H), 7.64 (dt, *J* = 8.0, 1.0 Hz, 2H), 7.29 – 7.21 (m, 2H), 2.51 (s, 6H); ^{13}C NMR (75 MHz, $CDCl_3$) δ 139.5, 136.5, 133.4, 125.9, 122.9, 121.0, 21.8. The spectroscopic properties of **10d** were consistent with the data available in a literature.[4]

Synthesis of 2,8-dimethyl-dibenzo[*b*,*d*]thiophene (10e)

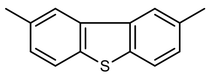

10e

To a flame dried flask was quickly added 2,8-dibromo-dibenzo[*b*,*d*]thiophene[5] (1.00 g, 2.93 mmol) under argon atmosphere. Then, 30 mL of anhydrous Et_2O was added and the solution was cooled to -78 °C. *n*-BuLi (2.5 M, 2.50 mL) was added dropwise. The resulted mixture was stirred at -78 °C for 0.5 h and then at room temperature for 20 min, the mixture was cooled to -78 °C again and MeI (0.73 mL, 11.7 mmol) was added dropwise. The resulting mixture was stirred at -78 °C for 2 h and then at room temperature overnight, then quenched by the addition of brine and diethyl ether. The resulting layers were separated and the aqueous layer was extracted with diethyl ether (3 × 30 mL). The combined organic layers were washed with brine, dried over Na_2SO_4, filtered, and the solvents were evaporated under reduced pressure to give the crude product, which was purified by flash SiO_2 gel column chromatography (PE) to afford the product **10e** (0.45 g, 72%) as a white solid. ^{1}H NMR (300 MHz, $CDCl_3$) δ 7.95 (dt, *J* = 2.0, 1.0 Hz, 2H), 7.72 (d, *J* = 8.0 Hz, 2H), 7.30 – 7.24 (m, 2H), 2.54 (s, 6H); ^{13}C NMR (75 MHz, $CDCl_3$) δ137.0, 135.8, 134.1, 128.2, 122.6, 121.9, 21.6. The spectroscopic properties of **10e** were consistent with the data available in a literature.[4]

Synthesis of 1-(dibenzo[*b*,*d*]thiophen-2-yl)ethanol (10f)

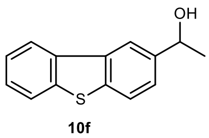

10f

10f was prepared according to a published procedure.[6] The spectroscopic properties of **10f** were consistent with the data available in the literature.[6]

Synthesis of 2,8-dibutyldibenzo[*b*,*d*]thiophene (10g)

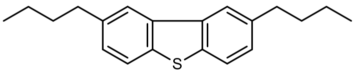

10g

To a flame dried flask was quickly added 2,8-dibromo-dibenzo[*b*,*d*]thiophene (1.00 g, 2.93 mmol) under argon atmosphere. Then, 10 mL of anhydrous THF was added and the solution was cooled to -78 °C. *n*-BuLi (2.5 M, 2.50 mL) was added dropwise. The resulted mixture was stirred at -78 °C for 0.5 h and then at room temperature for 20 min, the mixture was cooled to -78 °C again and *n*-iodobutane (1.40 mL, 12.3 mmol) was added dropwise. The resulting mixture was heated at reflux overnight. The reaction was cooled to room temperature then quenched by the addition of brine and diethyl ether. The resulting layers were separated and the aqueous layer was extracted with diethyl ether (3 × 30 mL). The combined organic layers were washed with brine, dried over Na_2SO_4, filtered, and the solvent was evaporated under reduced pressure to give the crude product, which was purified by flash chromatography (PE only) to afford **10g** (0.77 g, 89%) as a colorless syrup. TLC (PE): R_f = 0.92 (PE); ^{1}H NMR (300 MHz, $CDCl_3$) δ 7.96 (dd, *J* = 2.0, 1.0 Hz, 2H), 7.74 (d, *J* = 8.0 Hz, 2H), 7.31 – 7.27 (m, 2H), 2.85 – 2.75 (m, 4H), 1.79 – 1.67 (m, 4H), 1.44 (dt, *J* = 15.0, 7.5 Hz, 4H), 0.98 (t, *J* = 7.5 Hz, 6H); ^{13}C NMR (75 MHz, $CDCl_3$) δ 139.3, 137.2, 135.8, 127.6, 122.6, 121.2, 35.9, 34.3, 22.6, 14.2. The spectroscopic properties of **10g** were consistent with the data available in a literature.[7]

Synthesis of 2,8-bis(methoxymethyl)dibenzo[*b*,*d*]thiophene (10h)

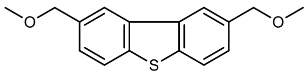

10h

Dibenzo[*b*,*d*]thiophene-2,8-diyldimethanol[8] (200 mg, 0.820 mmol) was dissolved in anhydrous THF (20 mL) under argon atmosphere, and then the solution was cooled to 0 °C. NaH (82 mg, 2.05 mmol) was added. The resulting mixture was then stirred at 0 °C for 20 min, then MeI (0.20 mL, 3.28 mmol) was added and the resulting solution was heated at reflux overnight. The reaction was cooled to room temperature then quenched by the addition of sat. NH_4Cl and diethyl ether. The resulting layers were separated and the aqueous layer was extracted with diethyl ether (3 × 10 mL). The combined organic layers were washed with brine, dried over Na_2SO_4, filtered, and the solvent was evaporated under reduced pressure to give the crude product, which was purified by flash chromatography (PE: EA = 5:1 to 2:1) to afford **10h** (156 mg, 70%) as a white solid. mp: 107-110 °C; TLC (PE:DCM, 3:5 V/V): R_f = 0.28; ^{1}H NMR (300 MHz, $CDCl_3$) δ 8.17 (dd, *J* = 2.0, 1.0 Hz,

2H), 7.88 – 7.78 (m, 2H), 7.44 (dd, J = 8.0, 2.0 Hz, 2H), 4.64 (s, 4H), 3.46 (s, 6H); ^{13}C NMR (75 MHz, $CDCl_3$) δ 139.2, 135.7, 134.7, 126.7, 122.9, 121.0, 74.8, 58.2; IR (film): 2926, 2820, 1474, 1437, 1377, 1271, 1232, 1192, 1150, 1092, 1067, 1028, 962. 912, 881, 806, 743, 719, 698, 681 cm^{-1}; ESI-HRMS (m/z): $[M+Na]^+$ calcd for $C_{16}H_{16}O_2NaS$: 295.0763; found: 295.0763.

Synthesis of 2,8-bis((allyloxy)methyl)dibenzo[*b*,*d*]thiophene (10i)

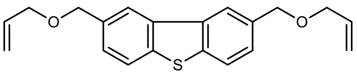

10i

Dibenzo[*b*,*d*]thiophene-2,8-diyldimethanol[8] (200 mg, 0.820 mmol) was dissolved in anhydrous THF (20 mL) under argon atmosphere, and then the solution was cooled to 0 °C. NaH (82 mg, 2.05 mmol) was added. The resulting mixture was then stirred at 0 °C for 20 min, then allyl bromide (0.28 mL, 3.28 mmol) was added and the resulting solution was heated at reflux overnight. The reaction was cooled to room temperature then quenched by the addition of brine and diethyl ether. The resulting layers were separated and the aqueous layer was extracted with diethyl ether (3 × 10 mL). The combined organic layers were washed with brine, dried over Na_2SO_4, filtered, and the solvent was evaporated under reduced pressure to give the crude product, which was purified by flash chromatography (PE: EA = 8:1 to 2:1) to afford **10i** (149 mg, 56%) as a colorless syrup. TLC (PE:DCM, 2:1 V/V): R_f = 0.28; ^1H NMR (300 MHz, $CDCl_3$) δ 8.26 – 8.03 (m, 2H), 7.83 (d, J = 8.0 Hz, 2H), 7.46 (dd, J = 8.0, 1.5 Hz, 2H), 6.02 (ddt, J = 17.0, 10.0, 5.5 Hz, 2H), 5.49 – 5.16 (m, 4H), 4.71 (s, 4H), 4.11 (dt, J = 5.5, 1.5 Hz, 4H); ^{13}C NMR (75 MHz, $CDCl_3$) δ 139.2, 135.7, 134.9, 134.8, 126.8, 122.9, 121.1, 117.5, 72.3, 71.3; IR (film): 2926, 2849, 1356, 1263, 1082, 1026, 930, 808, 744, 702 cm^{-1}; ESI-HRMS (m/z): $[M+Na]^+$ calcd for $C_{20}H_{20}O_2NaS$: 347.1076; found: 347.1075.

Synthesis of 2,8-dimethoxy-dibenzo[*b*,*d*]thiophene (10j)

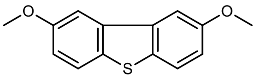

10j

This compound was prepared according to a literature procedure.[9] 2,8-Dibromo-dibenzo[*b*,*d*]thiophene (2.00 g, 5.85 mmol) was united with CuI (4.46 g, 23.4 mmol) and submitted to DMF (6.4 mL). A solution of MeONa/MeOH (5.4 M, 29 mL) was added under inert atmosphere in a three neck flask. The reactor was sealed and heated to 120 °C for 20 h. The crude is then filtered on a SiO_2 plug in ethyl acetate, and evaporated. The crude is then purified by flash SiO_2 gel column chromatography (PE:EA = 6:1 to 2:1) to give the product **10j** (1.19 g, 83%) as a white solid. TLC (PE:EA, 2:1 V/V): R_f = 0.67; ^1H NMR (300 MHz, $CDCl_3$) δ 7.70 (d, J = 8.5 Hz, 2H), 7.55 (d, J = 2.5 Hz, 2H), 7.09 (dd, J = 8.5, 2.5 Hz, 2H), 3.94 (s, 6H); ^{13}C NMR (75 MHz, $CDCl_3$) δ 157.6,

136.5, 132.5, 123.7, 115.9, 104.9, 55.8. The spectroscopic properties of **10j** were consistent with the data available in a literature.[10]

Synthesis of 1-(dibenzo[*b*,*d*]thiophen-2-yl)ethenone (10k)

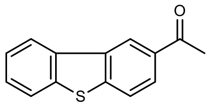

10k

10k was prepared according to a literature procedure. The spectroscopic properties of **10k** were consistent with the data available in the literature.[6]

Synthesis of dibenzo[*b*,*d*]thiophene 5-oxide (11a)

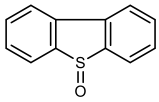

11a

Dibenzo[*b*,*d*]thiophene 5-oxide (**11a**) was synthesized according to **General procedure A:** DBT **10a** (1.00 g, 5.45 mmol), TFA (4.0 mL), 35% H_2O_2 (0.6 mL) were employed. Flash SiO_2 gel column chromatography (PE:EA = 4:1 to 2:1) afforded **11a** (1.01 g, 92%) as a colorless solid. ^{1}H NMR (400 MHz, $CDCl_3$) δ 8.00 (d, *J* = 7.5 Hz, 2H), 7.82 (d, *J* = 7.5 Hz, 2H), 7.61 (t, *J* = 7.5 Hz, 2H), 7.51 (t, *J* = 7.5 Hz, 2H); ^{13}C NMR (101 MHz, $CDCl_3$) δ 145.2, 137.2, 132.7, 129.7, 127.7, 122.0. The spectroscopic properties of **11a** were consistent with the data available in a literature.[11]

Synthesis of 4-methyldibenzo[*b*,*d*]thiophene 5-oxide (11b)

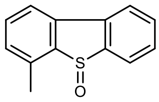

11b

4-Methyldibenzo[*b*,*d*]thiophene 5-oxide (**11b**) was synthesized according to **General procedure A: 10b** (540 mg, 2.70 mmol), TFA (2.0 mL), 35% H_2O_2 (0.3 mL) were employed. Flash SiO_2 gel column chromatography (PE:EA = 4:1 to 2:1) afforded **11b** (520 mg, 89%) as a colorless solid. ^{1}H NMR (300 MHz, $CDCl_3$) δ 7.98 (ddd, *J* = 7.5, 1.5, 1.0 Hz, 1H), 7.78 (ddd, *J* = 7.5, 1.5, 1.0 Hz, 1H), 7.67 – 7.54 (m, 2H), 7.54 – 7.42 (m, 2H), 7.24 (dt, *J* = 7.5, 1.0 Hz, 1H), 2.75 (s, 3H); ^{13}C NMR (75 MHz, $CDCl_3$) δ 144.8, 143.3, 139.5, 137.5, 137.4, 132.9, 132.5, 131.2, 129.5, 127.5, 122.1, 119.5, 18.9. The spectroscopic properties of **11b** were consistent with the data available in a literature.[12]

Synthesis of 4,6-dimethyldibenzo[*b*,*d*]thiophene 5-oxide (11c)

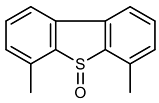

11c

4,6-Dimethyldibenzo[*b*,*d*]thiophene 5-oxide (**11c**) was synthesized according to **General procedure A: 10c** (1.04 g, 4.90 mmol), TFA (3.60 mL), 35% H_2O_2 (0.54 mL) were employed. Flash SiO_2 gel column chromatography (PE:EA = 4:1 to 1:1) afforded **11c** (590 mg, 53%) as a colorless solid. ^1H NMR (300 MHz, $CDCl_3$) δ 7.60 (dt, *J* = 7.5, 1.0 Hz, 2H), 7.46 (t, *J* = 7.5 Hz, 2H), 7.23 (dt, *J* = 7.5, 1.0 Hz, 2H), 2.74 (s, 6H); ^{13}C NMR (75 MHz, $CDCl_3$) δ 142.8, 139.2, 137.7, 132.7, 131.0, 119.6, 18.8. The spectroscopic properties of **11c** were consistent with the data available in a literature.[13]

Synthesis of 3,7-dimethyldibenzo[*b*,*d*]thiophene 5-oxide (11d)

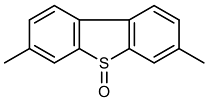

11d

3,7-Dimethyldibenzo[*b*,*d*]thiophene 5-oxide (**11d**) was synthesized according to **General procedure A: 10d** (350 mg, 1.65 mmol), TFA (1.2 mL), 35% H_2O_2 (0.2 mL) were employed. Flash SiO_2 gel column chromatography (PE:EA = 4:1 to 2:1) afforded **11d** (361 mg, 96%) as a colorless solid. ^1H NMR (300 MHz, $CDCl_3$) δ 7.81 – 7.74 (m, 2H), 7.65 (d, *J* = 8.0 Hz, 2H), 7.38 (ddd, *J* = 8.0, 1.5, 1.0 Hz, 2H), 2.46 (s, 6H). ^{13}C NMR (75 MHz, $CDCl_3$) δ 145.2, 139.7, 134.8, 133.4, 128.0, 121.5, 21.6. The spectroscopic properties of **11d** were consistent with the data available in a literature.[14]

Synthesis of 2-acetyldibenzo[*b*,*d*]thiophene 5-oxide (11k)

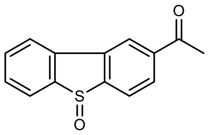

11k

2-Acetyldibenzo[*b*,*d*]thiophene 5-oxide (**11k**) was synthesized according to **General procedure A: 10k** (100 mg, 0.44 mmol), TFA (0.33 mL), 35% H_2O_2 (0.53 mL) were employed. Flash SiO_2 gel column chromatography (PE:EA = 4:1 to 1:1) afforded **11k** (71.0 mg, 67%) as a colorless solid. ^1H NMR (300 MHz, $CDCl_3$) δ 8.39 (dd, *J* = 1.5, 1.0 Hz, 1H), 8.13 – 8.06 (m, 2H), 8.06 – 8.00 (m, 1H), 7.92 (ddd, *J* = 7.5, 1.5, 1.0 Hz, 1H), 7.67 (tt, *J* = 7.5, 1.0 Hz, 1H), 7.57 (tt, *J* = 7.5, 1.0 Hz, 1H), 2.72 (d, *J* = 1.0 Hz, 3H); ^{13}C NMR (75 MHz, $CDCl_3$) δ 197.0, 149.3, 145.5, 140.5, 137.9, 136.4, 133.0, 130.3, 129.6, 127.9, 127.8, 122.5, 121.5, 27.2; ESI-HRMS (m/z): $[M+H]^+$calcd for $C_{14}H_{11}O_2S$: 243.0474; found: 243.0474.

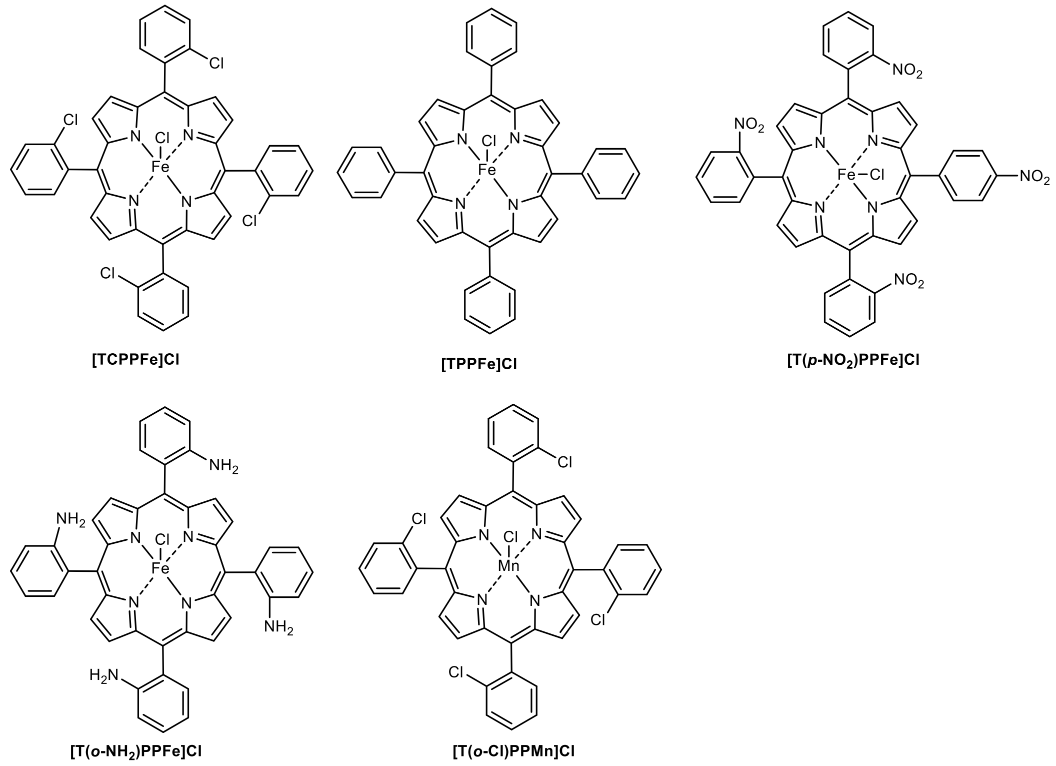

Supplementary Figure 2. Porphyrin Catalysts involved in this study. [TCPPFe]Cl, [TPPFe]Cl, [T(*p*-NO_2)PPFe]Cl, [T(*o*-NH_2)PPFe]Cl, [TCPPMn]Cl were synthesized according to the reported procedures.[15-17] **[TPPFe]Cl**: *meso*-tetraphenylporphinatoiron (III) chloride; **[TCPPFe]Cl**: (5,10,15,20-Tetrakis(2-chlorophenyl) porphyrinato) iron (III) chloride); **[T(*o*-NH_2)PPFe]Cl**: (5,10,15,20-Tetrakis(2-amino) porphyrinato) iron (III) chloride); **[T(*o*-NO_2)PPFe]Cl**: (5,10,15,20-Tetrakis(2-nitro) porphyrinato) iron (III) chloride); **[TCPPMn]Cl**: (5,10,15,20-Tetrakis(2-chlorophenyl) porphyrinato)manganese (III) chloride.

Synthesis of dibenzo[*c*,*e*][1,2]oxathiine 6-oxide (13a) from 10a

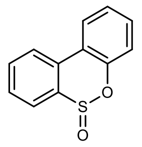

13a

Dibenzo[*c*,*e*][1,2]oxathiine 6-oxide (**13a**) was synthesized according to **General procedure B:** DBT **10a** (27.6 mg, 0.15 mmol) and *t*-BuOOH (160 μL, 6.0 equiv., 1.0 equiv. every 30 min) were employed. After stirring at 30 ˚C for 3 h, purification of the product by flash SiO_2 gel column chromatography (PE: EA = 10:1) afforded **13a** (27.9 mg, 86%) as a white solid. TLC (PE:EA, 2:1 V/V): R_f = 0.16; ^{1}H NMR (400 MHz $CDCl_3$) δ 8.04 (d, *J* = 8.0 Hz, 1H), 8.00 (d, *J* = 8.0 Hz, 1H), 7.73 – 7.70 (m, 2H), 7.55 (t, *J* = 7.5 Hz, 1H), 7.47 (m, t, *J* = 7.0 Hz, 1H), 7.37 (t, *J* = 7.5 Hz, 2H); ^{13}C NMR (101 MHz, $CDCl_3$) δ 145.0, 137.2, 133.2, 130.9, 128.6, 126.6, 125.8, 125.6, 124.7, 124.6, 121.6, 120.6; IR (KBr): 3065, 2925, 1584. 1474, 1451, 1426, 1289, 1237, 1180, 1142, 1122, 1107, 843,

758 cm^{-1}. ESI-HRMS (m/z): $[M+H]^+$ calcd for $C_{12}H_9O_2{}^{32}S$: 217.0323; found: 217.0309. The spectroscopic properties of **13a** were consistent with the data available in a literature.[18] (For HPLC traces, see Supplementary Figures 3-9).

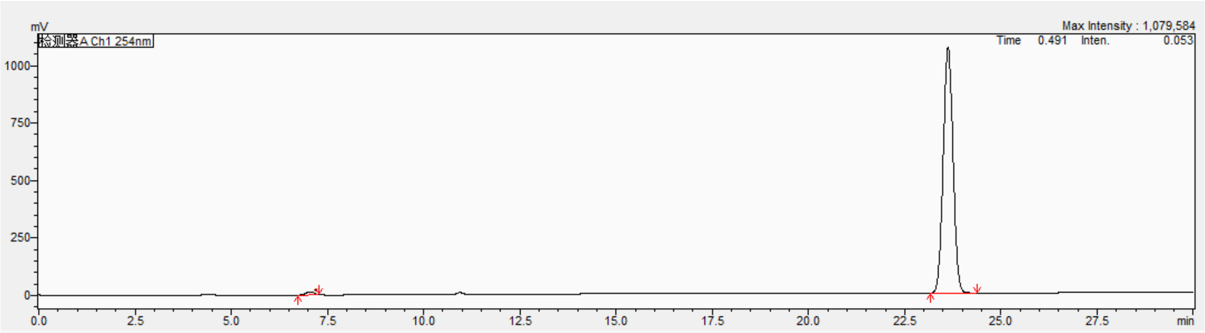

Peak No.	Retention time	Area	Area (%)	Height	Height (%)	S/N
1	7.089	206937	1.082	12462	1.152	36.73
2	23.629	18914658	98.918	1069246	98.848	3151.49
Total		19121596	100.00	1081708	100	

Supplementary Figure 3. The reaction progress monitored by HPLC (from DBT). HPLC trace at 0 h. Retention time: **11a** 7.1 min (1.1%);**10a** 23.6 min (98.9%).

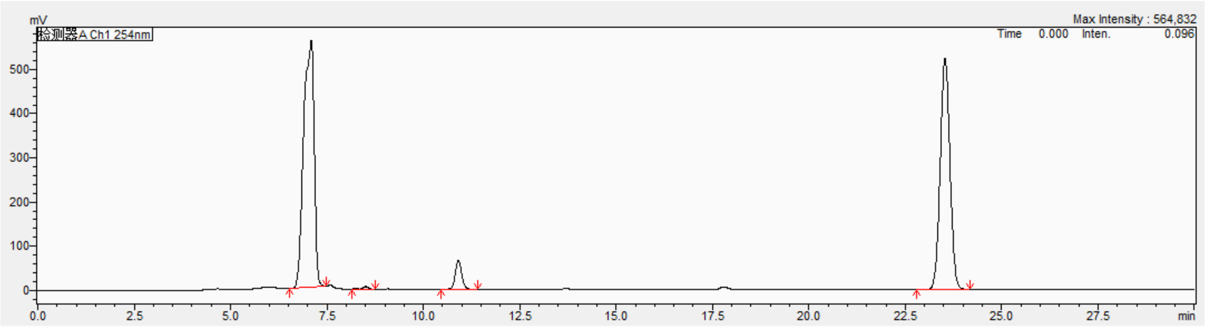

Peak No.	Retention time	Area	Area (%)	Height	Height (%)	S/N
1	7.096	10593763	51.262	557457	48.333	924.05
2	8.503	70635	0.342	5986	0.519	9.92
3	10.911	778624	3.768	66020	5.724	109.44
4	23.534	9222779	44.628	523904	45.424	868.43
Total		20665801	100.00	1153367	100.00	

Supplementary Figure 4. The reaction progress monitored by HPLC (from DBT). HPLC trace at 0.5 h. Retention time: **11a** 7.1 min (51.3% yield); **12a** 8.5 min (0.3% yield); **13a** 10.9 min (3.8% yield); **10a** 23.5 min (44.6%) (Conversion rate 55.4%).

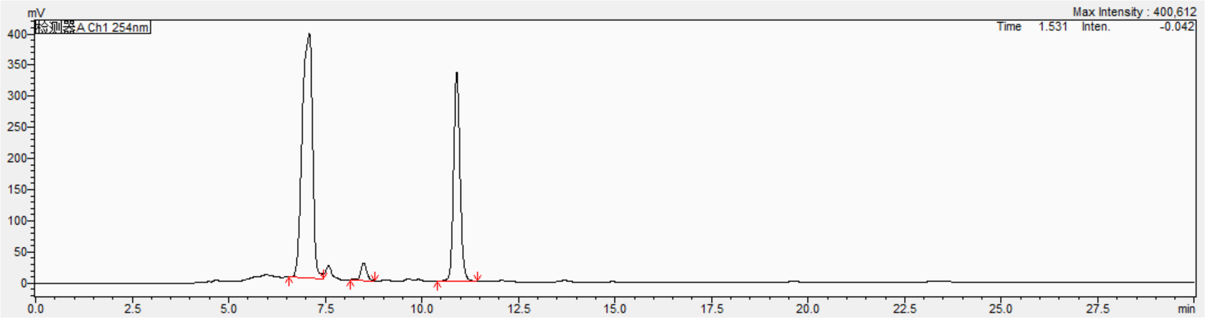

Peak No.	Retention time	Area	Area (%)	Height	Height (%)	S/N
1	7.089	7307309	63.492	392514	51.938	457.56
2	8.492	305696	2.656	28314	3.747	33.01
3	10.903	3896093	33.852	334903	44.315	390.40
Total		11509097	100.00	755732	100.00	

Supplementary Figure 5. The reaction progress monitored by HPLC (from DBT). HPLC trace at 1.0 h. Retention time: **11a** 7.1 min (63.5% yield); **12a** 8.5 min (2.7% yield); **13a** 10.9 min (33.9% yield); **10a** 23.5 min (Conversion rate >99%).

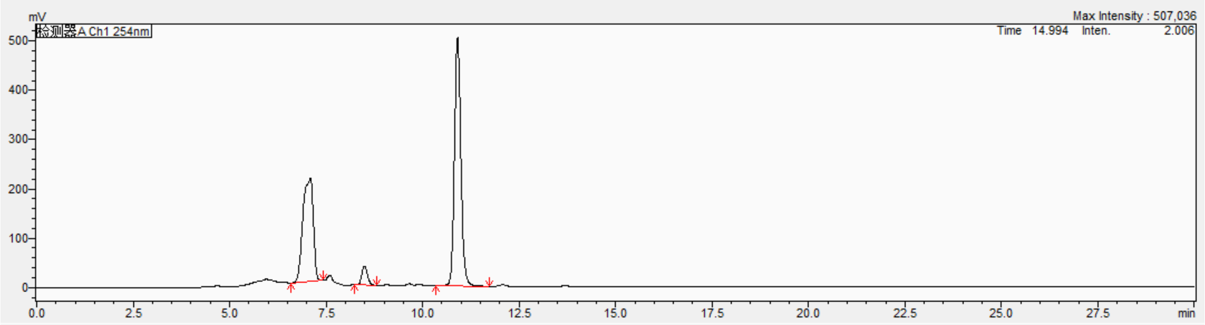

Peak No.	Retention time	Area	Area (%)	Height	Height (%)	S/N
1	7.092	3921920	38.658	209184	27.887	257.90
2	8.494	368681	3.634	37432	4.990	46.15
3	10.906	5854685	57.708	503489	67.123	620.75
Total		10145286	100.00	750104	100.00	

Supplementary Figure 6. The reaction progress monitored by HPLC (from DBT). HPLC trace at 1.5 h. Retention time: **11a** 7.1 min (38.7% yield); **12a** 8.5 min (3.6% yield); **13a** 10.9 min (57.7% yield).

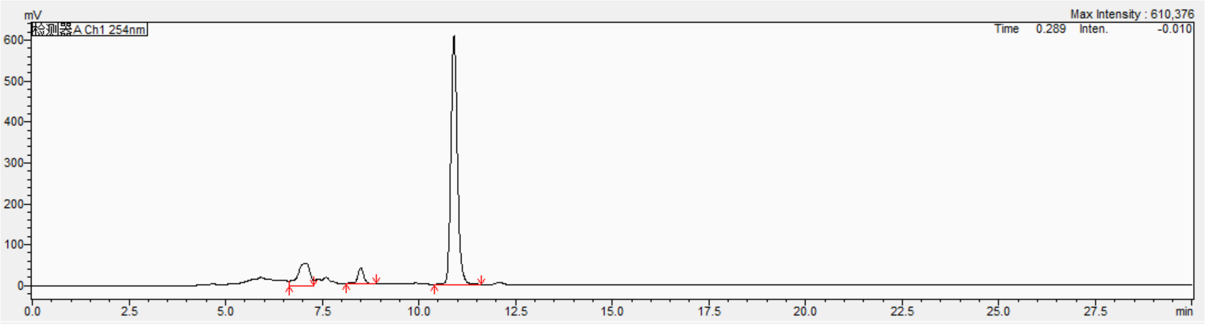

Peak No.	Retention time	Area	Area (%)	Height	Height (%)	S/N
1	7.090	1188719	13.641	53872	7.708	70.92
2	8.495	400950	4.601	37812	5.410	49.78
3	10.908	7124805	81.758	607182	86.881	799.36
Total		8714475	100.00	698866	100.00	

Supplementary Figure 7. The reaction progress monitored by HPLC (from DBT). HPLC trace at 2.0 h. Retention time: **11a** 7.1 min (13.7% yield); **12a** 8.5 min (4.6% yield); **13a** 10.9 min (81.8% yield).

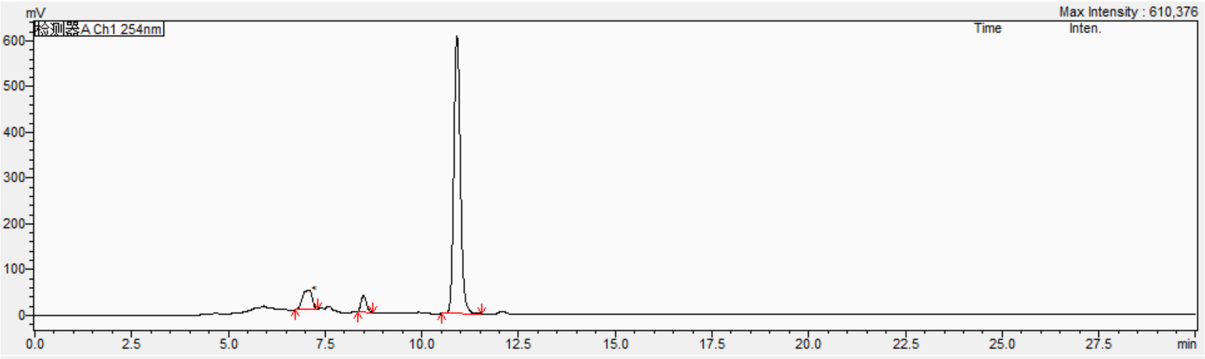

Peak No.	Retention time	Area	Area (%)	Height	Height (%)	S/N
1	7.090	717802	8.813	41501	6.070	49.64
2	8.495	331950	4.076	35546	5.199	42.52
3	10.908	7094605	87.111	606674	88.731	725.65
Total		8144357	100.00	683721	100.00	

Supplementary Figure 8. The reaction progress monitored by HPLC (from DBT). HPLC trace at 2.5 h. Retention time: **11a** 7.1 min (8.8% yield); **12a** 8.5 min (4.1% yield); **13a** 10.9 min (91.4% yield).

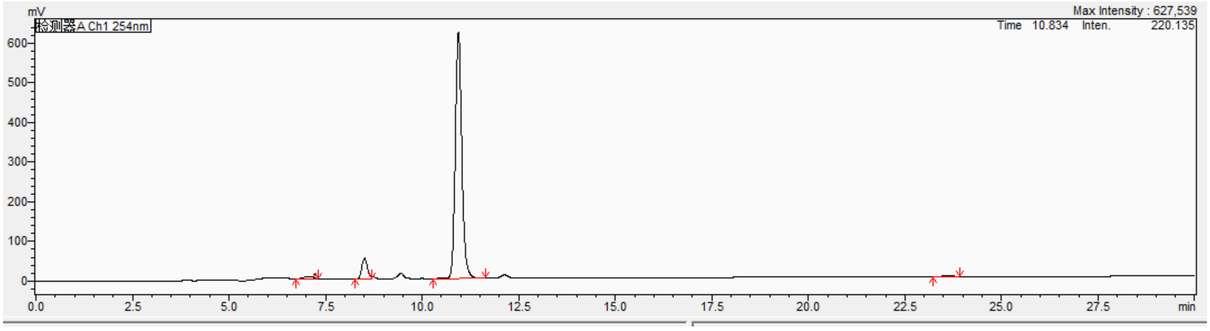

Peak No.	Retention time	Area	Area (%)	Height	Height (%)	S/N
1	7.112	91374	1.142	5476	0.803	6.71
2	8.508	524280	6.552	51816	7.600	63.51
3	10.940	7315313	91.414	620579	91.017	760.61
4	23.621	71442	0.893	3957	0.580	4.85
Total		8002409	100.00	681827	100.00	

Supplementary Figure 9. The reaction progress monitored by HPLC (from DBT). HPLC trace at 3.0 h. Retention time: **11a** 7.1 min (1.1% yield); **12a** 8.5 min (6.6% yield); **13a** 10.9 min (91.4% yield). **10a** 23.6 min (0.9%).

Synthesis of dibenzo[*c*,*e*][1,2]oxathiine 6-oxide (**13a) from 11a**

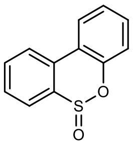

13a

Dibenzo[*c*,*e*][1,2]oxathiine 6-oxide (**13a**) was synthesized according to **General procedure C: 11a** (30.0 mg, 0.15 mmol) and *t*-BuOOH (110 μL, 4.0 equiv.; 1.0 equiv. every 30 min) were employed. After stirring at 30 °C for 2 h, purification of the product by flash SiO_2 gel column chromatography (PE: EA = 10:1) afforded **13a** (27.3 mg, 84%) as a white solid. (For HPLC traces, see Supplementary Figures 10-14).

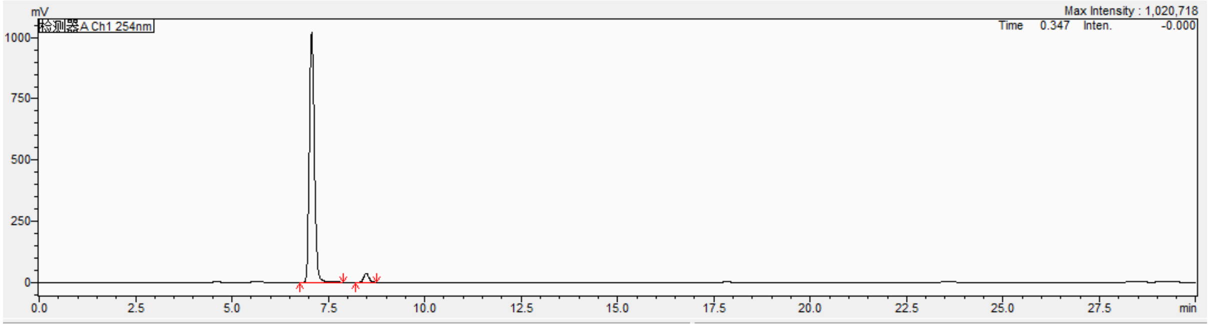

Peak No.	Retention time	Area	Area (%)	Height	Height (%)	S/N
1	7.070	9346814	96.567	1019779	96.597	6775.25
2	8.484	332323	3.433	35923	3.403	238.67
Total		9679137	100.00	1055702	100.00	

Supplementary Figure 10. The reaction progress monitored by HPLC (from DBTO). HPLC trace at 0 h. Retention time: **11a** 7.1 min (96.6%); **12a** 8.5 min (3.4%).

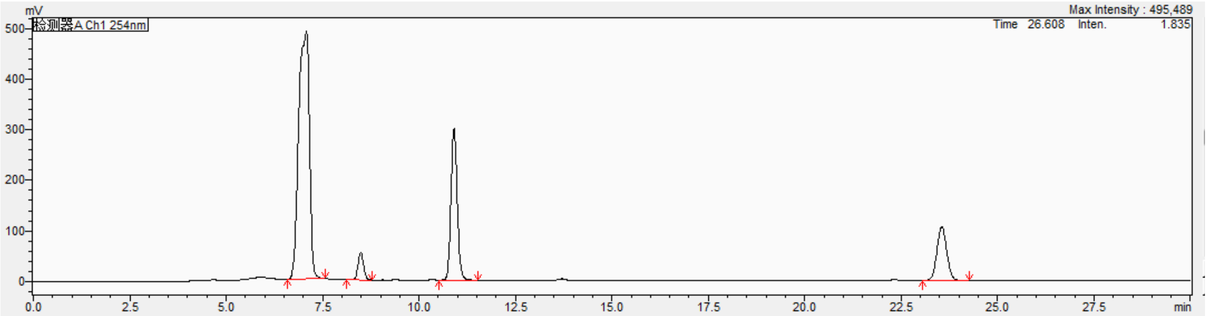

Peak No.	Retention time	Area	Area (%)	Height	Height (%)	S/N
1	7.082	9499707	61.884	490455	51.568	1305.54
2	8.489	546470	3.560	54042	5.682	143.85
3	10.907	3434267	22.372	300015	31.545	798.61
4	23.555	1870373	12.184	106570	11.205	283.68
Total		15350816	100.00	951082	100.00	

Supplementary Figure 11. The reaction progress monitored by HPLC (from DBTO). HPLC trace at 0.5 h. Retention time: **11a** 7.1 min (61.9%) (Conversion rate 38.1%); **12a** 8.5 min (3.6% yield); **13a** 10.9 min (22.4% yield); **10a** 23.6 min (12.2%).

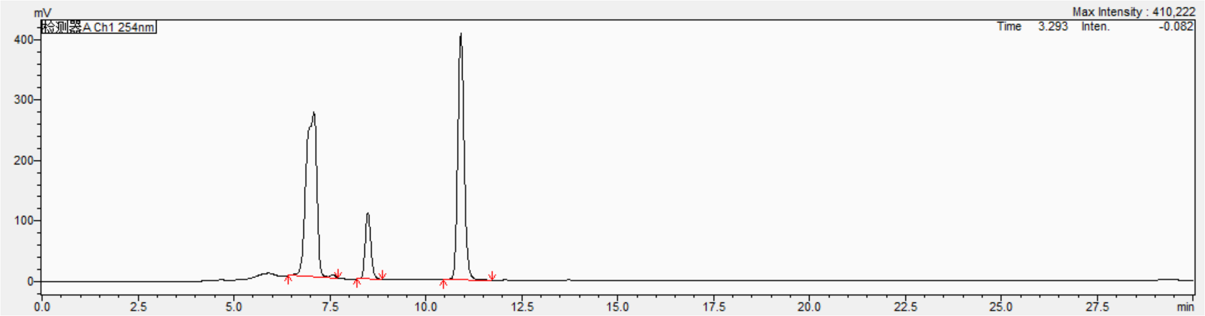

Peak No.	Retention time	Area	Area (%)	Height	Height (%)	S/N
1	7.088	5303283	47.569	272122	34.465	931.19
2	8.492	1106531	9.925	109643	13.887	375.20
3	10.914	4738887	42.506	407802	51.649	1395.48
Total		11148701	100.00	789567	100.00	

Supplementary Figure 12. The reaction progress monitored by HPLC (from DBTO). HPLC trace at 1.0 h. Retention time: **11a** 7.1 min (47.6%) (Conversion rate 52.4%); **12a** 8.5 min (9.9% yield); **13a** 10.9 min (42.5% yield).

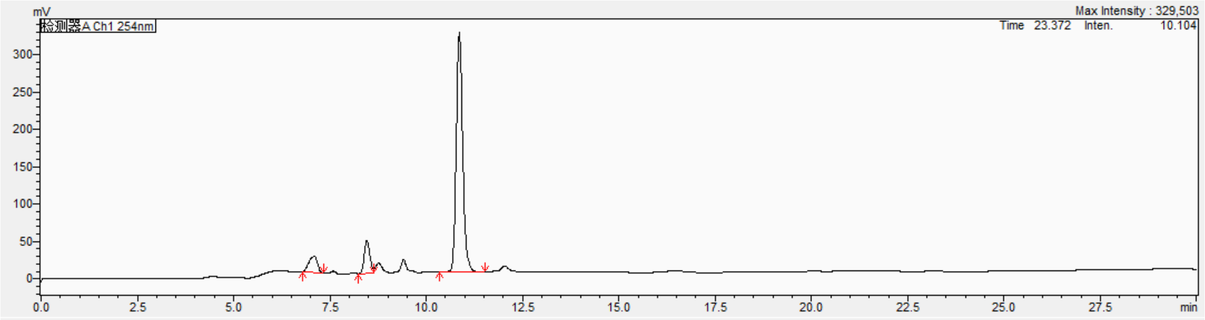

Peak No.	Retention time	Area	Area (%)	Height	Height (%)	S/N
1	7.089	329316	7.272	21651	5.617	19.90
2	8.459	446366	9.856	43535	11.295	40.02
3	10.858	3753146	82.872	320256	83.088	294.39
Total		4528829	100.00	385443	100.00	

Supplementary Figure 13. The reaction progress monitored by HPLC (from DBTO). HPLC trace at 1.5 h. Retention time: **11a** 7.1 min (7.3%) (Conversion rate 92.7%); **12a** 8.5 min (9.9% yield); **13a** 10.9 min (82.8% yield).

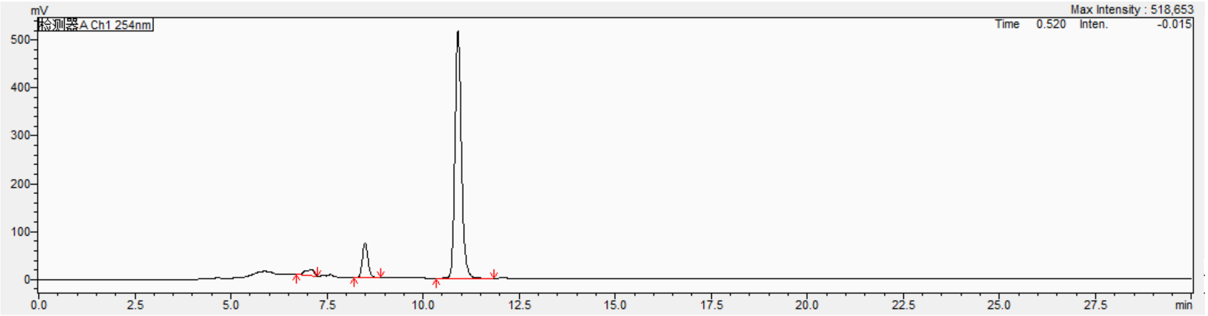

Peak No.	Retention time	Area	Area (%)	Height	Height (%)	S/N
1	7.099	186393	2.657	12906	2.146	27.49
2	8.493	717664	10.232	72351	12.030	154.11
3	10.911	6110068	87.111	516189	85.825	1099.50
Total		7014125	100.00	601447	100.00	

Supplementary Figure 14. The reaction progress monitored by HPLC (from DBTO). HPLC trace at 2.0 h. Retention time: **11a** 7.1 min (2.7%) (Conversion rate 97.3%); **12a** 8.5 min (10.2% yield); **13a** 10.9 min (87.1% yield).

Synthesis of 7-methyldibenzo[*c*,*e*][1,2]oxathiine 6-oxide (13b) and 4-methyldibenzo[*c*,*e*][1,2]oxathiine 6-oxide (13b') from 10b

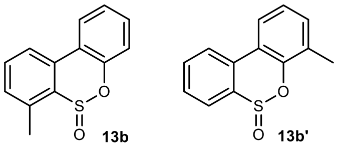

(**13b**:**13b'** = 1:1) (inseparable mixture)

7-Methyldibenzo[*c*,*e*][1,2]oxathiine 6-oxide (**13b**) and 4-methyldibenzo[*c*,*e*][1,2]oxathiine 6-oxide (**13b'**) were synthesized according to **General procedure B**: **10b** (29.7 mg, 0.15 mmol) and *t*-BuOOH (160 μL, 6.0 equiv.) were employed. After stirring at 30 ˚C for 2.5 h, purification of the product by flash SiO_2 gel column chromatography (PE: EA = 10:1) afforded **13b** and **13b'** (30.1 mg, 87%) as a white solid. TLC (PE:EA, 2:1 V/V): R_f = 0.17; ^1H NMR (300 MHz, $CDCl_3$) δ 7.91 (ddd, *J* = 15.5, 8.0, 2.0 Hz, 1H), 7.82 – 7.69 (m, 1H), 7.61 (t, *J* = 7.0 Hz, 1H), 7.53 – 7.33 (m, 1.5H), 7.33 – 7.11 (m, 2.5H), 2.63 (s, 1.35H), 2.36 (s, 1.65H); ^{13}C NMR (75 MHz, $CDCl_3$) δ 144.3, 143.3, 137.0, 136.2, 135.7, 133.1, 132.4, 132.3, 131.0, 130.8, 130.6, 128.4, 127.0 (2), 125.7, 125.6, 125.0 (2), 124.9, 122.4 (2), 121.4, 121.1, 120.4, 17.8, 16.3; IR (film): 2959, 2910, 1732, 1450, 1379, 1130, 1097, 1053, 1024, 762 cm^{-1}. APCI-HRMS (m/z): $[M+H]^+$ calcd for $C_{13}H_{11}O_2{}^{32}S$: 231.0474; found: 231.0472.

Synthesis of 7-methyldibenzo[*c,e*][1,2]oxathiine 6-oxide (13b) and 4-methyl-dibenzo[*c,e*][1,2]oxathiine 6-oxide (13b') from 11b

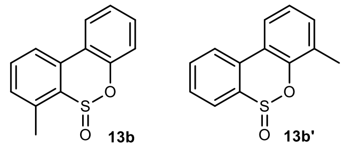

(**13b**:**13b'** = 1:1) (inseparable mixture)

7-Methyldibenzo[*c,e*][1,2]oxathiine 6-oxide (**13b**) and 4-methyldibenzo[*c,e*][1,2]oxathiine 6-oxide (**13b'**) were synthesized according to **General procedure C**: **11b** (32.1 mg, 0.15 mmol) and *t*-BuOOH (110 μL, 4.0 equiv.) were employed. After stirring at 30 ˚C for 2 h, purification of the product by flash SiO_2 gel column chromatography (PE: EA = 10:1) afforded **13b** and **13b'** (29.7 mg, 86%) as a white solid.

Synthesis of 4,7-dimethyldibenzo[*c,e*][1,2]oxathiine 6-oxide (13c) from 10c

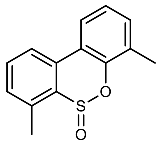

4,7-Dimethyldibenzo[*c,e*][1,2]oxathiine 6-oxide (**13c**) was synthesized according to **General procedure B: 10c** (31.8 mg, 0.15 mmol), *t*-BuOOH (160 μL, 6.0 equiv.) and [TCPPFe]Cl (4.00 mg, 3.2 mol%) were employed. After stirring at 30 ˚C for 2.5 h, a second portion of *t*-BuOOH (160 μL, 6.0 equiv.) and [TCPPFe]Cl (3.10 mg, 2.5 mol%), the reaction was stirred at 30 ˚C for another 2.5 h, purification of the product by flash SiO_2 gel column chromatography (PE: DCM = 10:1 to 2:1) afforded **13c** (20.2 mg, 55%) as a white solid. mp: 115-117 ˚C; TLC (PE:DCM, 2:1 V/V): R_f = 0.22; ^{1}H NMR (300 MHz, $CDCl_3$) δ 7.90 – 7.83 (m, 1H), 7.81 (dd, *J* = 8.0, 1.5 Hz, 1H), 7.56 (t, *J* = 8.0 Hz, 1H), 7.37 – 7.27 (m, 2H), 7.23 (t, *J* = 7.5 Hz, 1H), 2.72 (s, 3H), 2.46 (s, 3H); ^{13}C NMR (75 MHz, $CDCl_3$) δ 142.7, 136.1, 135.5, 132.3, 132.0, 130.7, 130.6, 127.4, 125.0, 122.7, 122.6, 120.8, 17.8, 16.2; IR (film): 2935, 2873, 1722, 1578, 1458, 1192, 1169, 1124, 1084, 845, 800, 775, 762, 694 cm^{-1}; APCI-HRMS (m/z): $[M+H]^+$ calcd for $C_{14}H_{13}O_2S$: 245.0631; found: 245.0630.

Synthesis of 4,7-dimethyldibenzo[*c,e*][1,2]oxathiine 6-oxide (13c) from 11c

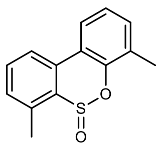

4,7-Dimethyldibenzo[*c*,*e*][1,2]oxathiine 6-oxide (**13c**) was synthesized according to **General procedure C: 11c** (34.2 mg, 0.15 mmol) and *t*-BuOOH (110 μL, 4.0 equiv.) and [TCPPFe]Cl (4.00 mg, 3.2 mol%) were employed. After stirring at 30 ˚C for 2 h, a second portion of *t*-BuOOH (110 μL, 4.0 equiv.) and [TCPPFe]Cl (3.10 mg, 2.5 mol%), the reaction was stirred at 30 °C for another 2.5 h, purification of the product by flash SiO_2 gel column chromatography (PE: DCM = 10:1 to 2:1) afforded **13c** (17.6 mg, 48%) as a white solid.

Synthesis of 3,8-dimethyldibenzo[*c*,*e*][1,2]oxathiine 6-oxide (13d) from 10d

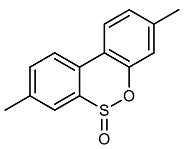

13d

3,8-Dimethyldibenzo[*c*,*e*][1,2]oxathiine 6-oxide (**13d**) was synthesized according to **General procedure B: 10d** (31.8 mg, 0.15 mmol) and *t*-BuOOH (160 μL, 6.0 equiv.) were employed. After stirring at 30 ˚C for 2.5 h, purification of the product by flash SiO_2 gel column chromatography (PE: DCM = 10:1 to 2:1) afforded **13d** (23.1 mg, 63%) as a white solid. TLC (PE:DCM, 2:1 V/V): R_f = 0.22; ^{1}H NMR (300 MHz, $CDCl_3$) δ 7.92 – 7.86 (m, 1H), 7.84 (d, *J* = 8.0 Hz, 1H), 7.53 – 7.44 (m, 2H), 7.14 (dt, *J* = 8.5, 1.0 Hz, 2H), 2.48 (s, 3H), 2.42 (s, 3H); ^{13}C NMR (75 MHz, $CDCl_3$) δ 144.7, 141.2, 138.6, 136.8, 134.0, 126.6, 126.1, 124.2 (2), 121.8, 118.0, 110.1, 21.5, 21.4; IR (film): 2930, 2875, 1262, 1137, 1119, 933, 814, 746, 719, 700 cm^{-1}; APCI-HRMS: m/z calcd for $C_{14}H_{13}O_2S[M+H]^+$: 245.0631; found: 245.0630.

Synthesis of 3,8-dimethyldibenzo[*c*,*e*][1,2]oxathiine 6-oxide (13d) from 11d

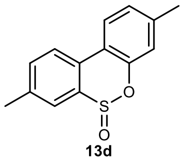

13d

3,8-Dimethyldibenzo[*c*,*e*][1,2]oxathiine 6-oxide (**13d**) was synthesized according to **General procedure C: 11d** (34.2 mg, 0.15 mmol) and *t*-BuOOH (110 μL, 4.0 equiv.) were employed. After stirring at 30 ˚C for 2.5 h, purification of the product by flash SiO_2 gel column chromatography (PE: DCM = 10:1 to 2:1) afforded **13d** (21.6 mg, 59%) as a white solid.

Synthesis of 2,9-dimethyldibenzo[*c*,*e*][1,2]oxathiine 6-oxide (13e) from 10e

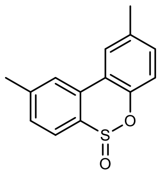

13e

2,9-Dimethyldibenzo[*c*,*e*][1,2]oxathiine 6-oxide (**13e**) was synthesized according to **General procedure B: 10e** (31.8 mg, 0.15 mmol) and *t*-BuOOH (160 μL, 6.0 equiv.) were employed. After stirring at 30 °C for 2.5 h, purification of the product by flash SiO_2 gel column chromatography (PE: DCM = 10:1 to 2:1) afforded **13e** (30.0 mg, 82%) as a white solid. mp: 136-138 °C; TLC (PE:DCM, 2:1 V/V): R_f = 0.22; ^{1}H NMR (300 MHz, $CDCl_3$) δ 7.85 – 7.75 (m, 2H), 7.59 (d, *J* = 8.0 Hz, 1H), 7.33 (ddd, *J* = 8.0, 1.5, 1.0 Hz, 1H), 7.25 (t, *J* = 1.0 Hz, 2H), 2.53 (s, 3H), 2.45 (s, 3H); ^{13}C NMR (75 MHz, $CDCl_3$) δ 143.6, 142.9, 135.1, 134.7, 131.4, 129.1, 126.6, 125.8, 125.0, 124.9, 121.2, 120.3, 22.1, 21.2; IR (film): 2926, 1605, 1263, 1180, 1151, 1121, 895, 816, 746, 712, 700, 677 cm^{-1}; APCI-HRMS (m/z): [M+H]$^+$ calcd for $C_{14}H_{13}O_2S$: 245.0631; found: 245.0630.

Synthesis of 9-(1-Hydroxyethyl) dibenzo[*c*,*e*][1,2]oxathiine 6-oxide (13f) and 2-(1-hydroxyethyl) dibenzo[*c*,*e*][1,2]oxathiine 6-oxide (13f') from 10f

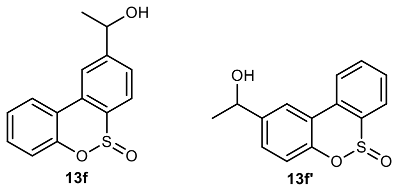

13f **13f'**

(**13f**:**13f'** = 1:1) (inseparable mixture)

9-(1-Hydroxyethyl) dibenzo[*c*,*e*][1,2]oxathiine 6-oxide (**13f**) and 2-(1-hydroxyethyl) dibenzo [*c*,*e*][1,2]oxathiine 6-oxide (**13f'**) were synthesized according to **General procedure B: 10f** (34.2 mg, 0.15 mmol) and *t*-BuOOH (160 μL, 6.0 equiv.) were employed. After stirring at 30 °C for 2.5 h, purification of the product by flash SiO_2 gel column chromatography (PE: EA = 8:1 to 1:2) afforded **13f** and **13f'** (31.2 mg, 80%) as a colorless syrup. TLC (PE:EA, 2:1 V/V): R_f = 0.18; ^{1}H NMR (300 MHz, $CDCl_3$) δ 8.10– 8.02 (m, 2H), 7.78 – 7.65 (m, 2H), 7.61 – 7.41 (m, 2H), 7.39 – 7.32 (m, 1H), 5.11 – 4.96 (m, 1H), 1.56 (d, *J* = 6.5 Hz, 3H); ^{13}C NMR (75 MHz, $CDCl_3$) δ 143.3, 133.2, 131.0, 128.7, 128.2, 128.0, 126.1, 125.9, 125.6, 124.8, 121.7, 121.6, 70.1 (2), 25.6; IR (film): 3423, 2978, 2924, 1591, 1566, 1483, 1437, 1412, 1369, 1290, 1236, 1182, 1139, 1121, 1092, 1074, 1038, 910, 891, 858, 798, 769, 685 cm^{-1}; APCI-HRMS (m/z): [M+H]$^+$calcd for $C_{14}H_{13}O_3S$: 261.0580; found: 261.0578.

Synthesis of 2,9-dibutyldibenzo[*c*,*e*][1,2]oxathiine 6-oxide (13g) from 10g

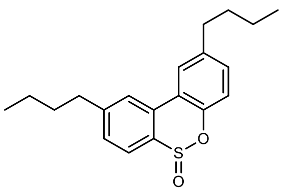

13g

2,9-Dibutyldibenzo[*c*,*e*][1,2]oxathiine 6-oxide (**13g**) was synthesized according to **General procedure B: 10g** (44.5 mg, 0.15 mmol) and *t*-BuOOH (160 μL, 6.0 equiv.) were employed. After stirring at 30 °C for 2.5 h, purification of the product by flash SiO_2 gel column chromatography (PE: DCM = 8:1 to 1:1) afforded **13g** (36.5 mg, 74%) as a colorless syrup. TLC (PE:DCM, 2:1 V/V): R_f = 0.48; ^{1}H NMR (300 MHz, $CDCl_3$) δ 7.86 – 7.76 (m, 2H), 7.62 (d, *J* = 8.0 Hz, 1H), 7.35 (dd, *J* = 8.0, 1.5 Hz, 1H), 7.29 – 7.25 (m, 2H), 2.79 (t, *J* = 8.0 Hz,2H), 2.72 (t, *J* = 7.5 Hz, 2H), 1.79 – 1.60 (m, 4H), 1.51 – 1.34 (m, 4H), 1.06 – 0.91 (m, 6H); ^{13}C NMR (75 MHz, $CDCl_3$) δ 148.6, 143.0, 140.2, 134.8, 130.8, 128.5, 126.7, 125.9, 124.4, 124.3, 121.3, 120.4, 36.2, 35.5, 34.0, 33.7, 22.5 (2), 14.1 (2); IR (film): 2961, 2930, 2872, 1599, 1564, 1458, 1410, 1263, 1188, 1148, 1120, 893, 822, 744, 719, 696 cm^{-1}; APCI-HRMS (m/z): $[M+H]^+$ calcd for $C_{20}H_{25}O_2S$: 329.1570; found: 329.1570.

Synthesis of 2,9-bis(methoxymethyl)dibenzo [*c*,*e*][1,2]oxathiine 6-oxide (13h) from 10h

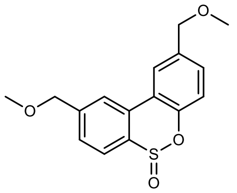

13h

2,9-Bis(methoxymethyl)dibenzo[*c*,*e*][1,2]oxathiine 6-oxide (**13h**) was synthesized according to **General procedure B: 10h** (40.9 mg, 0.15 mmol) and *t*-BuOOH (160 μL, 6.0 equiv.) were employed. After stirring at 30 °C for 2.5 h, purification of the product by flash SiO_2 gel column chromatography (PE: DCM = 8:1 to 1:2) afforded **13h** (32.0 mg, 70%) as a colorless syrup. TLC (PE:EA, 2:1 V/V): R_f = 0.21; ^{1}H NMR (300 MHz, $CDCl_3$) δ 8.05 (br s, 1H), 8.01 (d, *J* = 1.5 Hz, 1H), 7.70 (d, *J* = 8.0 Hz, 1H), 7.54 – 7.47 (m, 1H), 7.43 (dd, *J* = 8.5, 2.0 Hz, 1H), 7.34 (d, *J* = 8.5 Hz, 1H), 4.62 (s, 2H), 4.55 (s, 2H), 3.47 (s, 3H), 3.45 (s, 3H); ^{13}C NMR (75 MHz, $CDCl_3$) δ 144.5, 144.0, 136.4, 135.8, 130.2, 127.3, 126.8, 126.0, 124.0, 123.3, 121.5, 120.6, 74.1, 74.0, 58.7, 58.4; IR (film): 2926, 2854, 1493, 1454, 1410, 1364, 1265, 1190, 1146, 1130, 1101, 887, 817, 748, 717, 683 cm^{-1}; APCI-HRMS (m/z): $[M+H]^+$ calcd for $C_{16}H_{17}O_4S$: 305.0842; found: 305.0842.

Synthesis of 2,9-bis((allyloxy)methyl)dibenzo[*c*,*e*] [1,2]oxathiine 6-oxide (13i) from 10i

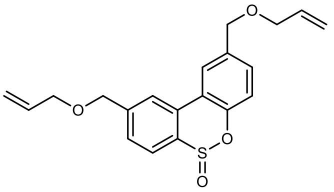

13i

2,9-Bis((allyloxy)methyl)dibenzo[*c*,*e*] [1,2]oxathiine 6-oxide (**13i**) was synthesized according to **General procedure B: 10i** (48.7 mg, 0.15 mmol) and *t*-BuOOH (160 μL, 6.0 equiv.) were employed. After stirring at 30 °C for 2.5 h, purification of the product by flash SiO_2 gel column chromatography (PE: DCM = 8:1 to 1:2) afforded **13i** (33.1 mg, 62%) as a colorless syrup. TLC (PE:DCM, 3:5 V/V): R_f = 0.28; ^{1}H NMR (300 MHz, $CDCl_3$) δ 8.04 (br s, 1H), 8.00 (d, *J* = 1.5 Hz, 1H), 7.70 (d, *J* = 8.0 Hz, 1H), 7.58 – 7.49 (m, 1H), 7.45 (dd, *J* = 8.5, 2.0 Hz, 1H), 7.34 (d, *J* = 8.5 Hz, 1H), 6.05 – 5.92 (m, 2H), 5.36 (dt, *J* = 4.0, 1.5 Hz, 1H), 5.32 (dt, *J* = 4.0, 1.5 Hz, 1H), 5.29 – 5.22 (m, 2H), 4.62 (s, 2H), 4.10 (tt, *J* = 5.5, 1.5 Hz, 4H); ^{13}C NMR (75 MHz, $CDCl_3$) δ 144.5, 144.2, 136.4, 135.9, 134.6, 134.4, 130.2, 127.4, 126.8, 126.0, 124.0, 123.3, 121.6, 120.6, 117.9, 117.6, 71.7, 71.6, 71.5, 71.4; IR (film): 2926, 1390, 1263, 1090, 1075, 1030, 926, 746, 717 cm^{-1}; APCI-HRMS (m/z): $[M+H]^+$ calcd for $C_{20}H_{21}O_4S$: 357.1155; found: 357.1153.

Synthesis of 2,9-dimethoxydibenzo[*c*,*e*][1,2]oxathiine 6-oxide (13j) and 2,8-dimethoxy dibenzo[*b*,*d*]thiophene 5,5-dioxide (13j') from 10j

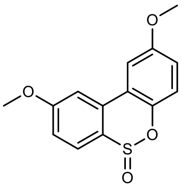

13j

2,9-Dimethoxydibenzo[*c*,*e*][1,2]oxathiine 6-oxide (**13j**) was synthesized according to **General procedure B: 10j** (36.6 mg, 0.15 mmol), *t*-BuOOH (160 μL, 6.0 equiv.) and 150 W Hg Lamp were employed. After stirring at 30 °C for 2.5 h, purification of the product by flash SiO_2 gel column chromatography (PE: EA = 8:1 to 2:1) afforded **13j** (8.3 mg, 20%) as colorless syrup, **13j'** (29.0 mg, 70%) as colorless solid. TLC (PE:EA, 2:1 V/V): R_f = 0.55; ^{1}H NMR (300 MHz, $CDCl_3$) δ 7.64 (d, *J* = 8.5 Hz, 1H), 7.42 (dd, *J* = 8.0, 2.5 Hz, 2H), 7.29 (d, *J* = 8.5 Hz, 1H), 7.10 – 6.97 (m, 2H), 3.95 (s, 3H), 3.89 (s, 3H); ^{13}C NMR (75 MHz, $CDCl_3$) δ 163.2, 157.1, 138.8, 130.3, 128.6, 127.7, 122.5, 121.4, 116.7, 113.8, 110.1, 109.5, 56.0, 55.9; APCI-HRMS (m/z): $[M+H]^+$ calcd for $C_{14}H_{13}O_4S$: 277.0529; found: 277.0528.

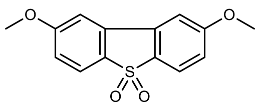

13j'

The spectroscopic data of 2,8-dimethoxydibenzo[*b*,*d*]thiophene 5,5-dioxide (**13j'**). mp: 96-98 °C; TLC (PE:EA, 2:1 V/V): R_f = 0.67; ^{1}H NMR (300 MHz, $CDCl_3$) δ 7.71 (d, *J* = 8.5 Hz, 2H), 7.57 (d, *J* = 2.5 Hz, 2H), 7.10 (dd, *J* = 8.5, 2.5 Hz, 2H), 3.96 (s, 6H); ^{13}C NMR (75 MHz, $CDCl_3$) δ 157.6, 136.6, 132.6, 123.7, 116.0, 105.0, 55.9; IR (film): 2965, 2826, 1599, 1556, 1479, 1456, 1425, 1329, 1302, 1263, 1226, 1205, 1167, 1141, 1028, 839, 810, 721, 700, 671 cm^{-1}; APCI-HRMS (m/z): $[M+H]^+$ calcd for $C_{14}H_{13}O_4S$: 277.0529; found: 277.0528.

Synthesis of 1-(6-oxidodibenzo[*c*,*e*][1,2]oxathiin-2-yl)ethan-1-one (13k) and 1-(6-oxidodi benzo[*c*,*e*] [1,2]oxathiin-9-yl)ethan-1-one (13k') from 10k

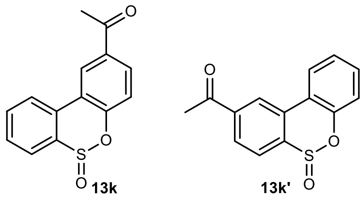

13k 13k'

We tried to synthesis of 1-(6-oxidodibenzo[*c*,*e*][1,2]oxathiin-2-yl)ethan-1-one (**13k**) and 1-(6-oxidodibenzo[*c*,*e*][1,2]oxathiin-9-yl)ethan-1-one (**13k'**) according to **General procedure B: 10k** (33.9 mg, 0.15 mmol) and *t*-BuOOH (160 μL, 6.0 equiv.) were employed. After stirring at 30 °C for 2.5 h, no desired product was isolated, only the starting material **10k** (31.5 mg, 93%) was recovered.

Synthesis of 1-(dibenzo[*b*,*d*]thiophen-2-yl)ethenone (10k) from 11k.

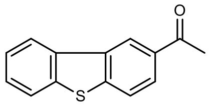

10k

1-(Dibenzo[*b*,*d*]thiophen-2-yl)ethenone (**10k**) was synthesized according to **General procedure C: 11k** (36.3 mg, 0.15 mmol) and *t*-BuOOH (160 μL, 6.0 equiv.) were employed. After stirring at 30 °C for 3.0 h, purification of the product by flash SiO_2 gel column chromatography (PE: EA = 6:1 to 2:1) afforded **10k** (30.2 mg, 89%) as a white solid. The spectroscopic properties of **10k** were consistent with the data available in the literature.[6]

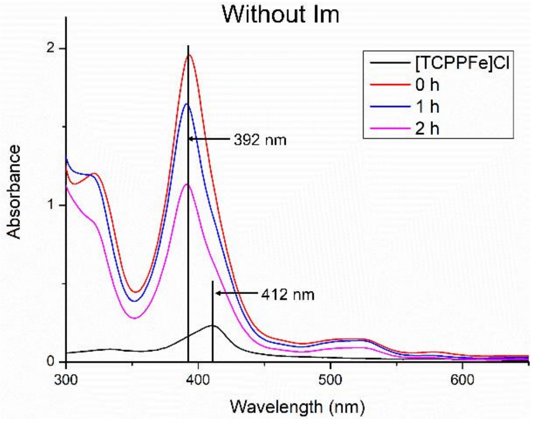

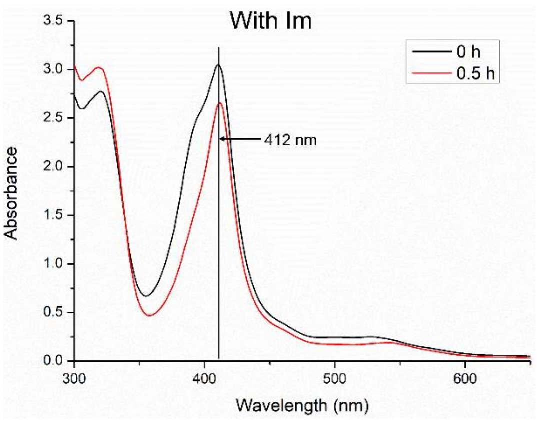

a. **b.**

Supplementary Figure 15. UV-Vis spectroscopy. a. UV-Vis spectrum of the reaction mixture using DBTO in the typical photocatalytic procedure; **b.** UV-Vis spectrum of the reaction mixture using DBTO with Im as extra ligand in the typical photocatalytic procedure.

Supplementary Table 1. Control experiments

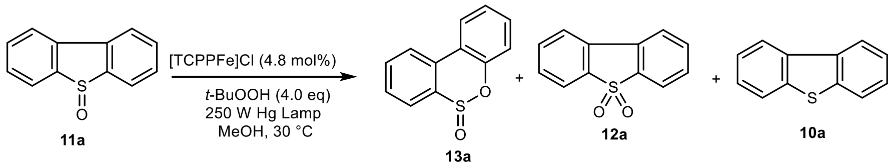

Entry	Variations from standard conditions	Conversion rate	Yield of **13a**[a]	Yield of **12a**[a]	Yield of **10a**[a]	Conclusions
1	none	97.3%	87.1% (84%)[b]	10.2%	0	_
2	Imidazole (1.0 eq) was added	69.9%	43.8%	8.9%	17.2%	_
3	no light[c,d]	trace	0	99% (89%)[b]	1%	_
4	no [TCPPFe]Cl, no *t*-BuOOH	93%	8.9%	4.8%	79.2%	_
5	no [TCPPFe]Cl	86%	16%	6.5%[c]	63.5%	_
6	no *t*-BuOOH	85.6%	15%	5.8%	4.8%	_
7	TEMPO (0.5 eq) was added	97.1%	48.1%	3.4%	45.6%	radical
8	TEMPO (1.0 eq) was added	78.7%	46%	2.9%	29.8%	radical
9	BHT (0.5 eq) was added	50.8%	40.3%	7.2%	3.2%	radical
10	BHT (1.0 eq) was added	69.1%	52.7%	13.3%	3.1%	radical
11	$CuCl_2$ (1.0 eq) was added	83.8%	58.8%	4.2%	20.8%	single electron
12	Benzophenone (1.0 eq) was added	95.8%	82.1%	10.5%	3.3%	triplet state

[a] Determined by HPLC analysis of the crude reaction mixture. [b] Isolated yield in bracket. [c] PhIO was used as oxidant. [d] When the reaction was performed under the standard reaction conditions using *t*-BuOOH as oxidant, after 24 h, the conversion rate of DBTO was 14%, and $DBTO_2$ was detected as only product in 14% HPLC yield.

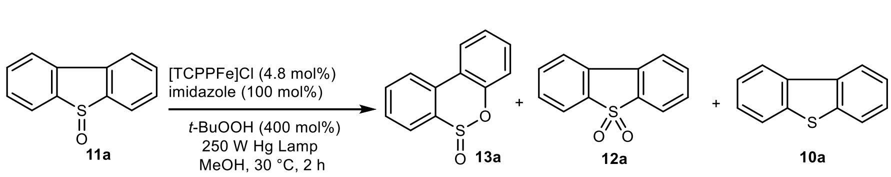

Supplementary Figure 16. Control experiment with imidazole. Supplementary Table 1, entry 2, according to **General procedure C: 11a** (30.1 mg, 0.15 mmol), *t*-BuOOH (110 μL, 4.0 equiv.) and imidazole (10.2 mg, 0.15 mmol) were employed. The reaction was monitored by HPLC, after stirring at 30 °C for 2 h, afforded **13a** (43.8%), **12a** (8.9%) and **10a** (17.2%). (For HPLC traces, see Supplementary Figures 17-21).

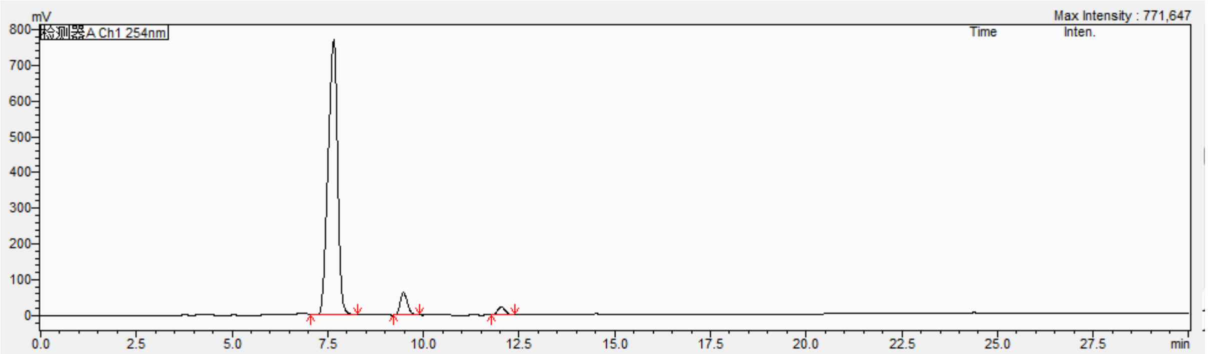

Peak No.	Retention time	Area	Area (%)	Height	Height (%)	S/N
1	7.656	13216516	92.040	769165	90.032	2737.50
2	9.479	828451	5.769	63068	7.382	224.46
3	12.034	314492	2.190	22092	2.586	78.63
Total		14359459	100.00	854325	100.00	

Supplementary Figure 17. The reaction progress with imidazole. HPLC trace at 5 min. Retention time: **11a** 7.7 min (92.0%) (Conversion rate 8.0%); **12a** 9.5 min (5.8% yield); **13a** 12.0 min (2.2% yield).

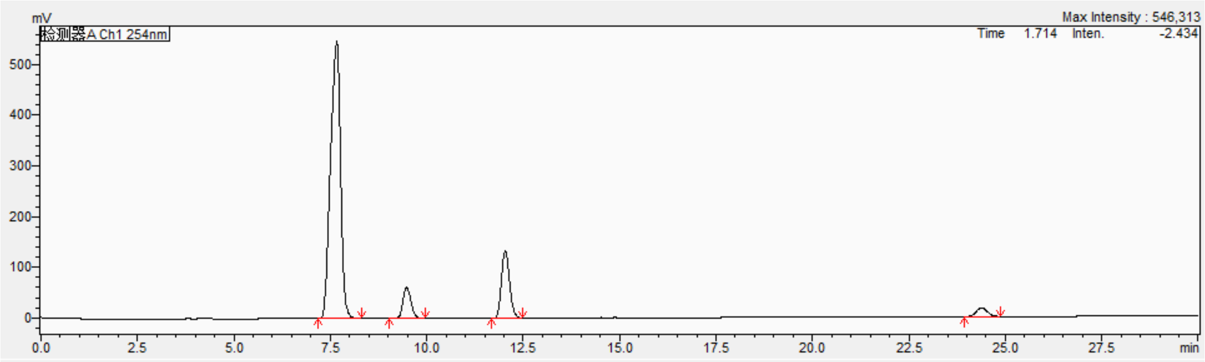

Peak No.	Retention time	Area	Area (%)	Height	Height (%)	S/N
1	7.665	9872278	75.604	547837	72.093	2152.64
2	9.478	875451	6.704	60911	8.016	239.34
3	12.035	1949707	14.931	133404	17.556	524.19
4	24.384	360369	2.760	17747	2.335	69.73
Total		13057806	100.00	759899	100.00	

Supplementary Figure 18. The reaction progress with imidazole. HPLC trace at 0.5 h. Retention time: **11a** 7.7 min (75.6%) (Conversion rate 24.4%); **12a** 9.5 min (6.7% yield); **13a** 12.0 min (14.9% yield); **10a** 24.4 min (2.8% yield).

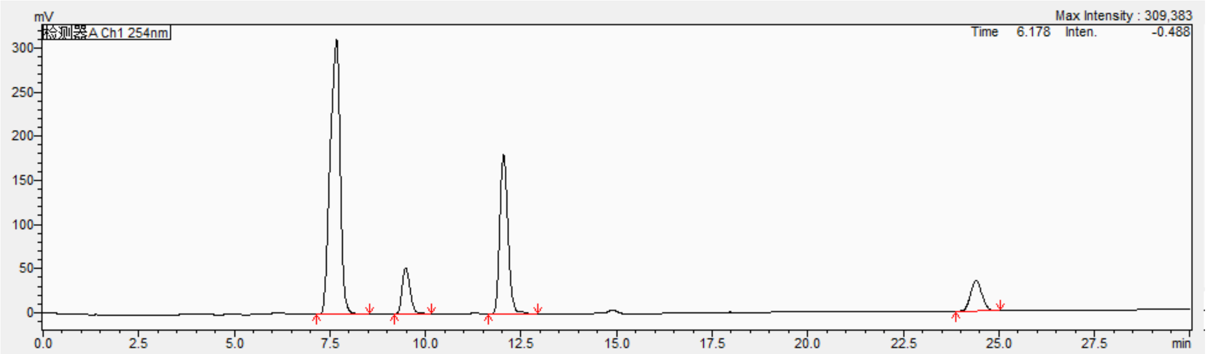

Peak No.	Retention time	Area	Area (%)	Height	Height (%)	S/N
1	7.672	5677109	57.531	311183	53.753	1049.80
2	9.484	753409	7.635	52086	8.997	175.71
3	12.043	2721554	27.580	180782	31.228	609.88
4	24.402	715861	7.254	34864	6.022	117.61
Total		9867933	100.00	578914	100.00	

Supplementary Figure 19. The reaction progress with imidazole. HPLC trace at 1.0 h. Retention time: **11a** 7.7 min (57.5%) (Conversion rate 42.5%); **12a** 9.5 min (7.6% yield); **13a** 12.0 min (27.6% yield); **10a** 24.4 min (7.3% yield).

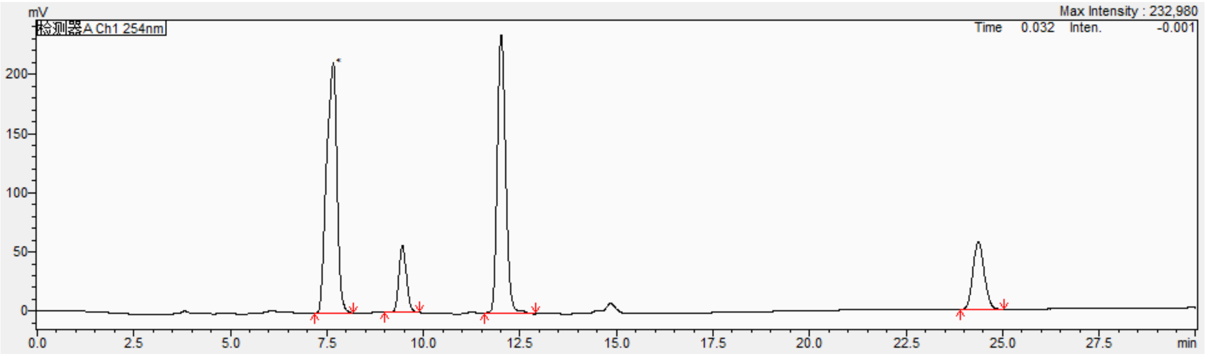

Peak No.	Retention time	Area	Area (%)	Height	Height (%)	S/N
1	7.664	3955437	41.971	211856	37.848	515.61
2	9.457	763399	8.100	56013	10.007	136.32
3	12.013	3529938	37.456	234609	41.913	570.98
4	24.373	1175551	12.474	57276	10.232	139.40
Total		9424324	100.00	559754	100.00	

Supplementary Figure 20. The reaction progress with imidazole. HPLC trace at 1.5 h. Retention time: **11a** 7.7 min (42.0%) (Conversion rate 58.0%); **12a** 9.5 min (8.1% yield); **13a** 12.0 min (37.5% yield); **10a** 24.4 min (12.5% yield).

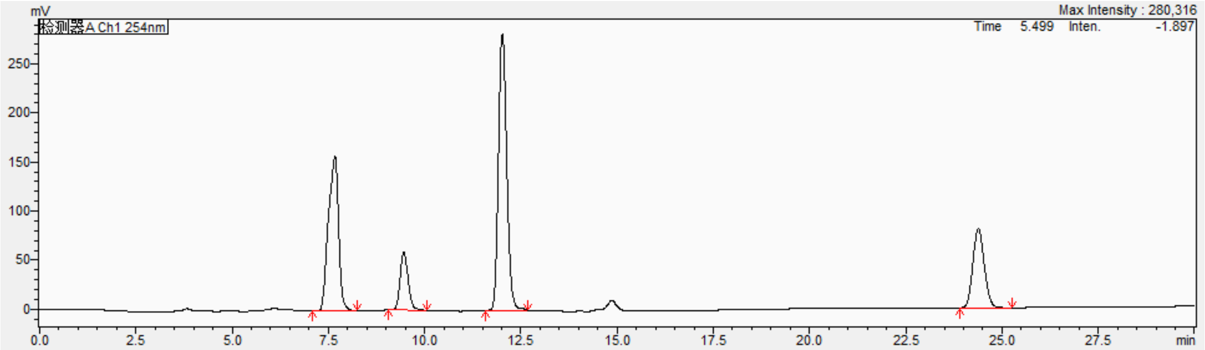

Peak No.	Retention time	Area	Area (%)	Height	Height (%)	S/N
1	7.669	2902871	30.066	157829	27.233	329.20
2	9.459	858639	8.893	59160	10.208	123.39
3	12.018	4229332	43.805	281719	48.610	587.61
4	24.386	1664115	17.236	80848	13.950	168.63
Total		9654957	100.00	579556	100.00	

Supplementary Figure 21. The reaction progress with imidazole. HPLC trace at 2.0 h. Retention time: **11a** 7.7 min (30.1%) (Conversion rate 69.9%); **12a** 9.5 min (8.9% yield); **13a** 12.0 min (43.8% yield); **10a** 24.4 min (17.2% yield).

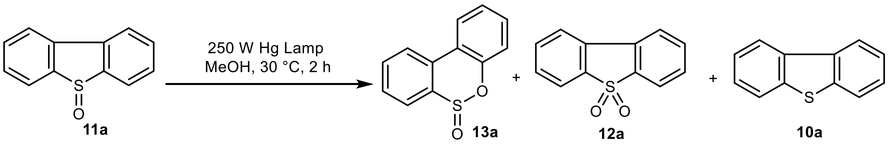

Supplementary Figure 22. Control experiment without of [TCPPFe]Cl and *t*-BuOOH. Supplementary Table 1, entry 4, according to **General procedure C: 11a** (30.1 mg, 0.15 mmol) was employed. The reaction was monitored by HPLC, after stirring at 30 ˚C for 2 h, afforded **13a** (8.9%), **12a** (4.8%) and **10a** (79.2%). (For HPLC traces, see Supplementary Figures 23-26).

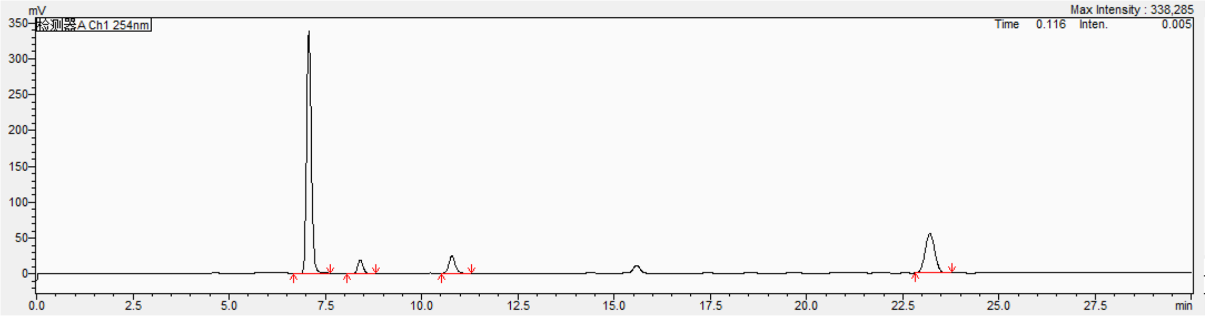

Peak No.	Retention time	Area	Area (%)	Height	Height (%)	S/N
1	7.072	2900909	67.110	337290	77.377	519.24
2	8.405	178661	4.133	19121	4.386	29.44
3	10.786	286980	6.639	24448	5.608	37.64
4	23.204	956059	22.118	55049	12.629	87.74
Total		4322609	100.00	435908	100.00	

Supplementary Figure 23. The reaction progress without [TCPPFe]Cl and *t*-BOOH. HPLC trace at 0.5 h. Retention time: **11a** 7.1 min (67.1%) (Conversion rate 32.9%); **12a** 8.4 min (4.1% yield); **13a** 10.8 min (6.6% yield); **10a** 23.2 min (22.1% yield).

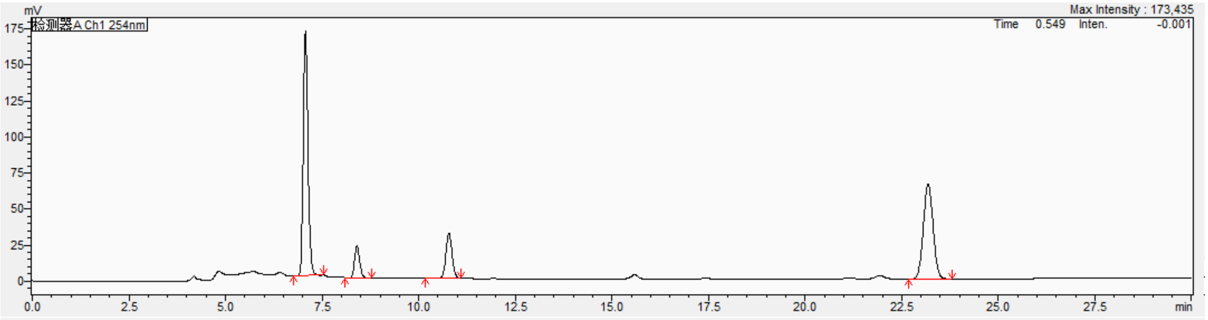

Peak No.	Retention time	Area	Area (%)	Height	Height (%)	S/N
1	7.070	1444827	45.878	169494	58.666	355.75
2	8.405	202357	6.425	22085	7.644	46.35
3	10.781	353964	11.239	31495	10.901	66.10
4	23.186	1148143	36.457	65838	22.788	138.19
Total		3149291	100.00	288911	100.00	

Supplementary Figure 24. The reaction progress without [TCPPFe]Cl and *t*-BOOH. HPLC trace at 1.0 h. Retention time: **11a** 7.1 min (45.9%) (Conversion rate 54.1%); **12a** 8.4 min (6.4% yield); **13a** 10.8 min (11.2% yield); **10a** 23.2 min (36.5% yield).

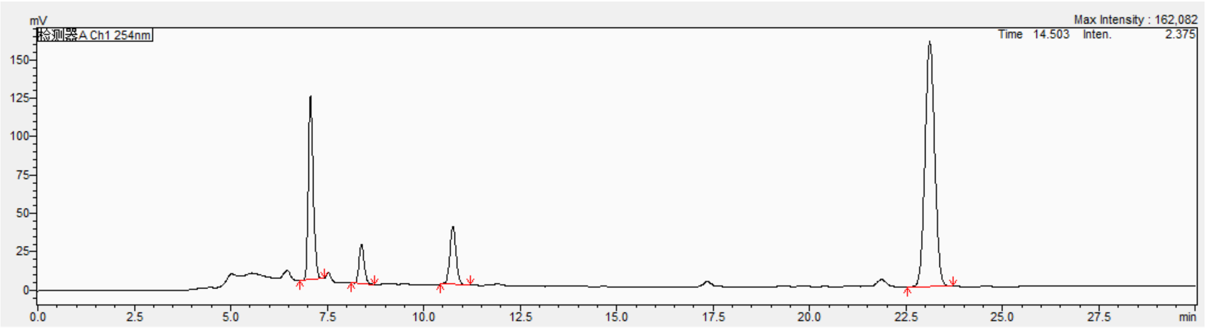

Peak No.	Retention time	Area	Area (%)	Height	Height (%)	S/N
1	7.068	1059619	23.462	119653	34.900	199.22
2	8.388	235303	5.210	25503	7.439	42.46
3	10.756	429199	9.503	38014	11.088	63.29
4	23.116	2792237	61.825	159674	46.573	265.86
Total		4516358	100.00	342844	100.00	

Supplementary Figure 25. The reaction progress without [TCPPFe]Cl and *t*-BOOH. HPLC trace at 1.5 h. Retention time: **11a** 7.1 min (23.5%) (Conversion rate 76.5%); **12a** 8.4 min (5.2% yield); **13a** 10.8 min (9.5% yield); **10a** 23.1 min (61.8% yield).

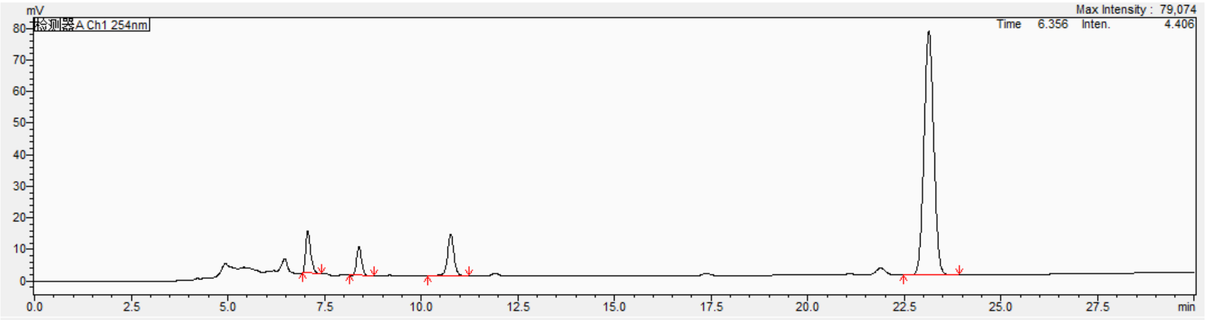

Peak No.	Retention time	Area	Area (%)	Height	Height (%)	S/N
1	7.070	119843	7.064	13425	11.922	52.14
2	8.395	81149	4.771	8987	7.981	34.90
3	10.764	152185	8.948	13093	11.627	50.85
4	23.134	1347622	79.235	77102	68.470	299.42
Total		1700800	100.00	112607	100.00	

Supplementary Figure 26. The reaction progress without [TCPPFe]Cl and *t*-BOOH. HPLC trace at 2.0 h. Retention time: **11a** 7.1 min (7.0%) (Conversion rate 93.0%); **12a** 8.4 min (4.8% yield); **13a** 10.8 min (8.9% yield); **10a** 23.2 min (79.2% yield).

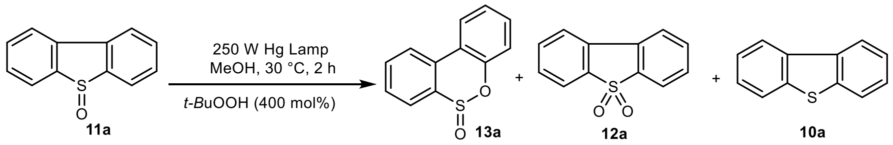

Supplementary Figure 27. Control experiment in the absence of [TCPPFe]Cl. Supplementary Table 1, entry 5, according to **General procedure C: 11a** (30.1 mg, 0.15 mmol), *t*-BuOOH (110 μL, 4.0 equiv.) were employed. The reaction was monitored by HPLC, after stirring at 30 °C for 2 h, afforded **13a** (16%), **12a** (6.5%) and **10a** (63.5%). (For HPLC traces, see Supplementary Figures 28-31).

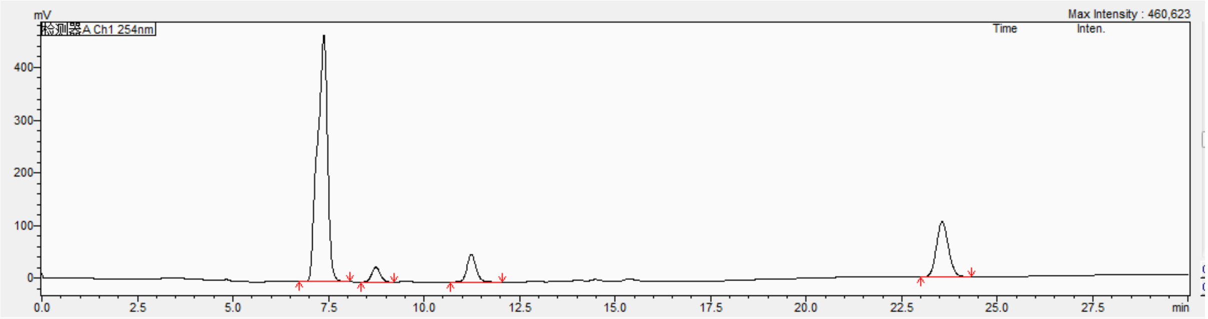

Peak No.	Retention time	Area	Area (%)	Height	Height (%)	S/N
1	7.380	8598053	70.731	466935	71.676	683.26
2	8.740	433241	3.564	28183	4.326	41.24
3	10.235	863804	7.106	52322	8.032	76.56
4	23.559	2260942	18.599	104017	15.967	152.21
Total		12156040	100.00	651456	100.00	

Supplementary Figure 28. The reaction progress in the absence of [TCPPFe]Cl. HPLC trace at 0.5 h. Retention time: **11a** 7.4 min (70.7%) (Conversion rate 29.3%); **12a** 8.7 min (3.6% yield); **13a** 11.2 min (7.1% yield); **10a** 23.6 min (18.6% yield).

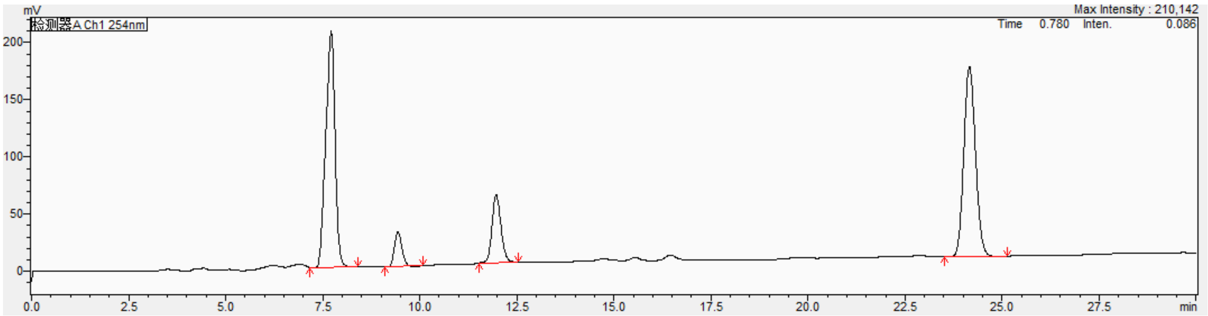

Peak No.	Retention time	Area	Area (%)	Height	Height (%)	S/N
1	7.717	3428860	41.677	206592	44.754	325.74
2	9.436	404055	4.911	30255	6.554	47.70
3	11.968	948045	11.523	58953	12.771	92.95
4	24.159	3446264	41.888	165813	35.920	261.44
Total		8227202	100.00	461613	100.00	

Supplementary Figure 29. The reaction progress in the absence of [TCPPFe]Cl. HPLC trace at 1.0 h. Retention time: **11a** 7.7 min (41.6%) (Conversion rate 58.4%); **12a** 9.4 min (4.9% yield); **13a** 12.0 min (11.5% yield); **10a** 24.2 min (41.9% yield).

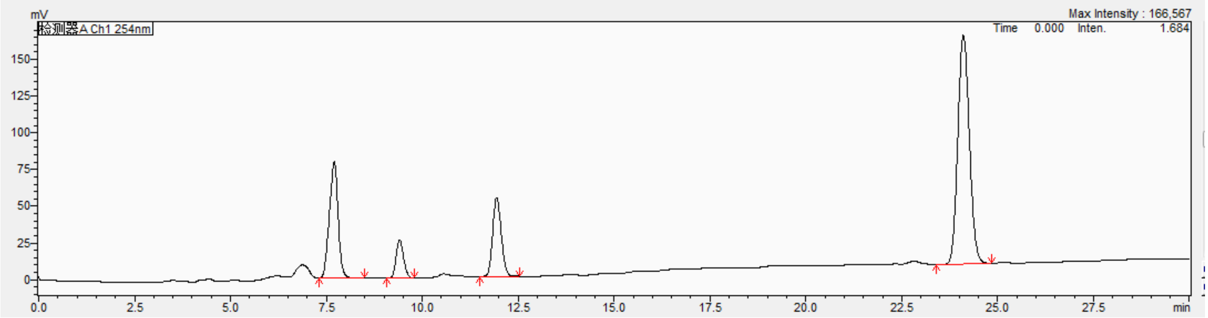

Peak No.	Retention time	Area	Area (%)	Height	Height (%)	S/N
1	7.706	1258378	22.088	79256	25.173	170.00
2	9.409	353085	6.198	26102	8.290	55.99
3	11.939	834455	14.647	53770	17.078	115.33
4	24.108	3251146	57.067	155720	49.459	334.01
Total		5697064	100.00	314848	100.00	

Supplementary Figure 30. The reaction progress in the absence of [TCPPFe]Cl. HPLC trace at 1.5 h. Retention time: **11a** 7.7 min (22.1%) (Conversion rate 77.9%); **12a** 9.4 min (6.2% yield); **13a** 11.9 min (14.6% yield); **10a** 24.1 min (57.1% yield).

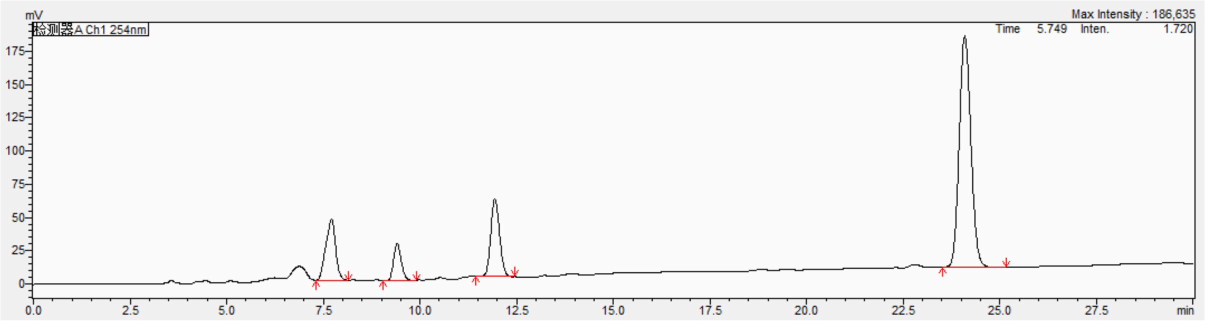

Peak No.	Retention time	Area	Area (%)	Height	Height (%)	S/N
1	7.713	800462	13.956	46186	15.087	90.33
2	9.408	372320	6.491	27639	9.029	54.06
3	11.936	918349	16.011	58224	19.020	113.88
4	24.092	3644443	63.541	174073	56.864	340.47
Total		5735574	100.00	306121	100.00	

Supplementary Figure 31. The reaction progress in the absence of [TCPPFe]Cl. HPLC trace at 2.0 h. Retention time: **11a** 7.7 min (14.0%) (Conversion rate 86.0%); **12a**9.4 min (6.5% yield); **13a** 11.9 min (16.0% yield); **10a** 24.1 min (63.5% yield).

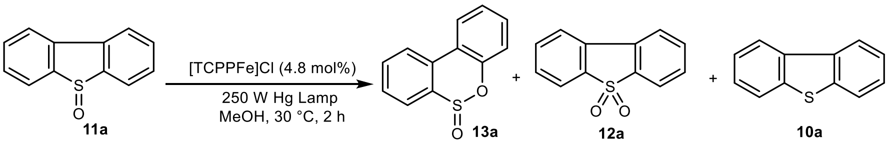

Supplementary Figure 32. Control experiment in the absence of *t*-BuOOH. Supplementary Table 1, entry 6, according to **General procedure C: 11a** (30.1 mg, 0.15 mmol), and [TCPPFe]Cl (6.0 mg, 4.8 mol%) were employed. The reaction was monitored by HPLC, after stirring at 30 °C for 2 h, afforded **13a** (15.1%), **12a** (5.8%) and **10a** (64.8%). (For HPLC traces, see Supplementary Figures 33-36).

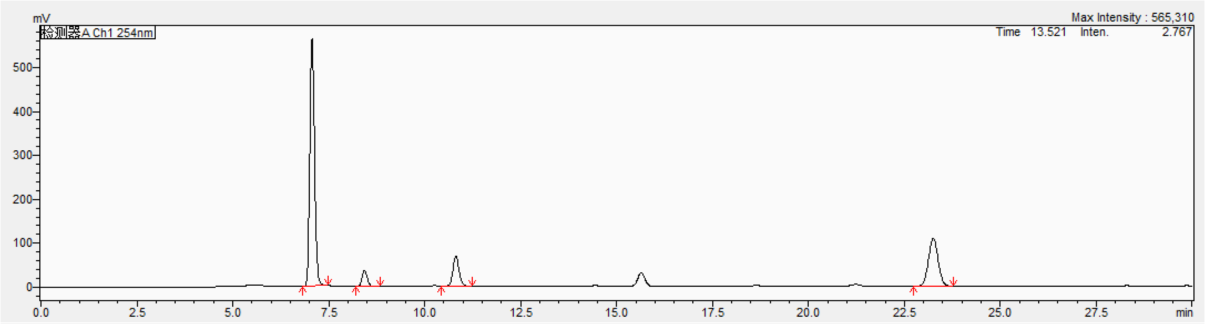

Peak No.	Retention time	Area	Area (%)	Height	Height (%)	S/N
1	7.066	4900712	62.043	563113	72.571	538.15
2	8.431	339557	4.299	36673	4.726	35.05
3	10.813	778921	9.861	68343	8.808	65.31
4	23.254	1879668	23.797	107817	13.895	103.04
Total		7898858	100.00	775946	100.00	

Supplementary Figure 33. The reaction progress in the absence of *t*-BuOOH. HPLC trace at 0.5 h. Retention time: **11a** 7.1 min (62.0%) (Conversion rate 38.0%); **12a** 8.4 min (4.3% yield); **13a** 10.8 min (9.9% yield); **10a** 23.3 min (23.8% yield).

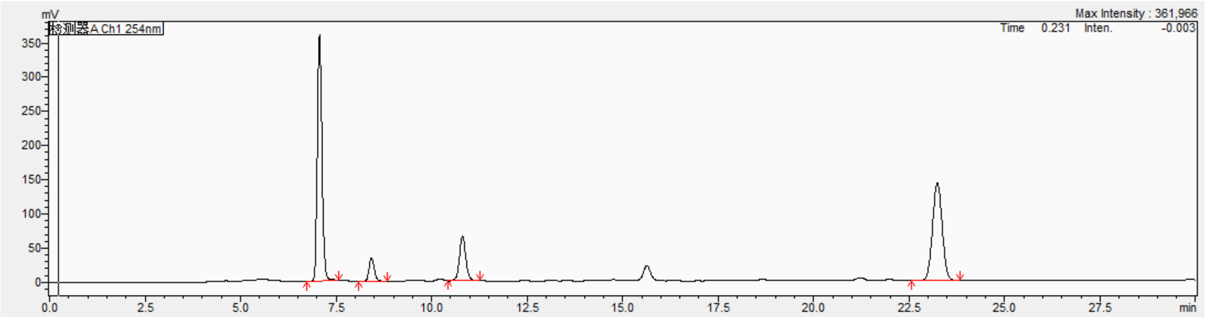

Peak No.	Retention time	Area	Area (%)	Height	Height (%)	S/N
1	7.067	3063128	46.152	359665	59.739	375.93
2	8.430	313844	4.729	33731	5.603	35.26
3	10.809	771294	11.621	65976	10.958	68.96
4	23.241	2488808	37.499	142690	23.700	149.14
Total		6637074	100.00	602061	100.00	

Supplementary Figure 34. The reaction progress in the absence of *t*-BuOOH. HPLC trace at 1.0 h. Retention time: **11a** 7.1 min (46.2%) (Conversion rate 53.8%); **12a** 8.4 min (4.7% yield); **13a** 10.8 min (11.6% yield); **10a** 23.2 min (37.5% yield).

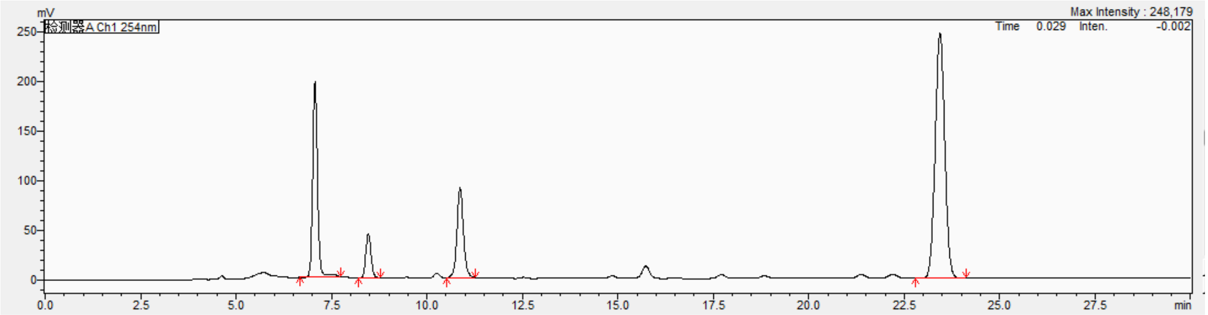

Peak No.	Retention time	Area	Area (%)	Height	Height (%)	S/N
1	7.068	1755130	23.223	196997	34.052	192.23
2	8.464	410021	5.427	44021	7.609	42.96
3	10.868	1079176	14.285	90891	15.711	88.69
4	23.437	4310290	57.055	246611	42.628	240.65
Total		7554617	100.00	578519	100.00	

Supplementary Figure 35. The reaction progress in the absence of *t*-BuOOH. HPLC trace at 1.5 h. Retention time: **11a** 7.1 min (23.2%) (Conversion rate 76.8%); **12a** 8.5 min (5.4% yield); **13a** 10.9 min (14.3% yield); **10a** 23.4 min (57.1% yield).

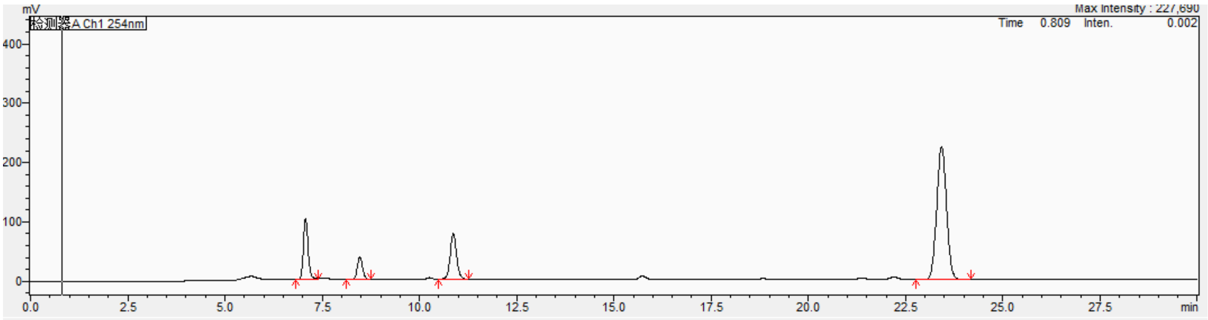

Peak No.	Retention time	Area	Area (%)	Height	Height (%)	S/N
1	7.075	875372	14.412	102507	23.153	153.45
2	8.467	349700	5.757	37589	8.490	56.27
3	10.871	915344	15.070	77742	17.560	116.38
4	23.426	3933431	64.760	224893	50.797	336.65
Total		6073846	100.00	442732	100.00	

Supplementary Figure 36. The reaction progress in the absence of without *t*-BuOOH. HPLC trace at 2.0 h. Retention time: **11a** 7.1 min (14.4%) (Conversion rate 85.6%); **12a** 8.5 min (5.8% yield); **13a** 10.9 min (15.1% yield); **10a** 23.4 min (64.8% yield).

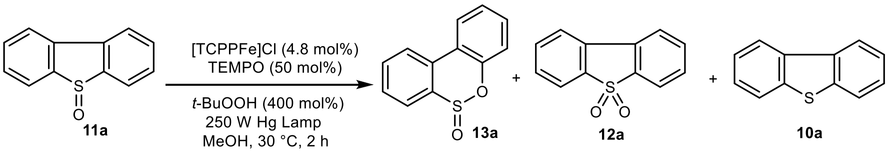

Supplementary Figure 37. Control experiment in the presence of 50 mol% TEMPO. Supplementary Table 1, entry 7, according to **General procedure C: 11a** (30.1 mg, 0.15 mmol), *t*-BuOOH (110 μL, 4.0 equiv.) and TEMPO (11.7 mg, 0.075 mmol) were employed. The reaction was monitored by HPLC, after stirring at 30 ˚C for 2 h, afforded **13a** (15.1%), **12a** (5.8%) and **10a** (64.8%). (For HPLC traces, see Supplementary Figures 38-42).

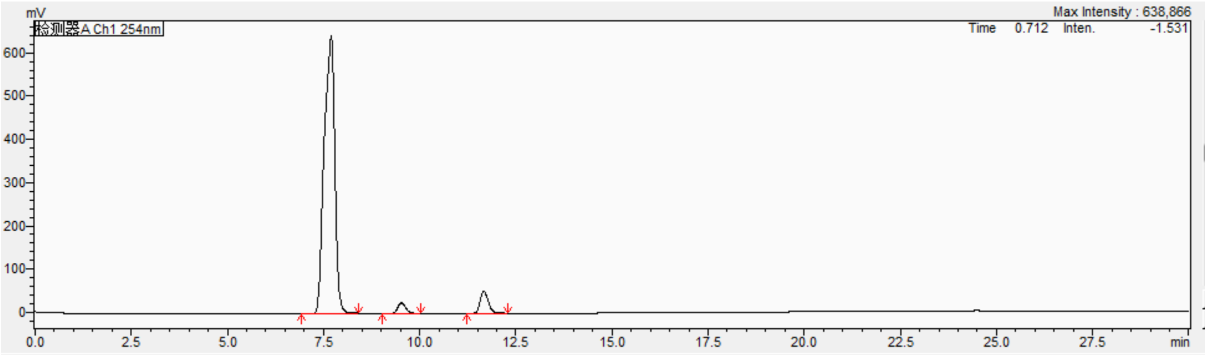

Peak No.	Retention time	Area	Area (%)	Height	Height (%)	S/N
1	7.695	12260706	91.893	640796	89.472	4359.12
2	9.522	333042	2.496	24059	3.359	163.66
3	11.688	748635	5.611	51339	7.168	349.24
Total		13342383	100.00	716194	100.00	

Supplementary Figure 38. The reaction progress with 50 mol% TEMPO. HPLC trace at 5 min. Retention time: **11a** 7.7 min (91.9%) (Conversion rate 8.1%); **12a** 9.5 min (2.5% yield); **13a** 11.7 min (5.6% yield).

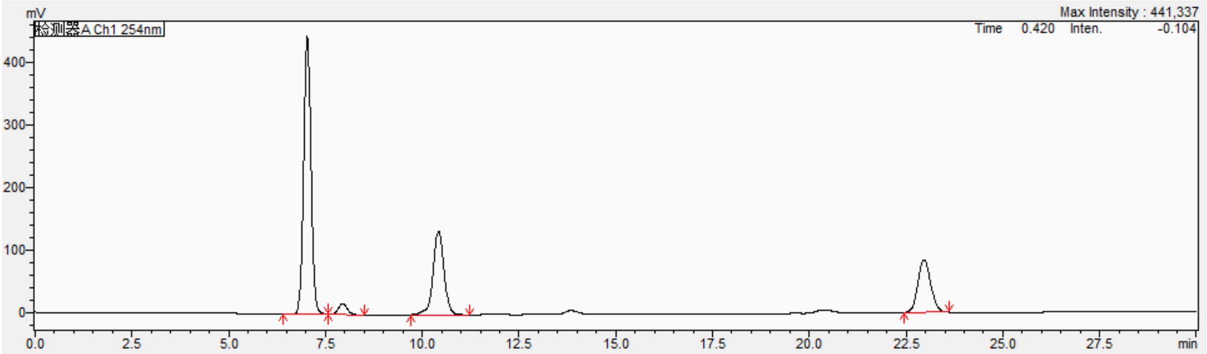

Peak No.	Retention time	Area	Area (%)	Height	Height (%)	S/N
1	7.031	5861834	54.823	443982	65.413	1045.34
2	7.977	271921	2.543	17225	2.538	40.56
3	10.422	2631986	24.616	133715	19.701	314.83
4	22.963	1926642	18.019	83816	12.349	197.34
Total		10692383	100.00	678739	100.00	

Supplementary Figure 39. The reaction progress with 50 mol% TEMPO. HPLC trace at 0.5 h. Retention time: **11a** 7.0 min (54.8%) (Conversion rate 45.2%); **12a** 7.9 min (2.5% yield); **13a** 10.4 min (24.6% yield); **10a** 23.0 min (18.0% yield).

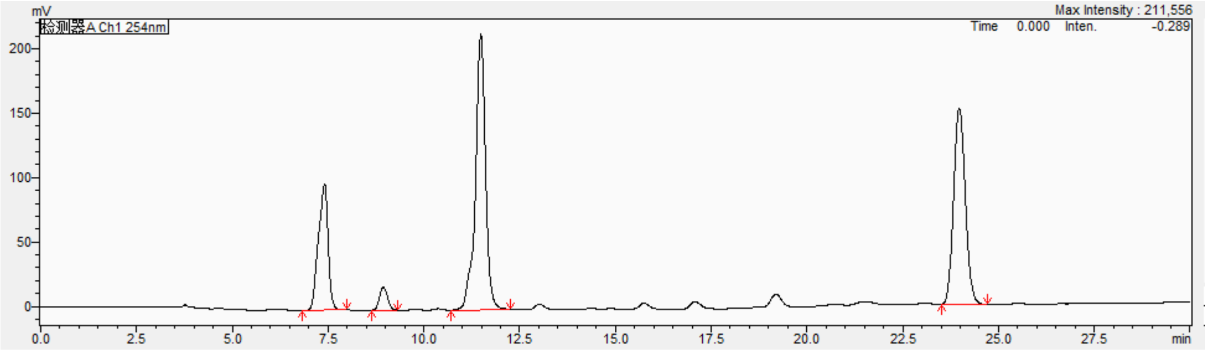

Peak No.	Retention time	Area	Area (%)	Height	Height (%)	S/N
1	7.408	1724066	18.852	98221	20.341	123.78
2	8.941	268865	2.940	18019	3.732	22.71
3	11.484	3912385	42.781	214205	44.361	269.95
4	23.974	3239911	35.427	152424	31.566	192.09
Total		9145227	100.00	482869	100.00	

Supplementary Figure 40. The reaction progress with 50 mol% TEMPO. HPLC trace at 1.0 h. Retention time: **11a** 7.4 min (18.9%) (Conversion rate 81.1%); **12a** 8.9 min (2.9% yield); **13a** 11.5 min (42.8% yield); **10a** 24.0 min (35.4% yield).

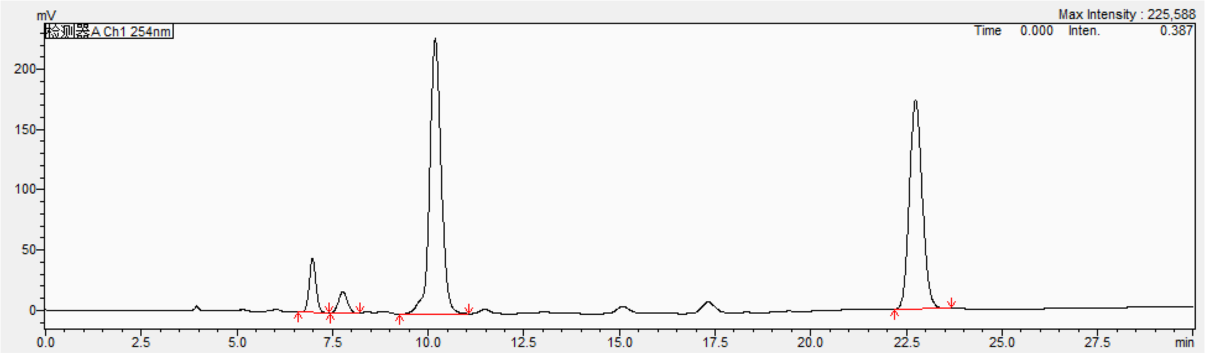

Peak No.	Retention time	Area	Area (%)	Height	Height (%)	S/N
1	6.979	558248	5.769	45103	9.698	68.00
2	7.768	288406	2.980	17825	3.833	26.87
3	10.183	4765789	49.251	288591	49.150	344.63
4	22.740	4064086	41.999	173574	37.320	261.68
Total		9676530	100.00	465093	100.00	

Supplementary Figure 41. The reaction progress with 50 mol% TEMPO. HPLC trace at 1.5 h. Retention time: **11a** 7.0 min (5.8%) (Conversion rate 94.2%); **12a** 7.8 min (3.0% yield); **13a** 10.2 min (49.3% yield); **10a** 22.7 min (42.0% yield).

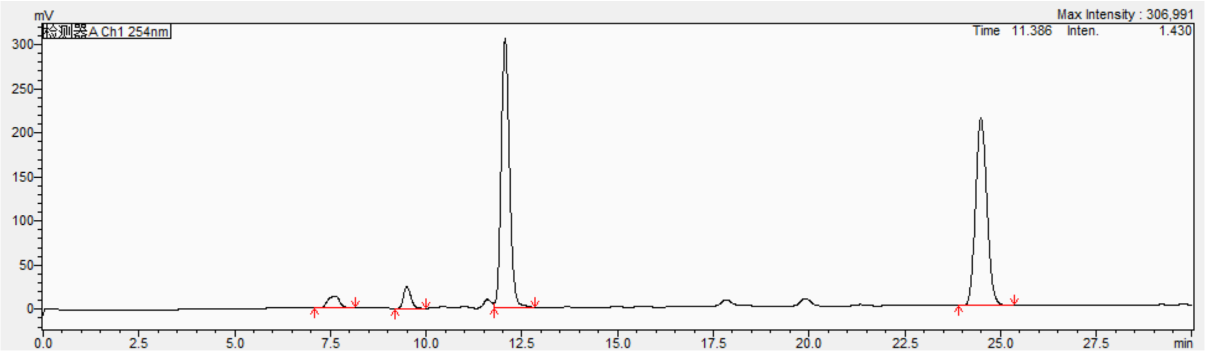

Peak No.	Retention time	Area	Area (%)	Height	Height (%)	S/N
1	7.628	265575	2.780	13017	2.347	15.79
2	9.496	328743	3.441	24481	4.413	29.69
3	12.060	4598936	48.143	305358	55.047	370.30
4	24.488	4359467	45.636	211870	38.194	256.93
Total		9552721	100.00	554725	100.00	

Supplementary Figure 42. The reaction progress with 50 mol% TEMPO. HPLC trace at 2.0 h. Retention time: **11a** 7.6 min (2.8%) (Conversion rate 97.2%); **12a** 9.5 min (3.4% yield); **13a** 12.0 min (48.1% yield); **10a** 24.5 min (45.6% yield).

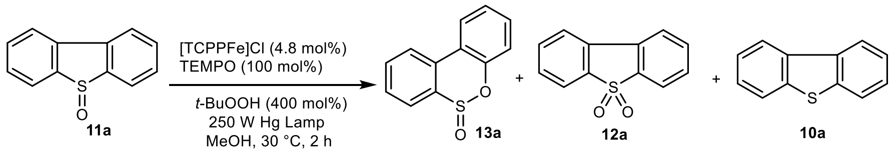

Supplementary Figure 43. Control experiment in the presence of 100 mol% TEMPO. Supplementary Table 1, entry 8, according to **General procedure C: 11a** (30.1 mg, 0.15 mmol), *t*-BuOOH (110 μL, 4.0 equiv.) and TEMPO (23.4 mg, 0.15 mmol) were employed. The reaction was monitored by HPLC, after stirring at 30 °C for 2 h, afforded **13a** (46.0%), **12a** (2.9%) and **10a** (29.8%). Please note, an unknown compound was formed during the reaction. The retention time of this compound is 6.9 min in the beginning of reaction, and changed to 11.7 min after 30 min's reaction. The yield of this compound is about 11%, which was excluded during the calculation of HPLC yield of DBTO, BPS and $DBTO_2$. (For HPLC traces, see Supplementary Figures 44-48).

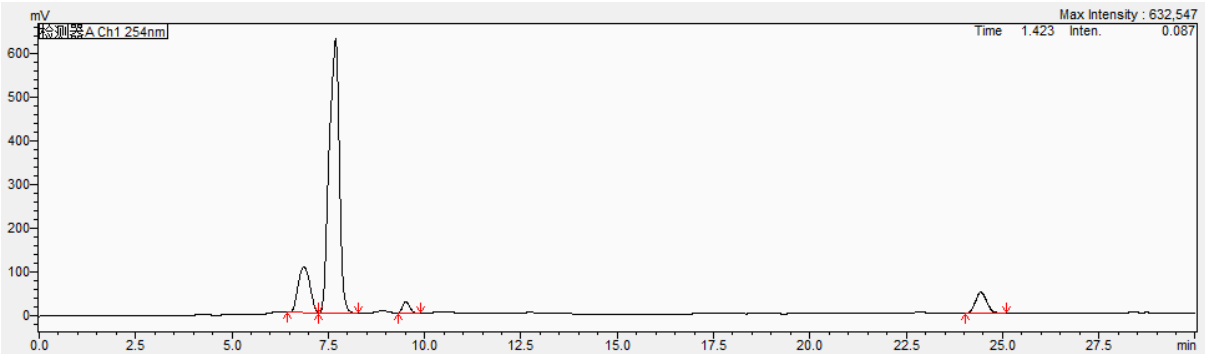

Peak No.	Retention time	Area	Area (%)	Height	Height (%)	S/N
1	6.865	2224975	14.824	104713	12.996	182.36
2	7.691	11488261	76.542	627239	77.845	1092.38
3	9.512	329010	2.192	25808	3.203	44.95
4	24.435	966929	6.442	47998	5.597	83.59
Total		15009175	100.00	805759	100.00	

Supplementary Figure 44. The reaction progress with 100 mol% TEMPO. HPLC trace at 5 min. Retention time: **11a** 7.7 min (89.9%) (Conversion rate 10.1%); **12a** 9.5 min (2.6% yield); **10a** 24.4 min (7.5% yield).

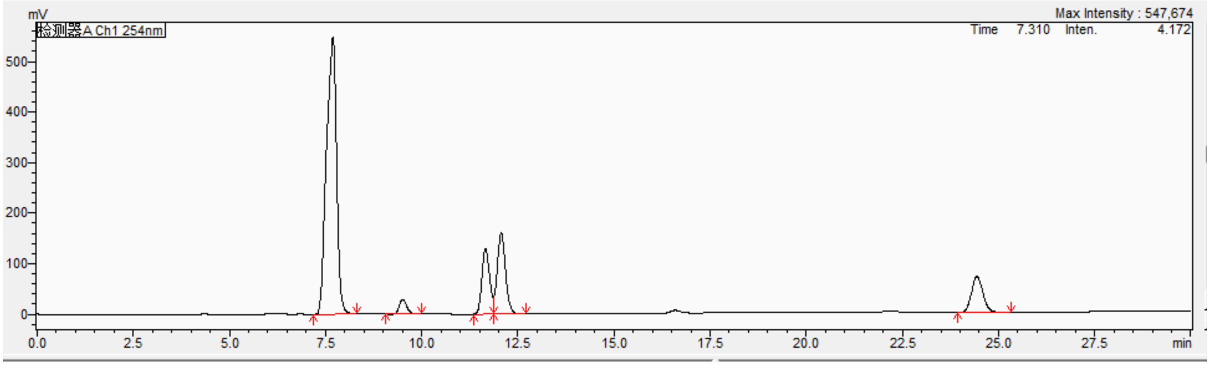

Peak No.	Retention time	Area	Area (%)	Height	Height (%)	S/N
1	7.689	10014551	62.237	547345	58.490	1692.16
2	9.505	387524	2.408	28821	3.080	89.10
3	11.660	1828300	11.362	128776	13.761	398.12
4	12.066	2412554	14.993	160287	17.128	495.54
5	24.439	1448156	9.000	70564	7.541	218.16
Total		16091086	100.00	482869	100.00	

Supplementary Figure 45. The reaction progress with 100 mol% TEMPO. HPLC trace at 0.5 h. Retention time: **11a** 7.7 min (70.2%) (Conversion rate 29.8%); **12a** 9.5 min (2.7% yield); **13a** 12.1 min (16.9% yield); **10a** 24.4 min (10.1% yield).

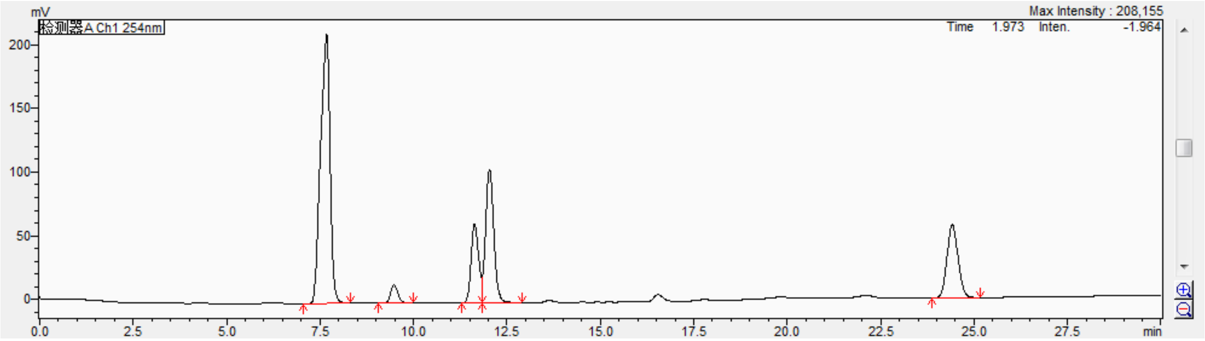

Peak No.	Retention time	Area	Area (%)	Height	Height (%)	S/N
1	7.673	3721026	49.136	211208	46.910	512.00
2	9.478	193281	2.552	14343	3.186	34.77
3	11.637	880552	11.628	62283	13.833	150.98
4	12.034	1601962	21.154	105033	23.328	254.62
5	24.418	1176034	15.530	57374	12.743	139.09
Total		7572855	100.00	450241	100.00	

Supplementary Figure 46. The reaction progress with 100 mol% TEMPO. HPLC trace at 1.0 h. Retention time: **11a** 7.7 min (55.6%) (Conversion rate 44.4%); **12a** 9.5 min (2.9% yield); **13a** 12.0 min (23.9% yield); **10a** 24.4 min (17.6% yield).

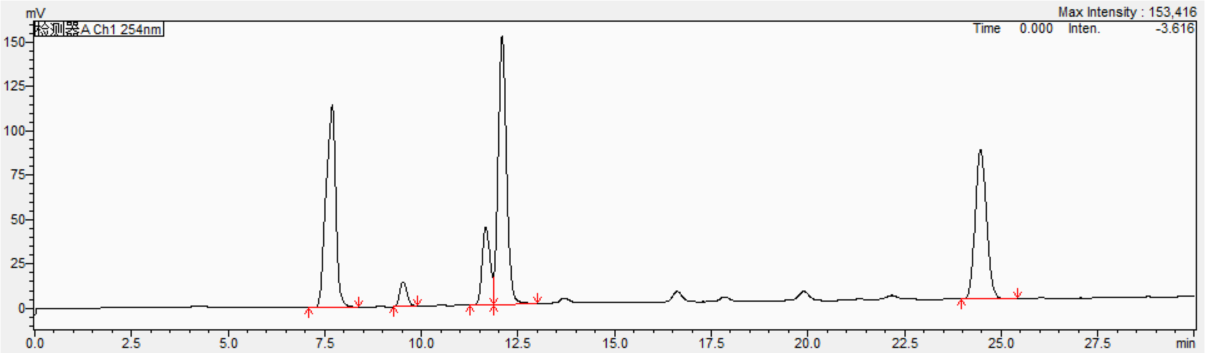

Peak No.	Retention time	Area	Area (%)	Height	Height (%)	S/N
1	7.692	1994377	29.263	114042	27.994	189.21
2	9.525	182352	2.676	13919	3.417	23.09
3	11.669	629364	9.235	43876	10.770	72.80
4	12.088	2290126	33.603	151200	37.115	250.86
5	24.468	1719096	25.224	84346	20.704	139.94
Total		6815315	100.00	407384	100.00	

Supplementary Figure 47. The reaction progress with 100 mol% TEMPO. HPLC trace at 1.5 h. Retention time: **11a** 7.7 min (32.2%) (Conversion rate 67.8%); **12a** 9.5 min (2.9% yield); **13a** 12.1 min (37.0% yield); **10a** 24.4 min (28.9% yield).

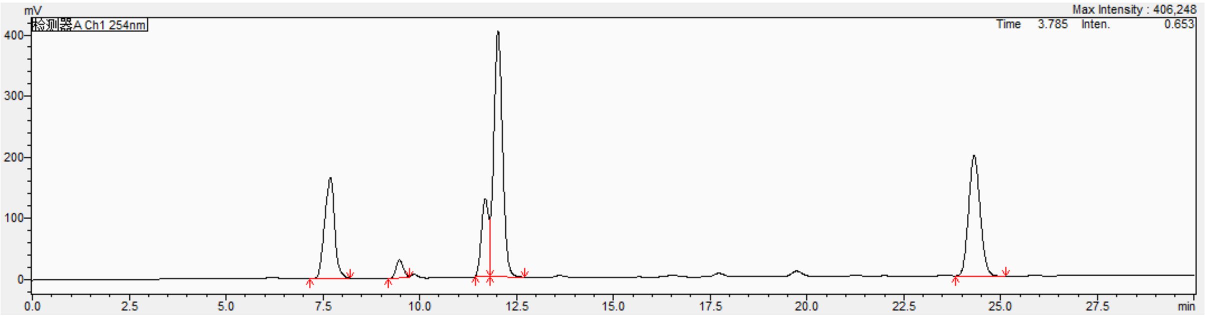

Peak No.	Retention time	Area	Area (%)	Height	Height (%)	S/N
1	7.698	2927008	18.963	165125	17.909	212.91
2	9.477	397254	2.574	29880	3.241	38.53
3	11.694	1663811	10.779	127164	13.792	163.96
4	12.022	6338674	41.067	401878	43.586	518.18
5	24.315	4108216	26.616	197993	21.473	255.29
Total		15434963	100.00	922040	100.00	

Supplementary Figure 48. The reaction progress with 100 mol% TEMPO. HPLC trace at 2.0 h. Retention time: **11a** 7.7 min (21.3%) (Conversion rate 78.7%); **12a** 9.5 min (2.9% yield); **13a** 12.0 min (46.0% yield); **10a** 24.3 min (29.8% yield).

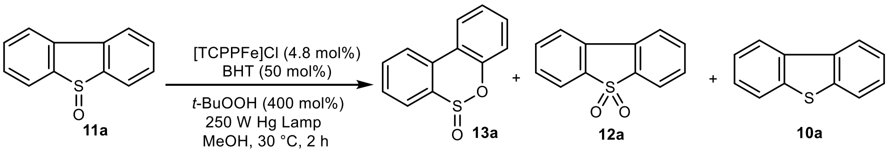

Supplementary Figure 49. Control experiment in the presence of 50 mol% BHT. Supplementary Table 1, entry 9, according to **General procedure C: 11a** (30.1 mg, 0.15 mmol), *t*-BuOOH (110 μL, 4.0 equiv.) and BHT (16.5 mg, 0.075 mmol) were employed. The reaction was monitored by HPLC, after stirring at 30 ˚C for 2 h, afforded **13a** (40.3%), **12a** (7.2%) and **10a** (3.2%). (For HPLC traces, see Supplementary Figures 50-54).

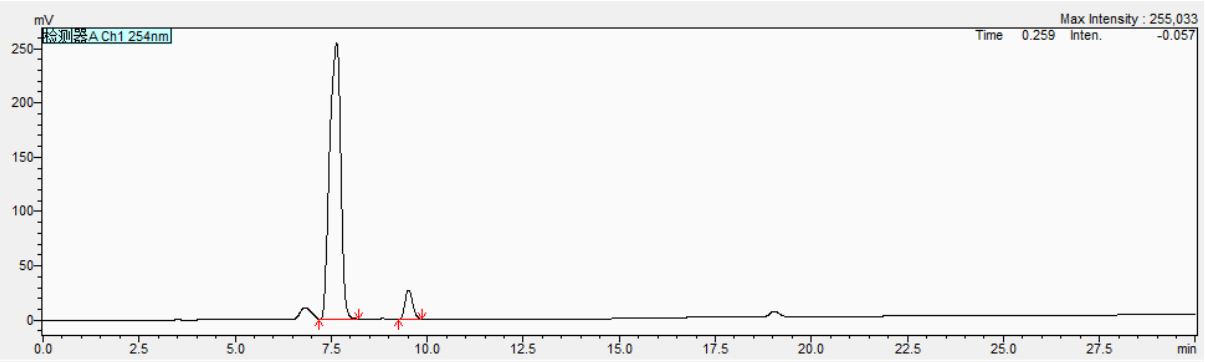

Peak No.	Retention time	Area	Area (%)	Height	Height (%)	S/N
1	7.643	5074909	93.266	253953	90.469	693.29
2	9.511	366391	6.734	26754	9.531	73.04
Total		5441301	100.00	280707	100.00	

Supplementary Figure 50. The reaction progress with 50 mol% BHT. HPLC trace at 5 min. Retention time: **11a** 7.6 min (93.2%); **12a** 9.5 min (6.7%).

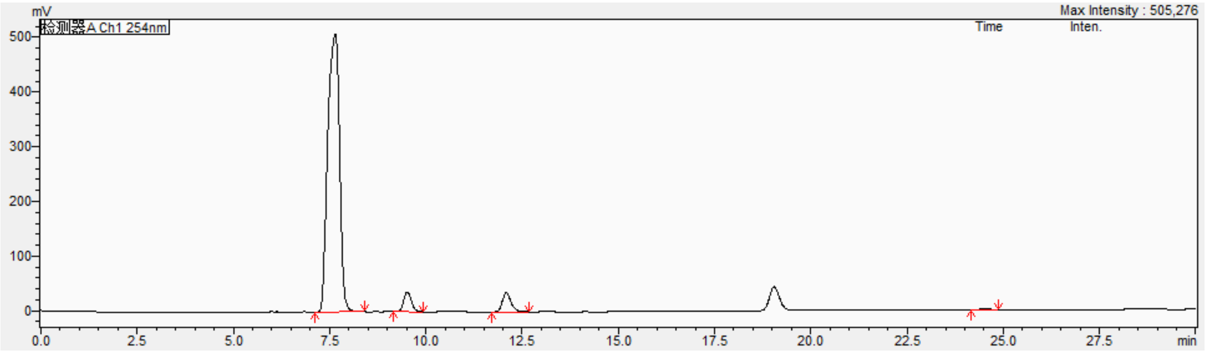

Peak No.	Retention time	Area	Area (%)	Height	Height (%)	S/N
1	7.646	10808480	90.845	507366	87.402	441.40
2	9.515	507876	4.269	36021	6.205	31.34
3	12.087	545383	4.584	35194	6.063	30.62
4	24.530	36041	0.303	1913	0.330	1.62
Total		11897780	100.00	580494	100.00	

Supplementary Figure 51. The reaction progress with 50 mol% BHT. HPLC trace at 0.5 h. Retention time: **11a** 7.7 min (90.8%) (Conversion rate 9.2%); **12a** 9.5 min (4.3% yield); **13a** 12.1 min (4.6% yield); **10a** 24.5 min (0.3% yield).

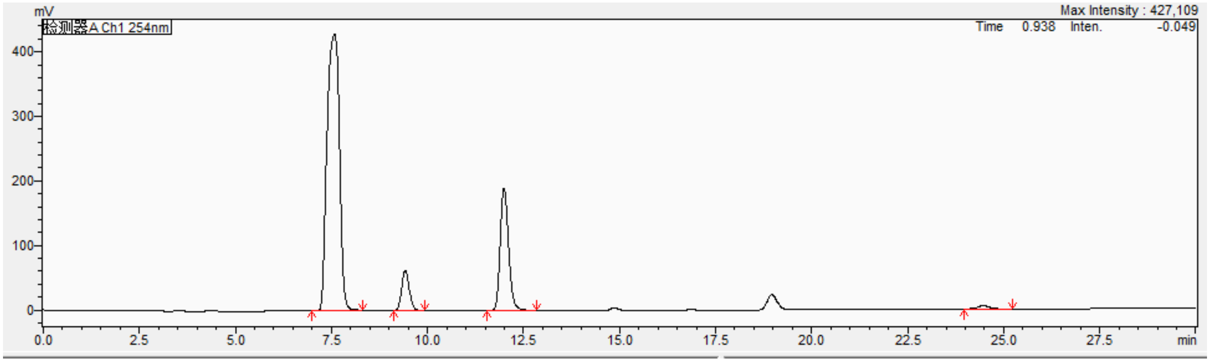

Peak No.	Retention time	Area	Area (%)	Height	Height (%)	S/N
1	7.583	9577882	71.062	428234	62.424	483.20
2	9.422	877478	6.510	62870	9.165	70.94
3	11.992	2907524	21.572	189839	27.673	214.21
4	24.473	115332	0.856	5063	0.738	5.71
Total		13478216	100.00	686006	100.00	

Supplementary Figure 52. The reaction progress with 50 mol% BHT. HPLC trace at 1.0 h. Retention time: **11a** 7.6 min (71.1%) (Conversion rate 28.9%); **12a** 9.4 min (6.5% yield); **13a** 12.0 min (21.6% yield); **10a** 24.5 min (0.9% yield).

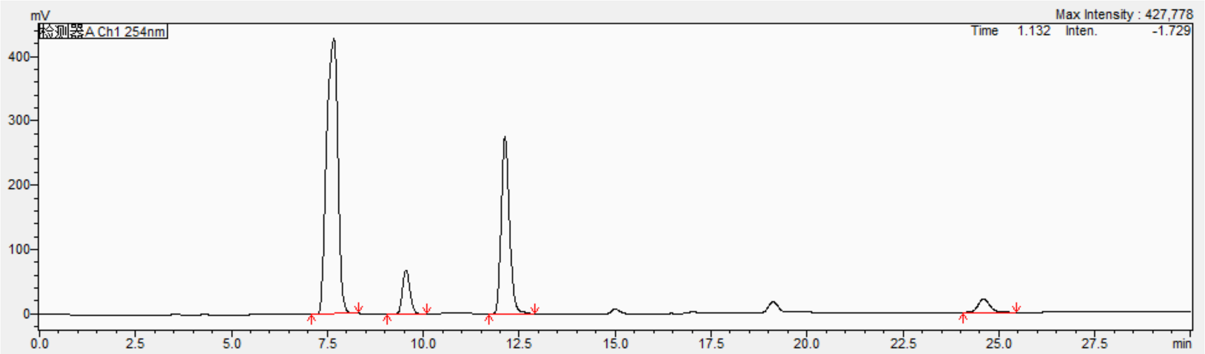

Peak No.	Retention time	Area	Area (%)	Height	Height (%)	S/N
1	7.666	8759148	61.260	427675	53.992	500.37
2	9.547	9453437	6.578	68621	8.663	80.29
3	12.128	4153437	29.049	274939	34.710	321.67
4	24.604	445149	3.113	20876	2.636	24.43
Total		14298244	100.00	792111	100.00	

Supplementary Figure 53. The reaction progress with 50 mol% BHT. HPLC trace at 1.5 h. Retention time: **11a** 7.7 min (61.3%) (Conversion rate 38.7%); **12a** 9.5 min (6.6% yield); **13a** 12.1 min (29.0% yield); **10a** 24.6 min (3.1% yield).

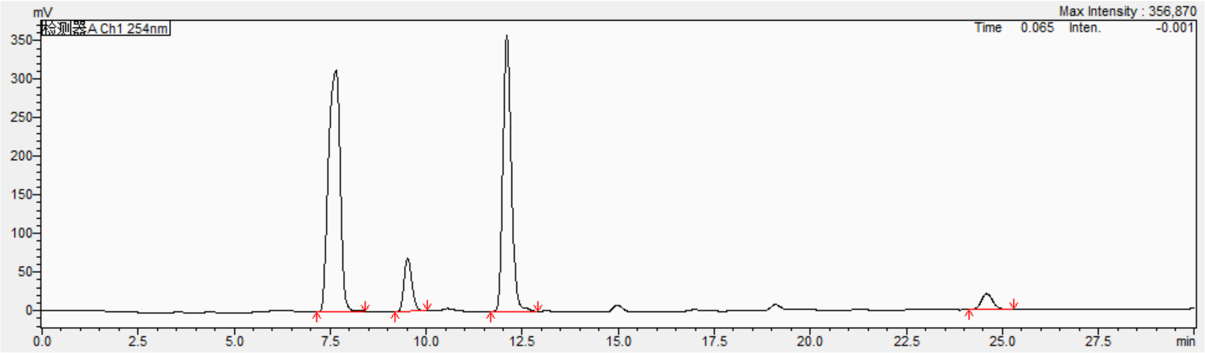

Peak No.	Retention time	Area	Area (%)	Height	Height (%)	S/N
1	7.648	6645348	49.212	312222	41.125	477.52
2	9.516	977403	7.238	68589	9.034	104.90
3	12.097	5446535	40.334	357747	47.121	547.14
4	24.595	434176	3.215	20648	2.720	31.58
Total		13503461	100.00	759206	100.00	

Supplementary Figure 54. The reaction progress with 50 mol% BHT. HPLC trace at 2.0 h. Retention time: **11a** 7.6 min (49.2%) (Conversion rate 50.8%); **12a** 9.5 min (7.2% yield); **13a** 12.1 min (40.3% yield); **10a** 24.6 min (3.2% yield).

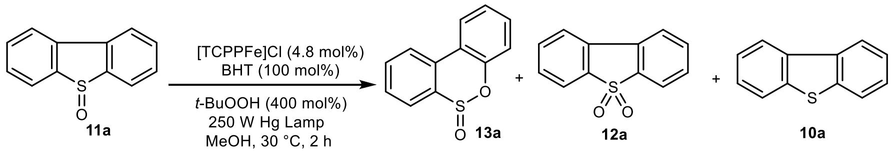

Supplementary Figure 55. Control experiment in the presence of 100 mol% BHT. Supplementary Table 1, entry 10, according to **General procedure C: 11a** (30.1 mg, 0.15 mmol), *t*-BuOOH (110 μL, 4.0 equiv.) and BHT (33.1 mg, 0.15 mmol) were employed. The reaction was monitored by HPLC, after stirring at 30 ˚C for 2 h, afforded **13a** (52.7%), **12a** 13.3%) and **10a** (3.1%). (For HPLC traces, see Supplementary Figures 56-60).

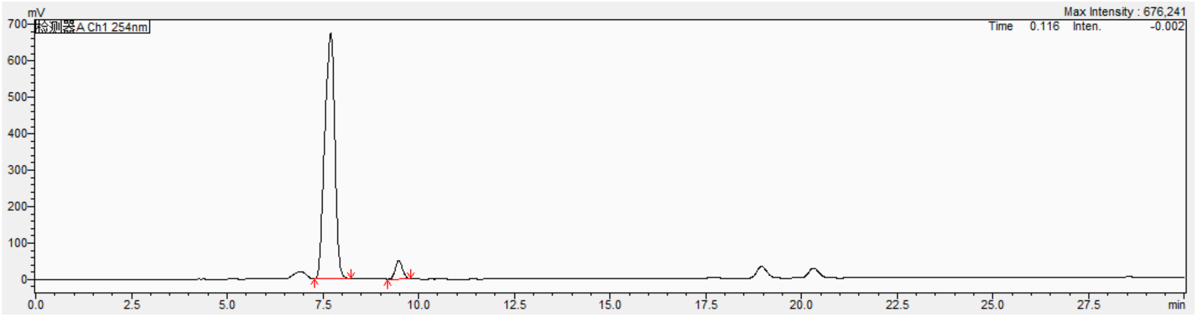

Peak No.	Retention time	Area	Area (%)	Height	Height (%)	S/N
1	7.706	12290995	94.885	674452	93.119	440.28
2	9.479	662598	5.115	49841	6.881	32.54
Total		12953594	100.00	724294	100.00	

Supplementary Figure 56. The reaction progress with 100 mol% BHT. HPLC trace at 0 h. Retention time: **11a** 7.7 min (94.9%); **12a** 9.5 min (5.1%).

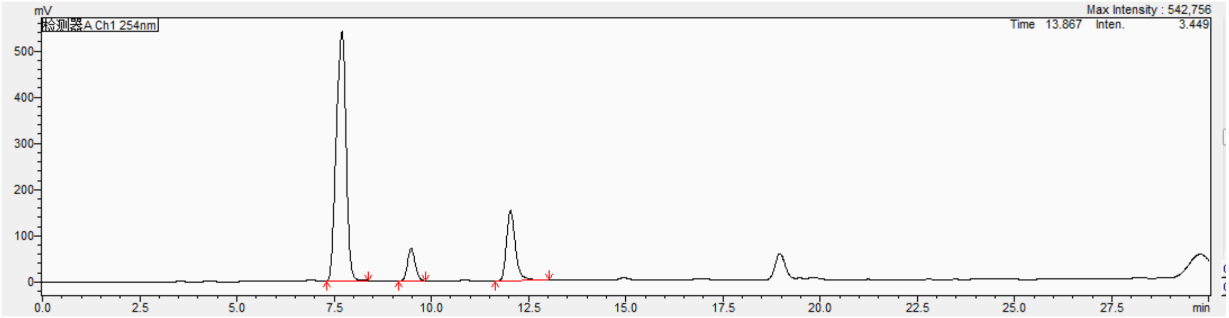

Peak No.	Retention time	Area	Area (%)	Height	Height (%)	S/N
1	7.702	9428639	73.671	540511	70.940	322.79
2	9.479	956555	7.474	69763	9.156	41.66
3	12.034	2413180	18.855	151655	19.904	90.57
Total		12798374	100.00	761929	100.00	

Supplementary Figure 57. The reaction progress with 100 mol% BHT. HPLC trace at 0.5 h. Retention time: **11a** 7.7 min (73.7%) (Conversion rate 26.3%); **12a** 9.5 min (7.5% yield); **13a** 12.0 min (18.9% yield).

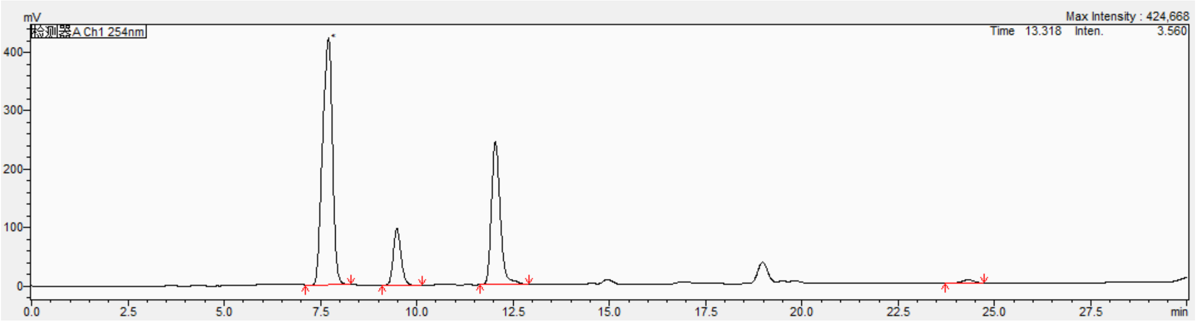

Peak No.	Retention time	Area	Area (%)	Height	Height (%)	S/N
1	7.710	7483916	58.230	421606	54.799	318.91
2	9.484	1366722	10.634	97178	12.631	73.51
3	12.041	3886572	30.240	245251	31.877	185.51
4	24.322	115058	0.895	5334	0.693	4.03
Total		12852268	100.00	769369	100.00	

Supplementary Figure 58. The reaction progress with 100 mol% BHT. HPLC trace at 1.0 h. Retention time: **11a** 7.7 min 58.2%) (Conversion rate 41.8%); **12a** 9.5 min (10.6% yield); **13a** 12.0 min (30.2% yield); **10a** 24.3 min (0.9% yield).

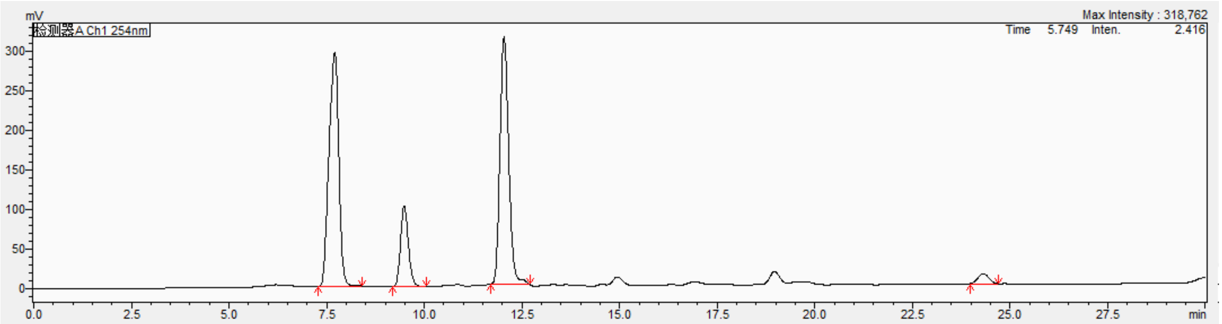

Peak No.	Retention time	Area	Area (%)	Height	Height (%)	S/N
1	7.708	5164575	44.009	296652	40.956	274.12
2	9.484	1423833	12.133	101439	14.005	93.73
3	12.040	4897717	41.735	313530	43.286	289.71
4	24.312	249251	2.124	12693	1.752	11.73
Total		11735377	100.00	724314	100.00	

Supplementary Figure 59. The reaction progress with 100 mol% BHT. HPLC trace at 1.5 h. Retention time: **11a** 7.7 min (44.0%) (Conversion rate 56.0%); **12a** 9.5 min (12.1% yield); **13a** 12.0 min (41.7% yield); **10a** 24.3 min (2.1% yield).

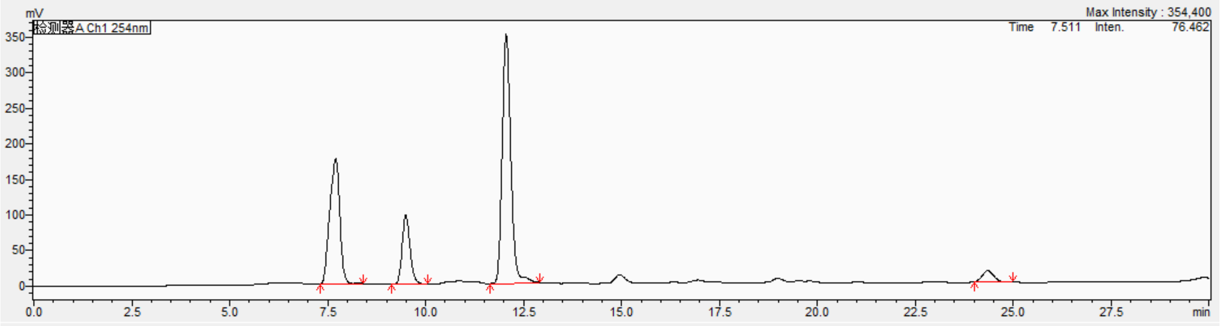

Peak No.	Retention time	Area	Area (%)	Height	Height (%)	S/N
1	7.707	3146637	30.888	175870	27.540	220.16
2	9.493	1354776	13.299	96672	15.138	121.02
3	12.054	5367320	52.686	350261	54.847	438.46
4	24.349	318568	3.127	15806	2.475	19.79
Total		10187302	100.00	638609	100.00	

Supplementary Figure 60. The reaction progress with 100 mol% BHT. HPLC trace at 2.0 h. Retention time: **11a** 7.7 min (30.9%) (Conversion rate 69.1%); **12a** 9.5 min (13.3% yield); **13a** 12.1 min (52.7% yield); **10a** 24.3 min (3.1% yield).

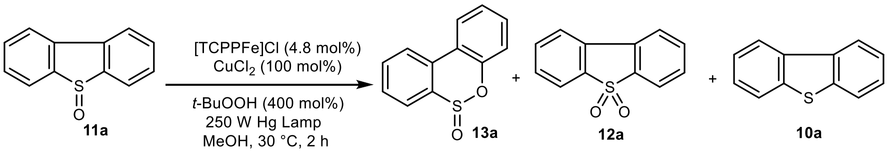

Supplementary Figure 61. Control experiment in the presence of 100 mol% $CuCl_2$. Supplementary Table 1, entry 11, according to **General procedure C: 11a** (30.1 mg, 0.15 mmol), *t*-BuOOH (82 μL, 3.0 equiv.) and $CuCl_2$ (20.2 mg, 0.15 mmol) were employed. The reaction was monitored by HPLC, after stirring at 30 ˚C for 2 h, afforded **13a** (58.8%), **12a** (4.2%) and **10a** (20.8%). (For HPLC traces, see Supplementary Figures 62-65).

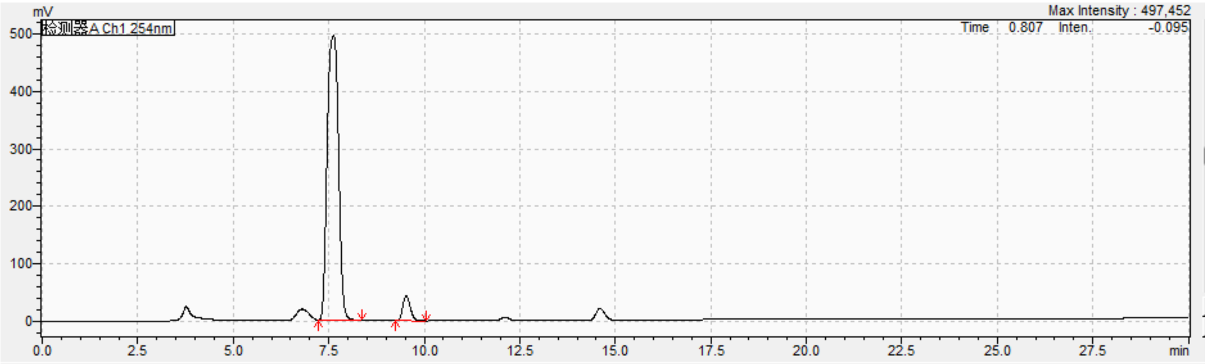

Peak No.	Retention time	Area	Area (%)	Height	Height (%)	S/N
1	7.624	10081739	94.505	495547	91.895	379
2	9.527	586243	5.495	43706	8.105	33
Total		10667982	100.00	539253	100.00	

Supplementary Figure 62. The reaction progress with 100 mol% $CuCl_2$. HPLC trace at 5 min. Retention time: **11a** 7.6 min (94.6%); **12a** 9.5 min (5.5% yield).

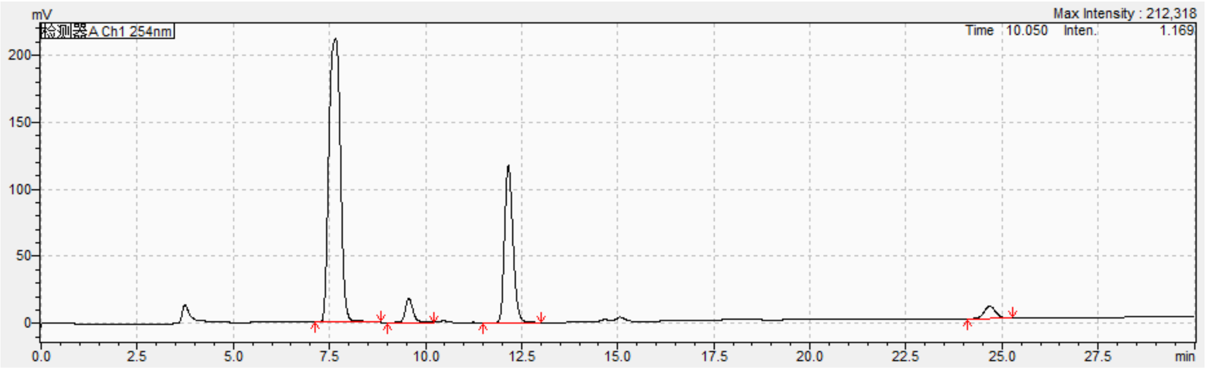

Peak No.	Retention time	Area	Area (%)	Height	Height (%)	S/N
1	7.658	4365197	66.151	211498	59.458	398
2	9.565	259583	3.934	17829	5.012	33
3	12.159	1783899	27.034	117327	32.984	221
4	24.680	190121	2.881	9057	2.546	17
Total		6598800	100.00	355711	100.00	

Supplementary Figure 63. The reaction progress with 100 mol% $CuCl_2$. HPLC trace at 0.5 h. Retention time: **11a** 7.7 min (66.2%) (Conversion rate 33.8%); **12a** 9.6 min (3.9% yield); **13a** 12.2 min (27.0% yield); **10a** 24.7 min (2.9% yield).

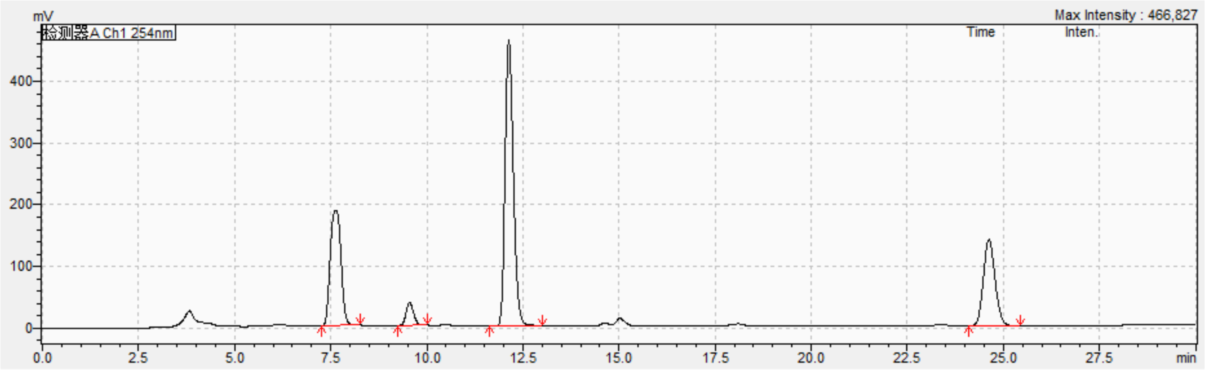

Peak No.	Retention time	Area	Area (%)	Height	Height (%)	S/N
1	7.631	3602123	25.755	187003	22.615	165
2	9.551	501426	3.585	37014	4.476	32
3	12.138	6965972	49.805	463343	56.034	410
4	24.626	2916850	20.855	139532	16.874	123
Total		13986370	100.00	826892	100.00	

Supplementary Figure 64. The reaction progress with 100 mol% $CuCl_2$. HPLC trace at 1.0 h. Retention time: **11a** 7.6 min (25.8%) (Conversion rate 74.2%); **12a** 9.6 min (3.6% yield); **13a** 12.1 min (49.8% yield); **10a** 24.6 min (20.9% yield).

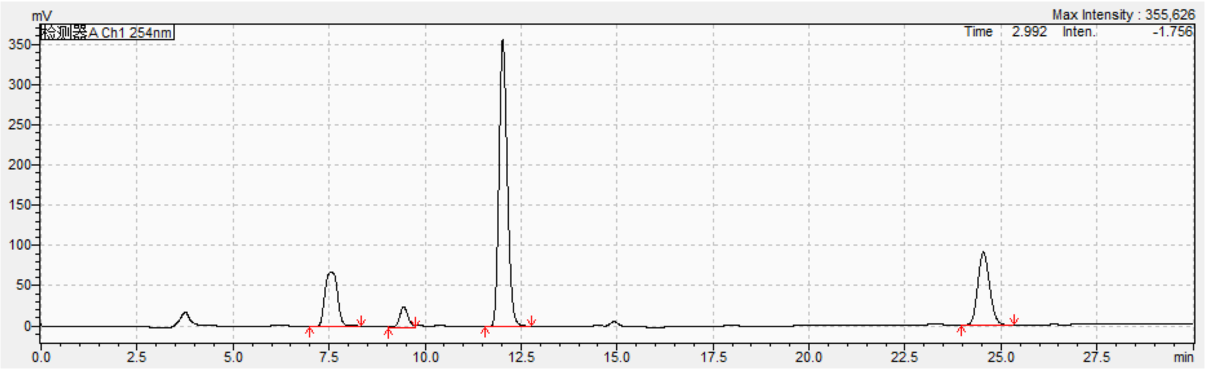

Peak No.	Retention time	Area	Area (%)	Height	Height (%)	S/N
1	7.544	1508140	16.232	66905	12.392	75
2	9.443	387134	4.167	25738	4.767	29
3	12.025	5464102	58.810	356487	66.025	402
4	24.544	1931757	20.791	90794	16.816	102
Total		9291132	100.00	539924	100.00	

Supplementary Figure 65. The reaction progress with 100 mol% $CuCl_2$. HPLC trace at 2.0 h. Retention time: **11a** 7.5 min (16.2%) (Conversion rate 83.8%); **12a** 9.4 min (4.2% yield); **13a** 12.0 min (58.8% yield); **10a** 24.5 min (20.8% yield).

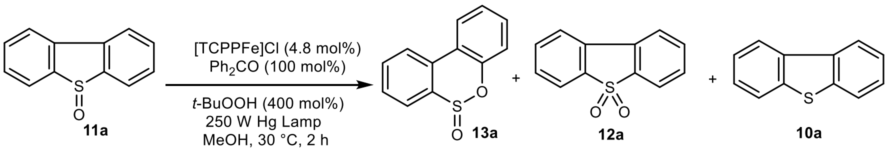

Supplementary Figure 66. Control experiment in the presence of 100 mol% Ph_2CO. Supplementary Table 1, entry 12, according to **General procedure C: 11a** (30.1 mg, 0.15 mmol), *t*-BuOOH (110 μL, 4.0 equiv.) and benzophenone (27.3 mg, 0.15 mmol) were employed. The reaction was monitored by HPLC, after stirring at 30 °C for 2 h, afforded **13a** (82.1%), **12a** (10.5%) and **10a** (3.3%). (For HPLC traces, see Supplementary Figures 67-71).

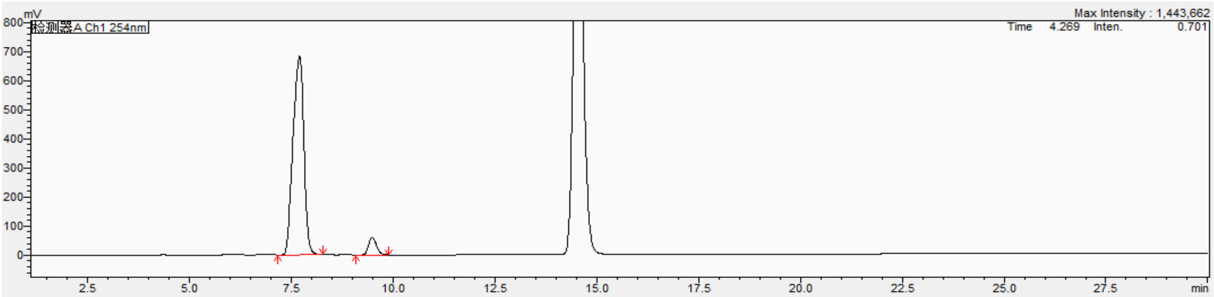

Peak No.	Retention time	Area	Area (%)	Height	Height (%)	S/N
1	7.697	12712647	93.963	683976	91.846	21.59
2	9.481	816780	6.037	60723	8.154	1.92
Total		13529427	100.00	744699	100.00	

Supplementary Figure 67. The reaction progress with 100 mol% Ph_2CO. HPLC trace at 0 h. Retention time: **11a** 7.7 min (94.0%); **12a** 9.5 min (6.0%).

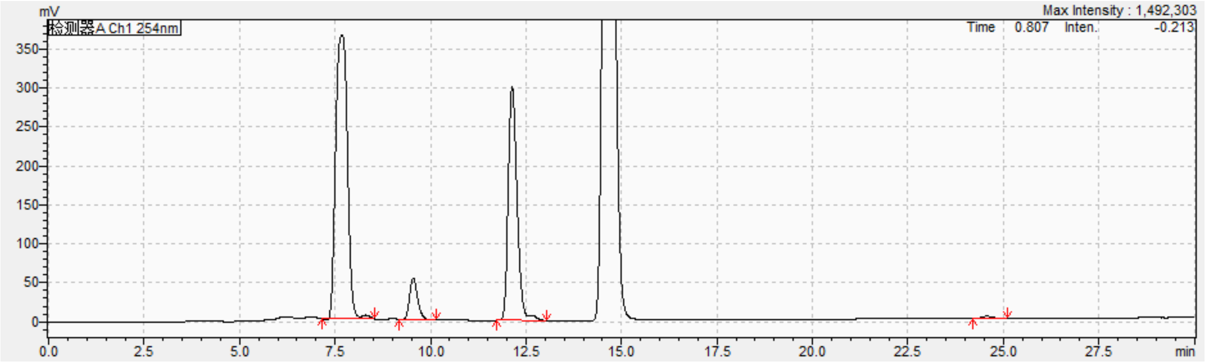

Peak No.	Retention time	Area	Area (%)	Height	Height (%)	S/N
1	7.686	7779278	57.717	364249	50.519	10
2	9.547	756556	5.613	52926	7.341	1
3	12.141	4874496	36.166	300787	41.717	8
4	24.572	67916	0.504	3053	0.423	0
Total		13478246	100.00	721015	100.00	

Supplementary Figure 68. The reaction progress with 100 mol% Ph_2CO. HPLC trace at 0.5 h. Retention time: **11a** 7.7 min (57.7%) (Conversion rate 42.3%); **12a** 9.5 min (5.6% yield); **13a** 12.1 min (36.2% yield); **10a** 24.6 min (0.5% yield).

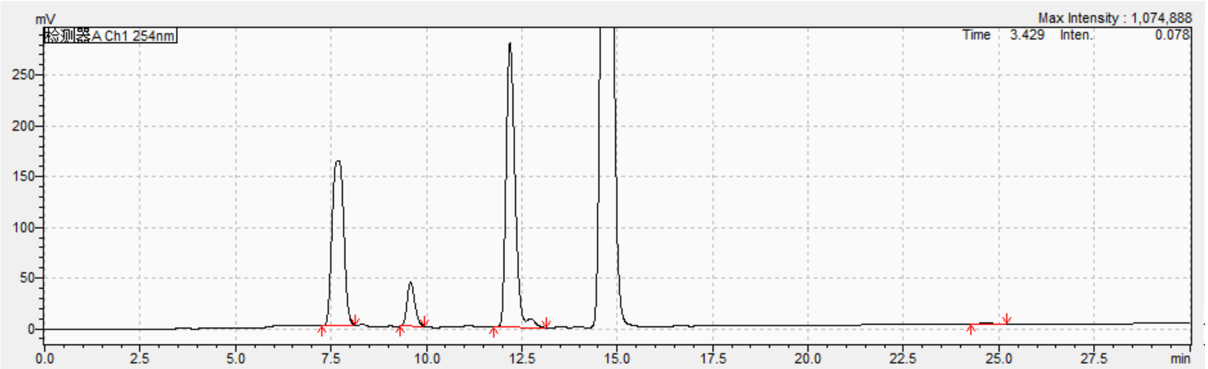

Peak No.	Retention time	Area	Area (%)	Height	Height (%)	S/N
1	7.689	3411340	39.592	162587	33.332	7
2	9.587	602760	6.996	43175	8.851	2
3	12.195	4558829	52.909	280133	57.430	13
4	24.670	43359	0.503	1891	0.388	0
Total		8616287	100.00	487785	100.00	

Supplementary Figure 69. The reaction progress with 100 mol% Ph_2CO. HPLC trace at 1.0 h. Retention time: **11a** 7.7 min (39.6%) (Conversion rate 60.4%); **12a** 9.6 min (7.0% yield); **13a** 12.2 min (52.9% yield); **10a** 24.7 min (0.5% yield).

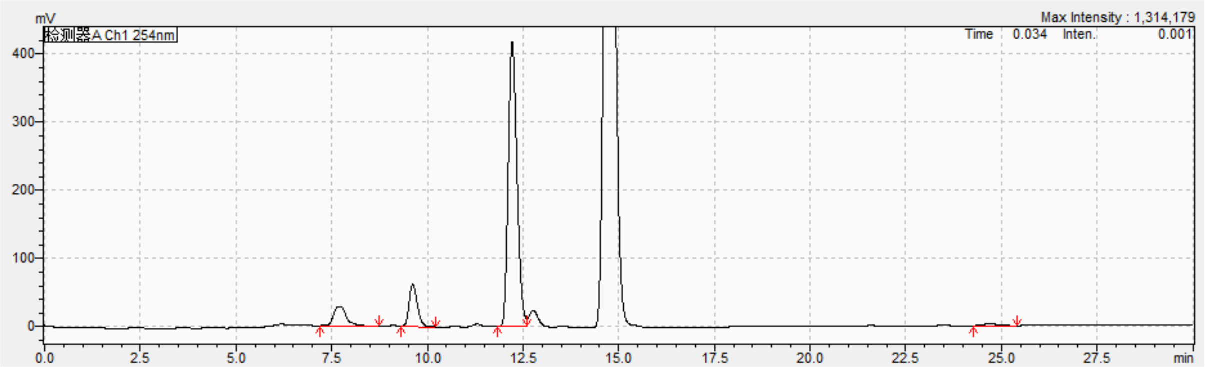

Peak No.	Retention time	Area	Area (%)	Height	Height (%)	S/N
1	7.709	673602	8.209	28455	5.584	0
2	9.619	909203	11.080	61825	12.133	1
3	12.220	6554885	79.882	416358	81.706	12
4	24.699	68038	0.829	2942	0.577	0
Total		8205728	100.00	509581	100.00	

Supplementary Figure 70. The reaction progress with 100 mol% Ph_2CO. HPLC trace at 1.5 h. Retention time: **11a** 7.7 min (8.2%) (Conversion rate 91.8%); **12a** 9.6 min (11.1% yield); **13a** 12.2 min (79.9% yield); **10a** 24.7 min (0.8% yield).

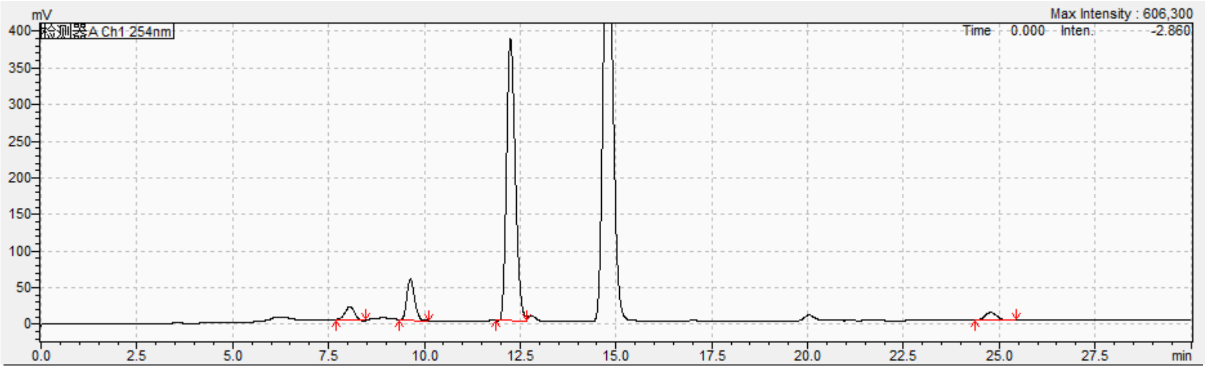

Peak No.	Retention time	Area	Area (%)	Height	Height (%)	S/N
1	8.062	306998	4.162	17940	3.815	1
2	9.635	772776	10.476	55928	11.894	4
3	12.251	6057212	82.110	385432	81.967	28
4	24.774	239938	3.253	10928	2.324	0
Total		7376924	100.00	470227	100.00	

Supplementary Figure 71. The reaction progress with 100 mol% Ph_2CO. HPLC trace at 2.0 h. Retention time: **11a** 8.1 min (4.2%) (Conversion rate 95.8%); **12a** 9.6 min (10.5% yield); **13a** 12.3 min (82.1% yield); **10a** 24.8 min (3.3% yield).

Synthesis of 2-(2-hydroxybiphenyl)-benzenesulfinate (14a)

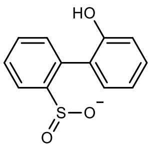

14a

A suspension of **13a** (200 mg, 0.925 mmol) in KPi buffer (0.05 M, pH = 7.5) and THF (V:V = 10:1, 22 mL) was stirred at room temperature for 10 h, and then the solution was heated at 80 ˚C for 48 h. The reaction was cooled to room temperature then was extracted with 1-butanol (4 × 30 mL). The combined organic layers were washed with brine, dried over Na_2SO_4, filtered, and the solvent was evaporated under reduced pressure to give **14a** (231 mg, 92%) as a colorless solid. ^{1}H NMR (400 MHz, DMSO-d_6) δ 10.87 (s, 1H), 7.77 (dd, *J* = 7.5, 1.5 Hz, 1H), 7.40 (t, *J* = 7.5 Hz, 1H), 7.31 (td, *J* = 7.5, 1.5 Hz, 1H), 7.20 (td, *J* = 7.5, 1.5 Hz, 1H), 7.06 (dd, *J* = 7.5, 1.5 Hz, 1H), 6.93 (d, *J* = 7.5 Hz, 1H), 6.89 (t, *J* = 7.5 Hz, 1H), 6.81 (d, *J* = 7.5 Hz, 1H); ^{13}C NMR (101 MHz, DMSO-d_6) δ 155.6, 154.4, 136.4, 132.2, 131.0, 130.9, 128.6, 128.0, 127.0, 121.6, 120.1, 119.7; IR (film): 3338-2412 (br), 1565, 1059, 971, 924, 844, 834 cm^{-1}; ESI-HRMS (m/z): [M-H]$^-$ calcd for $C_{12}H_{10}O_3S$: 233.0272; found: 233.0273. ESI-MS: 233.0 (M-H); 169.1 (M-H-SO_2).

Synthesis of 2'-hydroxy-5,5'-dimethyl-[1,1'-biphenyl]-2-sulfinate (14e)

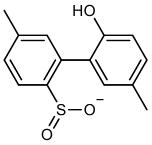

14e

A suspension of **13e** (100 mg, 0.409 mmol) in KPi buffer and THF (V:V = 10:1, 11 mL) was stirred at room temperature for 10 h, and then the solution was heated at 80 ˚C for 48 h. The reaction was cooled to room temperature then was extracted with ethyl acetate (5 × 30 mL). The combined organic layers were washed with brine, dried over Na_2SO_4, filtered, and the solvent was evaporated under reduced pressure to give **14e** (117 mg, 95%) as a colorless solid. ^{1}H NMR (400 MHz, DMSO-d_6) δ 10.67 (s, 1H), 7.62 (d, *J* = 8.0 Hz, 1H), 7.18 (d, *J* = 8.0 Hz, 1H), 6.98 (dd, *J* = 8.0, 2.0 Hz, 1H), 6.84 (d, *J* = 2.0 Hz, 1H), 6.72 (br s, 1H), 6.67 (d, *J* = 8.0 Hz, 1H), 2.29 (s, 3H), 2.23 (s, 3H); ^{13}C NMR (101 MHz, DMSO-d_6) δ 152.0, 137.0, 136.5, 131.5, 131.2, 129.0, 128.0, 127.4, 121.8, 120.0, 20.6, 20.2; IR (film): 3361-2710 (br), 1516, 1244, 950, 813, 778 cm^{-1}; ESI-HRMS (m/z): [M-H]$^-$ calcd for $C_{14}H_{14}O_3S$: 261.0585; found: 261.0583; ESI-MS: 261.1 (M-H); 197.1 (M-H-SO_2).

Synthesis of [1,1'-biphenyl]-2-ol (15a)

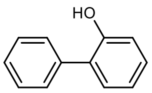

15a

A stirring solution of **13a** (21.6 mg, 0.10 mmol) and *t*-BuOK (22.4 mg, 0.20 mmol) in DMF (1.0 mL) was heated at 110 °C for 24 h, the solvent was evaporated under reduced pressure to give a crude product, which was purified by flash SiO_2 gel column chromatography (PE: EA = 10:1 to 5:1), **15a** (16.7 mg, 98%) was obtained as a colorless solid. ^1H NMR (400 MHz, $CDCl_3$) δ 7.55 – 7.44 (m, 4H), 7.43 – 7.36 (m, 1H), 7.30 – 7.21 (m, 2H), 7.03 – 6.96 (m, 2H), 5.20 (s, 1H); ^{13}C NMR (101 MHz, $CDCl_3$) δ 152.5, 137.2, 130.4, 129.5, 129.3, 129.2, 128.1, 121.0, 115.9, 100.1. The spectroscopic properties of **15a** were consistent with the data available in the literature.[19]

Synthesis of 3',5-dimethyl-[1,1'-biphenyl]-2-ol (15e).

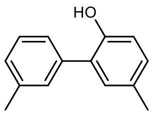

15e

A stirring solution of **13e** (24.6 mg, 0.10 mmol) and K_2CO_3 (27.6 mg, 0.20 mmol) in CH_3CN(1.0 mL) and H_2O (0.1 mL) was heated at 110 °C for 36 h, the solvent was evaporated under reduced pressure to give a crude product, which was purified by flash SiO_2 gel column chromatography (PE: EA = 10:1 to 5:1), **15e** (18.4 mg, 93%) was obtained as a colorless solid. ^1H NMR (400 MHz, $CDCl_3$) δ 7.38 (t, *J* = 7.5 Hz, 1H), 7.27– 7.25(m, 2H), 7.21 (d, *J* = 7.5 Hz, 1H), 7.06 (d, *J* = 8.0 Hz, 2H), 6.89 (d, *J* = 8.0 Hz, 1H), 5.11 (s, 1H), 2.42 (s, 3H), 2.32 (s, 3H); ^{13}C NMR (101 MHz, $CDCl_3$) δ 150.3, 139.2, 137.2, 130.7, 130.0, 129.9, 129.7, 129.6, 129.2, 128.7, 128.0, 115.6, 21.6, 20.6; IR (film): 3544 (br), 2988, 2956, 2923, 14653, 1276, 1261, 764, 750 cm^{-1}; APCI-HRMS (m/z): $[M+H]^+$calcd for $C_{14}H_{14}O$: 199.1117; found: 199.1112.

Synthesis of [1,1'-biphenyl-*d*3]-2-ol (16).

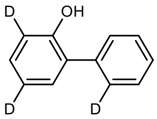

16

A stirring solution of **13a** (21.6 mg, 0.10 mmol) and K_2CO_3 (27.6 mg, 0.20 mmol) in CD_3CN (1.0 mL) and D_2O (0.1 mL) was heated at 110 °C for 48 h, the solvent was evaporated under reduced pressure to give a crude product, which was purified by flash SiO_2 gel column chromatography (PE: EA = 10:1 to 5:1), **16** (16.1 mg, 90%) was obtained as a colorless syrup. ^1H NMR (400 MHz, $CDCl_3$) δ 7.46 – 7.36 (m, 3H), 7.36 – 7.28 (m, 1H), 7.18 (d, *J* = 5.0 Hz, 2H), 5.12 (s, 1H); ^{13}C NMR (101 MHz, $CDCl_3$) δ 152.5, 137.1, 130.3, 129.4, 129.3, 129.2, 129.1, 128.2, 128.0, 120.9; IR (film): 3553 (br), 3005, 2989, 1453, 1275, 1261, 764, 750 cm^{-1}; APCI-HRMS (m/z): $[M+H]^+$ calcd for $C_{12}H_7D_3O$: 174.0993; found: 174.0994.

Synthesis of 3',5-bimethyl-[1,1'-biphenyl-*d*$_2$]-2-ol (17).

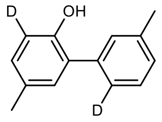

17

A stirring solution of **13e** (24.6 mg, 0.10 mmol) and K_2CO_3 (27.6 mg, 0.20 mmol) in CD_3CN (1.0 mL) and D_2O (0.1 mL) was heated at 110 °C for 48 h, the solvent was evaporated under reduced pressure to give a crude product, which was purified by flash SiO_2 gel column chromatography (PE: EA = 10:1 to 5:1), **17** (19.2 mg, 96%) was obtained as a colorless syrup. ^{1}H NMR (400 MHz, $CDCl_3$) δ 7.36 (dt, *J* = 7.0, 3.5 Hz, 1H), 7.26 (s, 1H), 7.22 – 7.17 (m, 1H), 7.08 – 7.01 (m, 2H), 6.87 (d, *J* = 8.0 Hz, 0.25H), 5.09 (s, 1H), 2.41 (s, 3H), 2.31 (s, 3H). ^{13}C NMR (101 MHz, $CDCl_3$) δ 150.3, 139.2, 137.2, 130.7, 130.0, 129.9, 129.6, 129.3, 129.2, 128.7, 128.0, 115.6, 21.6, 20.6; IR (film): 3536 (br), 3005, 2988, 1474, 1463, 1275, 1260, 764, 750 cm^{-1}; APCI-HRMS (m/z): [M+H]$^+$calcd for $C_{14}H_{13}D_2O$: 201.1243; found: 201.1245.

Synthesis of 2'-(benzylsulfinyl)-[1,1'-biphenyl]-2-ol (18)

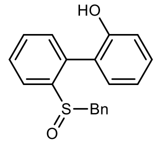

18

Benzylmagnesium chloride (0.12 mmol) in ether was added dropwise to a solution of **13a** (21.6 mg, 0.10 mmol) in Et_2O (2.0 mL) at -78 °C. The solution was stirred at same temperature for 3 h, then the reaction mixture was warmed to 0 °C, benzylmagnesium chloride (60 μmol) was added, and the resulting mixture was stirred at 0 °C for another 30 min, saturated NH_4Cl (0.3 mL) was added, the solvent was evaporated under reduced pressure to give a crude product, which was purified by flash SiO_2 gel column chromatography (PE: EA = 5:1 to 2:1), **18** (28.4 mg, 92%) was obtained as a colorless oil. ^{1}H NMR (400 MHz, CD_3OD) δ 7.69 (d, *J* = 6.0 Hz, 1H), 7.59 (td, *J* = 7.5, 1.5 Hz, 1H), 7.53 (t, *J* = 7.0 Hz, 1H), 7.35 – 7.17 (m, 5H), 7.12 – 6.77 (m, 5H), 4.10 (br s, 1H), 3.76 (d, *J* = 13.0 Hz, 1H); ^{13}C NMR (101 MHz, CD_3OD) δ 151.3, 145.4, 139.0, 134.0, 132.4, 131.6, 131.4, 129.5 (2), 129.3, 126.2, 125.2, 121.1, 116.9, 107.8, 30.8; IR (film): 3152 (br), 2924, 2854, 1609, 1456, 1449, 1276, 1261, 1013, 1001, 764, 750 cm^{-1}; ESI-HRMS: m/z calcd for $C_{19}H_{16}O_2S$[M+H]$^+$: 309.0944; found: 309.0945.

Synthesis of 2'-(phenylsulfinyl)-[1,1'-biphenyl]-2-ol (19)

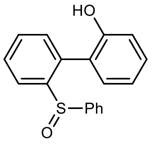

19

Phenylmagnesium bromide (0.12 mmol) in ether was added dropwise to a solution of **13a** (21.6 mg, 0.10 mmol) in Et_2O (2.0 mL) at -78 °C. The solution was stirred at same temperature for 45 min, saturated NH_4Cl (0.3 mL) was added, the solvent was evaporated under reduced pressure to give a crude product, which was purified by flash SiO_2 gel column chromatography (PE: EA = 5:1 to 2:1), **19** (25.9 mg, 88%) was obtained as a colorless solid. ^1H NMR (500 MHz, CD_3OD) δ 8.12 – 7.65 (m, 1H), 7.66 – 7.50 (m, 2H), 7.52 – 7.05 (m, 8H), 6.89 – 6.68 (m, 3H); ^{13}C NMR (126 MHz, CD_3OD) δ 145.1, 143.1, 132.7, 132.6, 132.4, 131.3, 130.3, 129.8, 126.6, 120.9, 116.9, 115.6; IR (film): 3554 (br), 2989, 1607, 1470, 1448, 1275, 1261, 764 cm^{-1}; ESI-HRMS (m/z): $[M+H]^+$ calcd for $C_{18}H_{15}O_2S$: 295.0787; found: 295.0787.

Synthesis of 2'-(methylsulfonyl)-[1,1'-biphenyl]-2-ol (20)

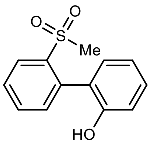

20

A stirring solution of **13a** (21.6 mg, 0.10 mmol) and NaOH (4.80 mg, 0.12 mmol) in DMF (1.0 mL) was stirred at room temperature for 8 h, then MeI (7.5 μL, 0.12 mmol) was added, the resulting mixture was stirred at room temperature for another 16 h, the solvent was evaporated under reduced pressure to give a crude product, which was purified by flash SiO_2 gel column chromatography (PE: EA = 8:1 to 3:1), **20** (20.6 mg, 83%) was obtained as a colorless solid. ^1H NMR (500 MHz, $CDCl_3$) δ 8.24 (dd, J = 8.0, 1.5 Hz, 1H), 7.71 (td, J = 7.5, 1.5 Hz, 1H), 7.62 (td, J = 7.5, 1.5 Hz, 1H), 7.41 – 7.34 (m, 2H), 7.16 (dd, J = 7.5, 1.5 Hz, 1H), 7.11 – 7.03 (m, 2H), 5.63 (s, 1H), 2.78 (s, 3H); ^{13}C NMR (126 MHz, $CDCl_3$) δ 153.4, 137.4, 134.1, 133.6, 131.1, 130.9, 128.9, 128.6, 121.5, 119.3, 43.0; IR (film): 3400 (br), 3005, 2990, 1470, 1449, 1276, 1261, 1148, 764, 750 cm^{-1}; ESI-HRMS (m/z): $[M+H]^+$calcd for$C_{13}H_{12}O_3S$: 249.0580; found: 249.0578.

Synthesis of 2-methoxy-2'-(methylsulfonyl)-1,1'-biphenyl (21)

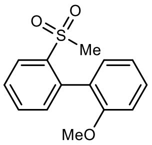

21

A stirring solution of **13a** (21.6 mg, 0.10 mmol) and NaOH (12.0 mg, 0.30 mmol) in DMF (1.0 mL) was stirred at room temperature for 8 h, then MeI (22.5 μL, 0.30 mmol) was added, the resulting mixture was stirred at room temperature for another 16 h, the solvent was evaporated under reduced pressure to give a crude product, which was purified by flash SiO_2 gel column chromatography (PE: EA = 10:1 to 3:1), **21** (24.9 mg, 95%) was obtained as a colorless solid. ^1H NMR (500 MHz, $CDCl_3$) δ 8.20 (dd, *J* = 8.0, 1.5 Hz, 1H), 7.63 (td, *J* = 7.5, 1.5 Hz, 1H), 7.55 (td, *J* = 8.0, 1.5 Hz, 1H), 7.42 (ddd, *J* = 8.5, 7.5, 2.0 Hz, 1H), 7.34 – 7.31 (m, 1H), 7.27 (dt, *J* = 4.0, 1.0 Hz, 1H), 7.04 (td, *J* = 7.5, 1.0 Hz, 1H), 6.98 (d, *J* = 8.5 Hz, 1H), 3.75 (s, 3H), 2.77 (s, 3H); ^{13}C NMR (126 MHz, $CDCl_3$) δ 156.7, 139.7, 138.4, 133.1, 133.0, 131.9, 130.2, 128.8, 128.1, 127.5, 120.2, 110.8, 55.6, 43.6; IR (film): 3066, 2840, 1601, 1499, 1485, 1468, 1306, 1278, 1249, 1149, 1122, 1024, 956, 758 cm^{-1}; ESI-HRMS (m/z): $[M+H]^+$ calcd for $C_{14}H_{15}O_3S$: 263.0736; found: 263.0740.

Synthesis of 2'-mercapto-5,5'-dimethyl-[1,1'-biphenyl]-2-ol (22)

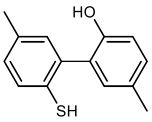

22

To a solution of **13e** (24.6 mg, 0.10 mmol) in Et_2O (2.0 mL) at 0 °C was added $LiAlH_4$ (11.4 mg, 0.30 mmol). The reaction was stirred at 0 °C for 4 h. Upon completion, water (0.1 mL), 15% aq. NaOH (0.1 mL) and a final portion of water (0.2 mL) were added. The mixture was filtered through Celite® and the filter cake was rinsed with Et_2O. The combined organic extracts were dried over Na_2SO_4, filtered and concentrated *in vacuo* to afford the crude product, by flash SiO_2 gel column chromatography (PE: EA = 10:1 to 3:1), **22** (22.6 mg, 98%) was obtained as a colorless oil. ^1H NMR (400 MHz, $CDCl_3$) δ 7.31 (d, *J* = 8.0 Hz, 1H), 7.14 – 7.06 (m, 3H), 6.95 (d, *J* = 2.0 Hz, 1H), 6.92 (d, *J* = 8.5 Hz, 1H), 4.81 (br, s, 1H), 3.36 (s, 1H), 2.33 (s, 3H), 2.32 (s, 3H); ^{13}C NMR (101 MHz, $CDCl_3$) δ 150.3, 136.1, 135.2, 132.2, 130.8, 130.6, 130.3, 129.9, 129.6, 128.6, 126.9, 116.1, 21.0, 20.6; IR (film): 3564 (br), 3306 (br), 2926, 2853, 2560, 1498, 1472, 1183, 814, 772 cm^{-1}; APCI-HRMS (m/z): $[M+H]^+$ calcd for $C_{14}H_{15}OS$: 231.0838; found: 231.0828.

Synthesis of dibenzo[c,e][1,2]oxathiine 6,6-dioxide (23)

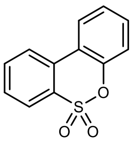

23

23 was prepared according to a literature procedure. To a stirred solution of 30% H_2O_2 (2.0 mL) in acetic acid (5.0 mL) was added BPS **13a** (100 mg, 0.46 mmol). The solution was refluxed for 23 h, cooled, and poured onto ice. The solid was dissolved in $CHCl_3$, and the $CHC1_3$ solution was

washed successively with water, saturated $NaHCO_3$, and water and dried (Na_2SO_4). Evaporation of the $CHCl_3$, gave the title compound as white solid (101.5 mg, 95%). ^1H NMR (300 MHz, $CDCl_3$) δ 7.95 (ddd, *J* = 7.8, 1.4, 0.5 Hz, 1H), 7.89 (ddd, *J* = 7.7, 2.4, 1.4 Hz, 2H), 7.71(ddd, *J* = 8.0, 7.4, 1.4 Hz, 1H), 7.53 (td, *J* = 7.7, 1.1 Hz, 1H), 7.46–7.33 (m, 2H),7.33–7.25 (m, 1H); ^{13}C NMR (75 MHz, $CDCl_3$) δ 149.8, 133.7, 132.4, 131.7, 131.2, 129.1, 126.7, 125.2, 124.8, 124.3, 121.6, 120.1; IR (film): 2925, 1474, 1454, 1427, 1361, 1201, 1172, 1136, 1070 cm^{-1}; ESI-HRMS (m/z): $[M+Na]^+$ calcd for $C_{12}H_8O_3NaS$: 255.0086; found: 255.0085.

Synthesis of 2'-(phenylsulfonyl)-[1,1'-biphenyl]-2-ol (24a)

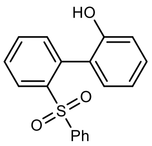

24a

Phenyllithium (0.12 mmol) in THF was added dropwise to a solution of **13a** (21.6 mg, 0.1 mmol) in THF (0.5 mL) at 0 °C. The solution was stirred at same temperature for 1 h, saturated NH_4Cl (0.3 mL) was added, the solvent was evaporated under reduced pressure to give a crude product, which was purified by flash SiO_2 gel column chromatography (PE: EA = 10:1 to 4:1), **24a** (19.2 mg, 62% yield) was obtained as a colorless solid. ^1H NMR (400 MHz, $CDCl_3$) δ 8.54 – 8.36 (m, 1H), 7.72 – 7.57 (m, 2H), 7.42 (t, *J* = 7.5 Hz, 1H), 7.26 – 7.19 (m, 6H), 6.91 (d, *J* = 8.0 Hz, 1H), 6.67 (t, *J* = 7.5 Hz, 1H), 6.41 (dd, *J* = 7.5, 1.0 Hz, 1H), 5.29 (s, 1H); ^{13}C NMR (101 MHz, $CDCl_3$) δ 153.4, 140.3, 139.8, 137.7, 134.0, 133.6, 133.0, 131.1 (2), 128.7 (2), 127.9, 127.2, 120.9, 118.3; IR (film): 3423 (br), 3007, 1448, 1276, 1261, 1151, 764, 750 cm^{-1}; ESI-HRMS (m/z): $[M+H]^+$ calcd for $C_{18}H_{15}O_3S$: 311.0736; found: 311.0738.

Synthesis of 2'-(butylsulfonyl)-[1,1'-biphenyl]-2-ol (24b)

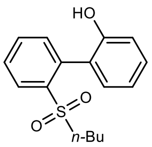

24b

n-BuLi (0.25 mmol) in THF was added dropwise to a solution of **13a** (21.6 mg, 0.1 mmol) in THF (1.0 mL) at -78 °C. The solution was stirred at same temperature for 1 h, saturated NH_4Cl (0.3 mL) was added, the solvent was evaporated under reduced pressure to give a crude product, which was purified by flash SiO_2 gel column chromatography (PE: EA = 10:1 to 5:1), **24b** (18.9 mg, 65% yield) was obtained as a colorless oil. ^1H NMR (400 MHz, $CDCl_3$) δ 8.20 (dd, *J* = 8.0, 1.0 Hz, 1H), 7.71 (td, *J* = 7.5, 1.0 Hz, 1H), 7.62 (td, *J* = 8.0, 1.0 Hz, 1H), 7.42 – 7.33 (m, 2H), 7.14 – 7.08 (m, 2H), 7.05 (t, *J* = 7.5 Hz, 1H), 5.85 (s, 1H), 2.91 – 2.63 (m, 2H), 1.67 – 1.51 (m, 2H), 1.24 (dtd, *J* = 11.5, 7.5,

4.0 Hz, 2H), 0.78 (t, J = 7.5 Hz, 3H); ^{13}C NMR (101 MHz, $CDCl_3$) δ 153.5, 138.0, 137.6, 134.0, 133.6, 130.8, 130.7, 129.4, 128.8, 128.3, 121.5, 119.6, 53.9, 24.5, 21.5, 13.5; IR (film): 3398 (br), 2960, 2926, 2872, 2864, 1608, 1591, 1467, 1450, 1290, 1275, 1143, 1123, 757 cm^{-1}; ESI-HRMS: m/z calcd for $C_{16}H_{19}O_3S$[M+H]$^+$: 291.1049; found: 291.1045.

Synthesis of *N*-benzyl-2'-hydroxy-[1,1'-biphenyl]-2-sulfonamide 24c

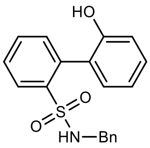

24c

n-BuLi (0.21 mmol) in THF was added dropwise to a solution of $BnNH_2$ (22 μL, 0.20 mmol) in THF (1.0 mL) at 0 °C. The solution was stirred at same temperature for 5 min, **13a** (21.6 mg, 0.10 mmol) was added, the resulting mixture was stirred at 0 °C for 3 h, saturated NH_4Cl (0.30 mL) was added, the solvent was evaporated under reduced pressure to give a crude product, which was purified by flash SiO_2 gel column chromatography (PE: EA = 3:1 to 1:1), **24c** (28.8 mg, 85% yield) was obtained as a colorless oil. ^{1}H NMR (400 MHz, $CDCl_3$) δ 8.20 (dd, J = 8.0, 1.0 Hz, 1H), 7.66 (td, J = 7.5, 1.5 Hz, 1H), 7.58 (td, J = 7.5, 1.5 Hz, 1H), 7.33 (dd, J = 7.5, 1.0 Hz, 1H), 7.31 – 7.21 (m, 4H), 7.10 (dd, J = 6.5, 3.0 Hz, 2H), 7.00 (d, J = 8.5 Hz, 1H), 6.96 – 6.88 (m, 2H), 5.46 (br s, 1H), 4.11 – 3.95 (m, 2H), 3.90 – 3.86 (m, 1H); ^{13}C NMR (101 MHz, $CDCl_3$) δ 153.2, 139.0, 136.2, 136.1, 133.2, 133.1, 130.6, 130.0, 129.5, 128.8, 128.7, 128.1, 127.2, 121.2, 118.4, 47.4; IR (film): 3358 (br), 3005, 2934, 1608, 1590, 1448, 1325, 1158, 752 cm^{-1}; ESI-HRMS (m/z): [M+Na]$^+$ calcd for $C_{19}H_{17}NNaO_3S$: 362.0821; found: 362.0816.

Synthesis of *N*-allyl-2'-hydroxy-[1,1'-biphenyl]-2-sulfonamide (24d)

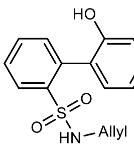

24d

n-BuLi (0.21 mmol) in THF was added dropwise to a solution of allylamine (15 μL, 0.20 mmol) in THF (1.0 mL) at 0 °C. The solution was stirred at same temperature for 5 min, **13a** (21.6 mg, 0.10 mmol) was added, the resulting mixture was stirred at 0 °C for 3 h, saturated NH_4Cl (0.30 mL) was added, the solvent was evaporated under reduced pressure to give a crude product, which was purified by flash SiO_2 gel column chromatography (PE: EA = 3:1 to 1:1), **24d** (28.1 mg, 97% yield) was obtained as a colorless oil. ^{1}H NMR (400 MHz, $CDCl_3$) δ 8.16 (d, J = 7.5 Hz, 1H), 7.66 (t, J = 7.0 Hz, 1H), 7.57 (t, J = 7.5 Hz, 1H), 7.34 (dd, J = 12.0, 4.5 Hz, 2H), 7.15 (dd, J = 7.5, 1.0 Hz, 1H), 7.05 – 7.00 (m, 2H), 5.64 (ddd, J = 13.0, 8.0, 3.5 Hz, 1H), 5.50 (brs, 1H), 5.08 (t, J = 13.0 Hz, 2H), 3.89 (br,

s, 1H), 3.45 (dd, *J* = 14.5, 6.0 Hz, 1H), 3.33 (dd, *J* = 14.5, 6.0 Hz, 1H); ^{13}C NMR (101 MHz, $CDCl_3$) δ 153.2, 138.9, 136.1, 133.20, 133.15, 132.7, 130.7, 130.3, 129.6, 128.8, 127.2, 121.3, 118.5, 118.1, 45.9; IR (film): 3553 (br), 3005, 2989, 1459, 1448, 1275, 1261, 1159, 764, 750 cm^{-1}; ESI-HRMS (m/z): $[M+Na]^+$ calcd for $C_{15}H_{15}NNaO_3S$: 312.0665; found: 312.0666.

Synthesis of 2'-(piperidin-1-ylsulfonyl)-[1,1'-biphenyl]-2-ol (24e)

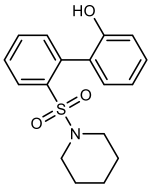

24e

n-BuLi (0.21 mmol) in THF was added dropwise to a solution of piperidine (20 μL, 0.20 mmol) in THF (1.0 mL) at 0 °C. The solution was stirred at same temperature for 5 min, **13a** (21.6 mg, 0.10 mmol) was added, the resulting mixture was stirred at 0 °C for 3 h, saturated NH_4Cl (0.30 mL) was added, the solvent was evaporated under reduced pressure to give a crude product, which was purified by flash SiO_2 gel column chromatography (PE: EA = 3:1 to 1:1), **24e** (30.5 mg, 96% yield) was obtained as a colorless oil. ^{1}H NMR (400 MHz, $CDCl_3$) δ 8.15 (d, *J* = 8.0 Hz, 1H), 7.61 (t, *J* = 7.5 Hz, 1H), 7.52 (t, *J* = 7.5 Hz, 1H), 7.35 – 7.22 (m, 2H), 7.11 (d, *J* = 8.0 Hz, 1H), 7.04 – 6.98 (m, 2H), 5.64 (s, 1H), 2.75 (q, *J* = 12.0 Hz, 4H), 1.40 (br s, 6H); ^{13}C NMR (101 MHz, $CDCl_3$) δ 153.1, 138.6, 137.1, 133.9, 133.0, 131.2, 130.4, 130.0, 128.6, 128.4, 120.8, 118.4, 45.6, 25.4, 23.8; IR (film): 3416 (br), 2926, 2855, 1450, 1276, 1261, 1149, 764, 750 cm^{-1}; ESI-HRMS (m/z): $[M+H]^+$ calcd for $C_{17}H_{20}NO_3S$: 318.1158; found: 318.1156.

Synthesis of 2'-(pyrrolidin-1-ylsulfonyl)-[1,1'-biphenyl]-2-ol (24f)

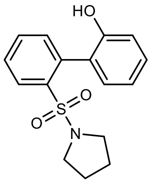

24f

n-BuLi (0.21 mmol) in THF was added dropwise to a solution of pyrrolidine (17 μL, 0.20 mmol) in THF (1.0 mL) at 0 °C. The solution was stirred at same temperature for 5 min, **13a** (21.6 mg, 0.10 mmol) was added, the resulting mixture was stirred at 0 °C for 3 h, saturated NH_4Cl (0.30 mL) was added, the solvent was evaporated under reduced pressure to give a crude product, which was purified by flash SiO_2 gel column chromatography (PE: EA = 3:1 to 1:1), **24f** (30.0 mg, 99% yield) was obtained as a colorless oil. ^{1}H NMR (400 MHz, $CDCl_3$) δ 8.16 (d, *J* = 8.0 Hz, 1H), 7.60 (t, *J* = 7.5 Hz, 1H), 7.52 (t, *J* = 7.5 Hz, 1H), 7.37 – 7.22 (m, 2H), 7.09 – 7.03 (m, 2H), 6.99 (t, *J* = 7.5 Hz, 1H), 5.79 (s, 1H), 2.86 (d, *J* = 6.0 Hz, 2H), 2.72 (d, *J* = 6.0 Hz, 2H), 1.66 (br s, 4H); ^{13}C NMR (101

MHz, $CDCl_3$) δ 153.2, 139.2, 137.0, 133.6, 133.0, 130.7, 130.2, 130.0, 129.0, 128.5, 120.9, 118.7, 46.9, 25.8; IR (film): 3414 (br), 3006, 2989, 1469, 1463, 1275, 1261, 764, 750 cm^{-1}; ESI-HRMS (m/z): $[M+H]^+$ calcd for$C_{16}H_{18}NO_3S$: 304.1002; found: 304.0999.

Synthesis of [1,1'-biphenyl]-2,2'-diol (25a)

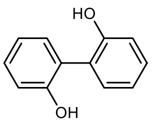

25a

25a was prepared according to a published procedure. The spectroscopic properties of **25a** were consistent with the data available in the literature.[21]

Synthesis of 5,5'-dimethyl-[1,1':2',1''-terphenyl]-2-ol (25b)

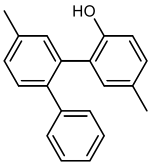

25b

A mixture of iodobenzene (14 μL, 0.12 mmol), **15e** (19.8 mg, 0.10 mmol), $Pd(OAc)_2$ (2.2 mg, 10μmol), Cs_2CO_3 (39.0 mg, 0.12 mmol), and DMF (0.50 mL) was stirred under nitrogen at 100 °C for 24 h. After cooling, to the reaction mixture was added HCl (aq) (1.0 M, 0.50 mL), the solvent was evaporated under reduced pressure to give a crude product, which was purified by flash SiO_2 gel column chromatography (PE: EA = 15:1 to 8:1), **25b** (17.1 mg, 62% yield) was obtained as a colorless oil. ^1H NMR (400 MHz, $CDCl_3$) δ 7.40 (d, *J* = 8.0 Hz, 1H), 7.29 (d, *J* = 8.0 Hz, 1H), 7.23 – 7.16 (m, 6H), 6.96 (dd, *J* = 8.0, 1.5 Hz, 1H), 6.89 (s, 1H), 6.67 (d, *J* = 8.0 Hz, 1H), 4.62 (s, 1H), 2.44 (s, 3H), 2.22 (s, 3H); ^{13}C NMR (101 MHz, $CDCl_3$) δ 150.2, 140.5, 138.7, 138.0, 135.2, 132.2, 131.5, 130.7, 129.7, 129.6, 129.5, 129.2, 128.2, 127.8, 127.0, 115.4, 21.2, 20.6; IR (film): 3539 (br), 3437, 3034, 2926, 1497, 1276, 1182, 764, 750 cm^{-1}; APCI-HRMS (m/z): $[M+H]^+$ calcd for $C_{20}H_{19}O$: 275.1430; found: 275.1425.

Synthesis of (*E*)-ethyl 3-(2'-hydroxy-5,5'-dimethyl-[1,1'-biphenyl]-2-yl)acrylate (25c)

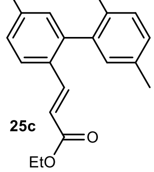

25c

15e (19.8 mg, 0.10 mmol), ethyl acrylate (22 μL, 0.20 mmol), $Pd(OAc)_2$ (2.2 mg, 10 μmol), 1,4-benzoquinone (10.8 mg, 0.10 mmol), and AcOH (0.20 mL)were added to a sealed tube with a

Teflon-lined cap. The mixture was heated at 80 °C (oil bath temperature) for 24 h and then cooled to room temperature. The volatiles were removed under reduced pressure, and the residue was purified by flash SiO_2 gel column chromatography (PE:EA = 10:1 to 8:1), **25c** (14.2 mg, 48% yield) was obtained as a colorless oil. ^1H NMR (400 MHz, $CDCl_3$) δ 7.67 (d, *J* = 8.0 Hz, 1H), 7.53 (d, *J* = 16.0 Hz, 1H), 7.24 (d, *J* = 7.5 Hz, 1H), 7.15 (s, 1H), 7.09 (dd, *J* = 8.0, 2.0 Hz, 1H), 6.89 (d, *J* = 2.0 Hz, 1H), 6.86 (d, *J* = 8.0 Hz, 1H), 6.35 (d, *J* = 16.0 Hz, 1H), 4.67 (s, 1H), 4.17 (q, *J* = 7.0 Hz, 2H), 2.40 (s, 3H), 2.30 (s, 3H), 1.26 (t, *J* = 7.0 Hz, 3H); ^{13}C NMR (101 MHz, $CDCl_3$) δ 167.1, 150.5, 142.6, 141.0, 137.9, 132.0, 131.4, 131.0, 130.4, 130.0, 129.6, 126.9, 125.9, 118.7, 115.7, 60.5, 21.5, 20.6, 14.4; IR (film): 3411 (br), 2926, 1688, 1631, 1605, 1511, 1316, 1180, 1035, 816 cm^{-1}; ESI-HRMS (m/z): $[M+H]^+$ calcd for $C_{19}H_{21}O_3$: 297.1485; found: 297.1487.

Computational Method

All the theoretical calculations were carried out with the density-functional theory (DFT)[22] methods in Gaussian 09 package.[23] Both the geometry optimization of ground state and transition state were performed at the B3LYP[24] method with SDD basis set[25] for Fe atom and 6-31G (d, p) basis set[26] for remaining atoms in the PCM solvent continuum models[27](Ethanol). The vibration frequency calculations were carried out at the same computational method and basis set to obtain the Gibbs free energy, as well as make sure that the optimized structures were true energy minima and transition states. Meanwhile, the IRC calculations were also employed for the optimized transition state to ensure the structure veracity of the reactants, transition states and products.

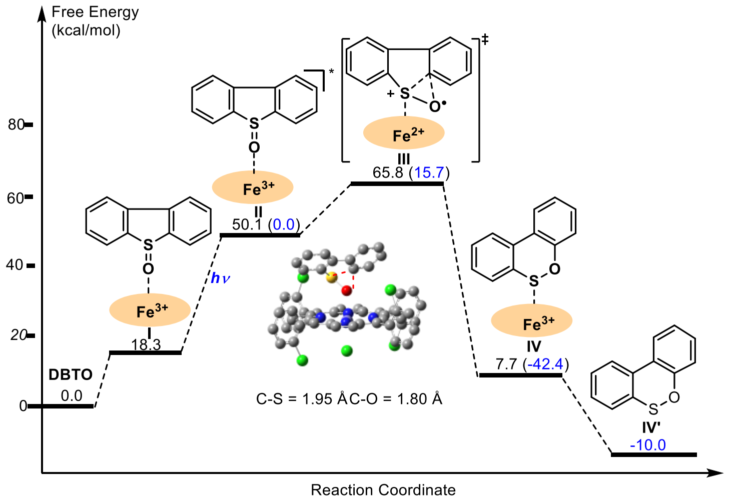

Supplementary Figure 72. Computational studies.

Supplementary Table 2. The energy for molecules in the Supplementary Figure 72.

Molecules	Energy (Hartree)	Relative Energy (kcal/mol)
DBTO<br>Fe(III)	-935.343299<br>-4334.685603	0.00
I	-5269.999726	18.31
II	-5269.949077	50.09 (0.00)
III	-5269.924071	65.78 (15.69)
IV	-5270.016611	7.71 (-42.38)
IV'	-935.359302	-10.04
DBTO-TS-IV	-935.268345	47.03

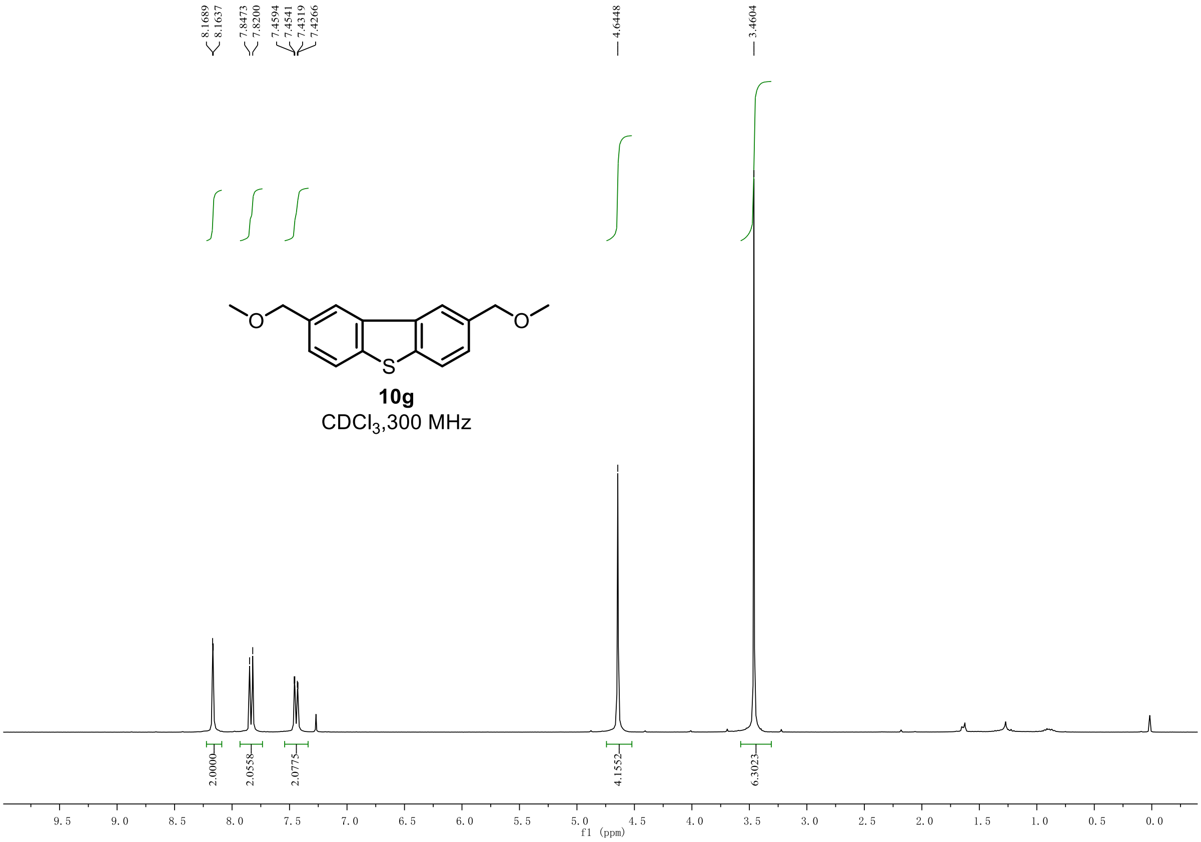

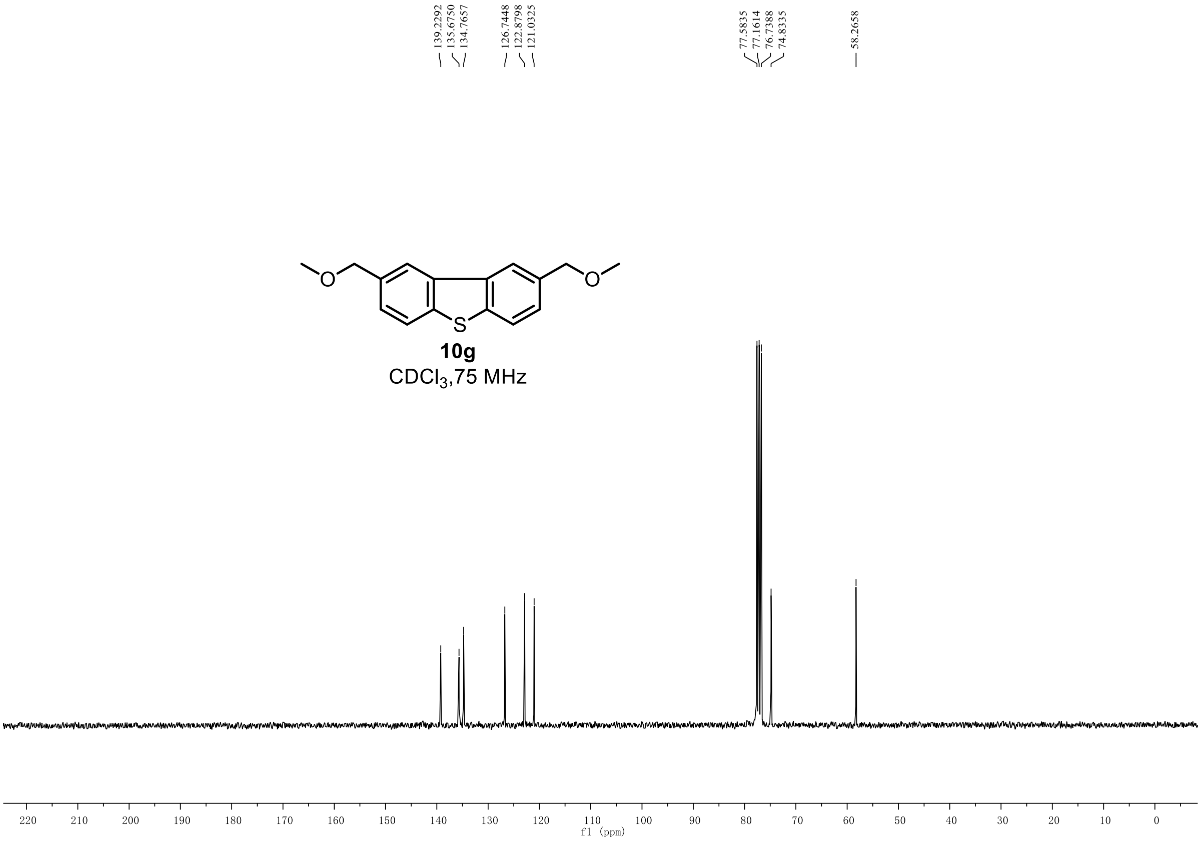

Supplementary Figure 73. NMR spectra of 10g.

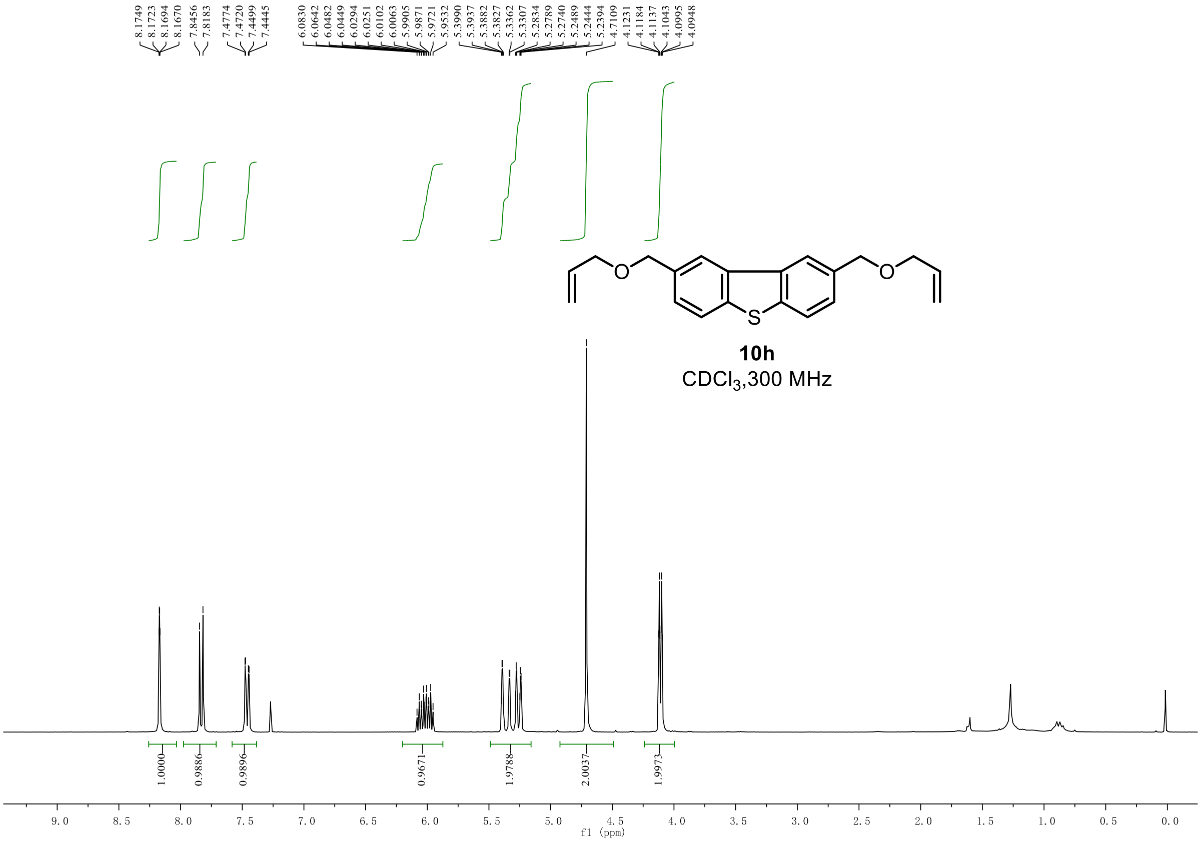

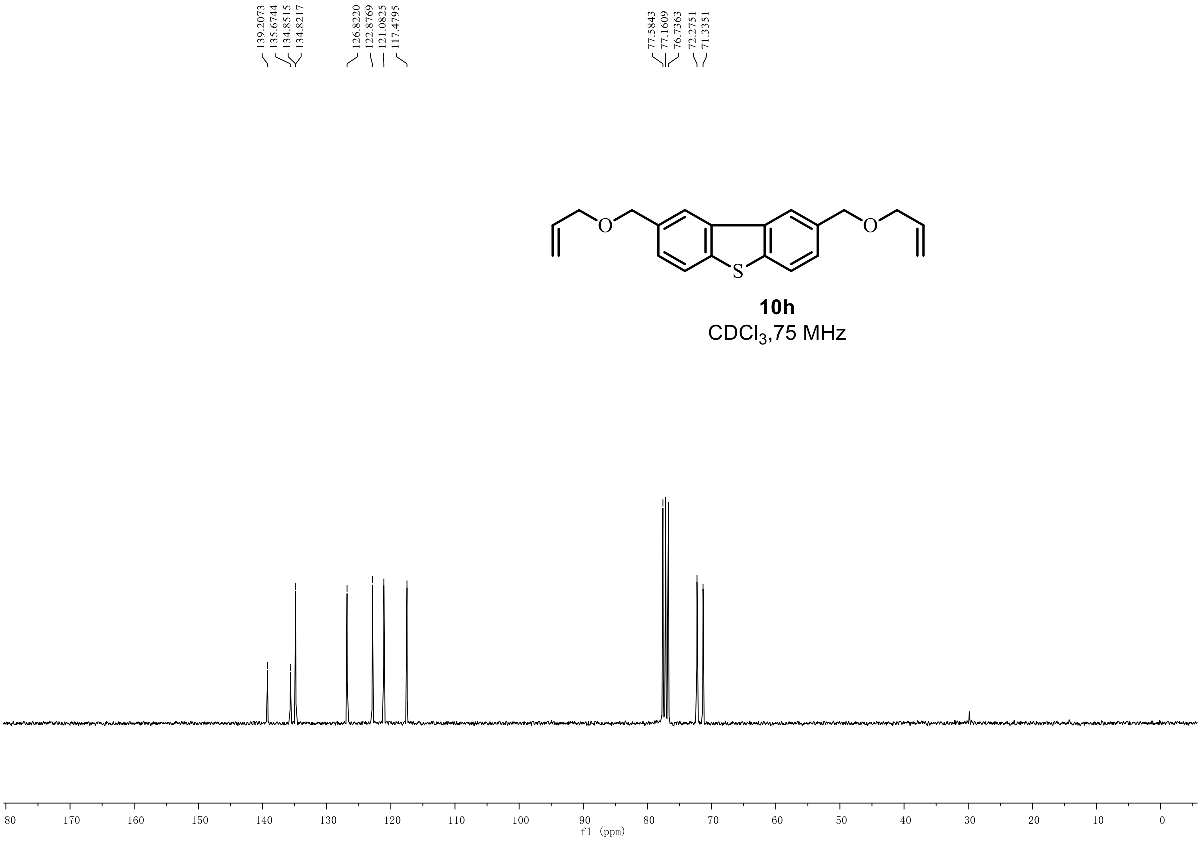

Supplementary Figure 74. NMR spectra of 10h.

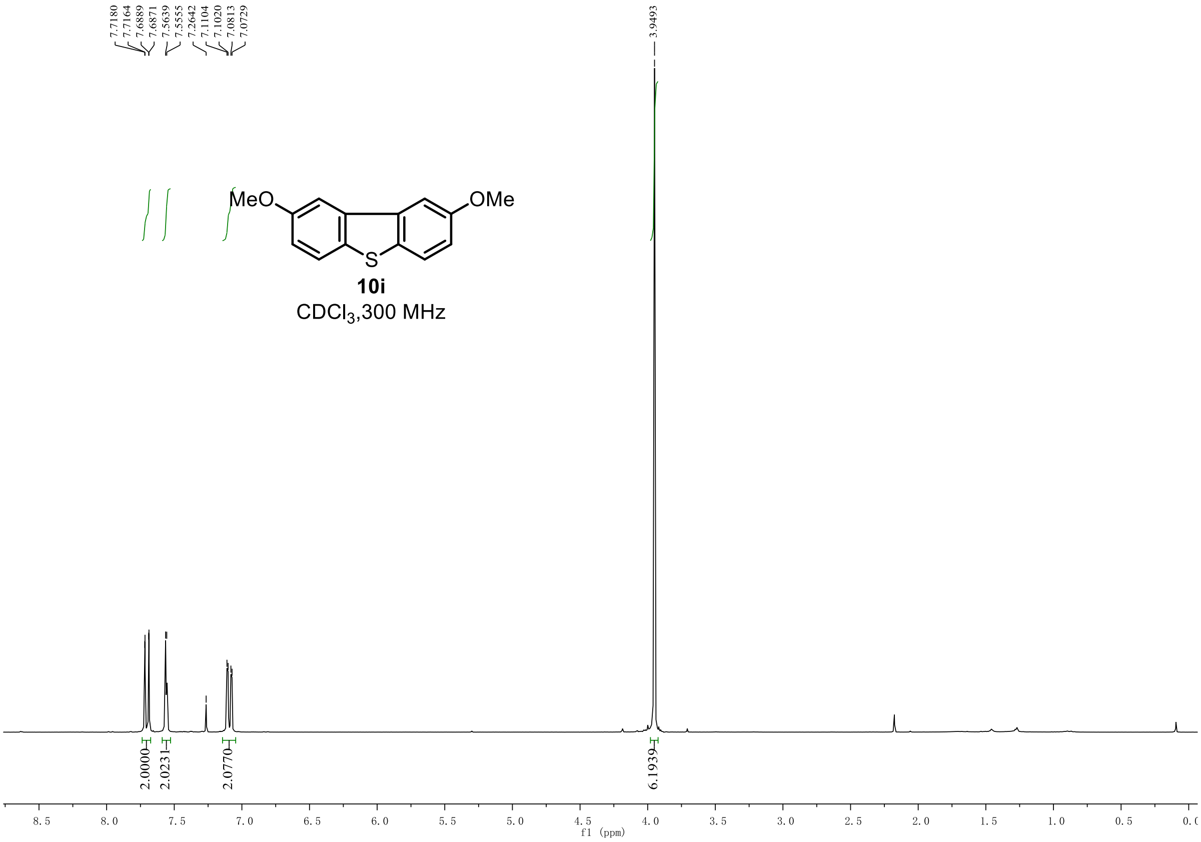

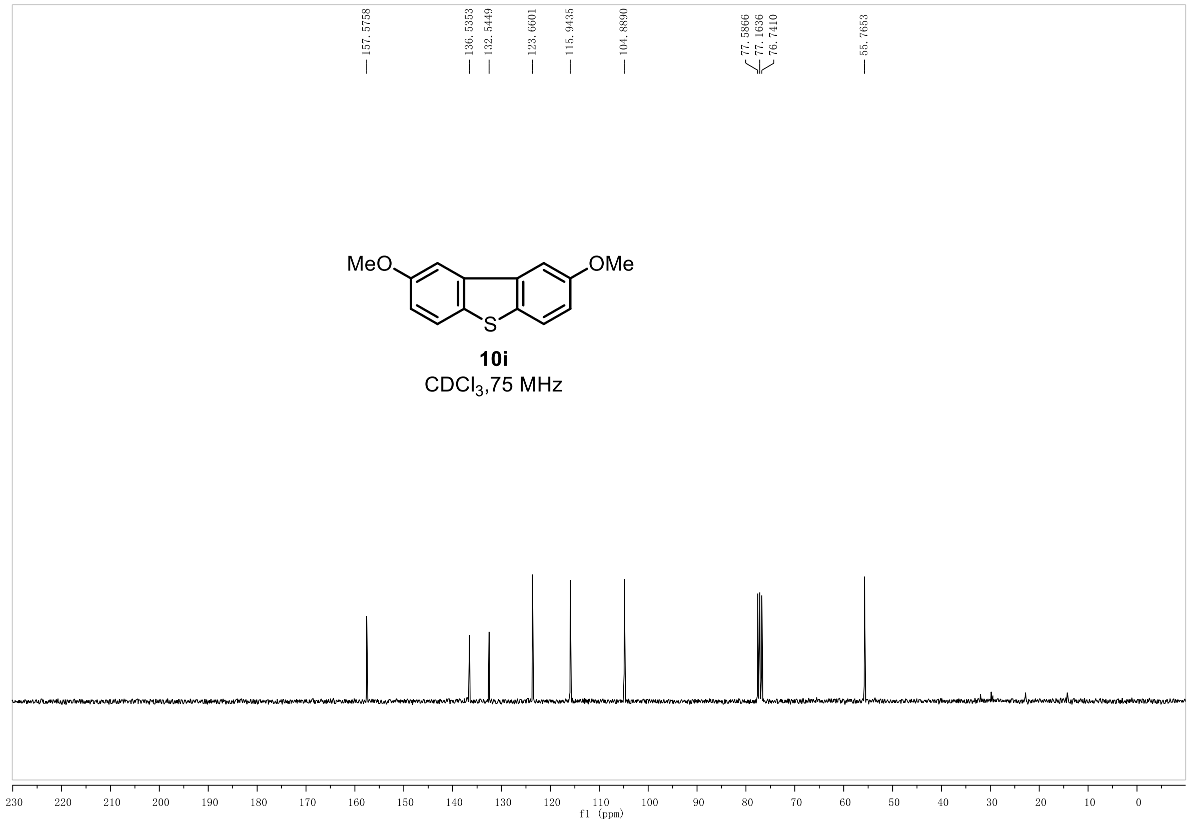

Supplementary Figure 75. NMR spectra of 10i.

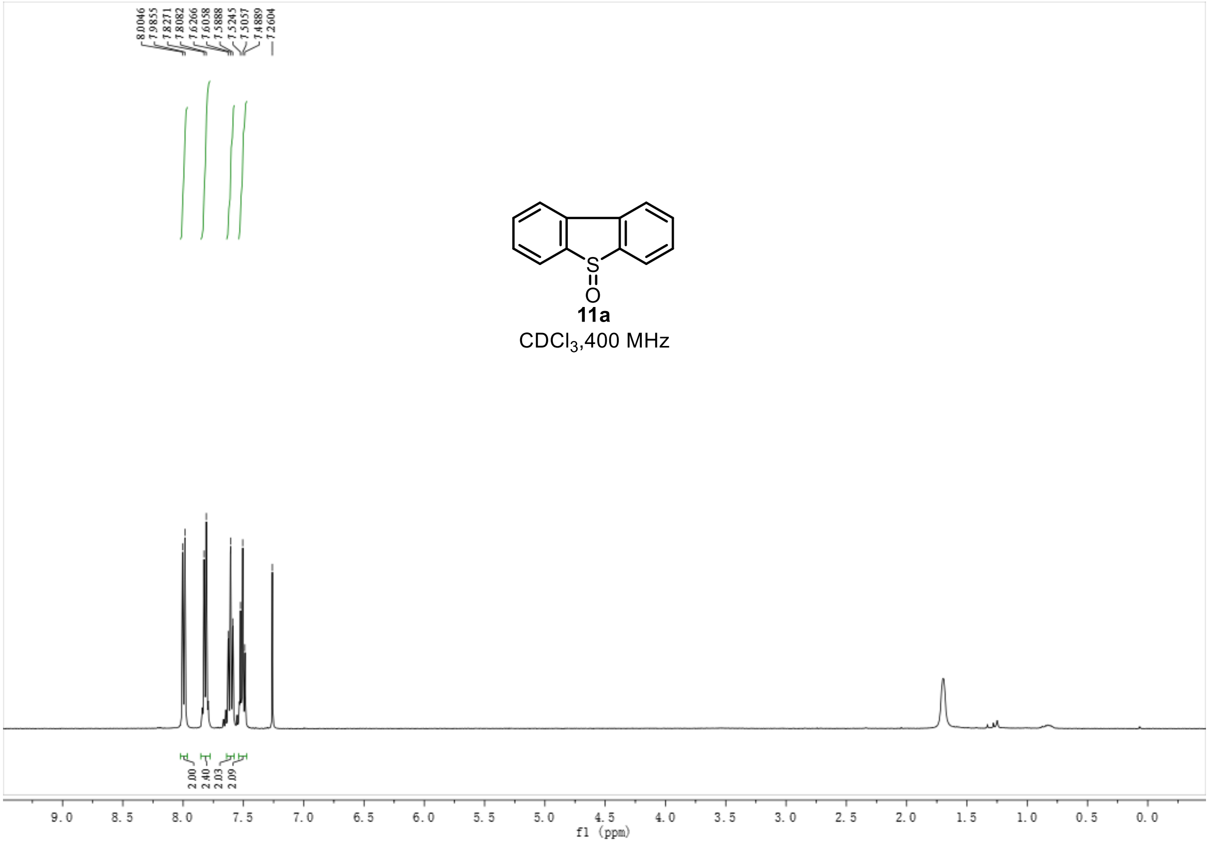

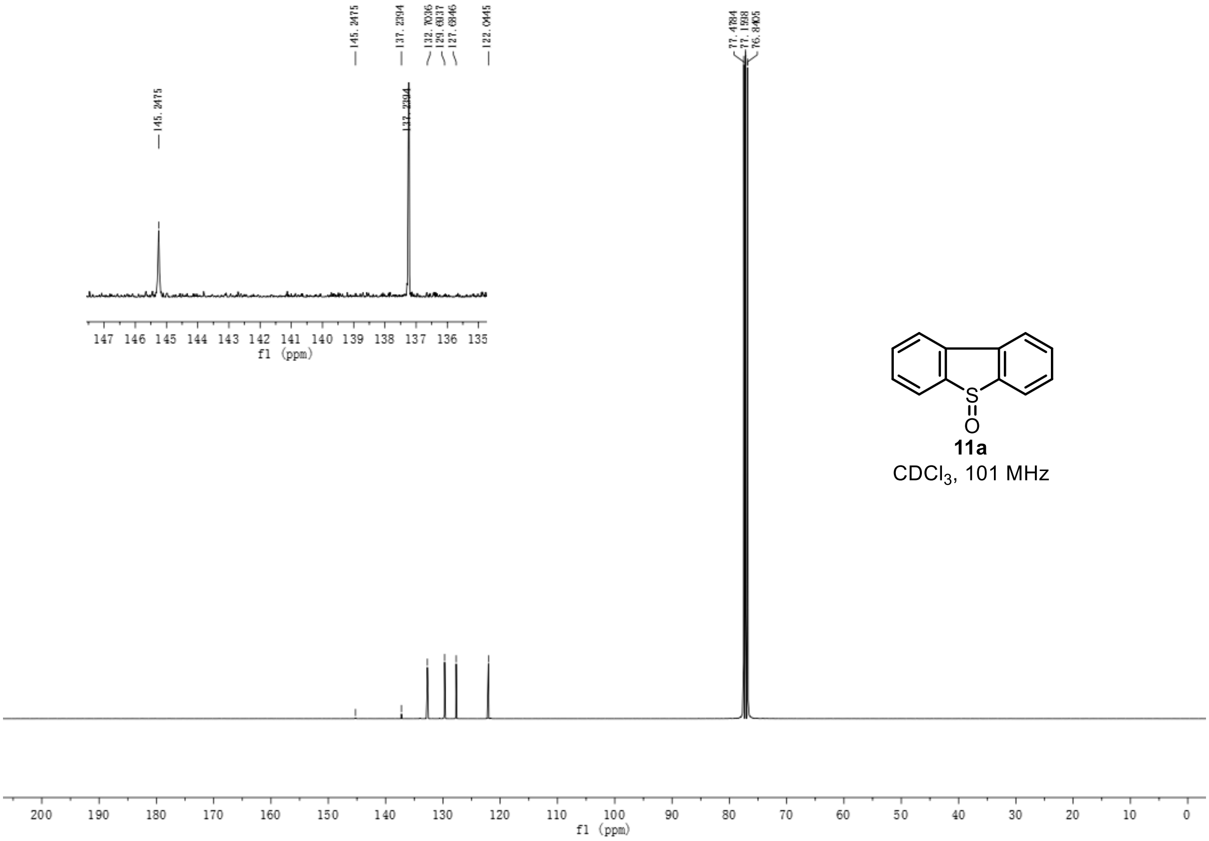

Supplementary Figure 76. NMR spectra of 11a.

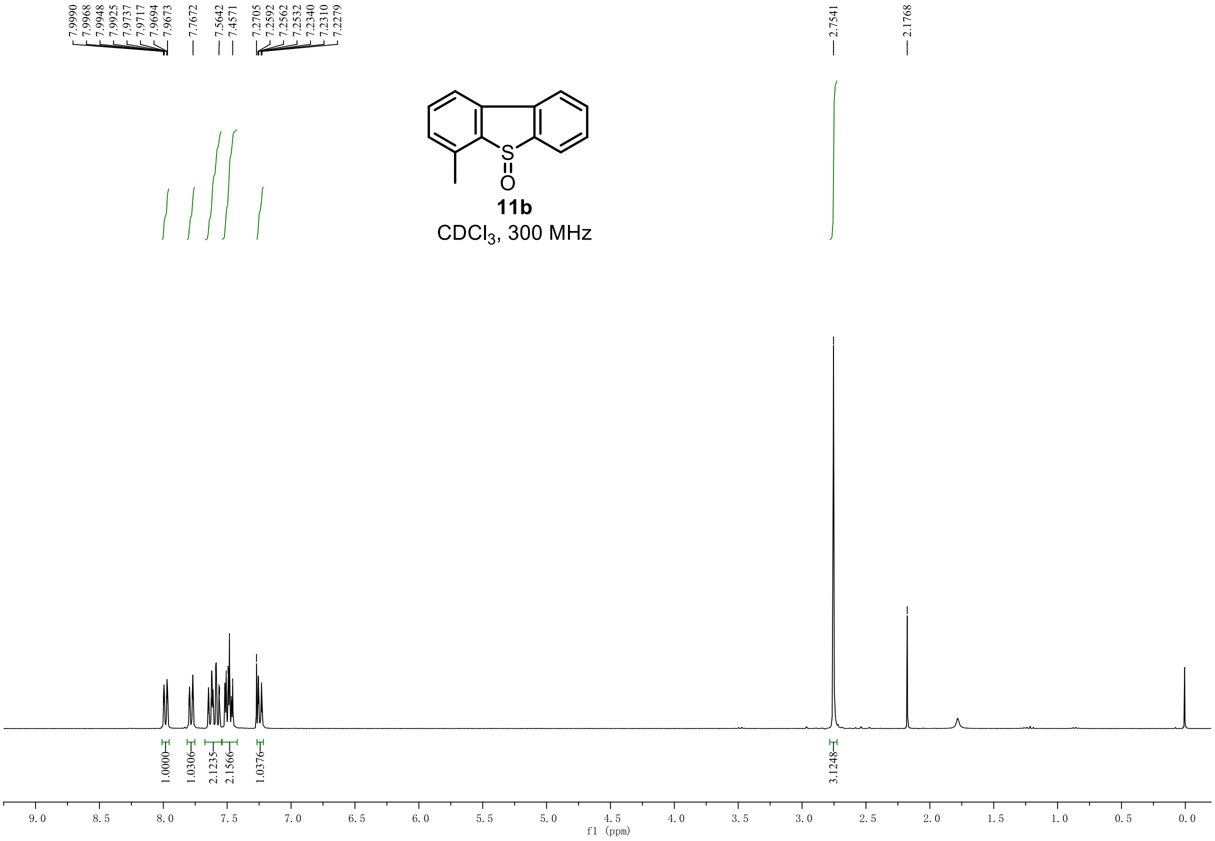

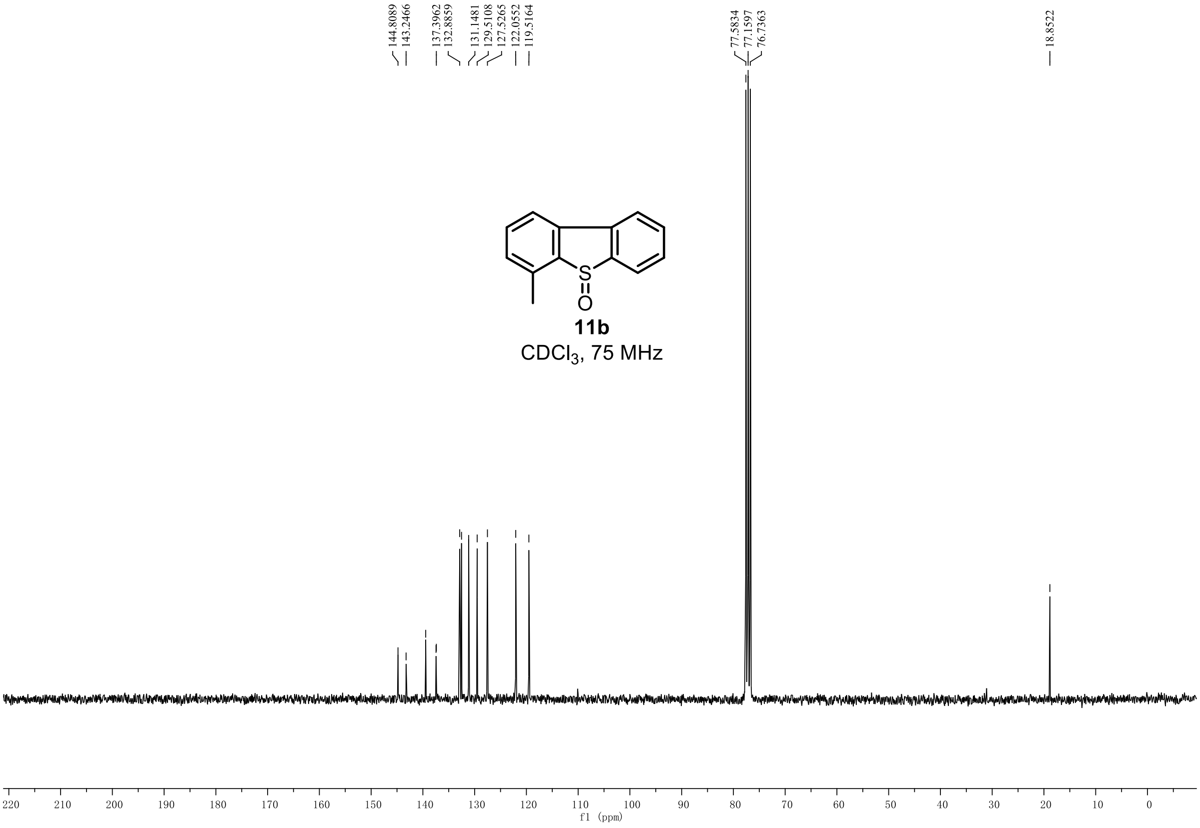

Supplementary Figure 77. NMR spectra of 11b.

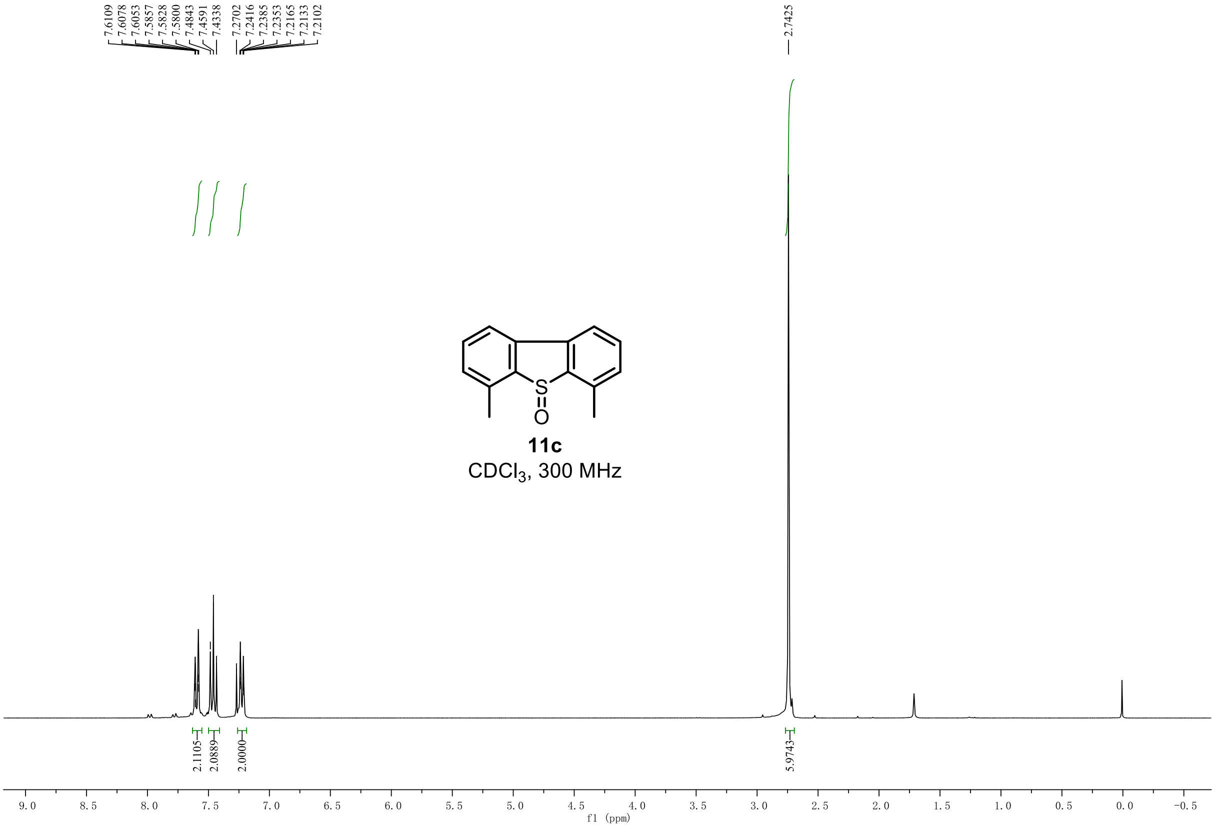

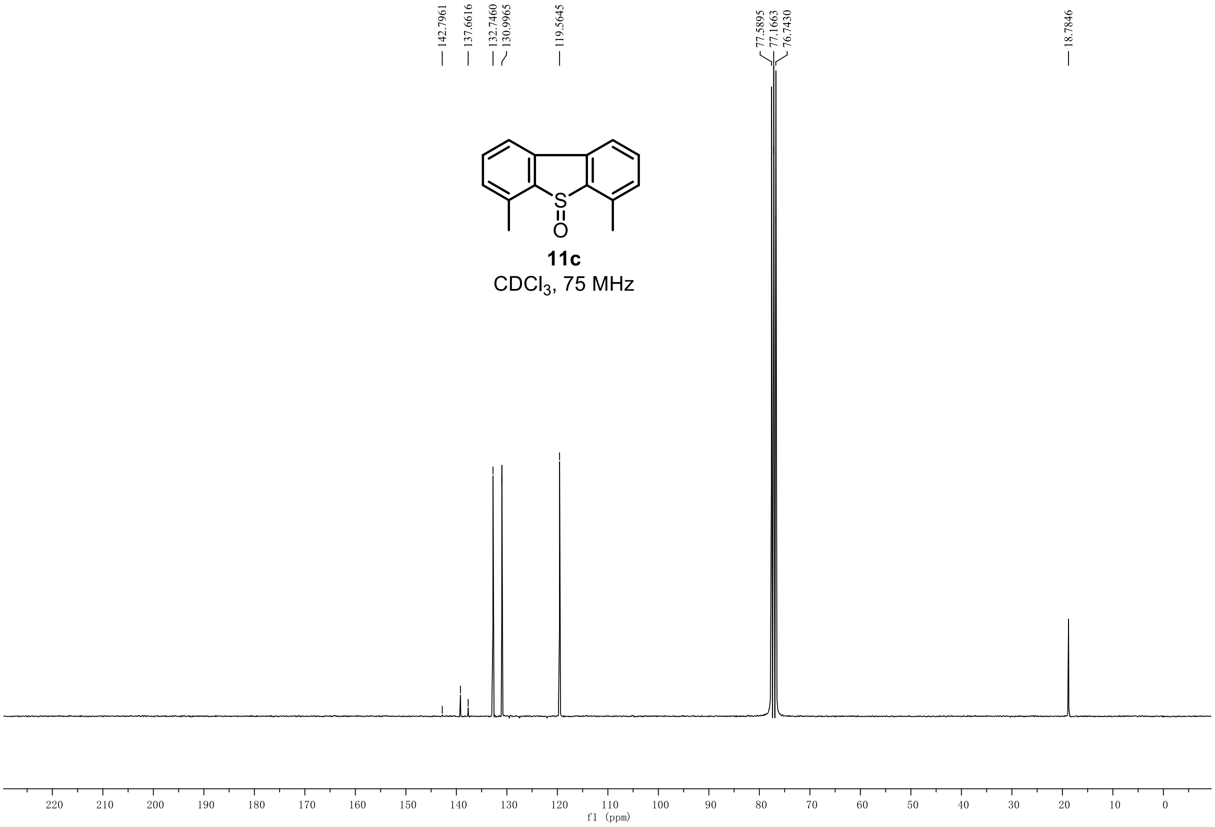

Supplementary Figure 78. NMR spectra of 11c.

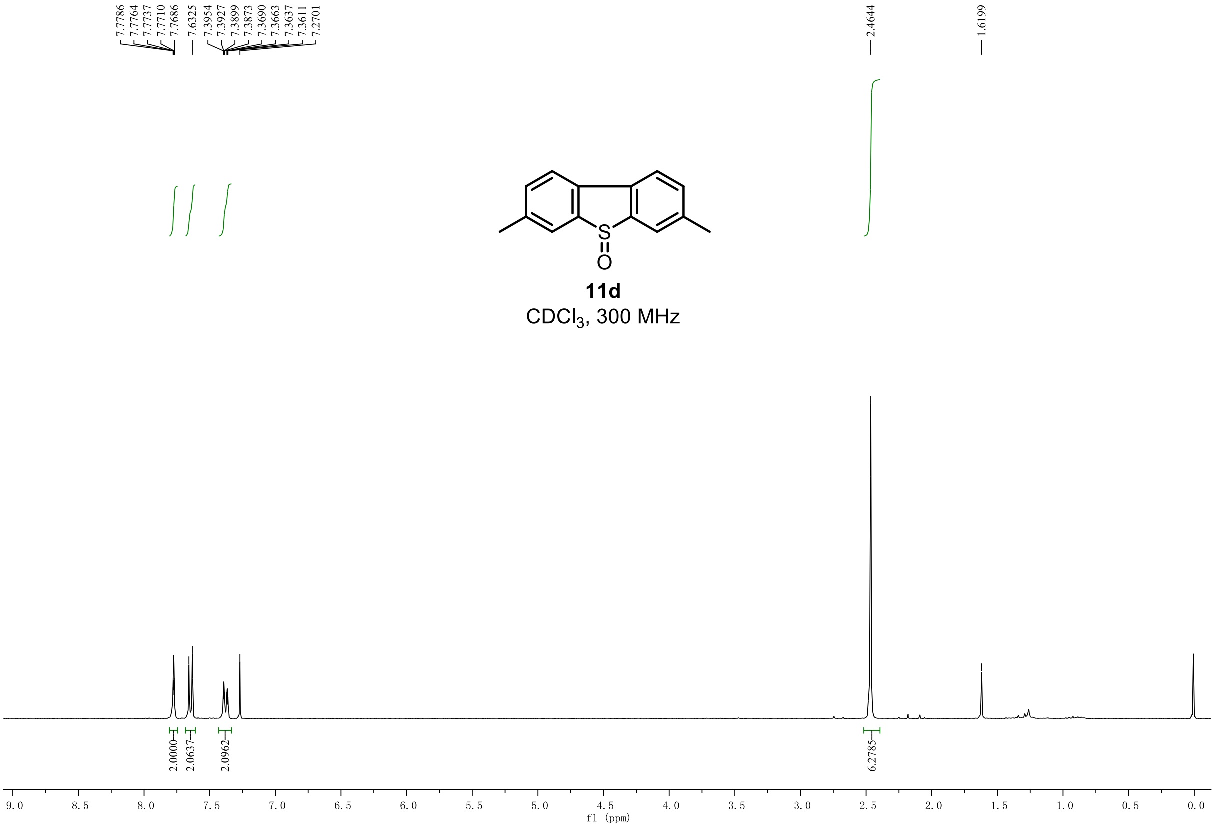

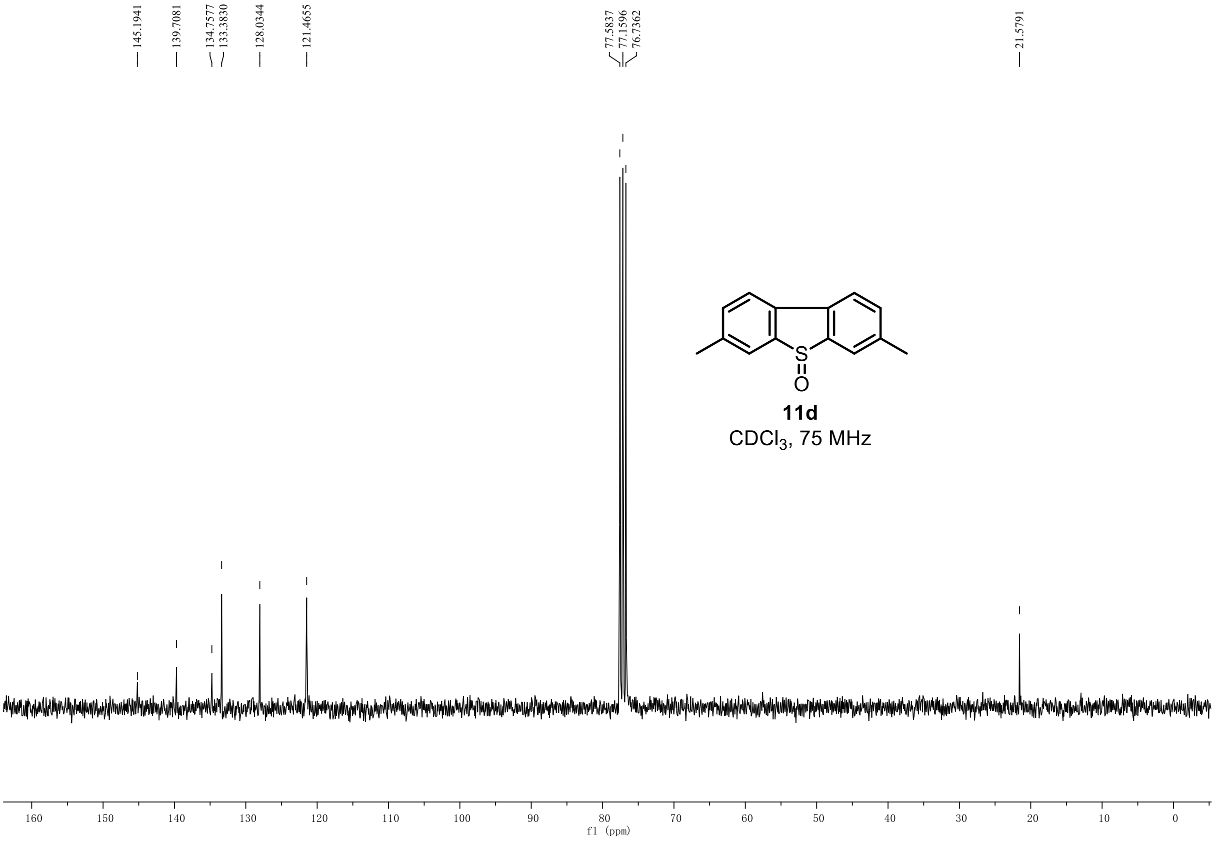

Supplementary Figure 79. NMR spectra of 11d.

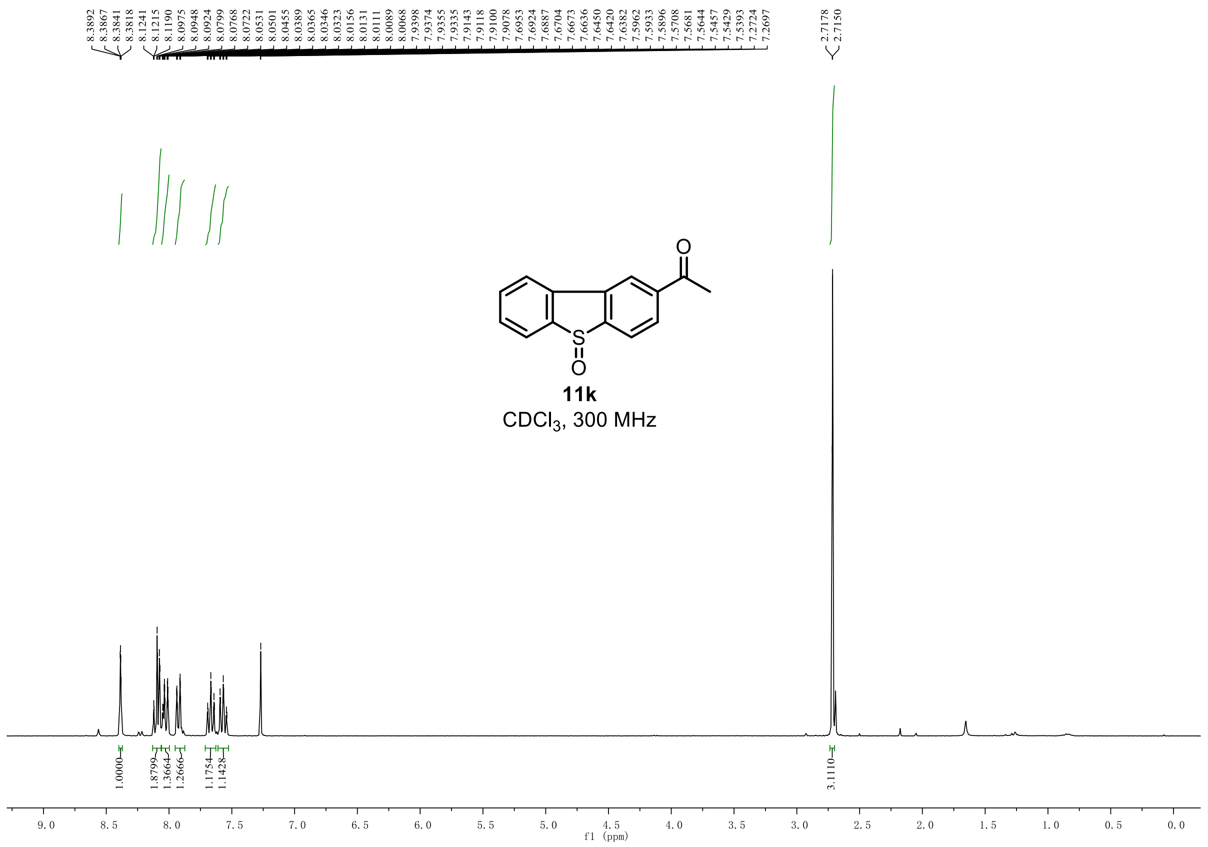

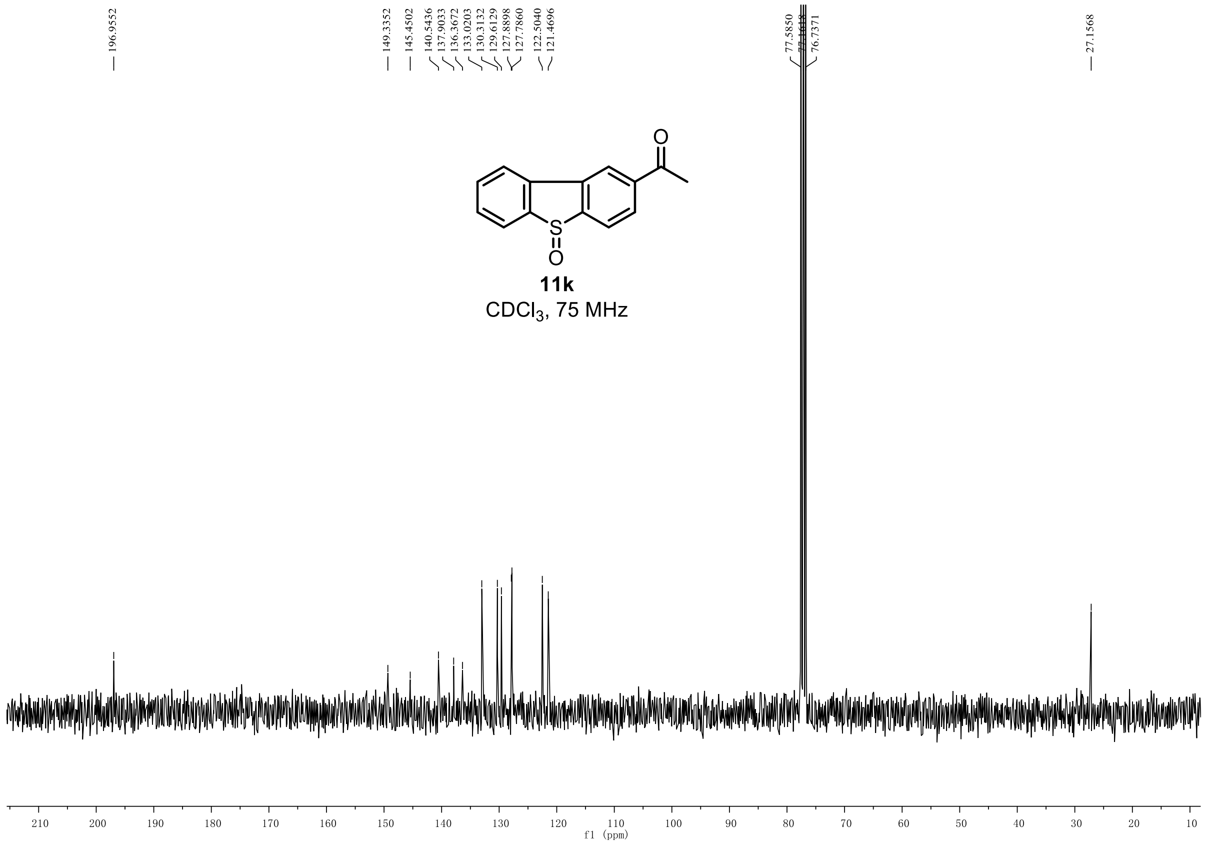

Supplementary Figure 80. NMR spectra of 11k.

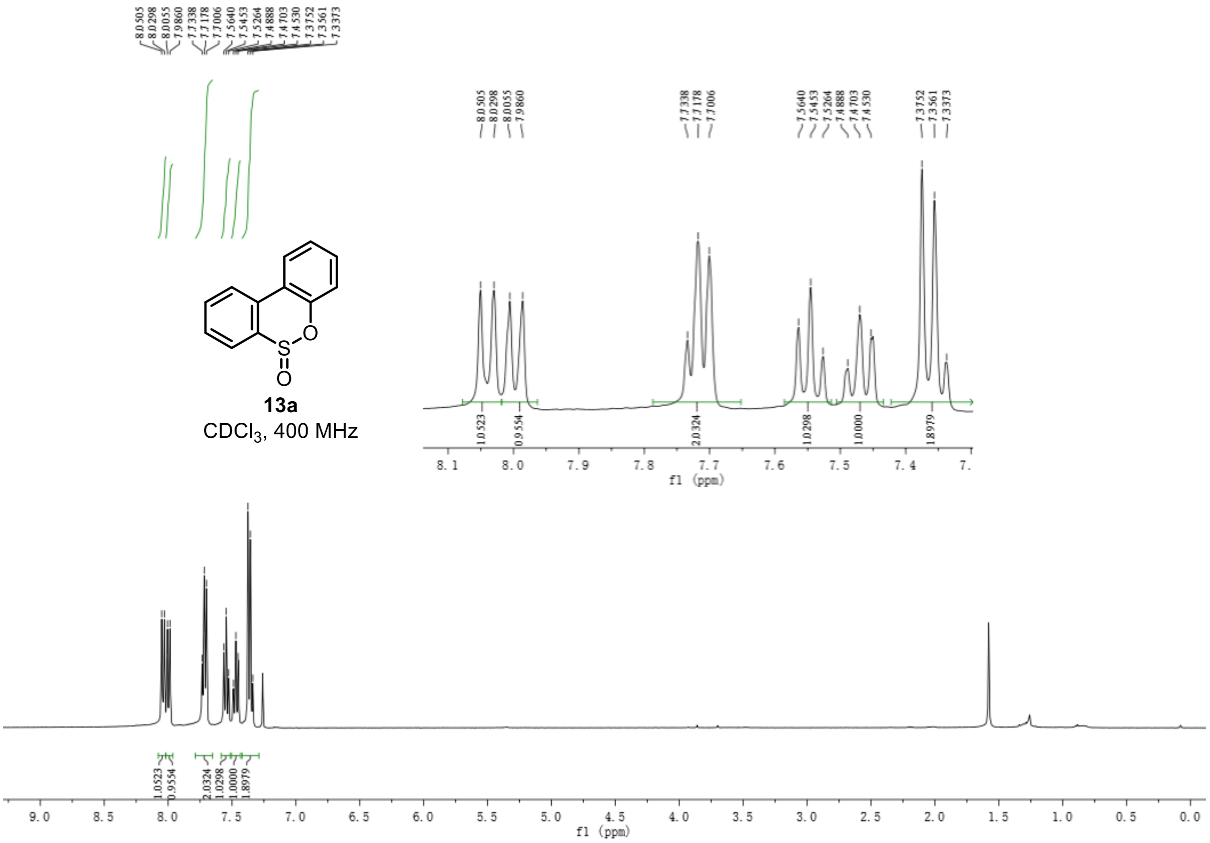

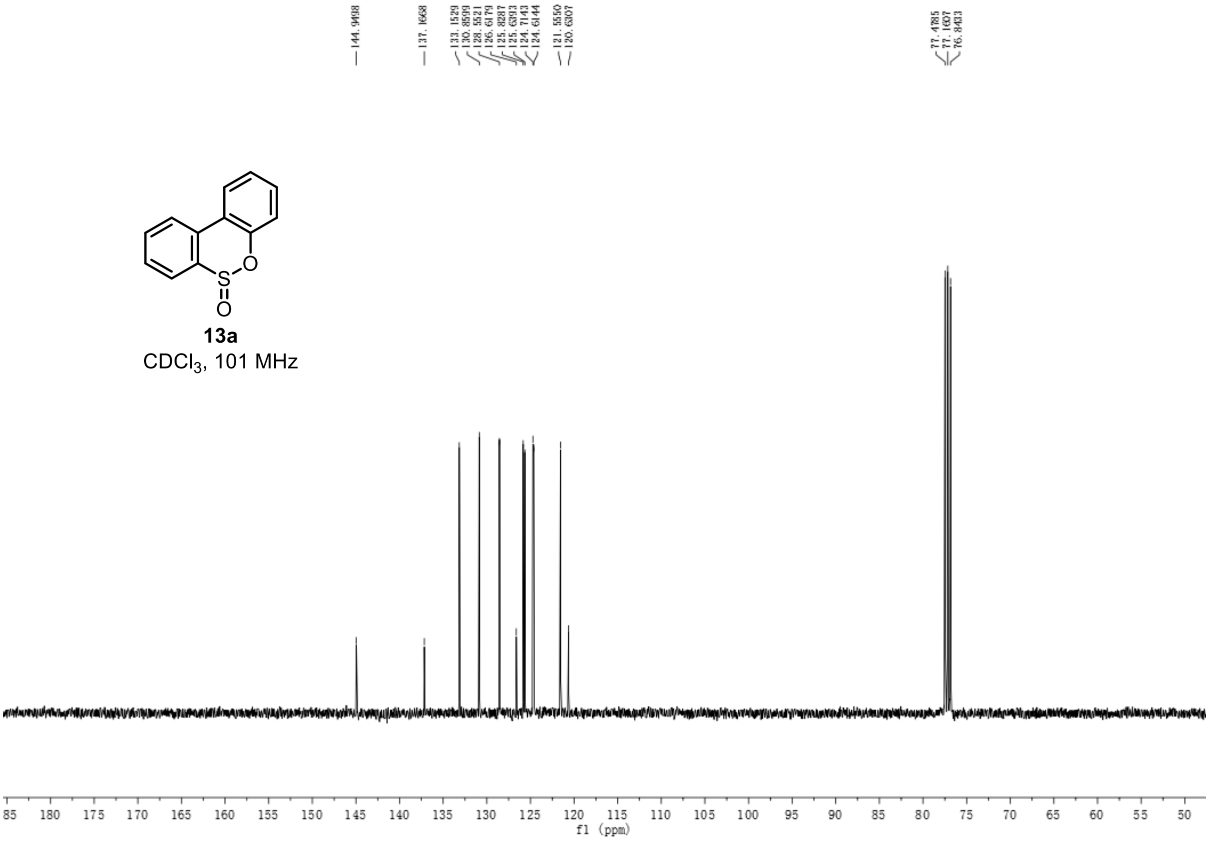

Supplementary Figure 81. NMR spectra of 13a.

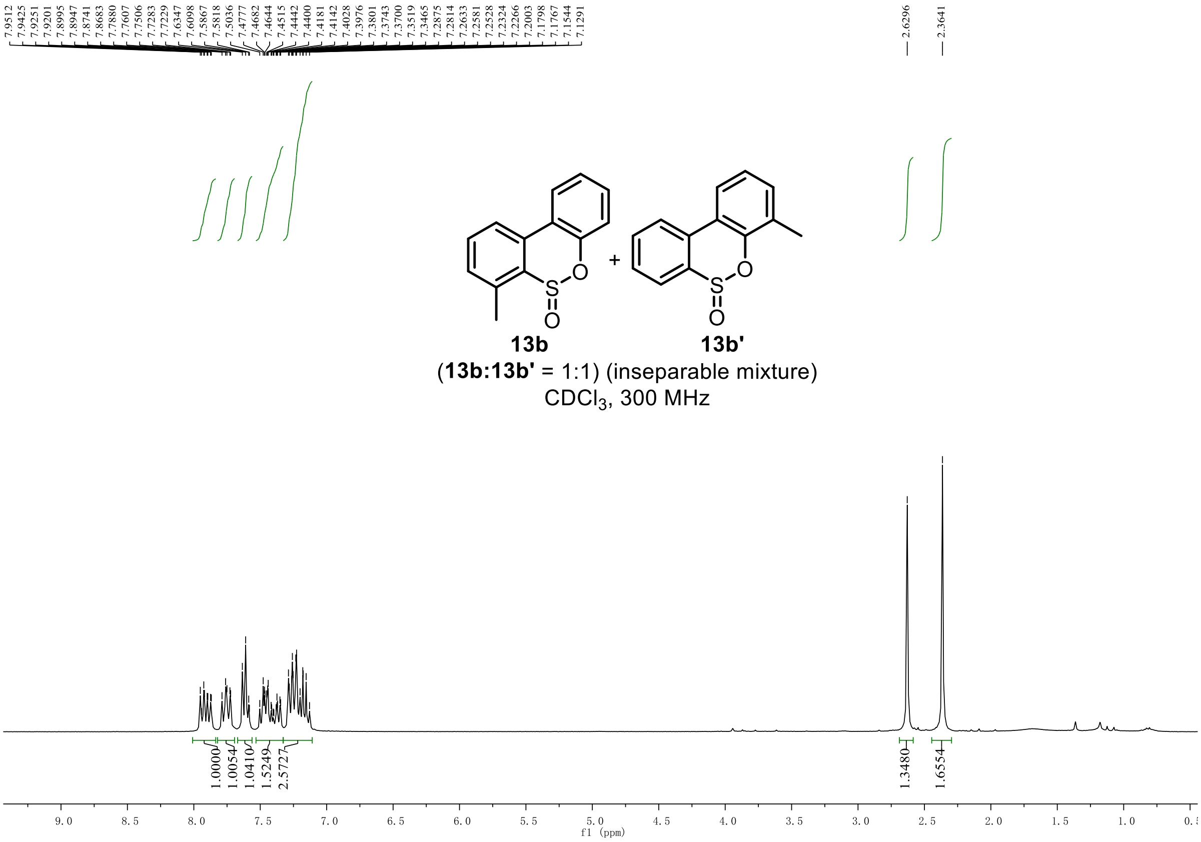

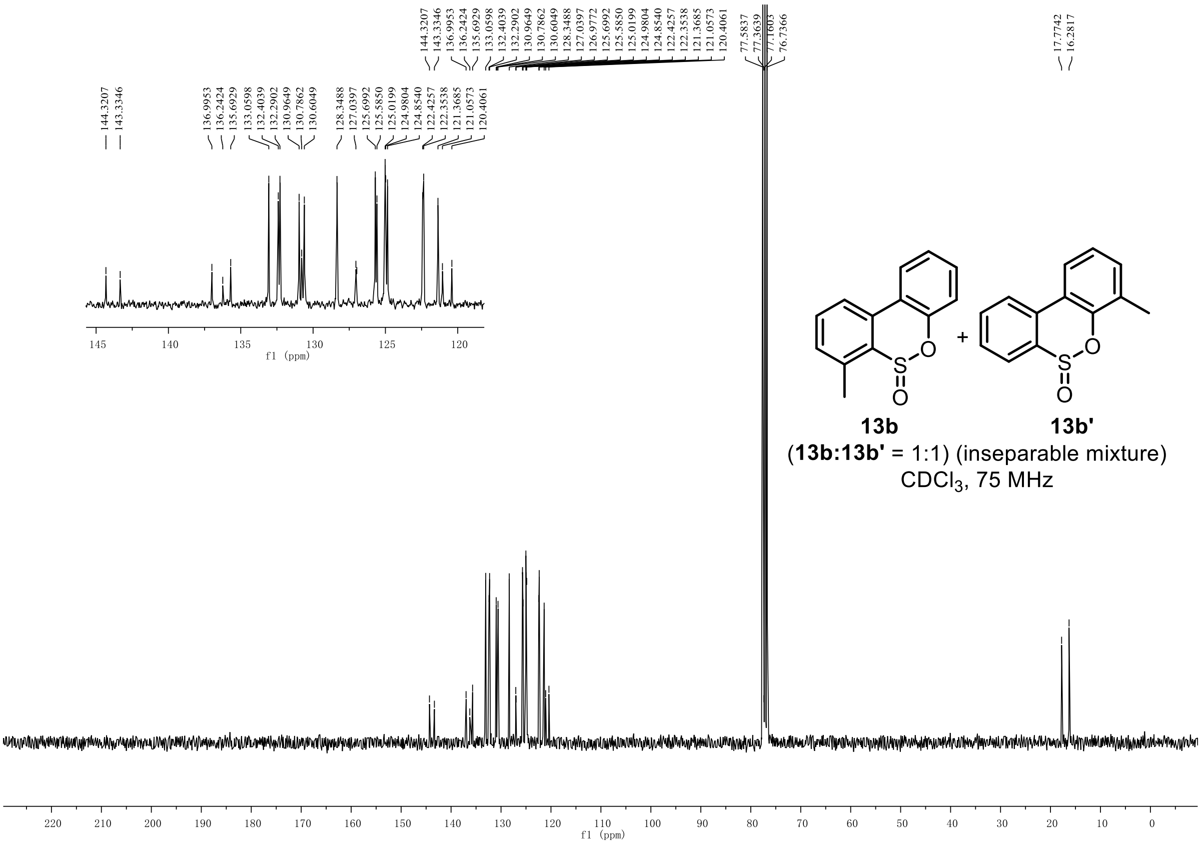

Supplementary Figure 82. NMR spectra of 13b and 13b'.

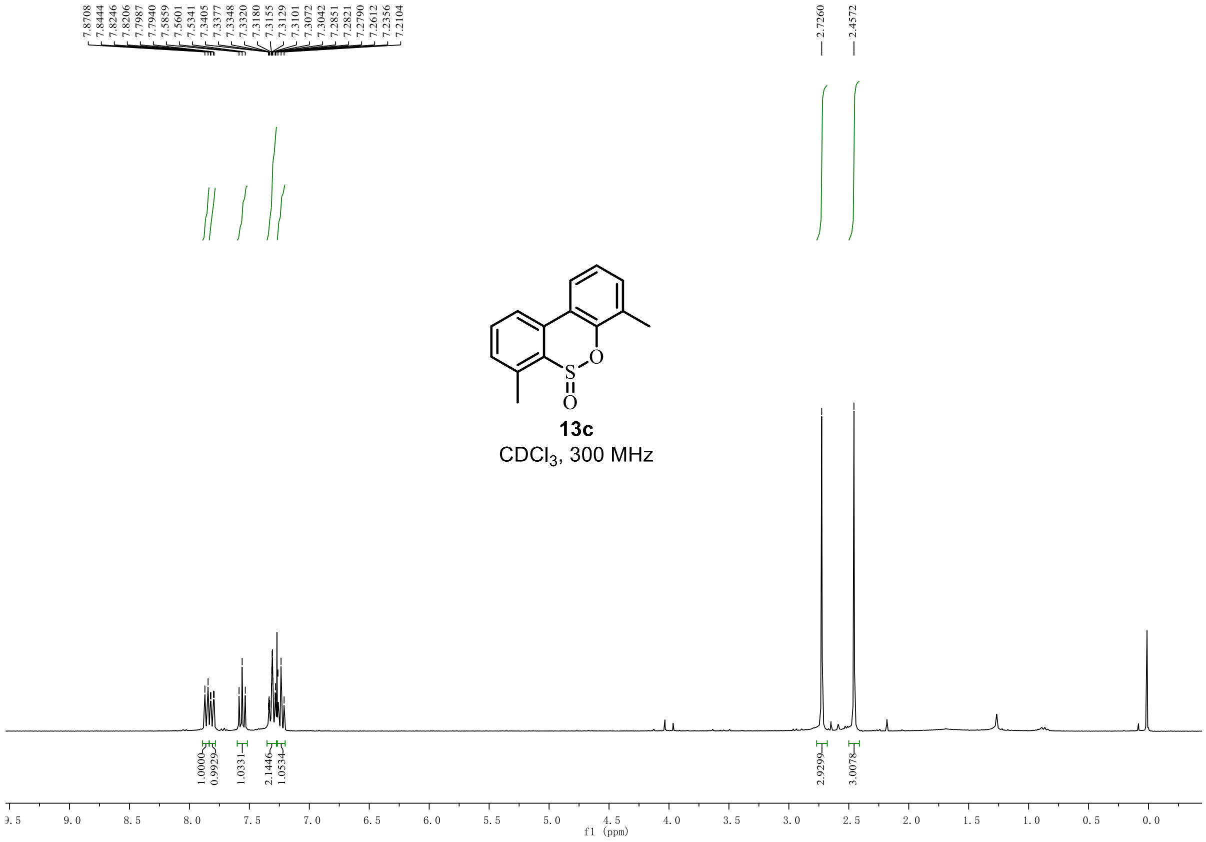

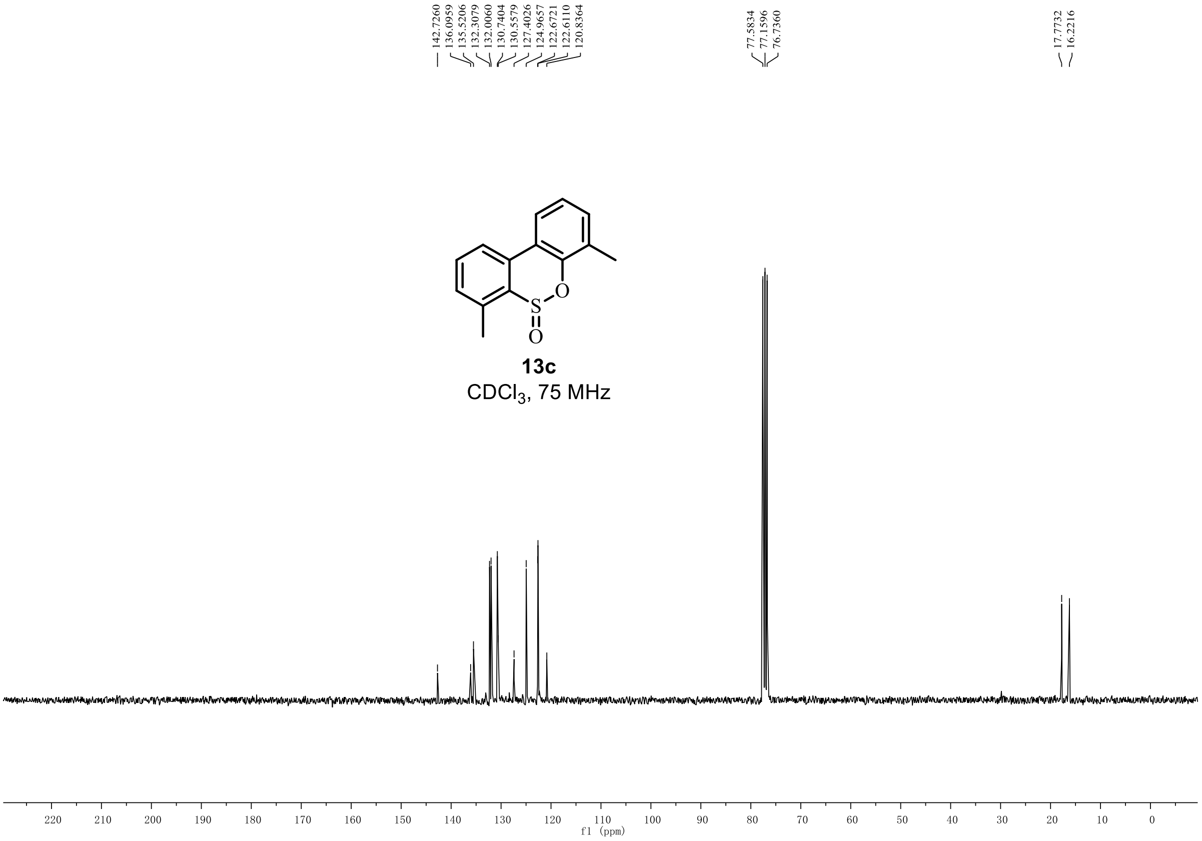

Supplementary Figure 83. NMR spectra of 13c.

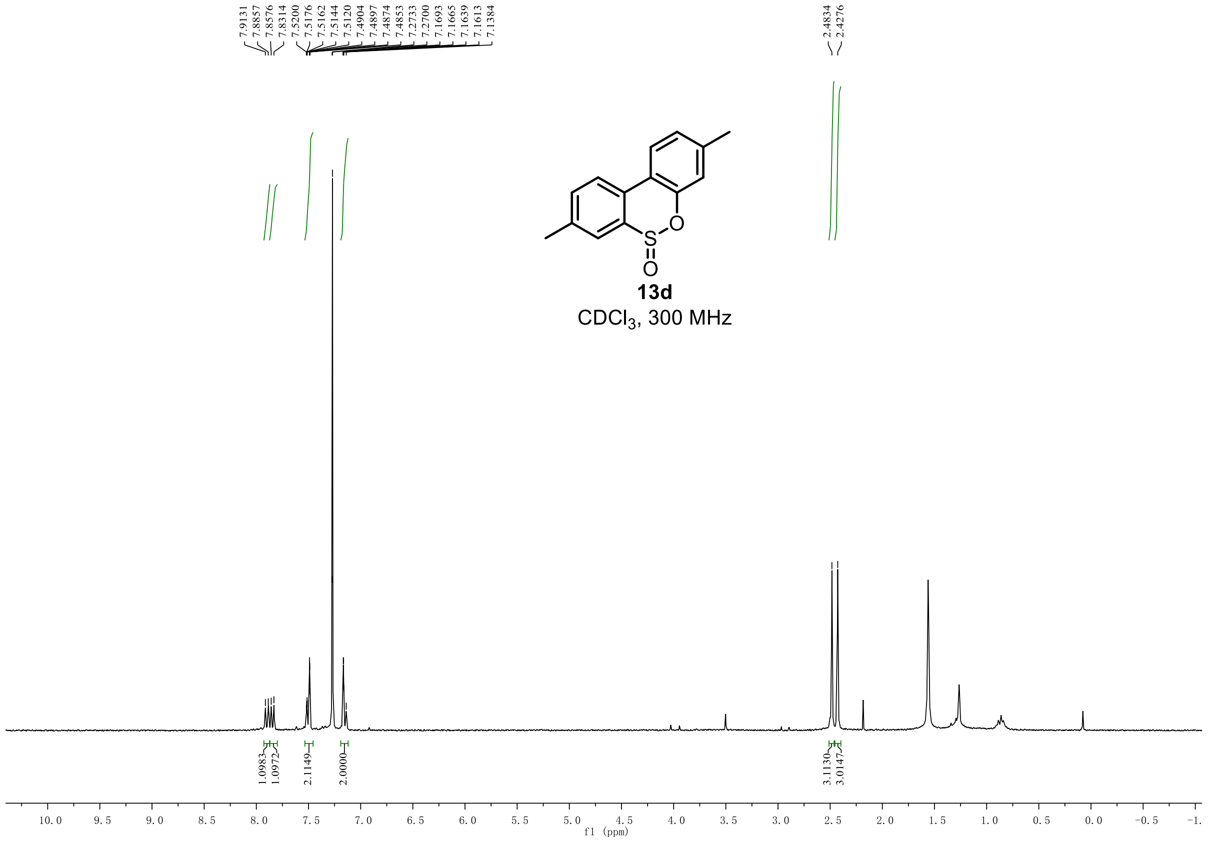

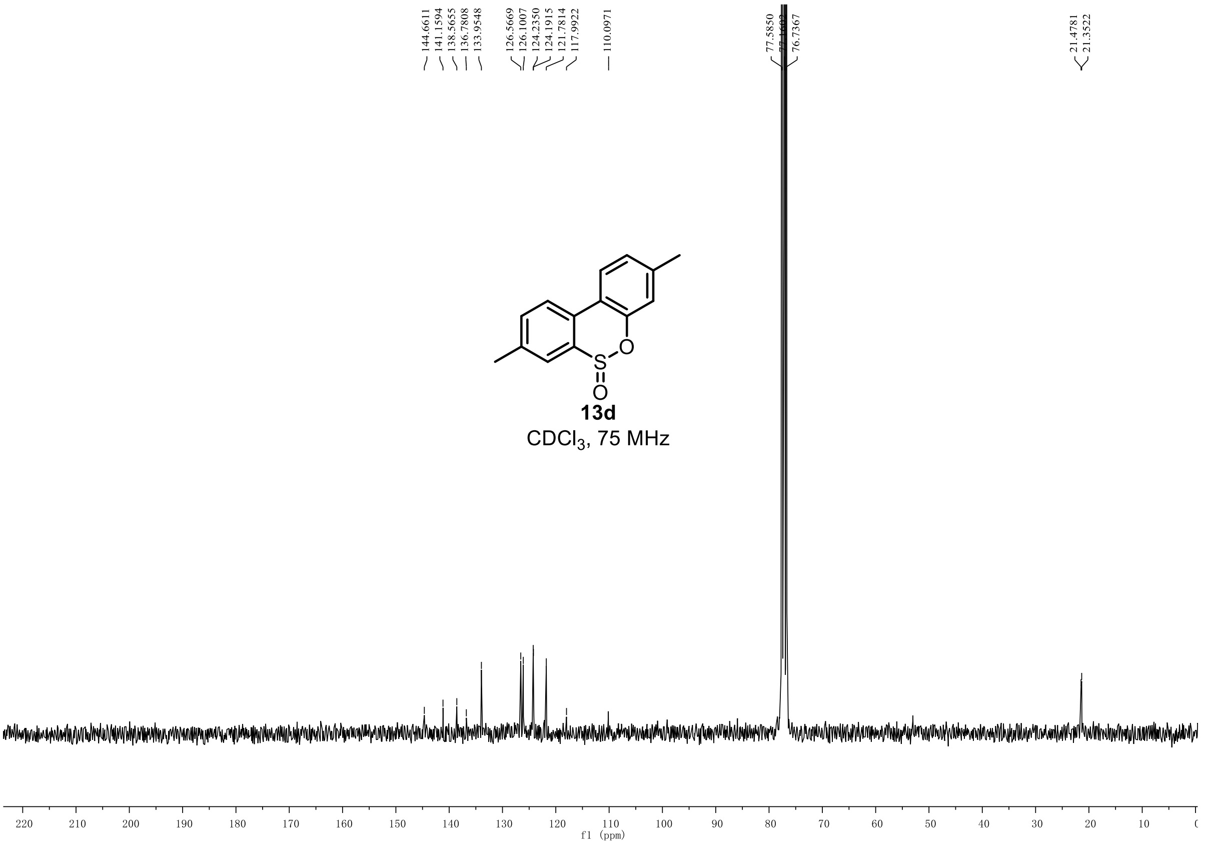

Supplementary Figure 84. NMR spectra of 13d.

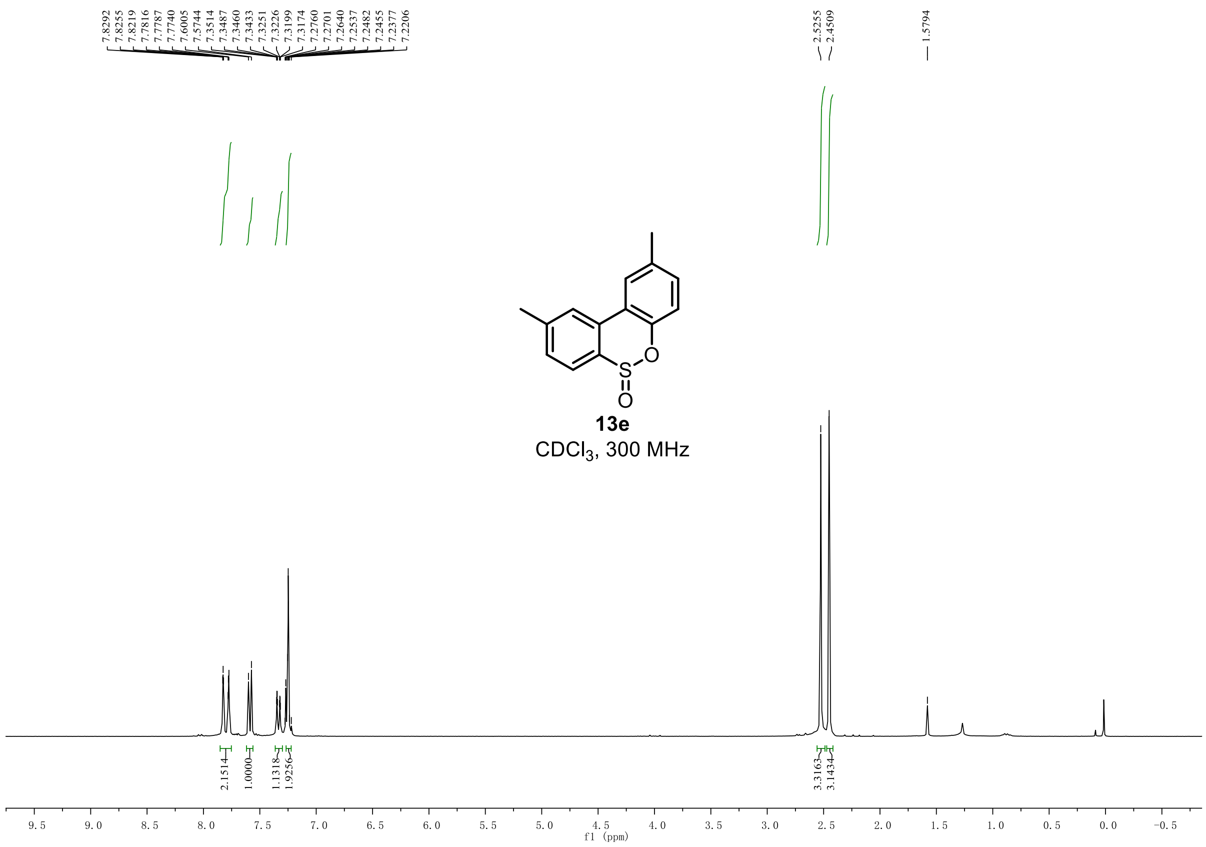

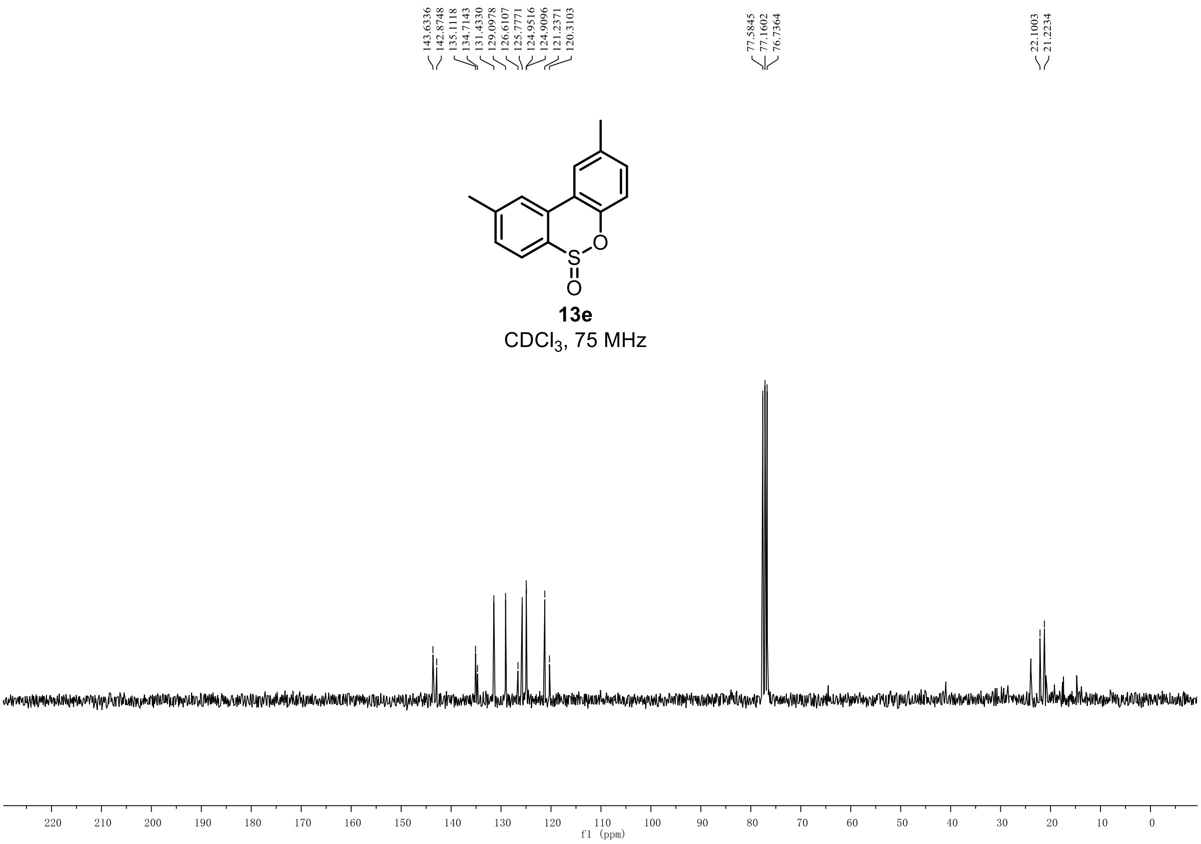

Supplementary Figure 85. NMR spectra of 13e.

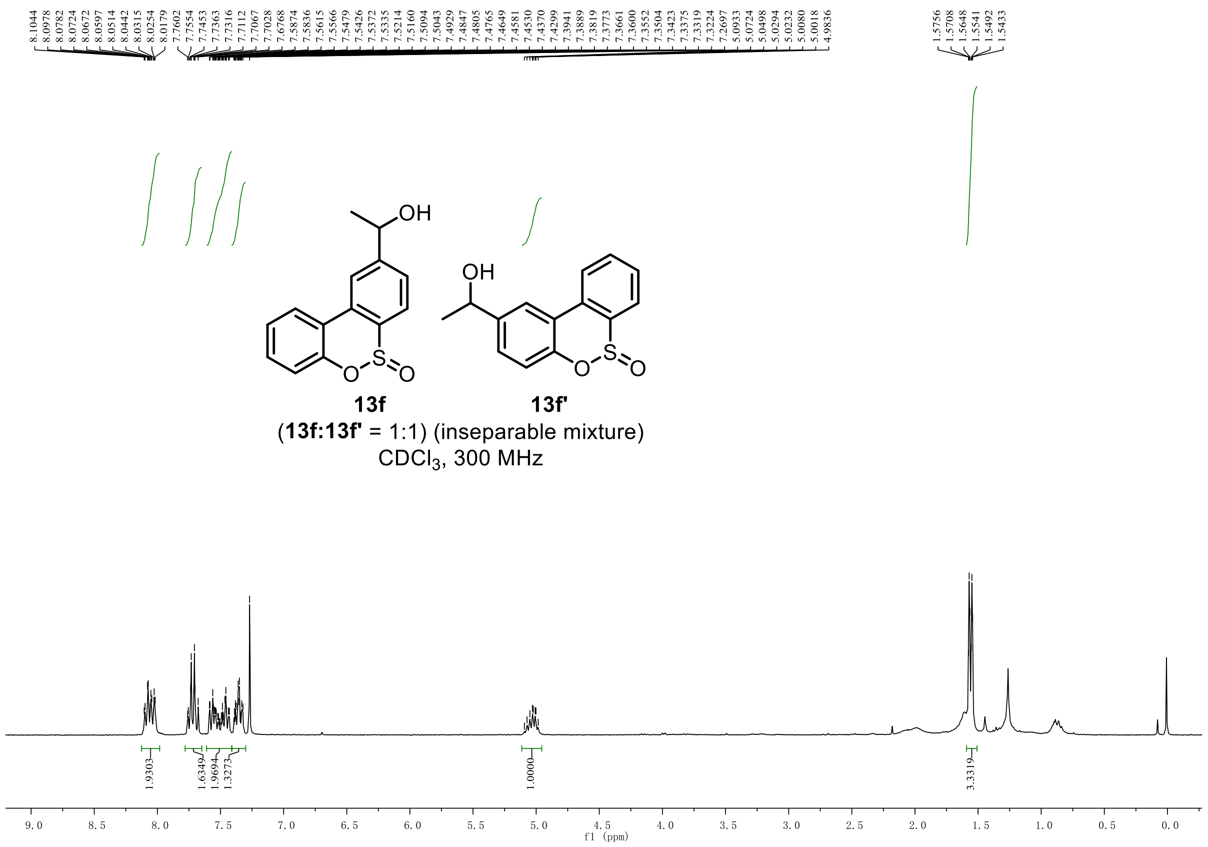

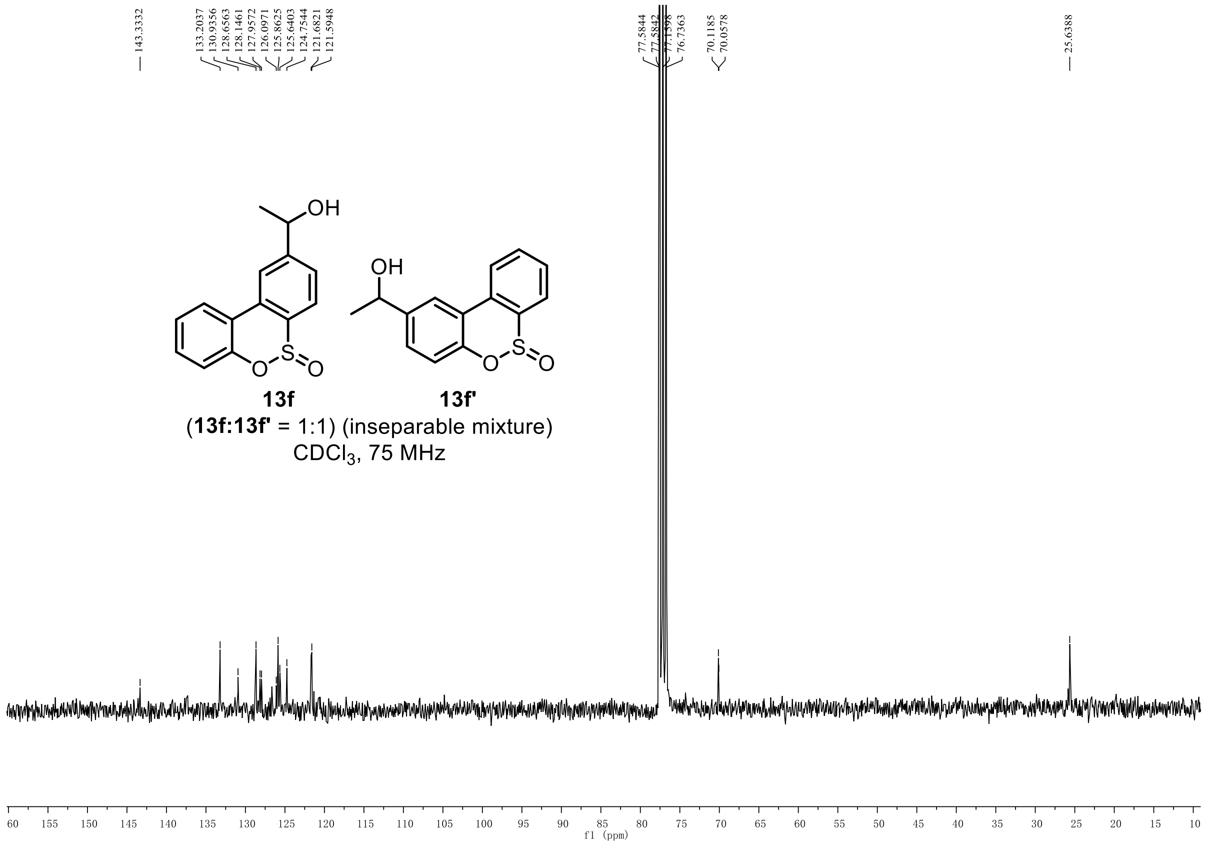

Supplementary Figure 86. NMR spectra of 13f and 13f'.

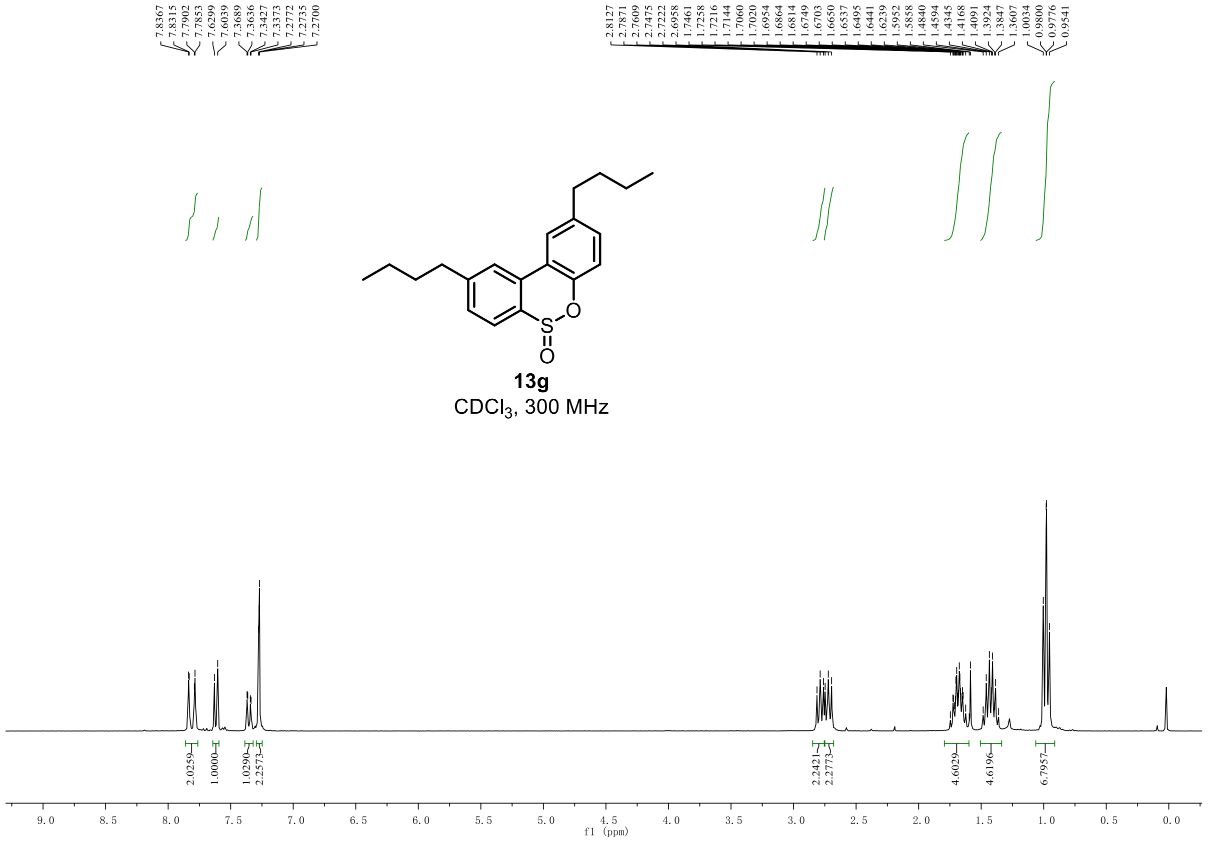

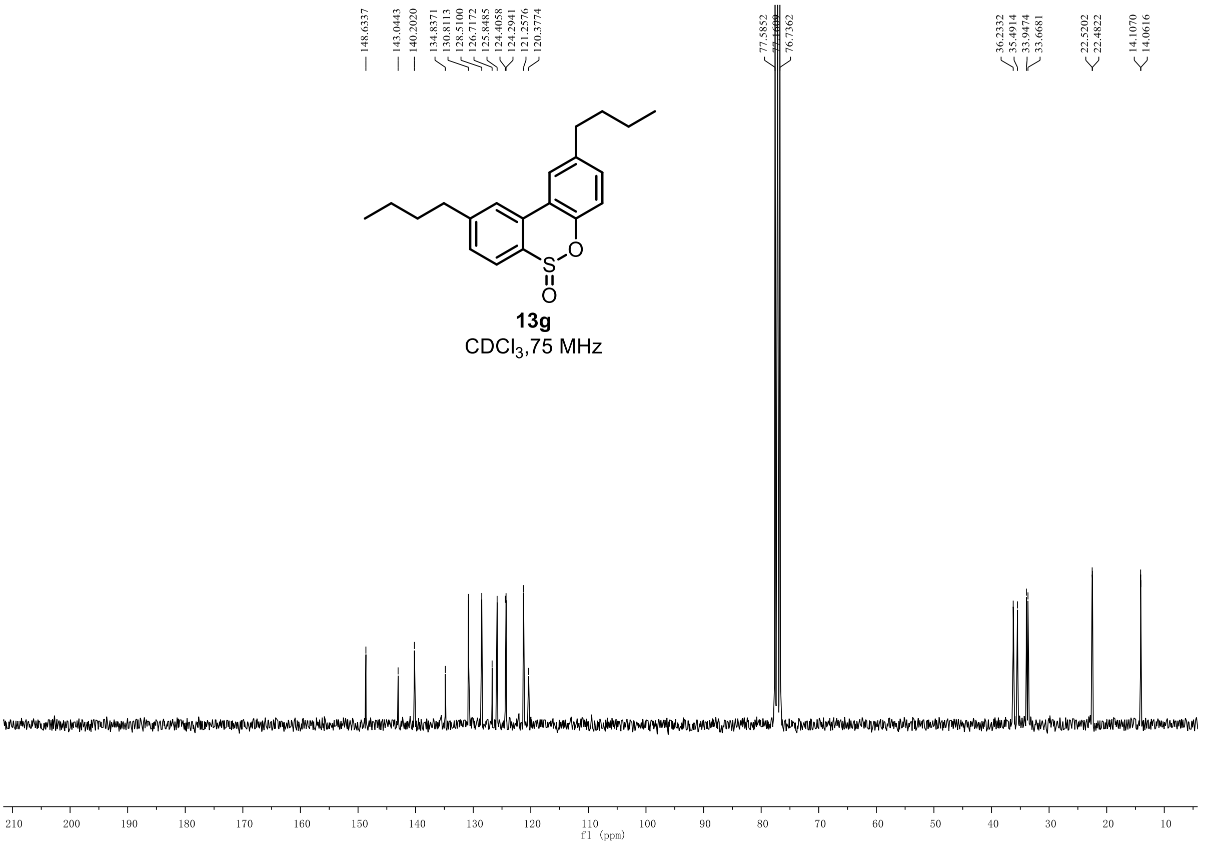

Supplementary Figure 87. NMR spectra of 13g.

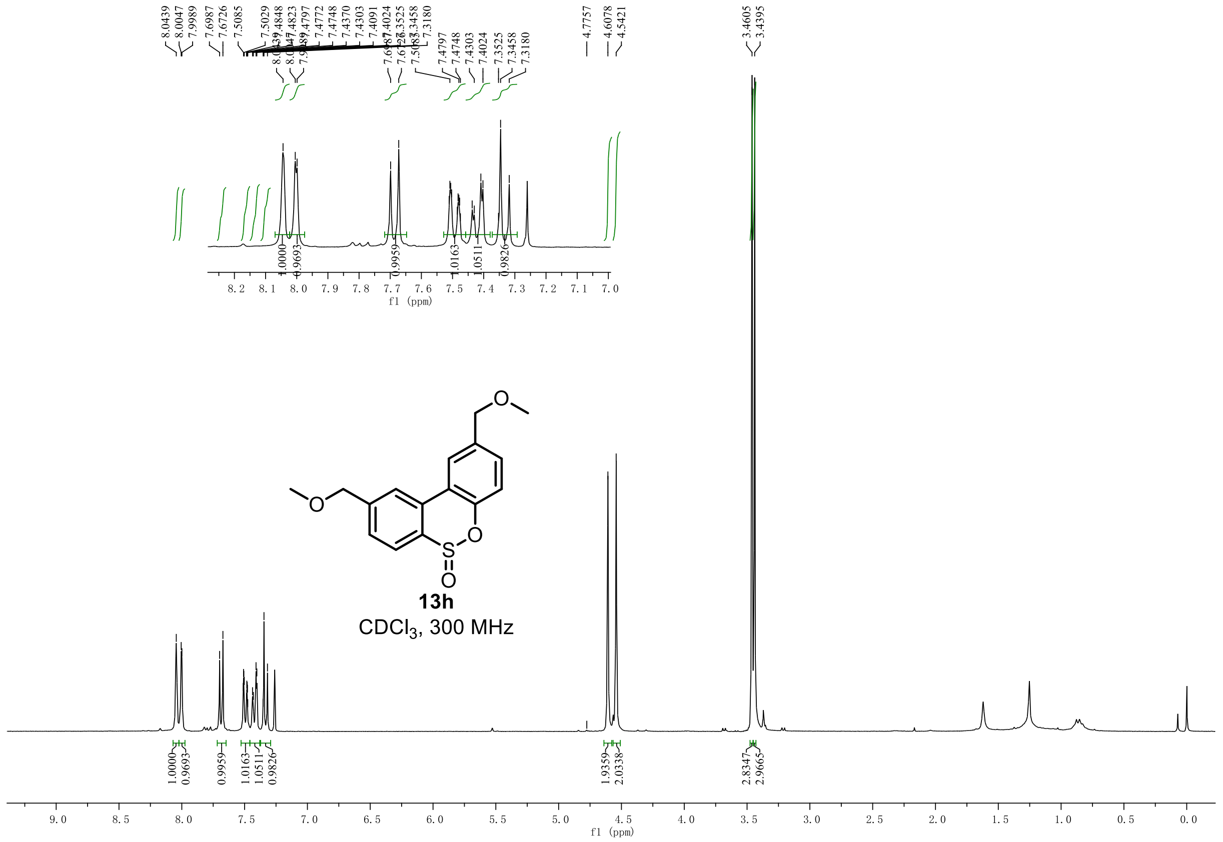

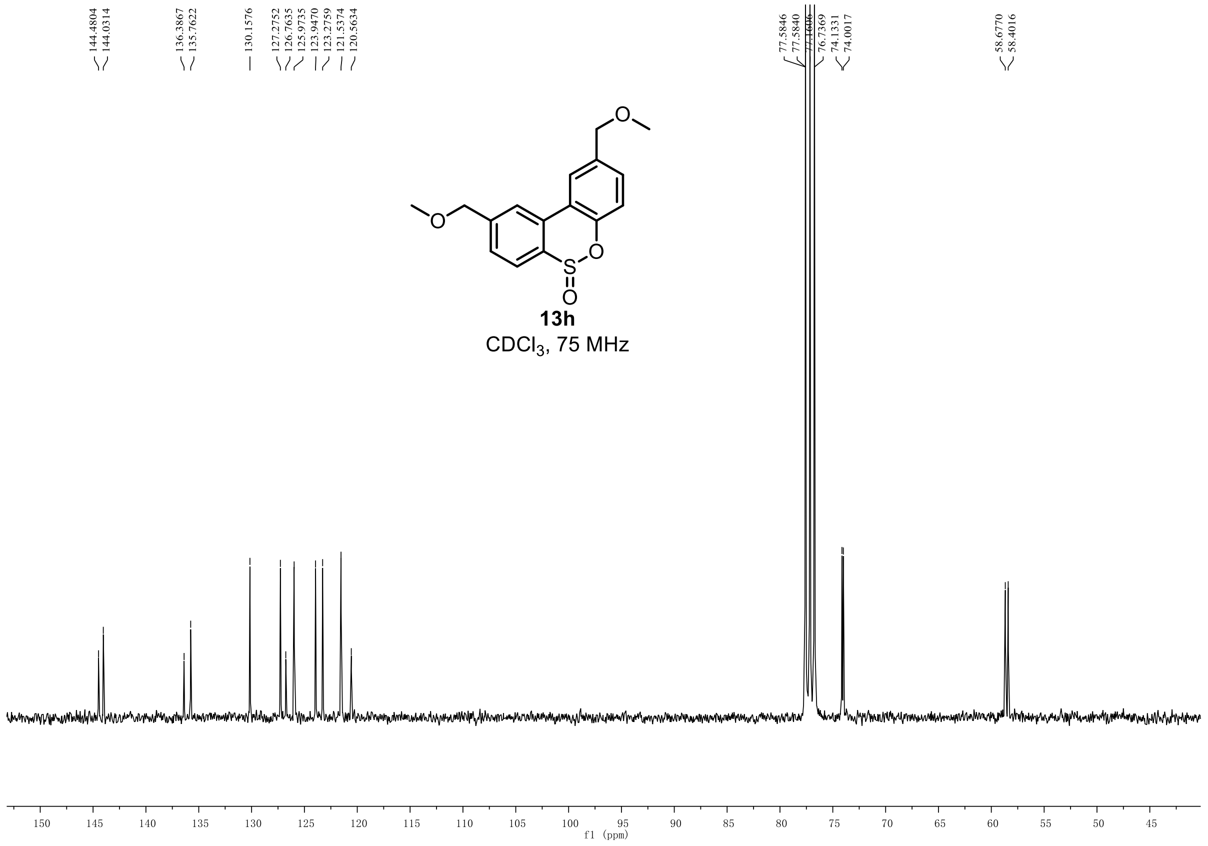

Supplementary Figure 88. NMR spectra of 13h.

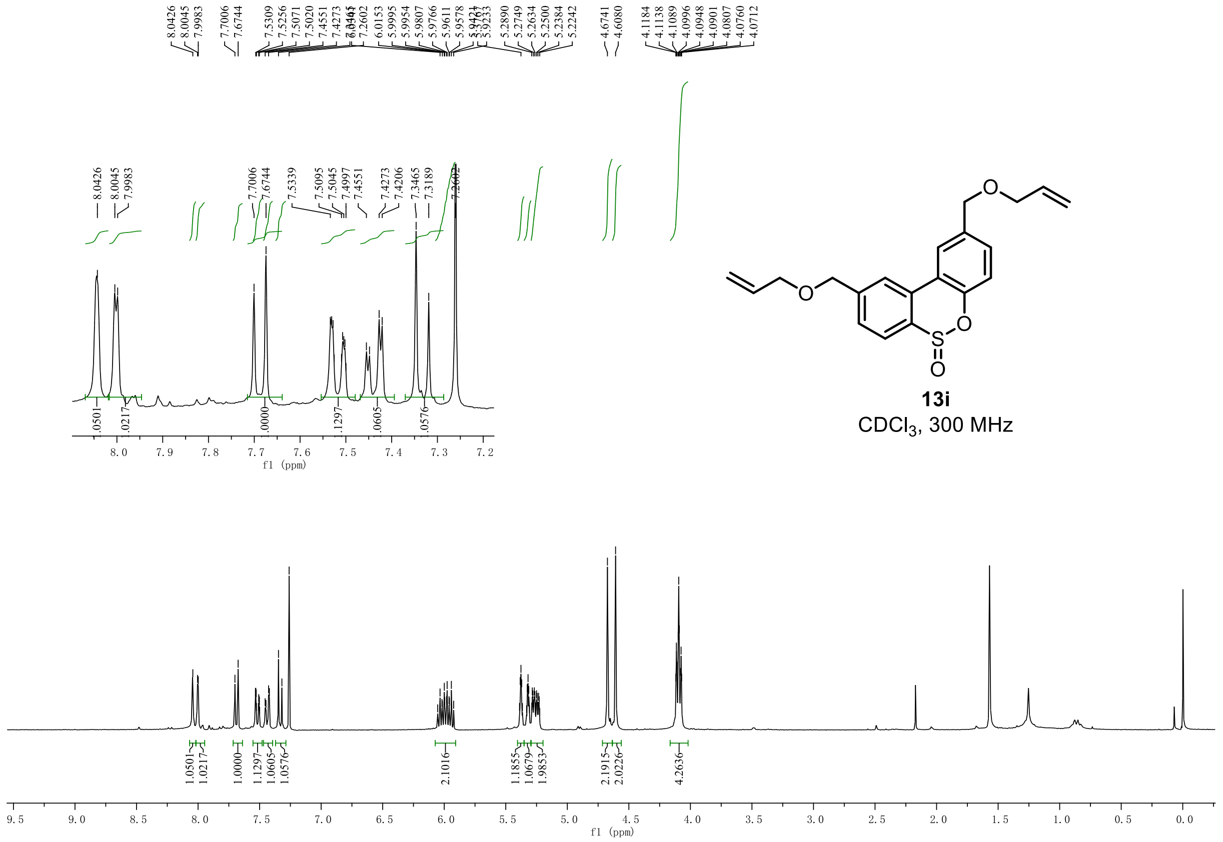

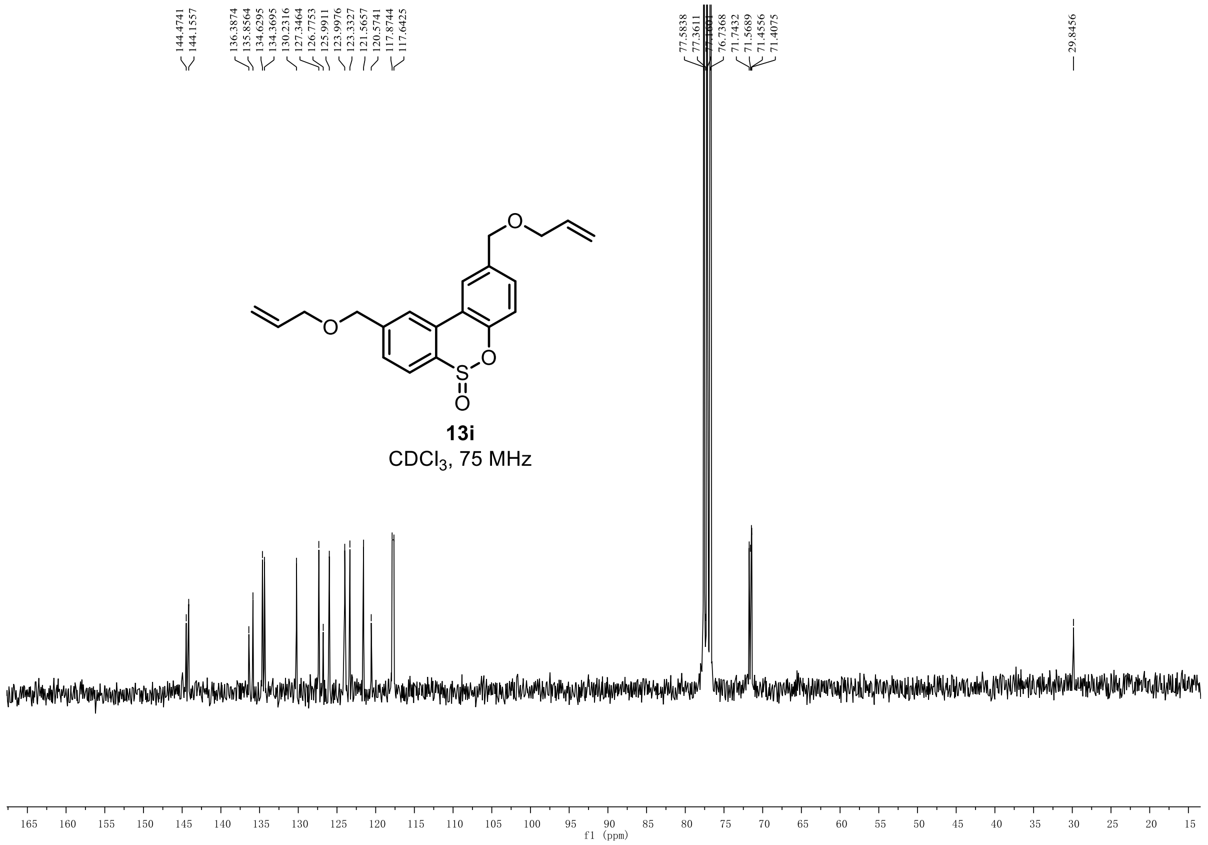

Supplementary Figure 89. NMR spectra of 13i.

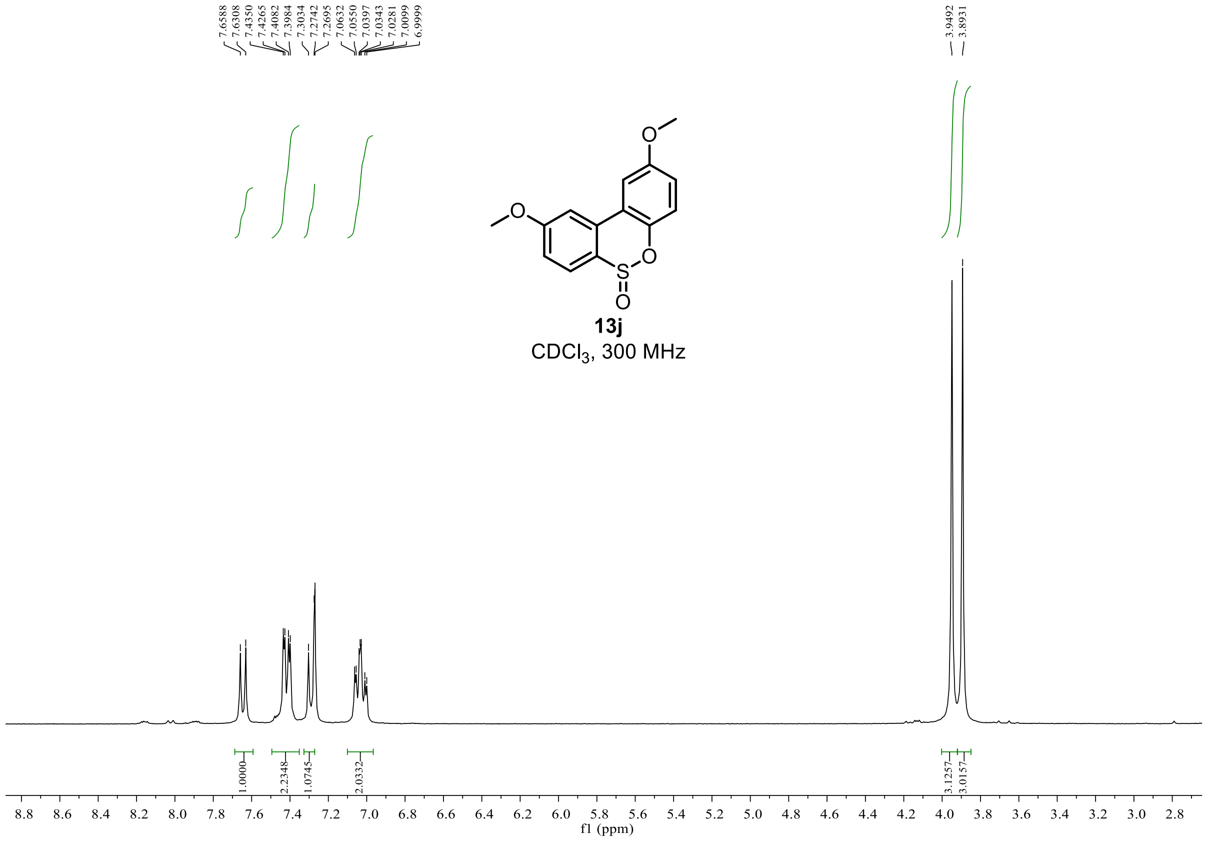

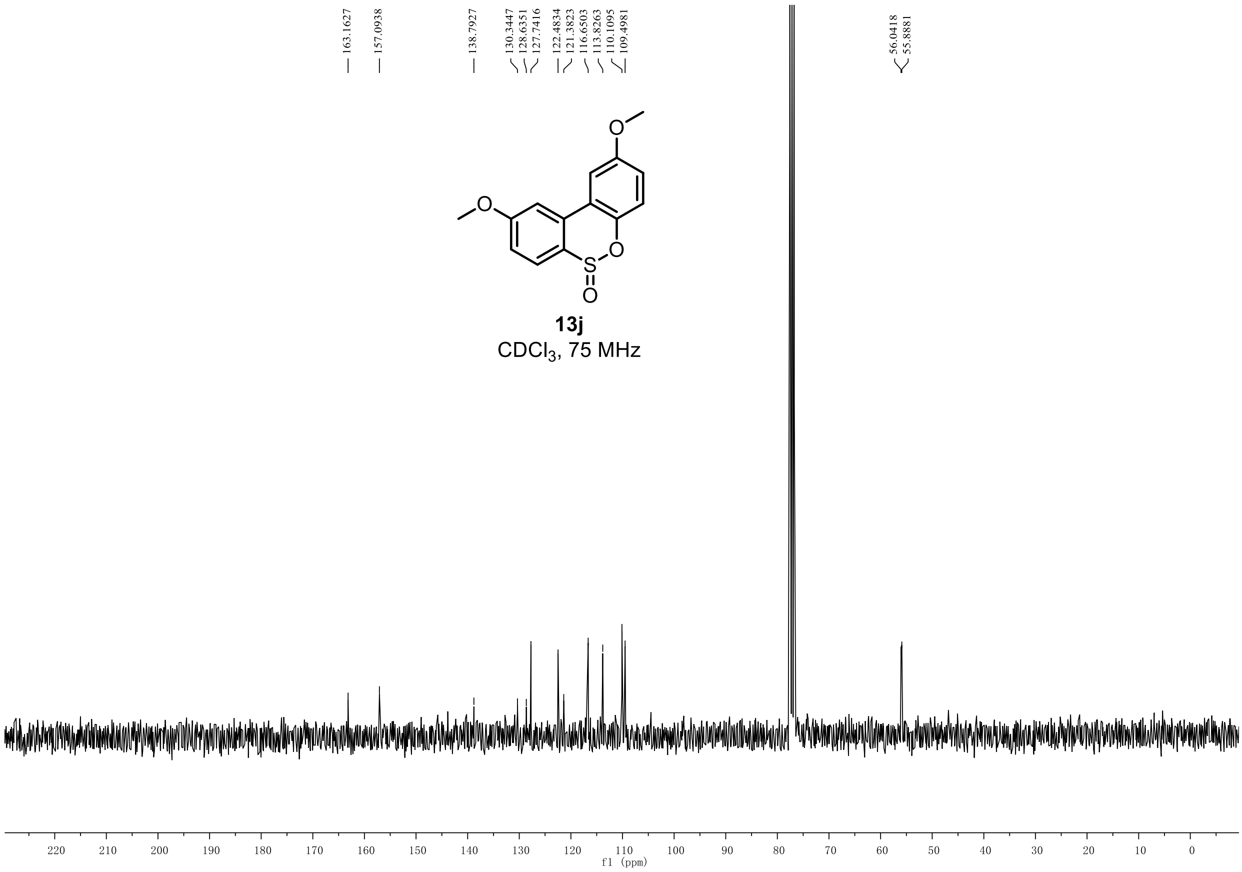

Supplementary Figure 90. NMR spectra of 13j.

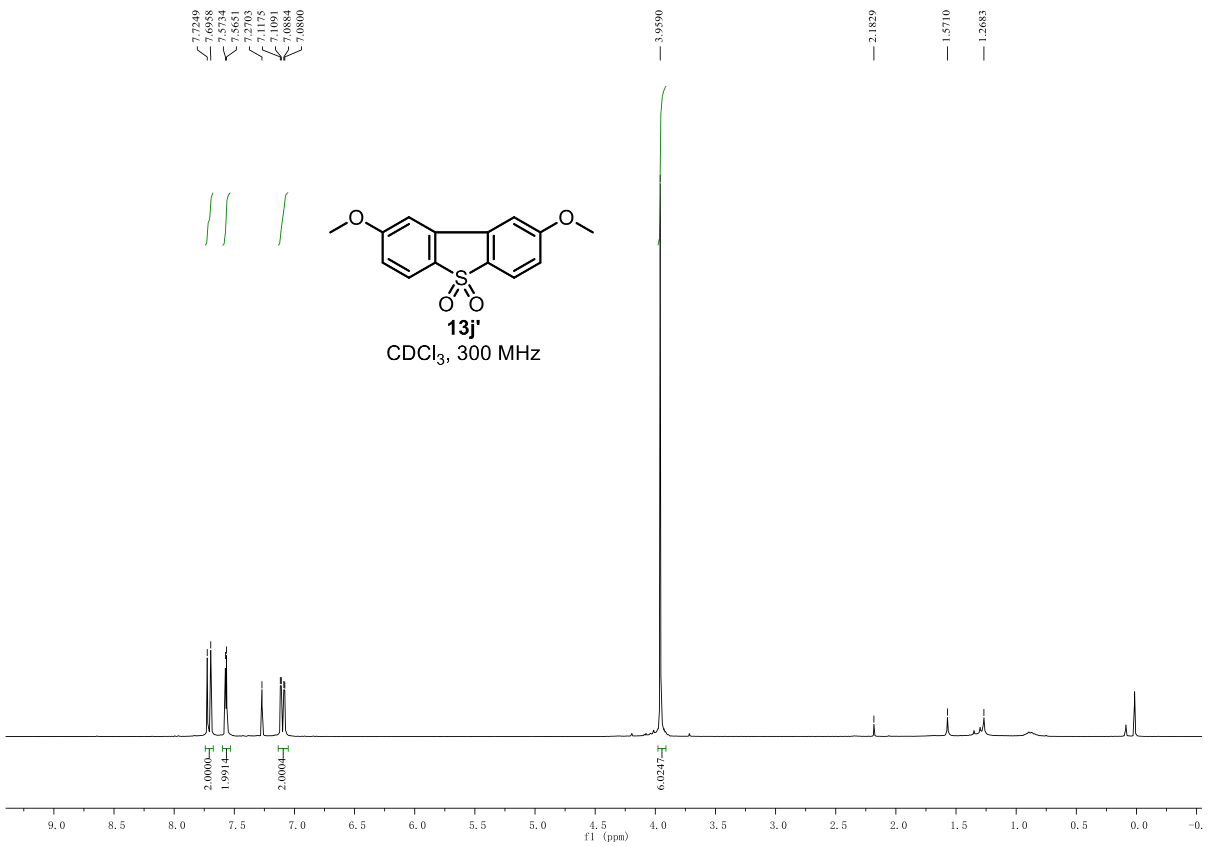

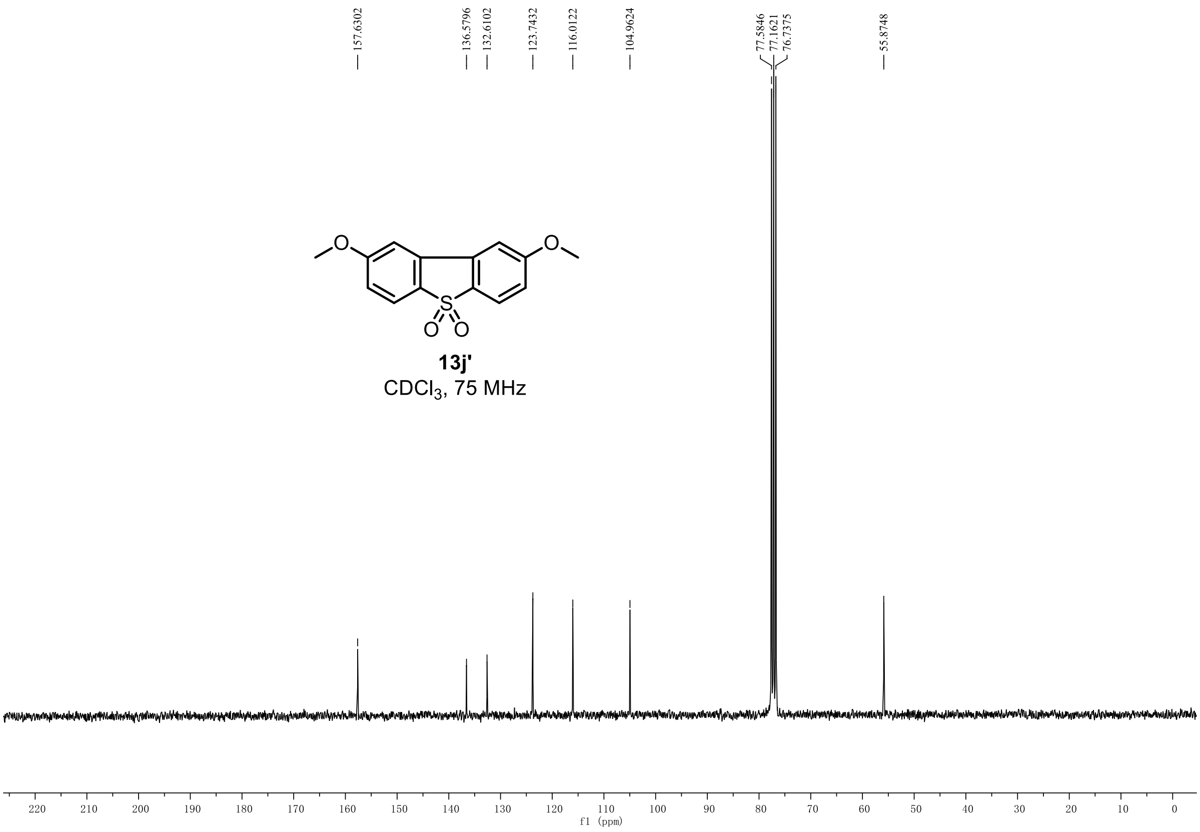

Supplementary Figure 91. NMR spectra of 13j'.

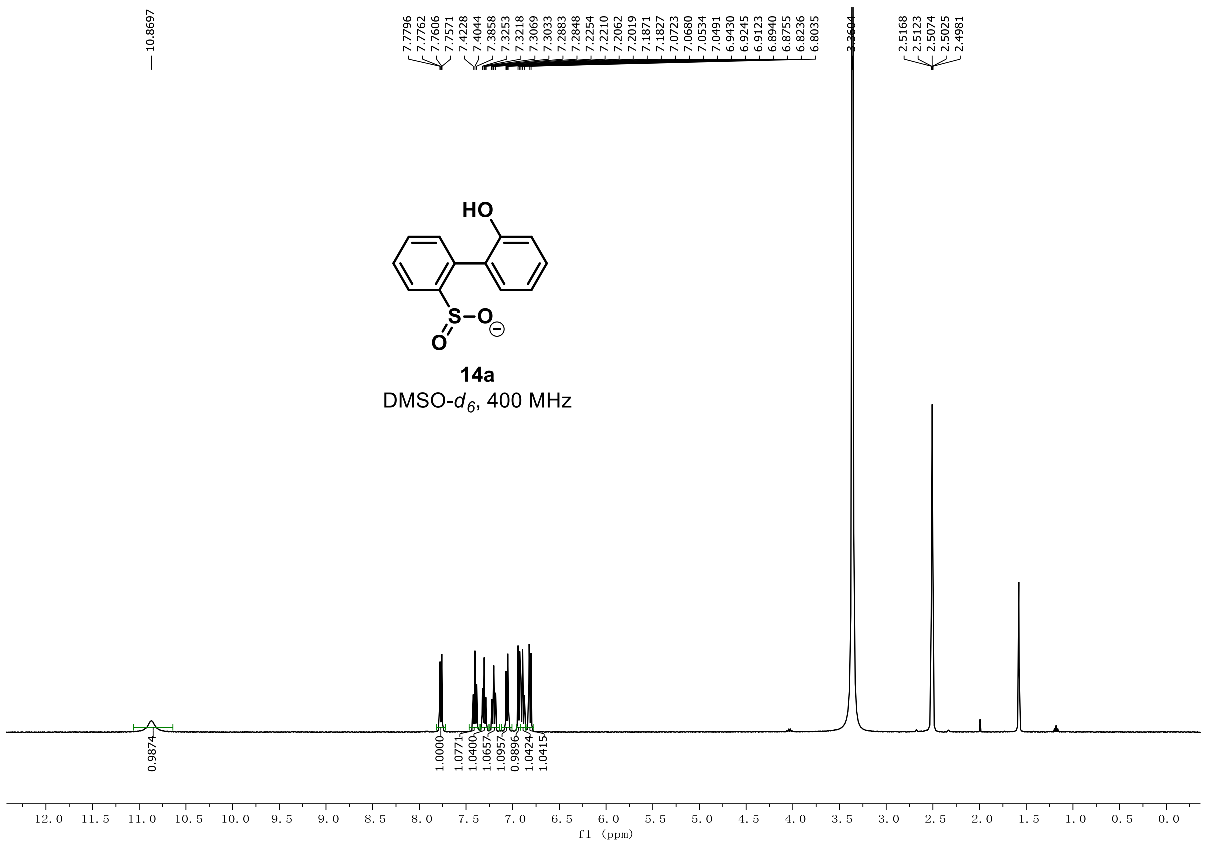

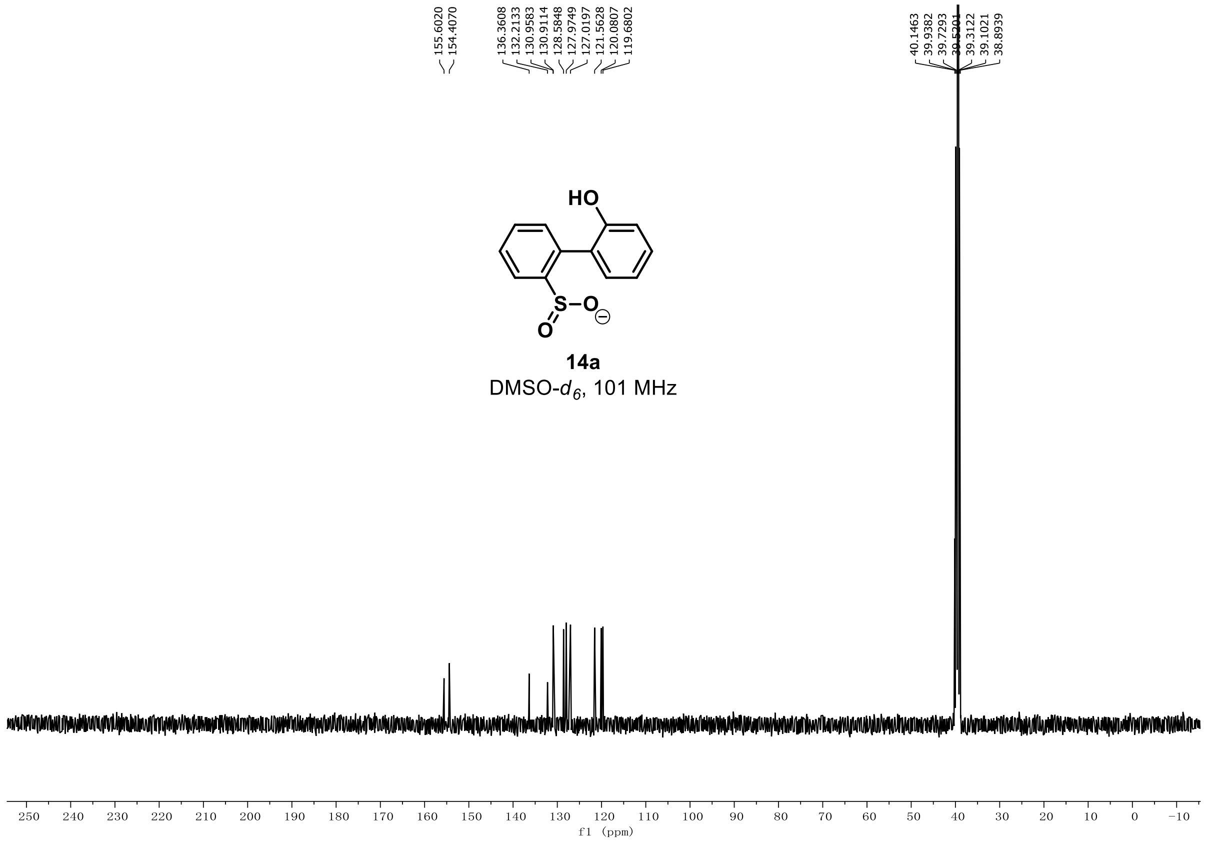

Supplementary Figure 92. NMR spectra of 14a.

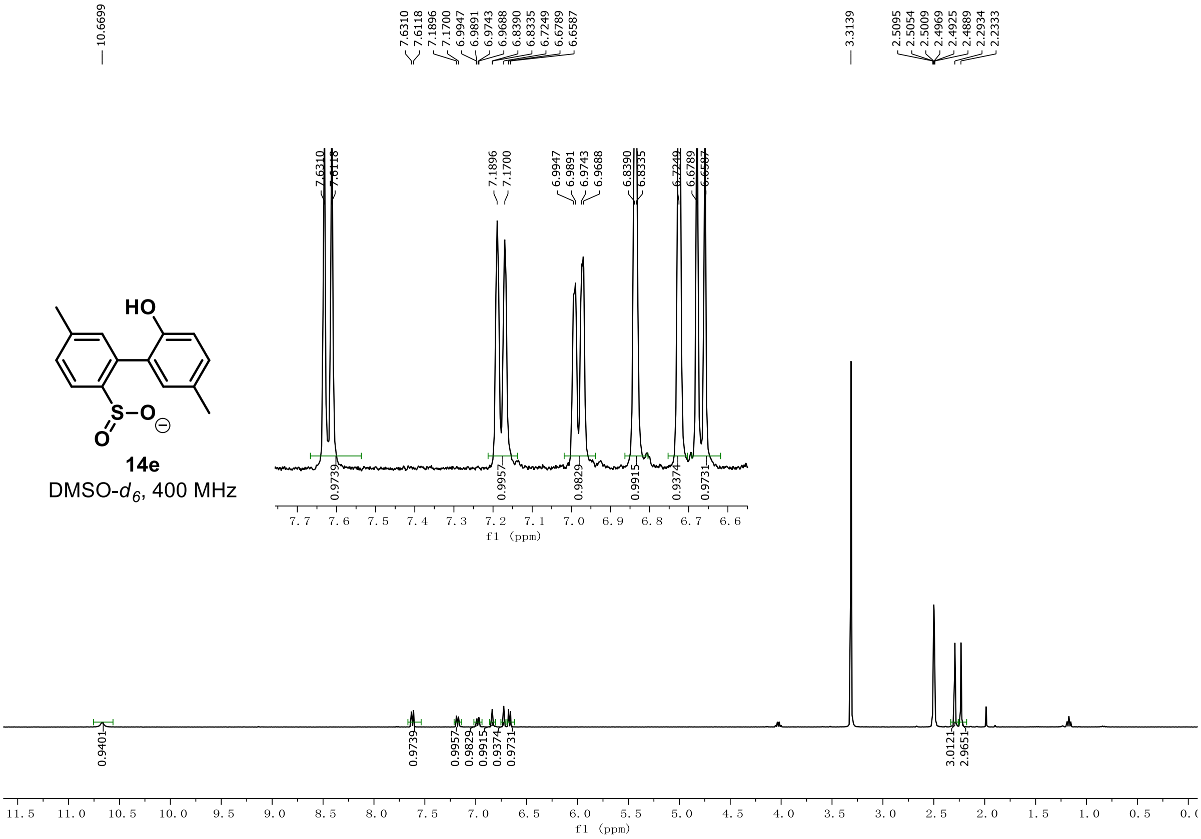

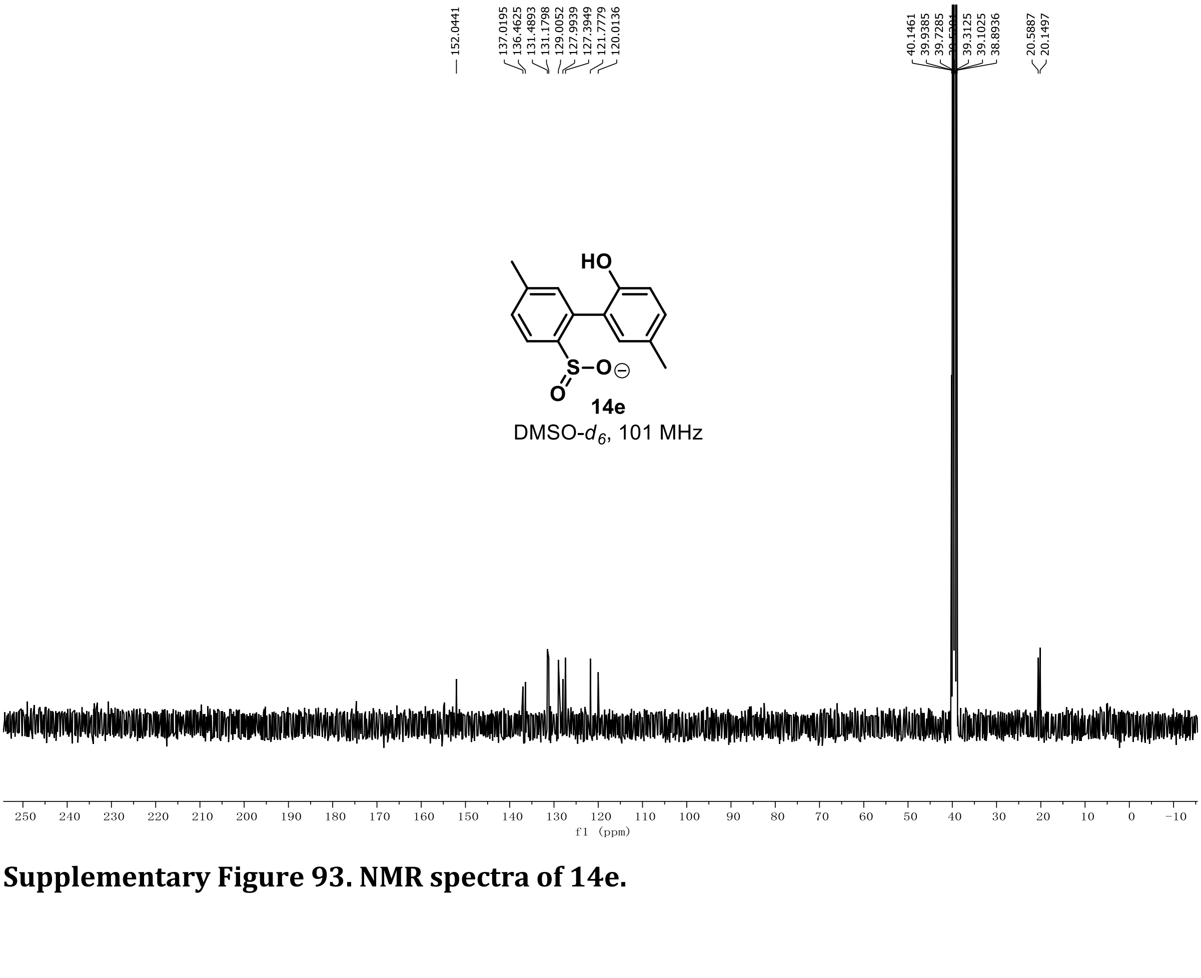

Supplementary Figure 93. NMR spectra of 14e.

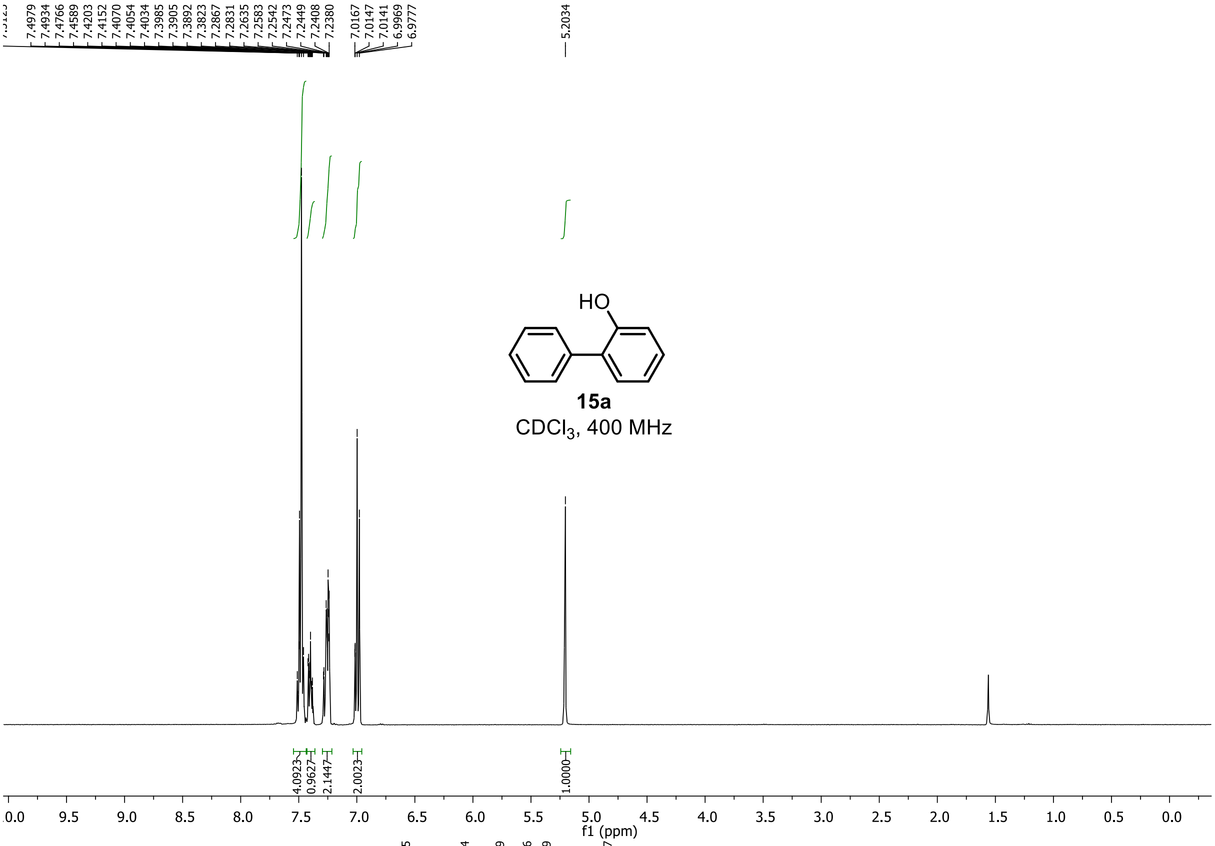

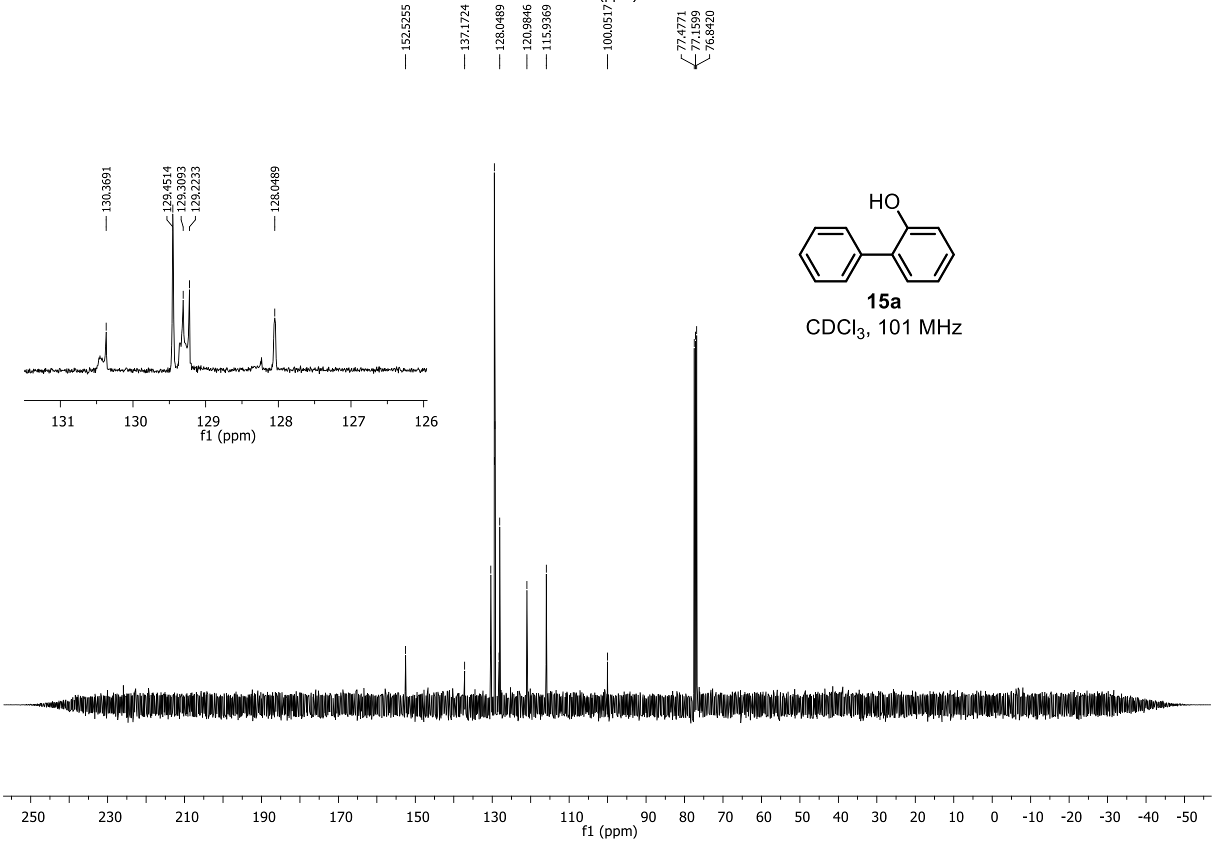

Supplementary Figure 94. NMR spectra of 15a.

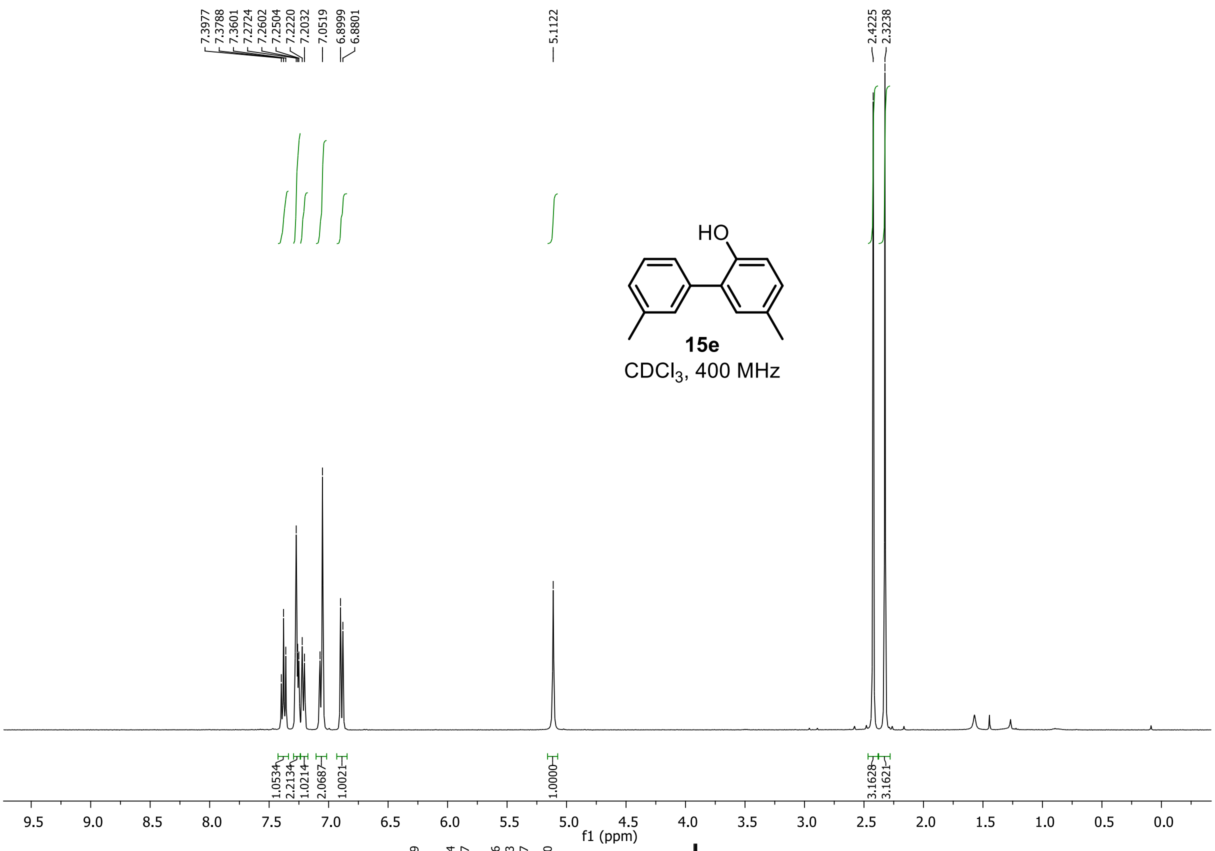

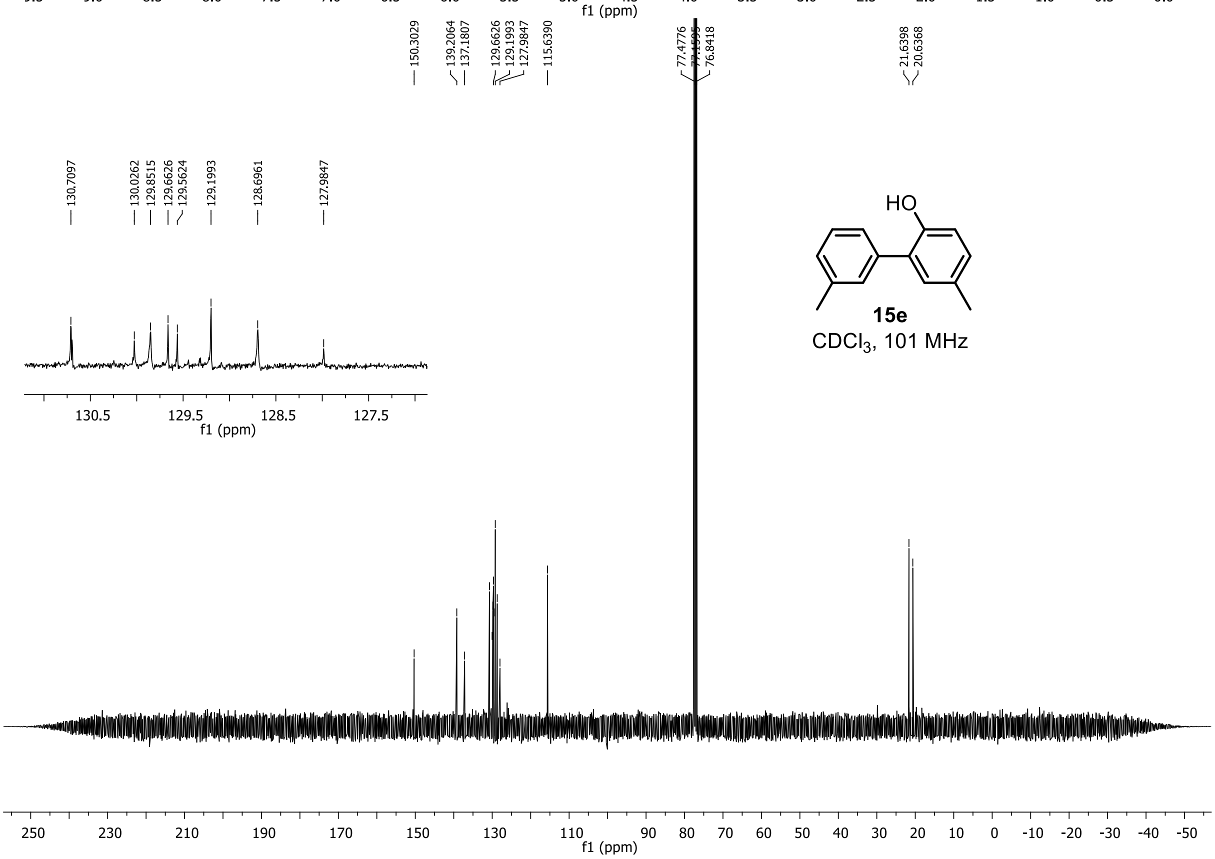

Supplementary Figure 95. NMR spectra of 15e.

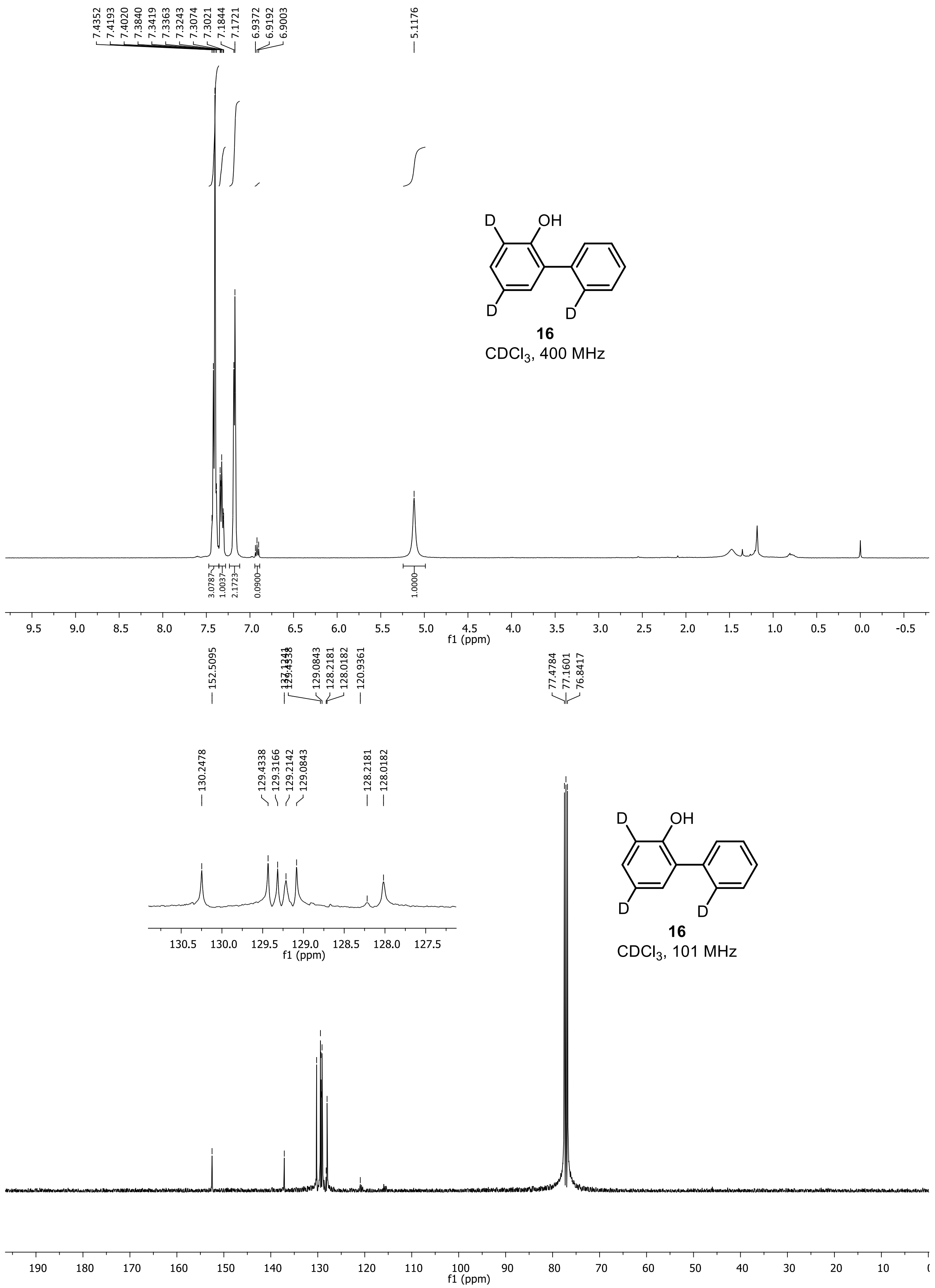

Supplementary Figure 96. NMR spectra of 16.

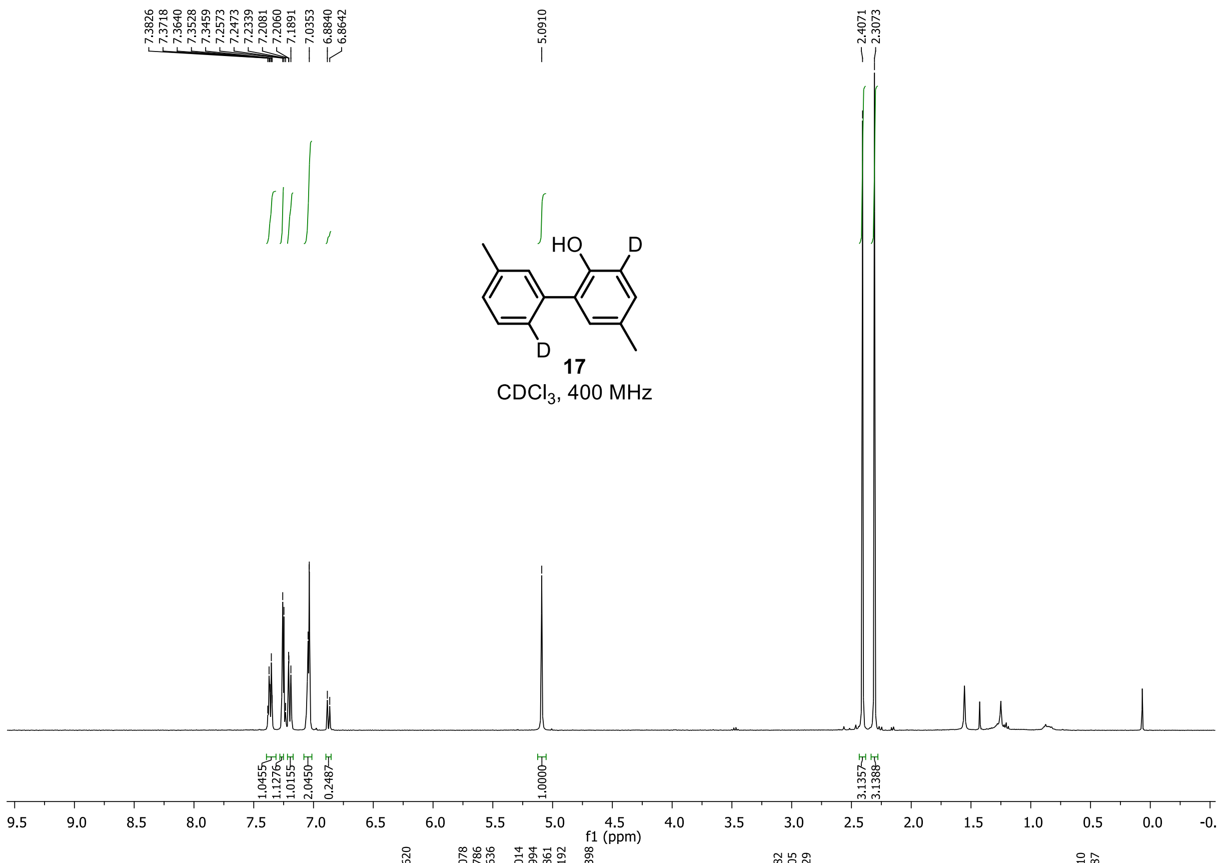

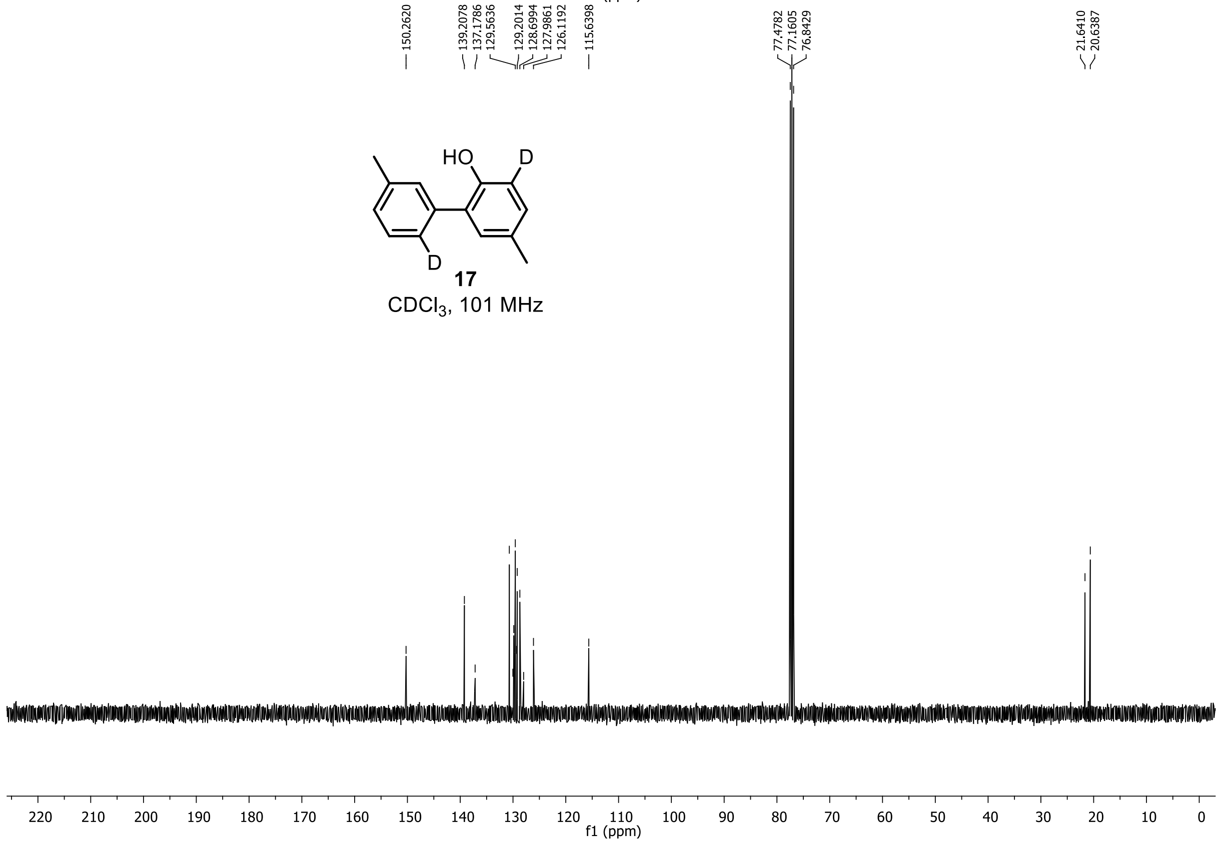

Supplementary Figure 97. NMR spectra of 17.

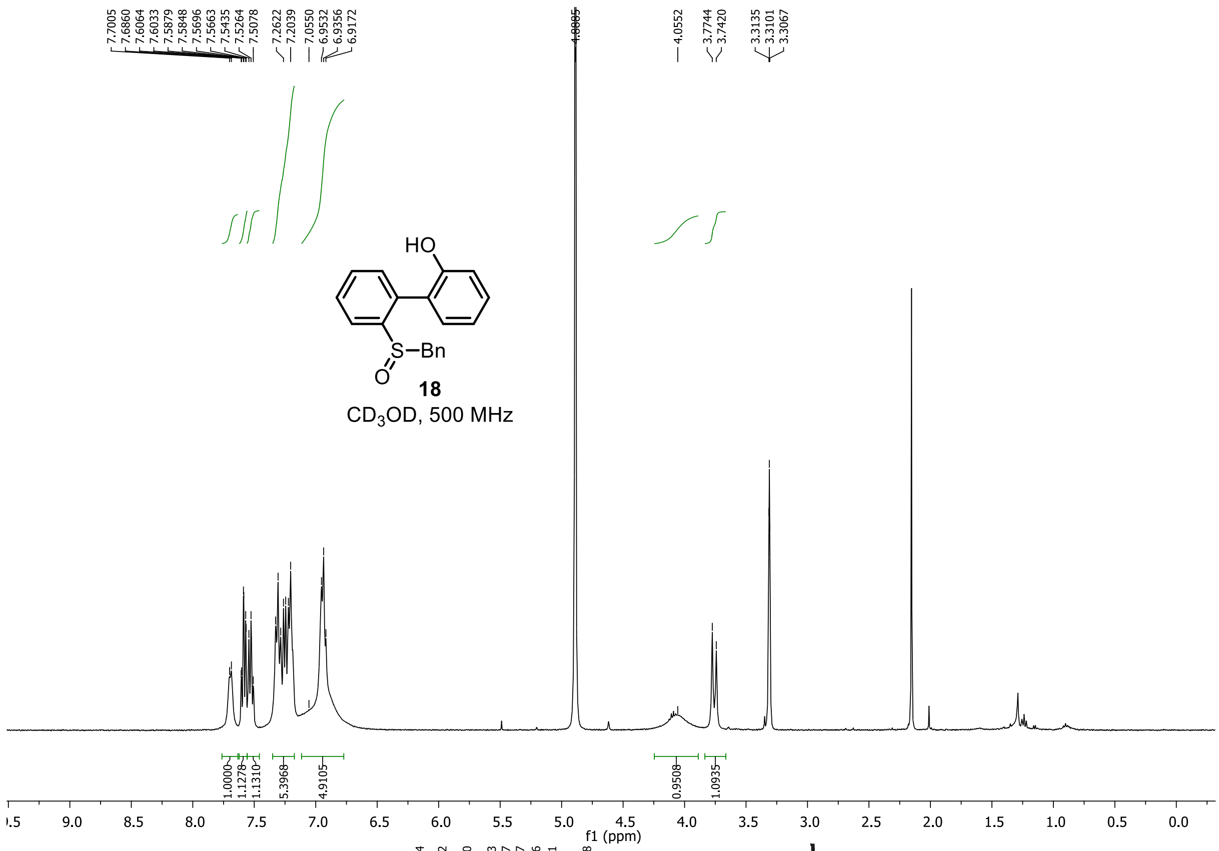

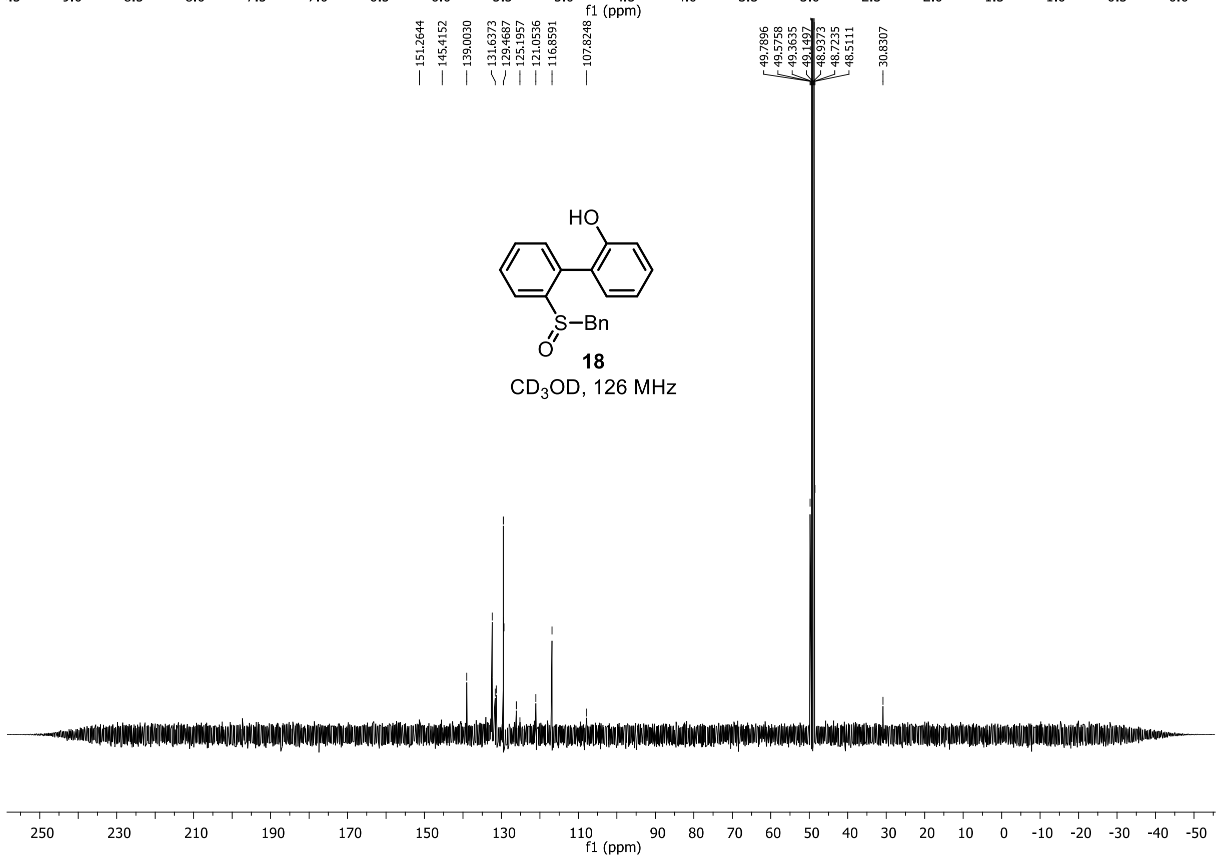

Supplementary Figure 98. NMR spectra of 18.

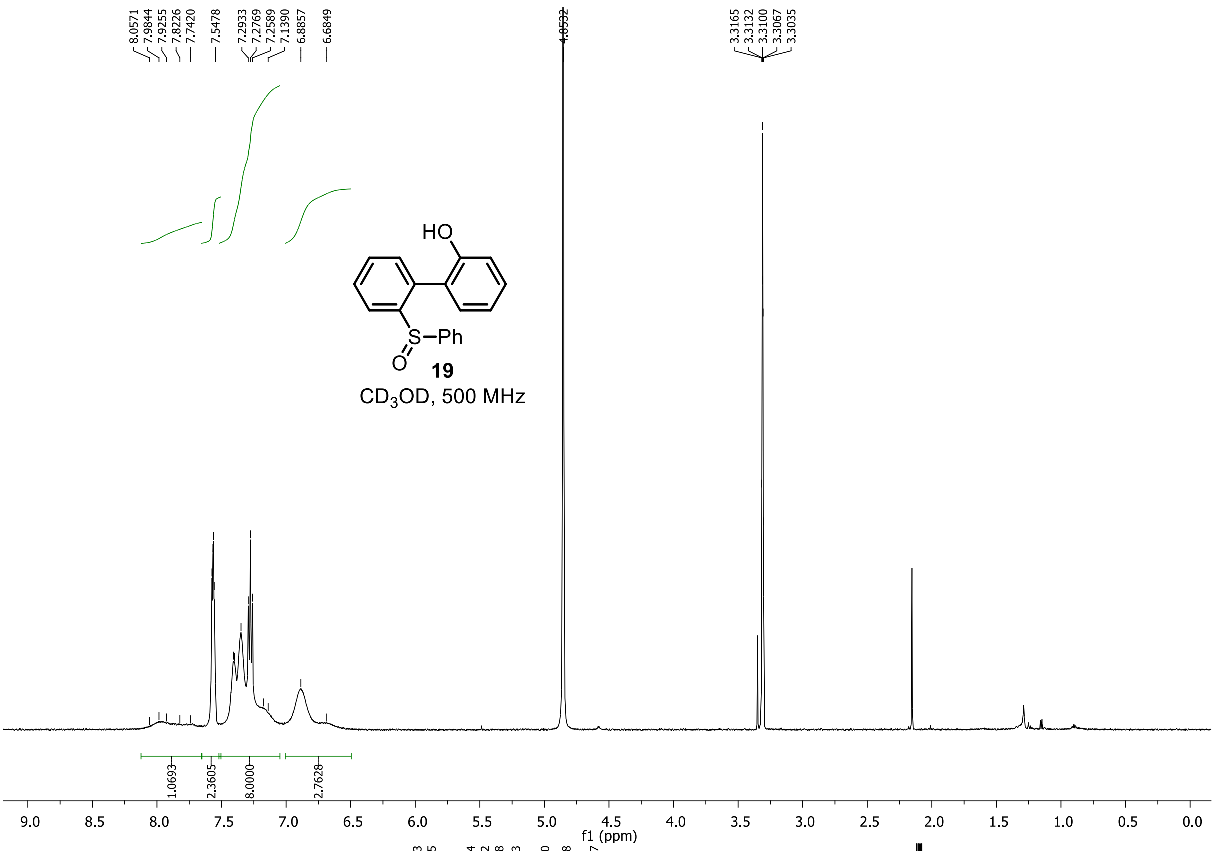

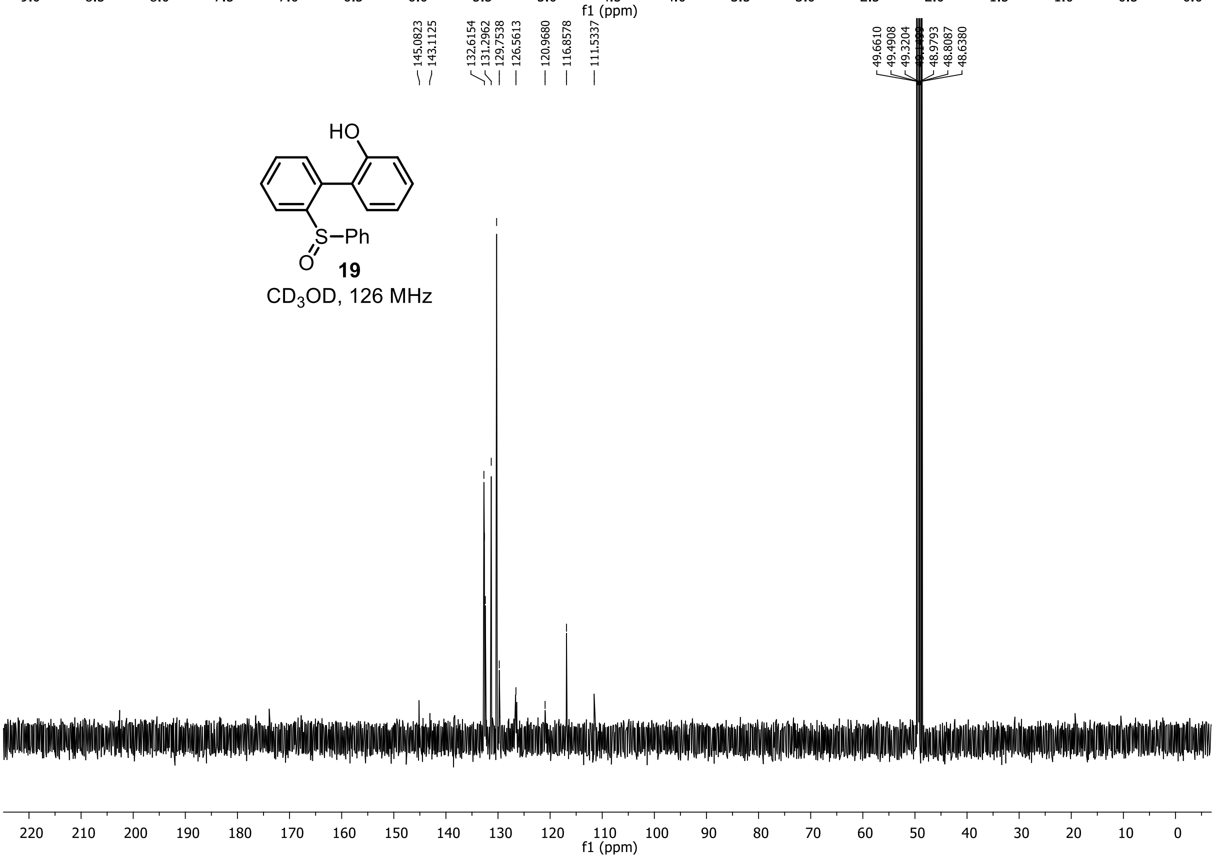

Supplementary Figure 99. NMR spectra of 19.

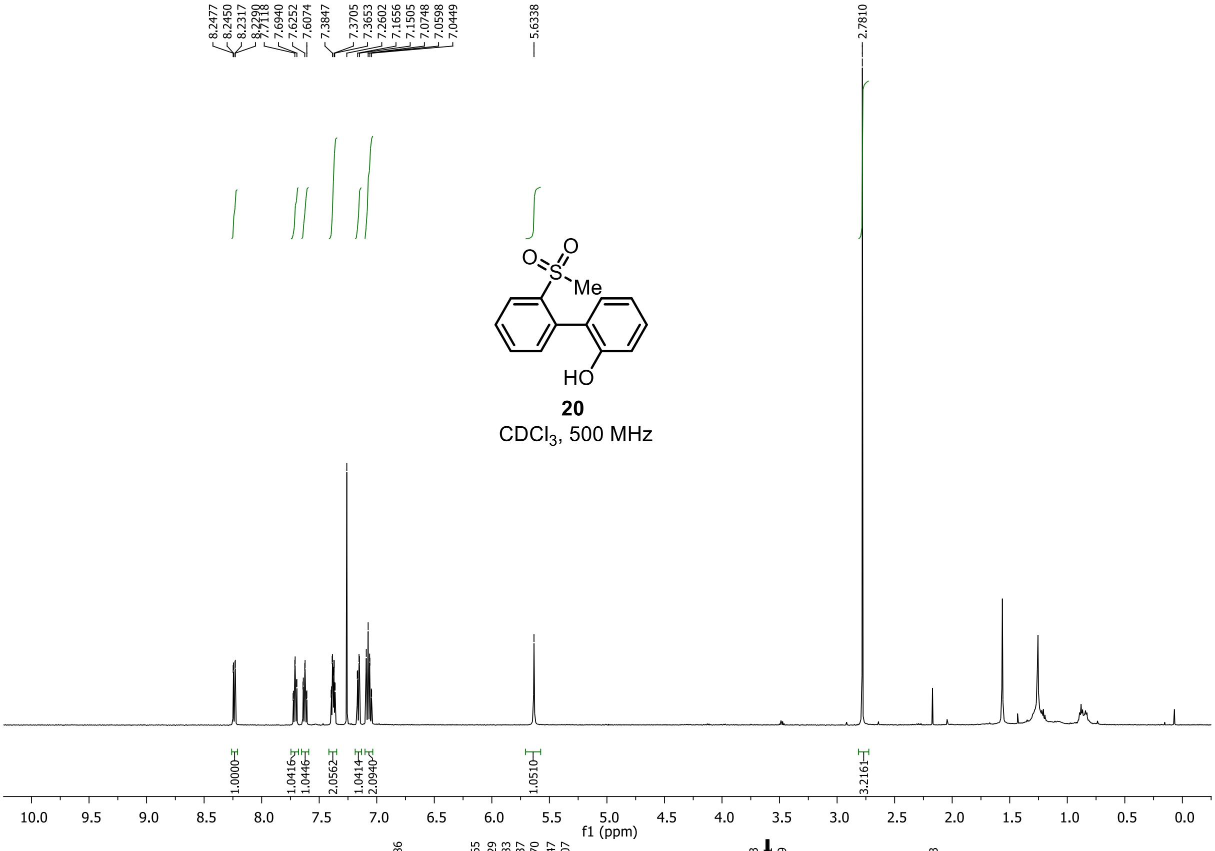

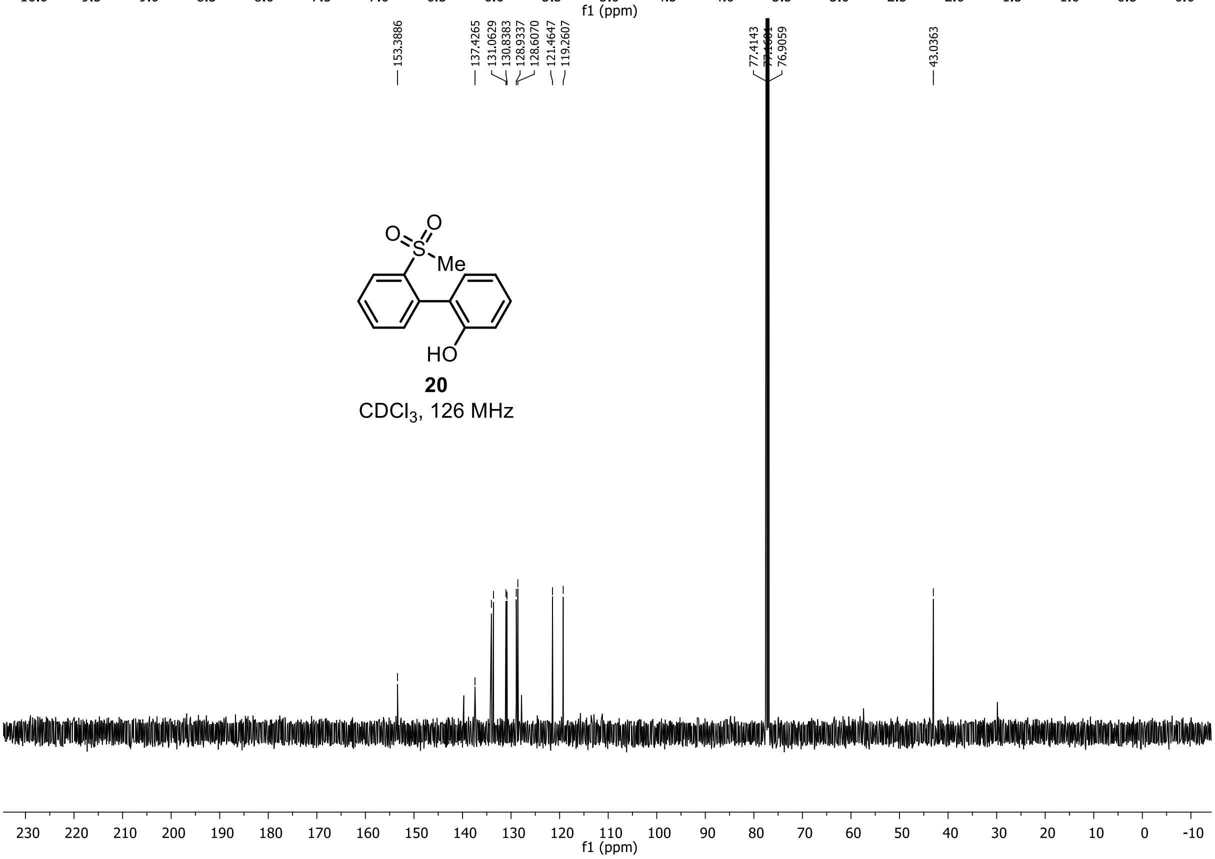

Supplementary Figure 100. NMR spectra of 20.

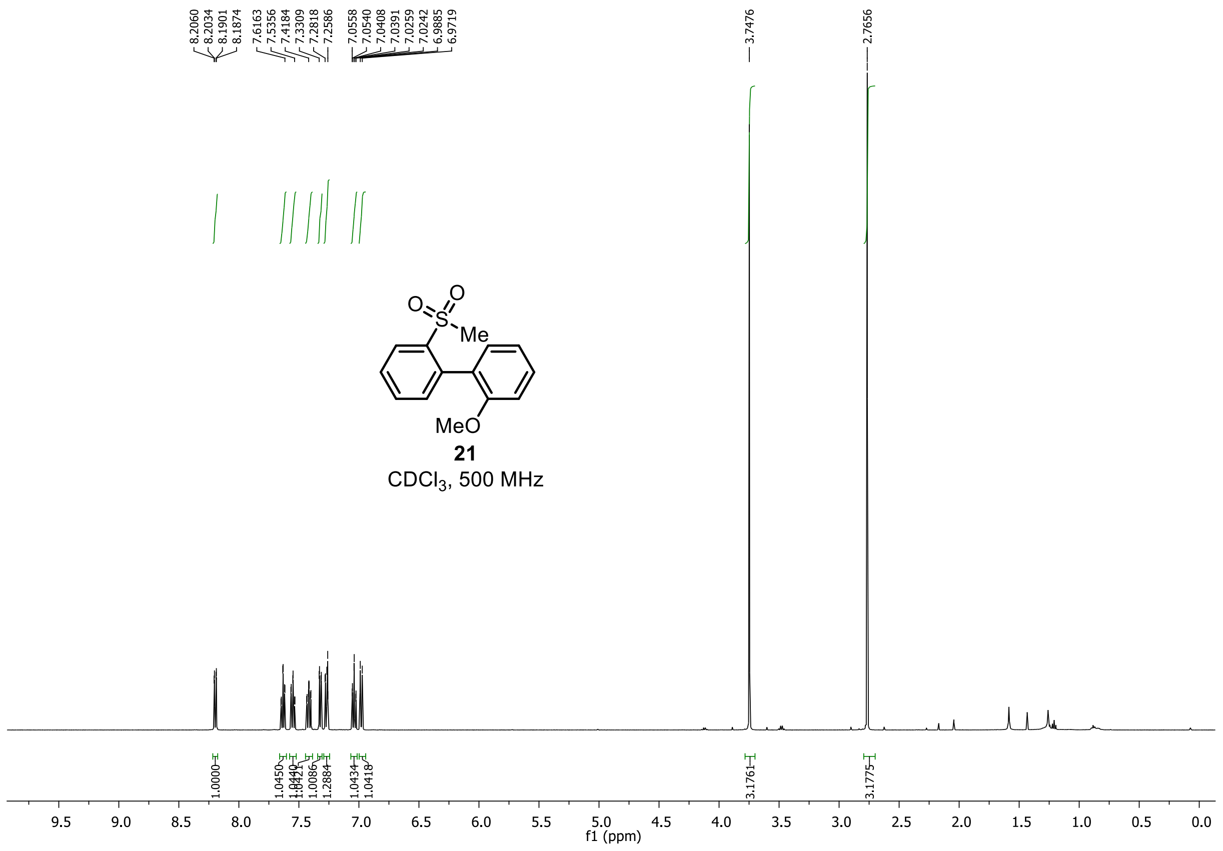

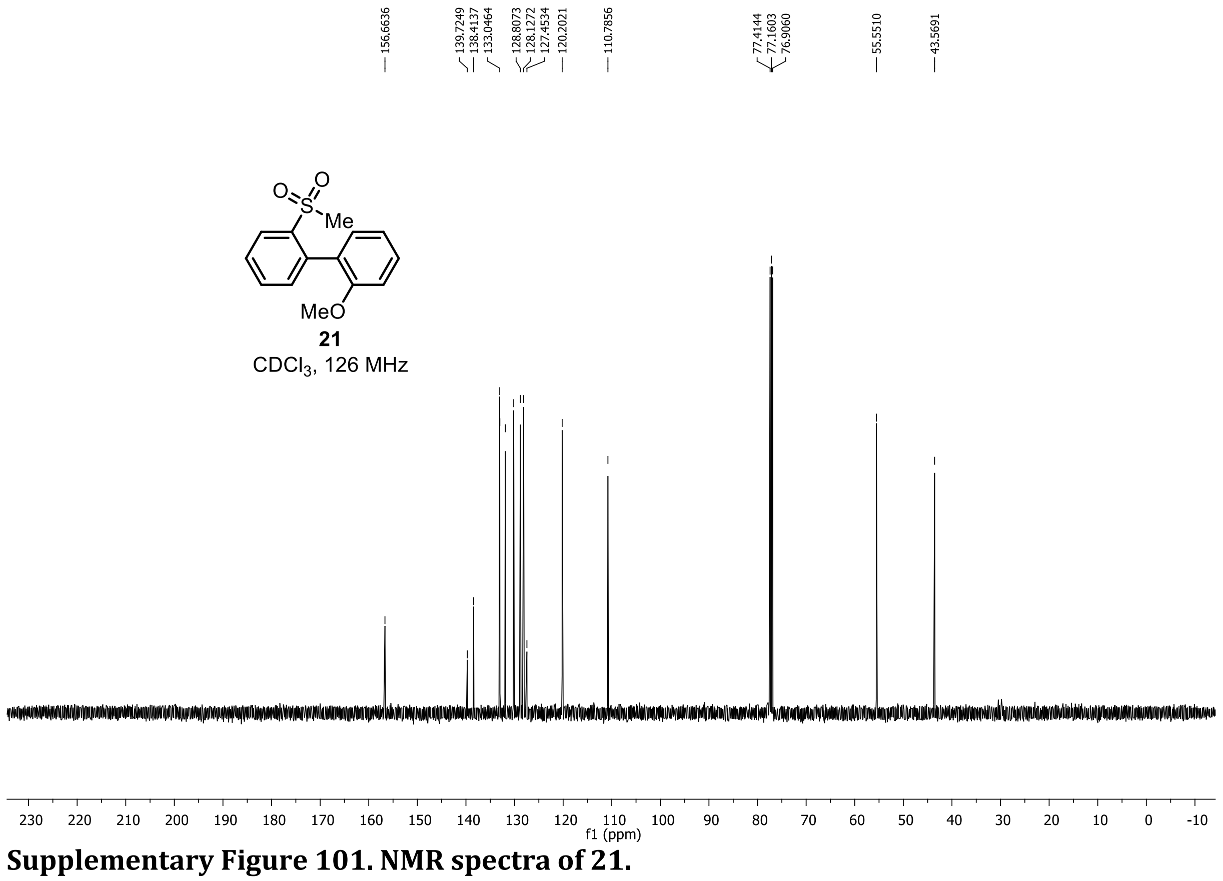

Supplementary Figure 101. NMR spectra of 21.

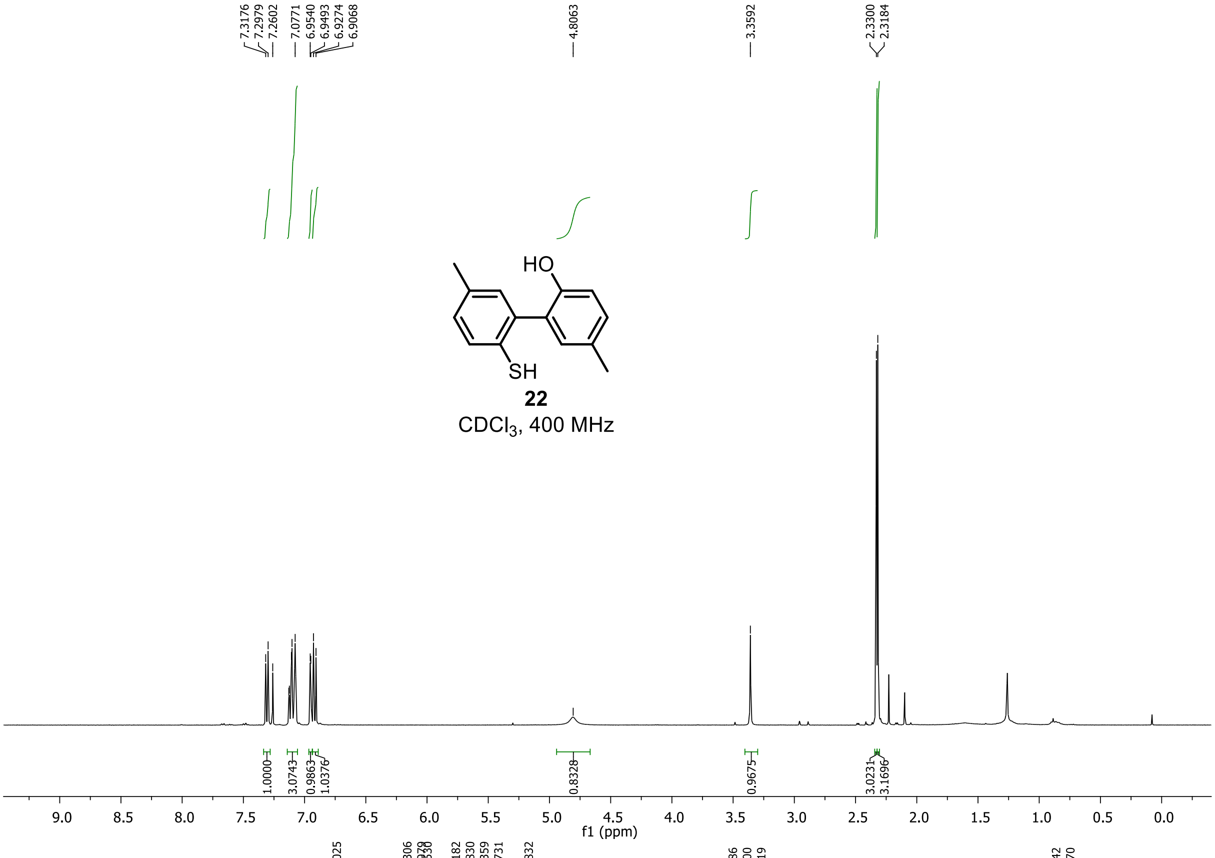

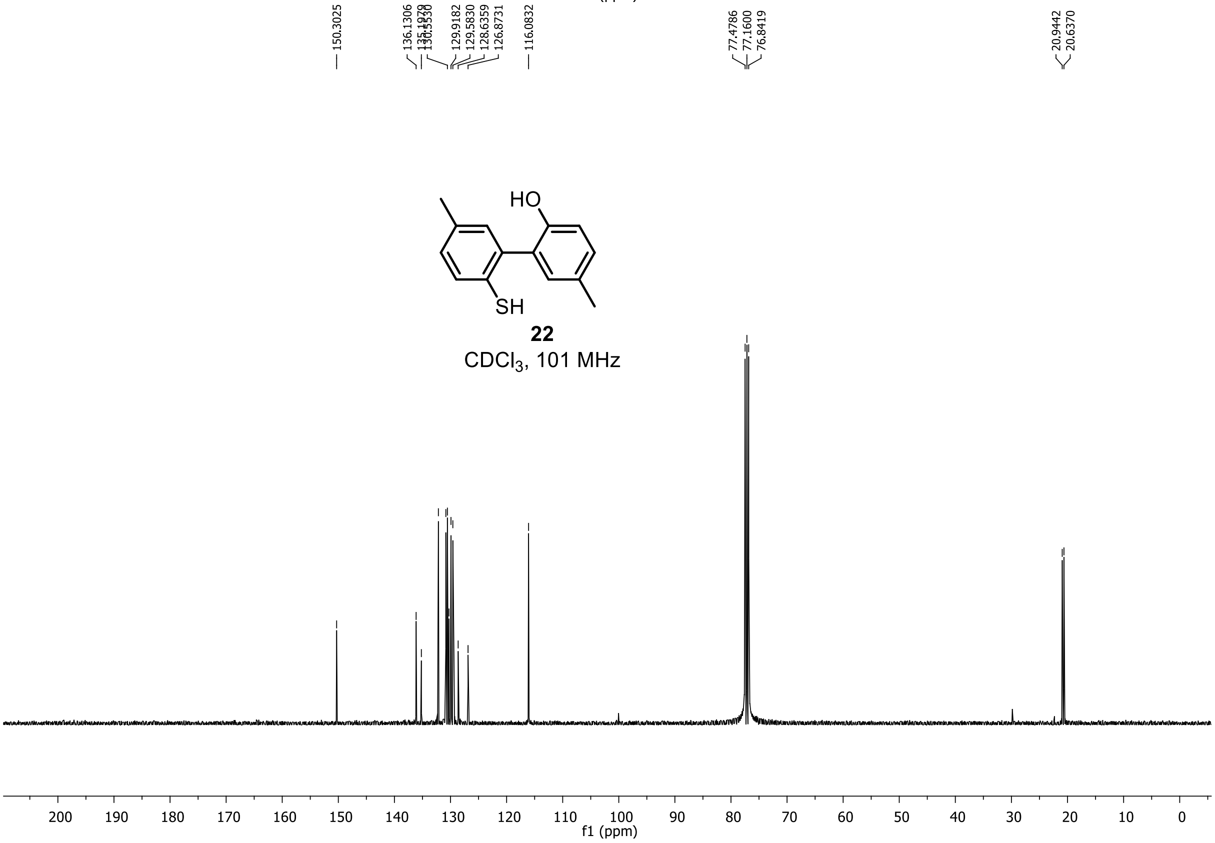

Supplementary Figure 102. NMR spectra of 22.

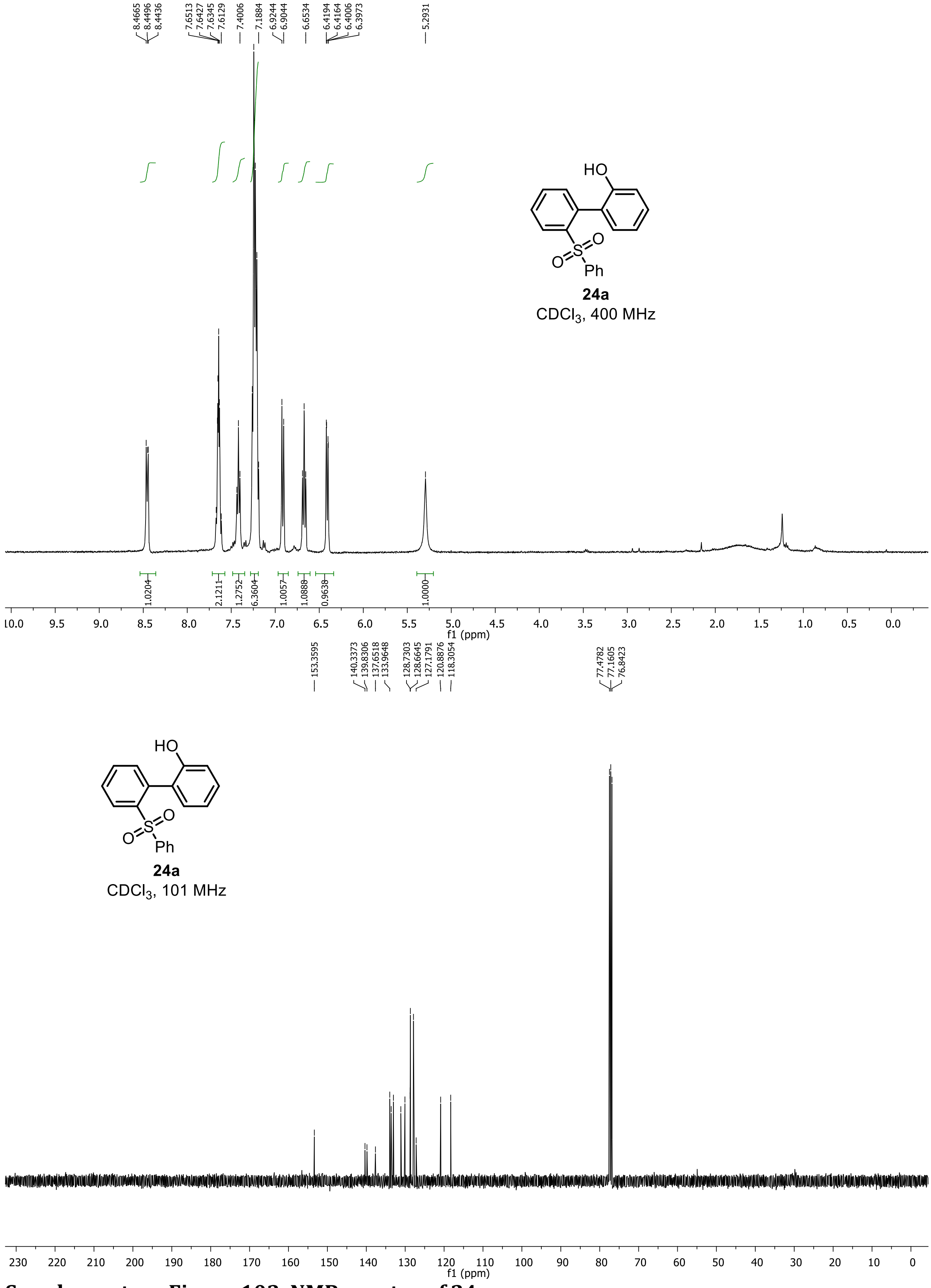

Supplementary Figure 103. NMR spectra of 24a.

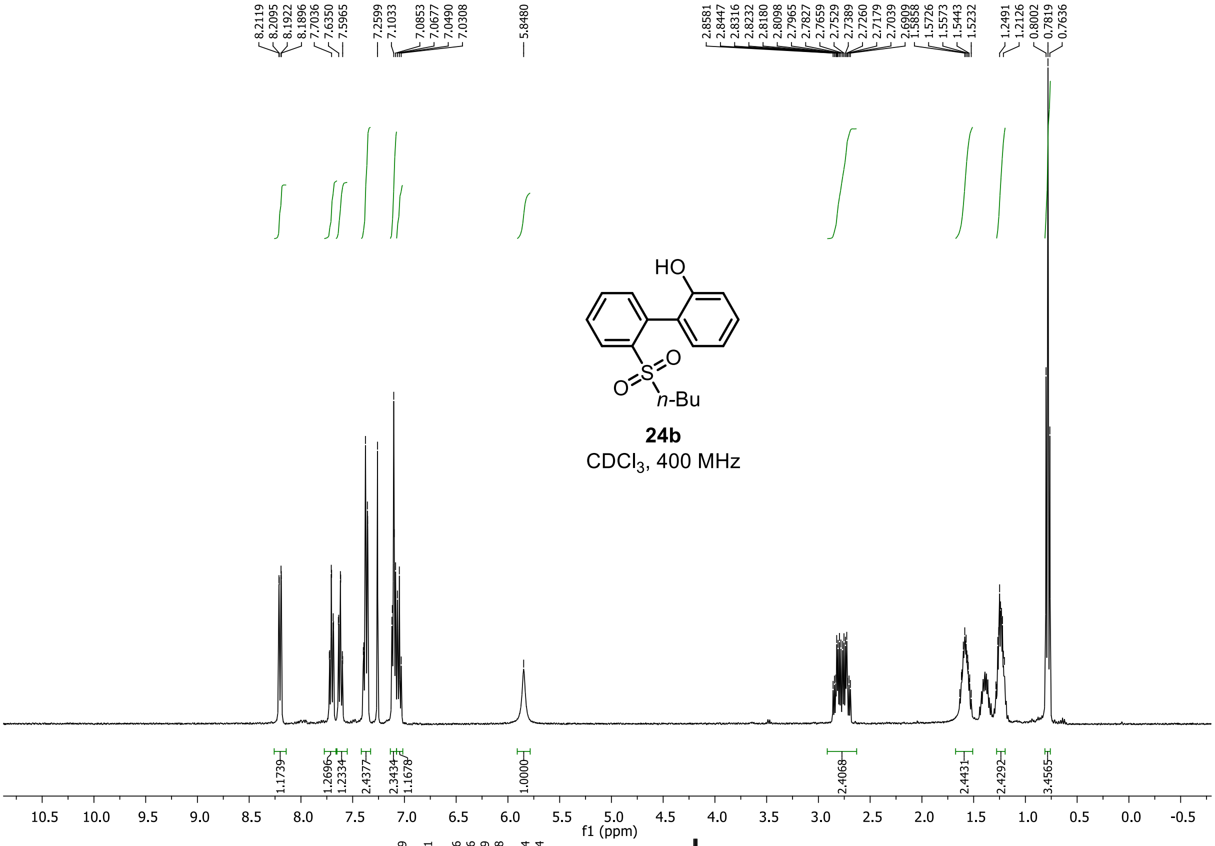

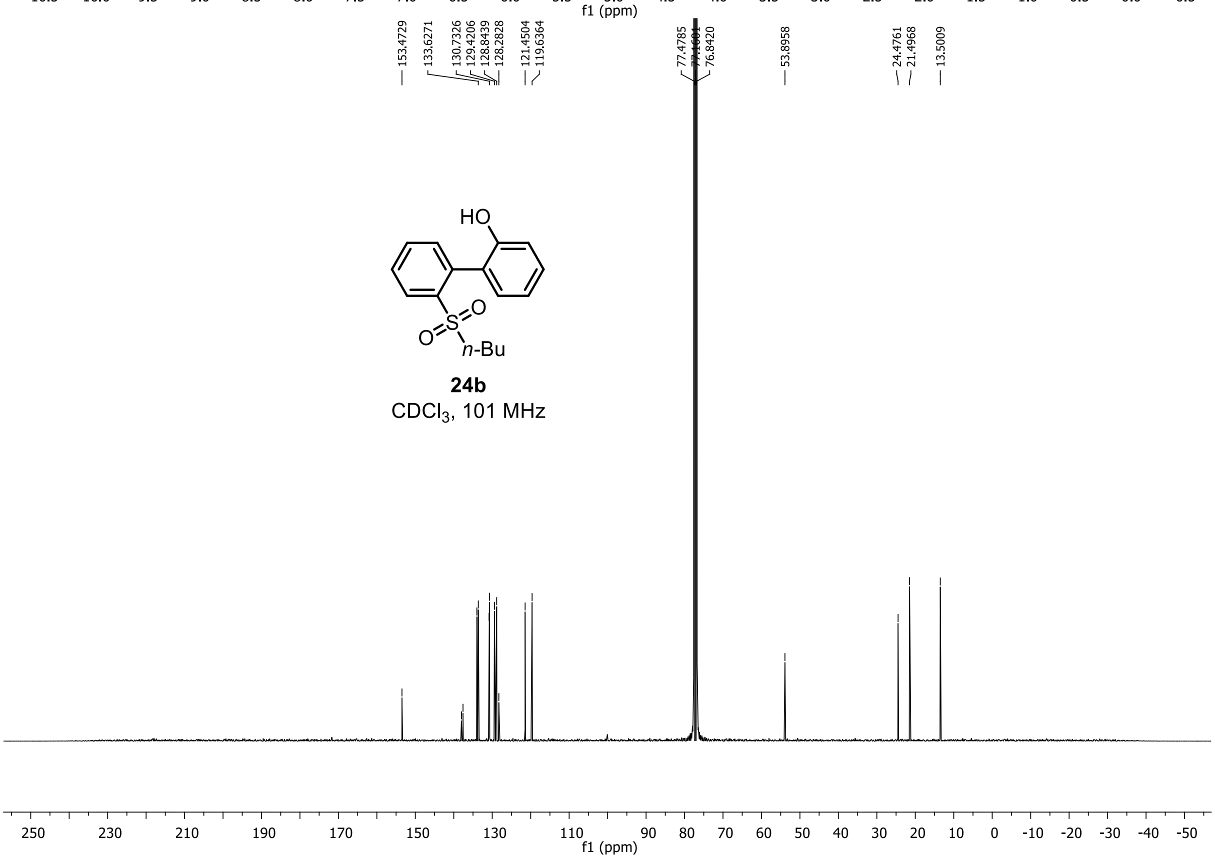

Supplementary Figure 104. NMR spectra of 24b.

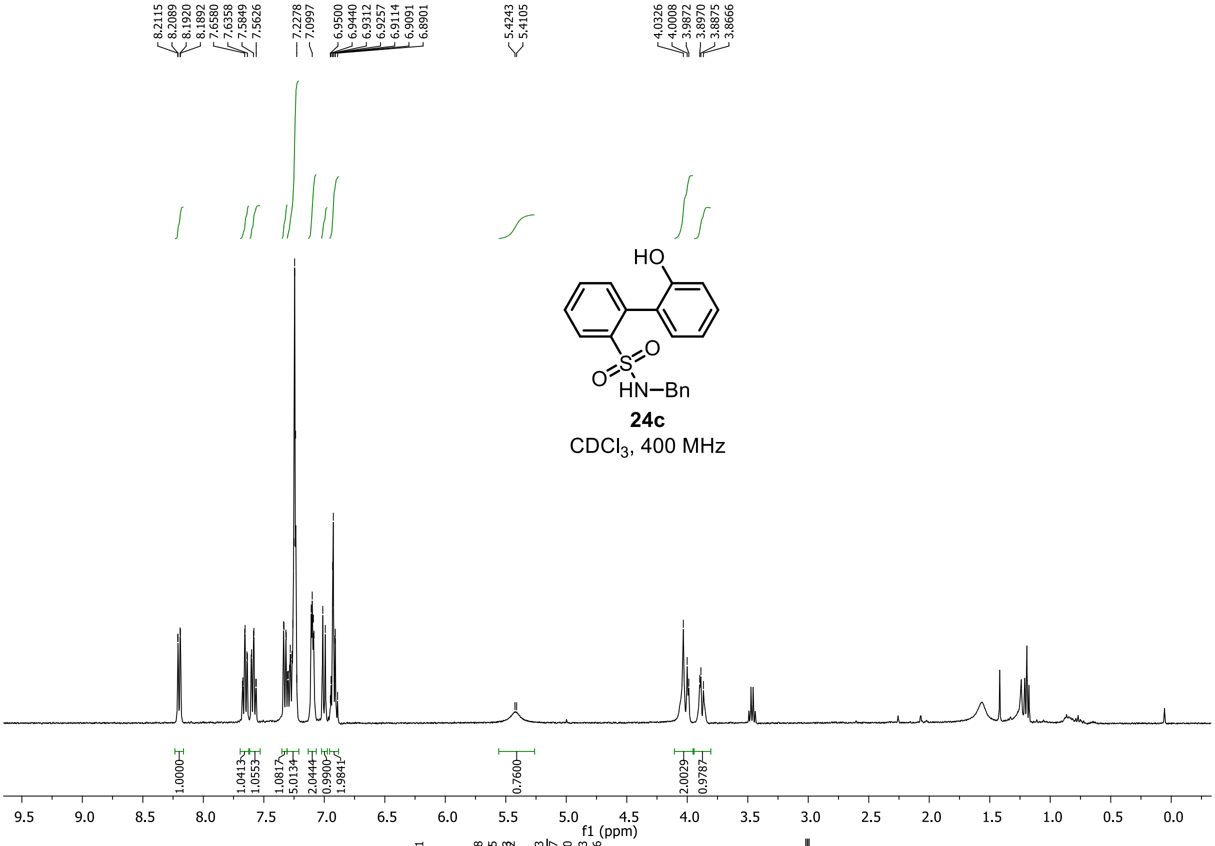

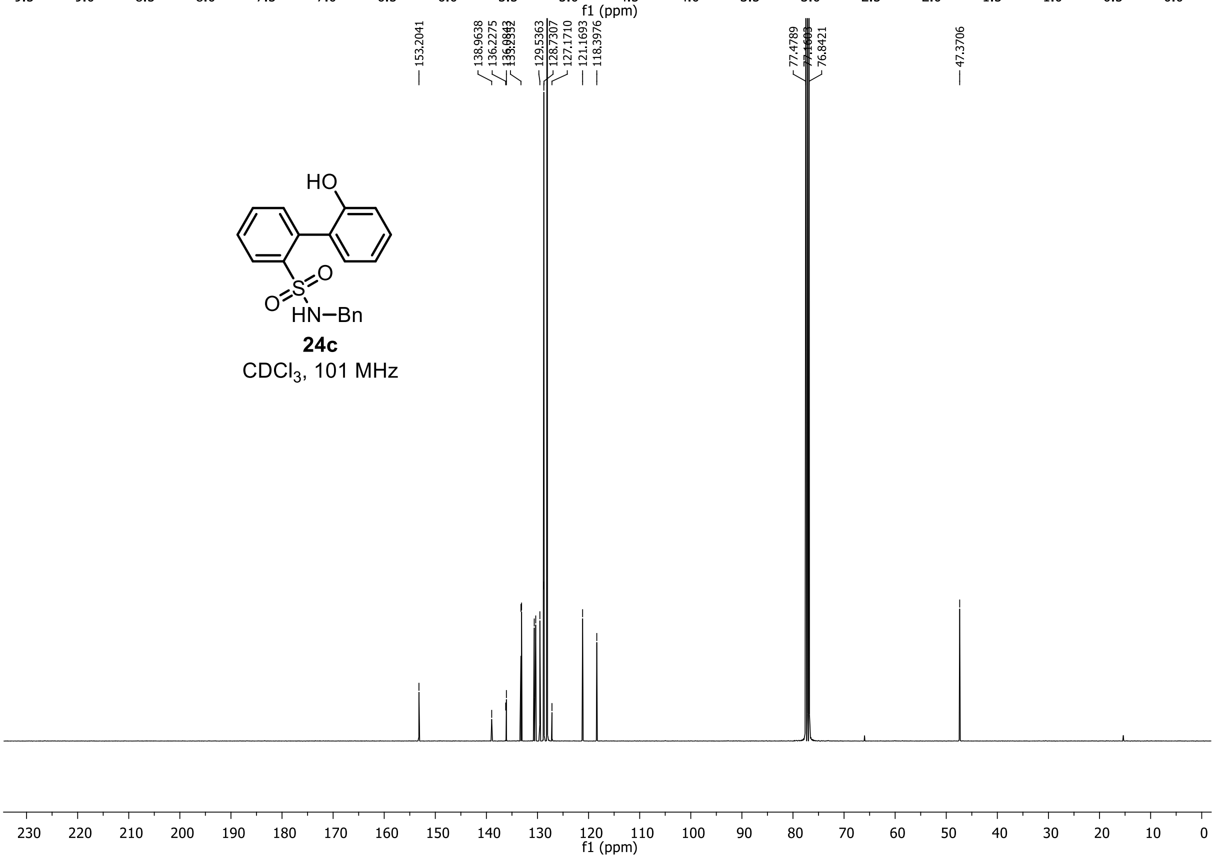

Supplementary Figure 105. NMR spectra of 24c.

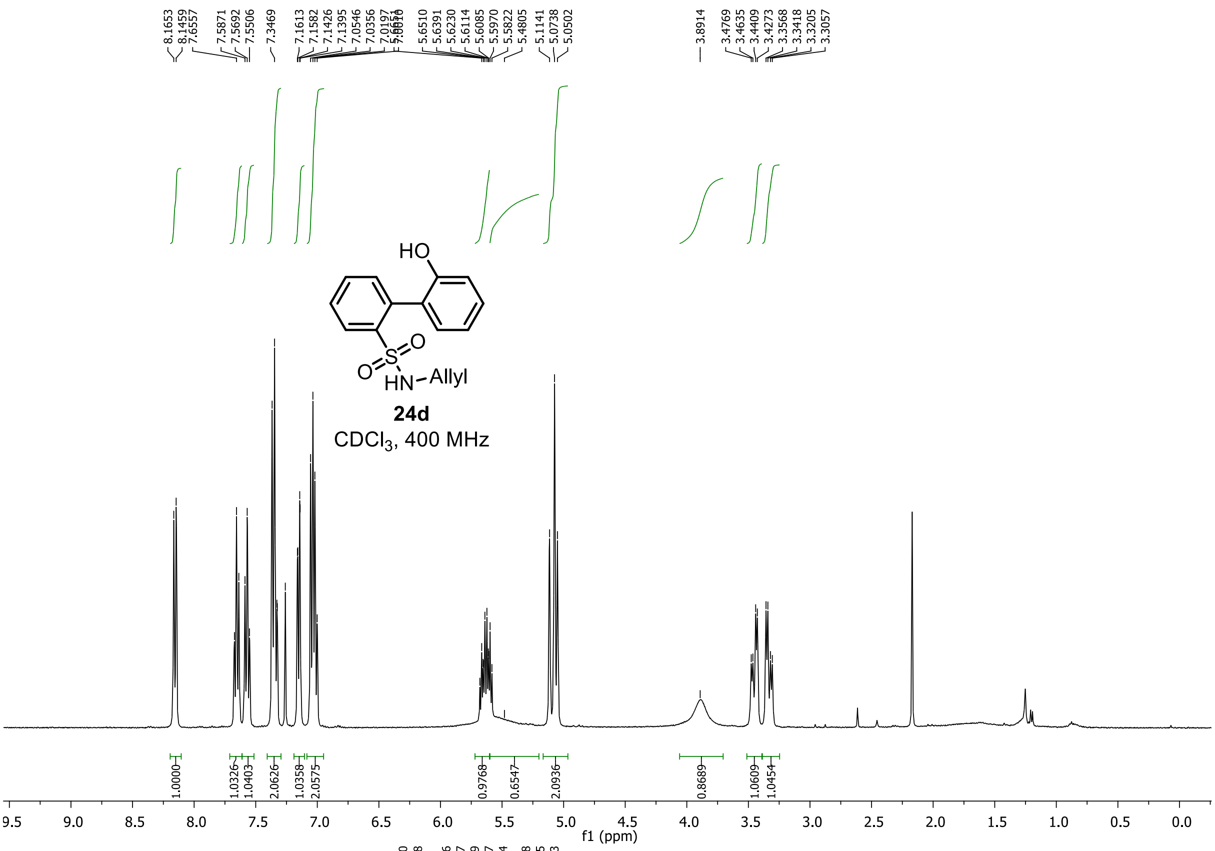

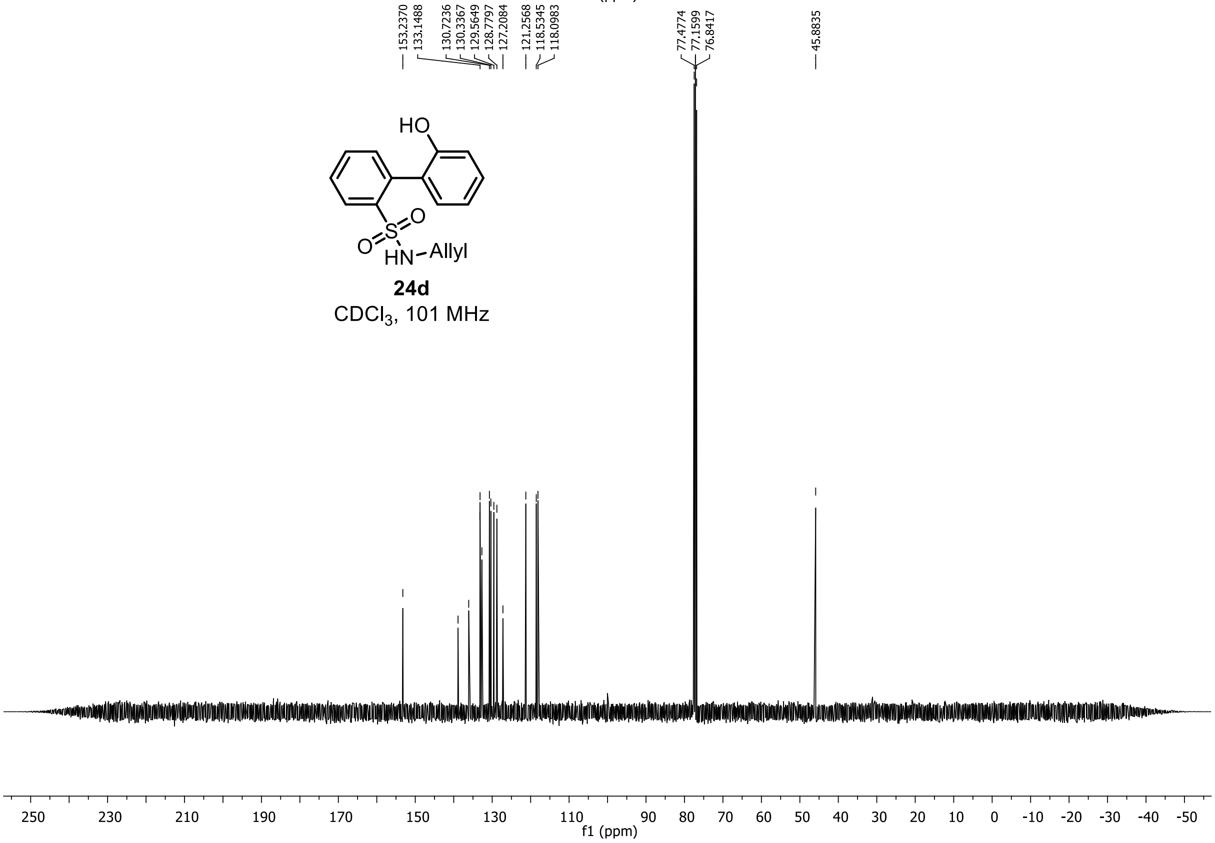

Supplementary Figure 106. NMR spectra of 24d.

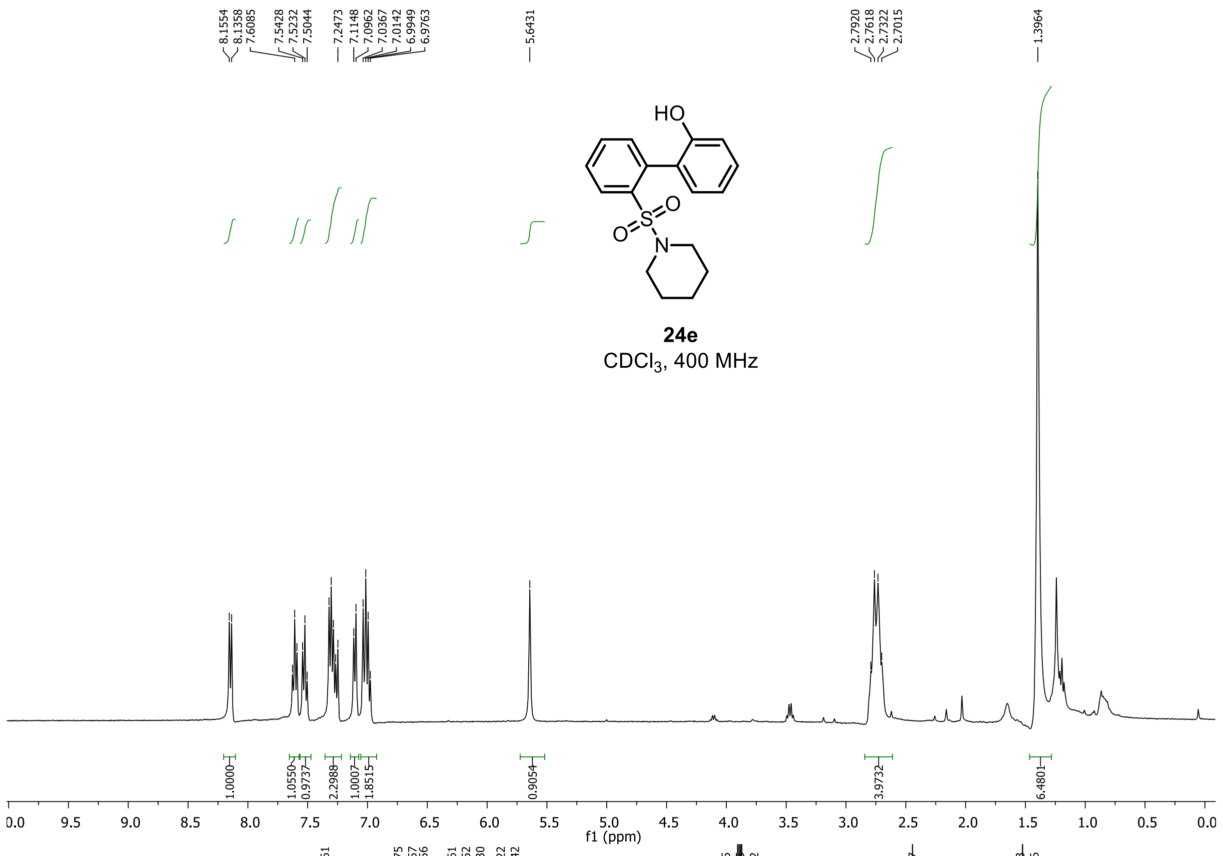

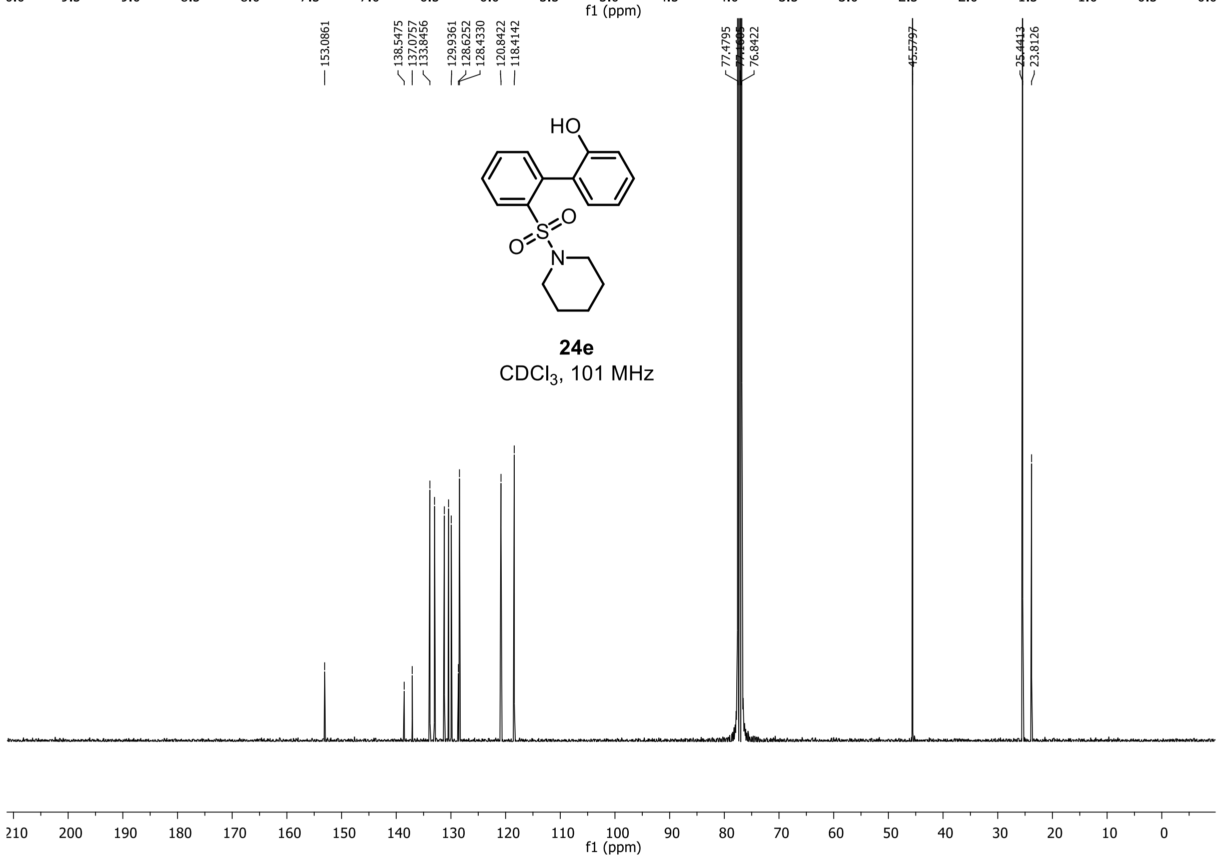

Supplementary Figure 107. NMR spectra of 24e.

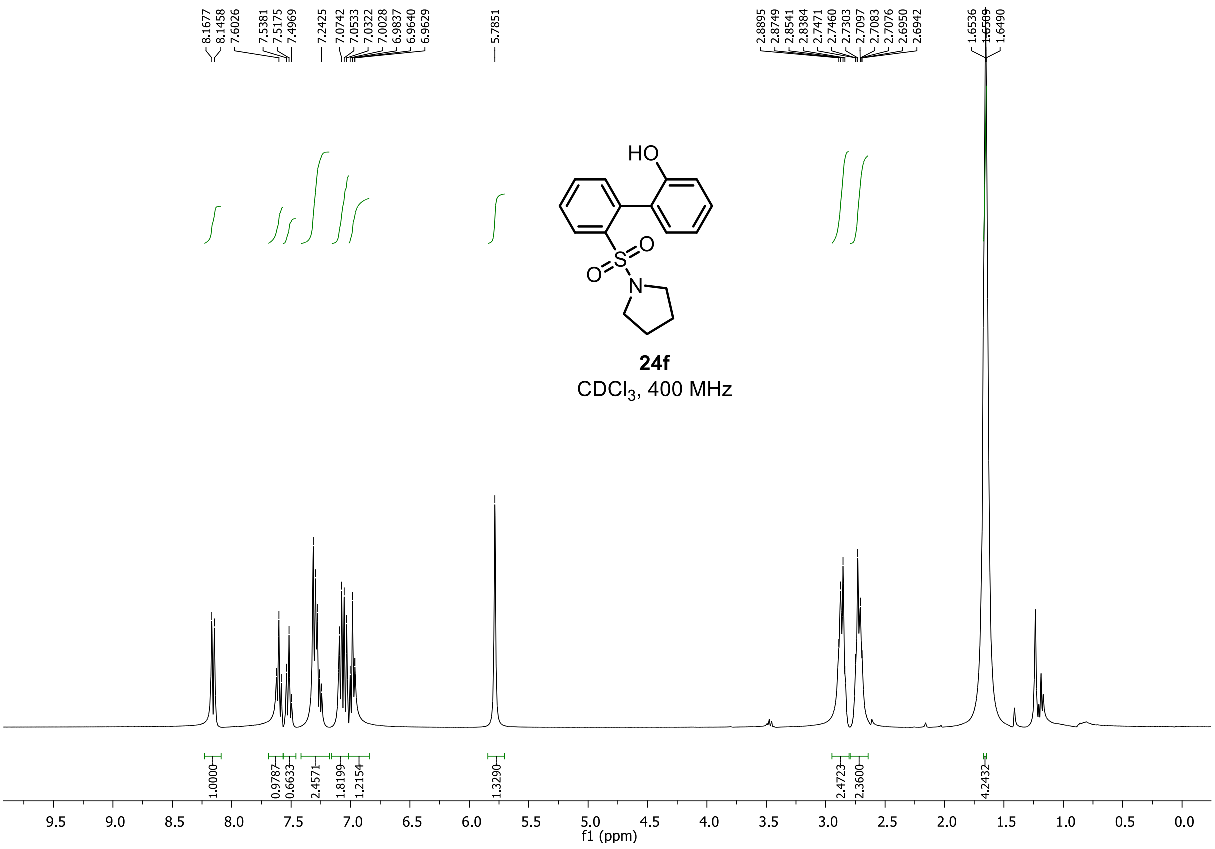

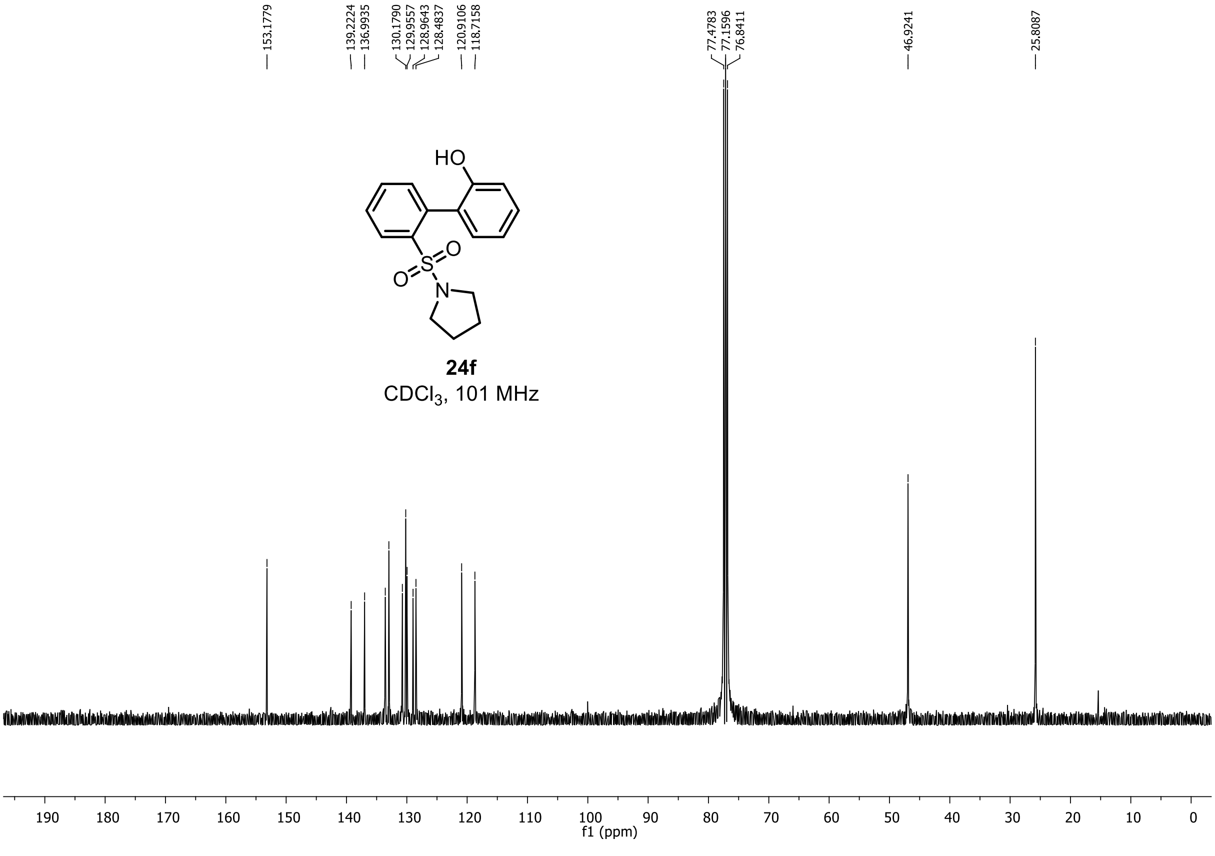

Supplementary Figure 108. NMR spectra of 24f.

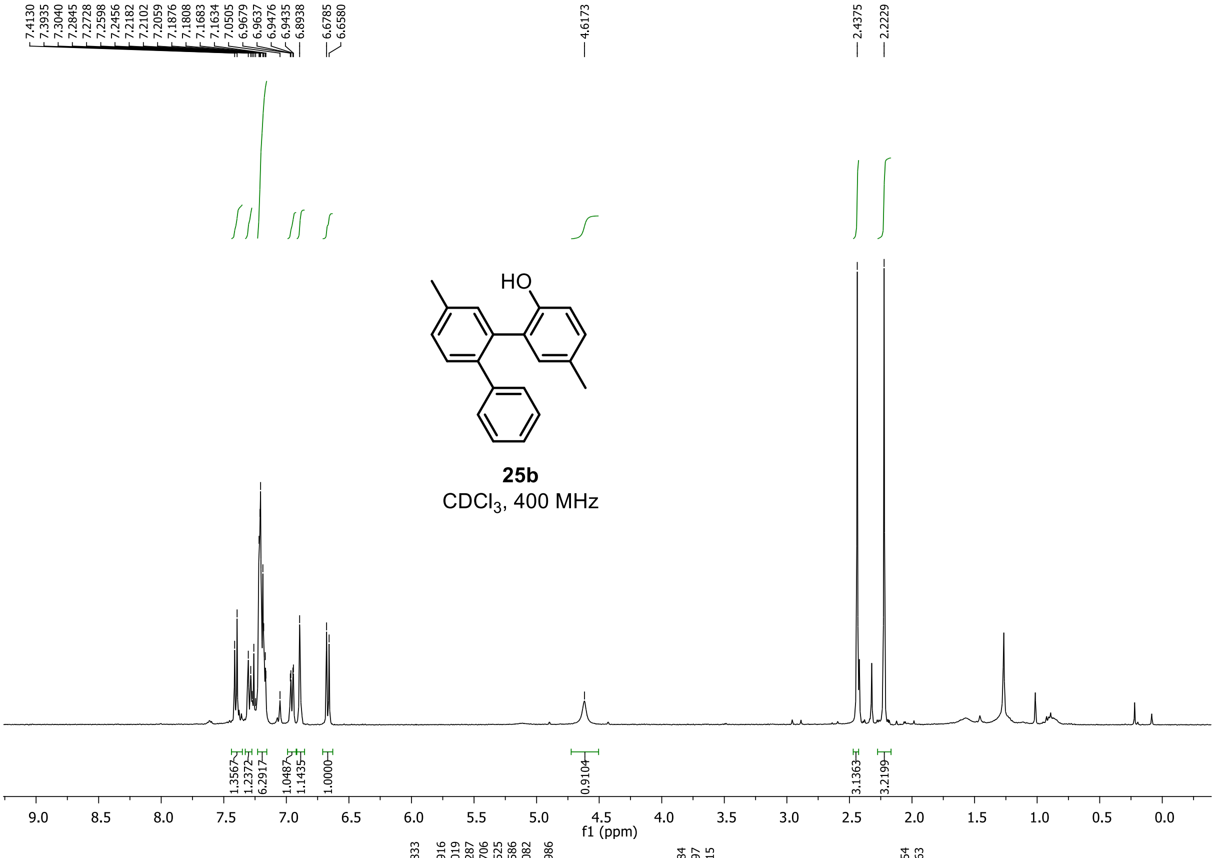

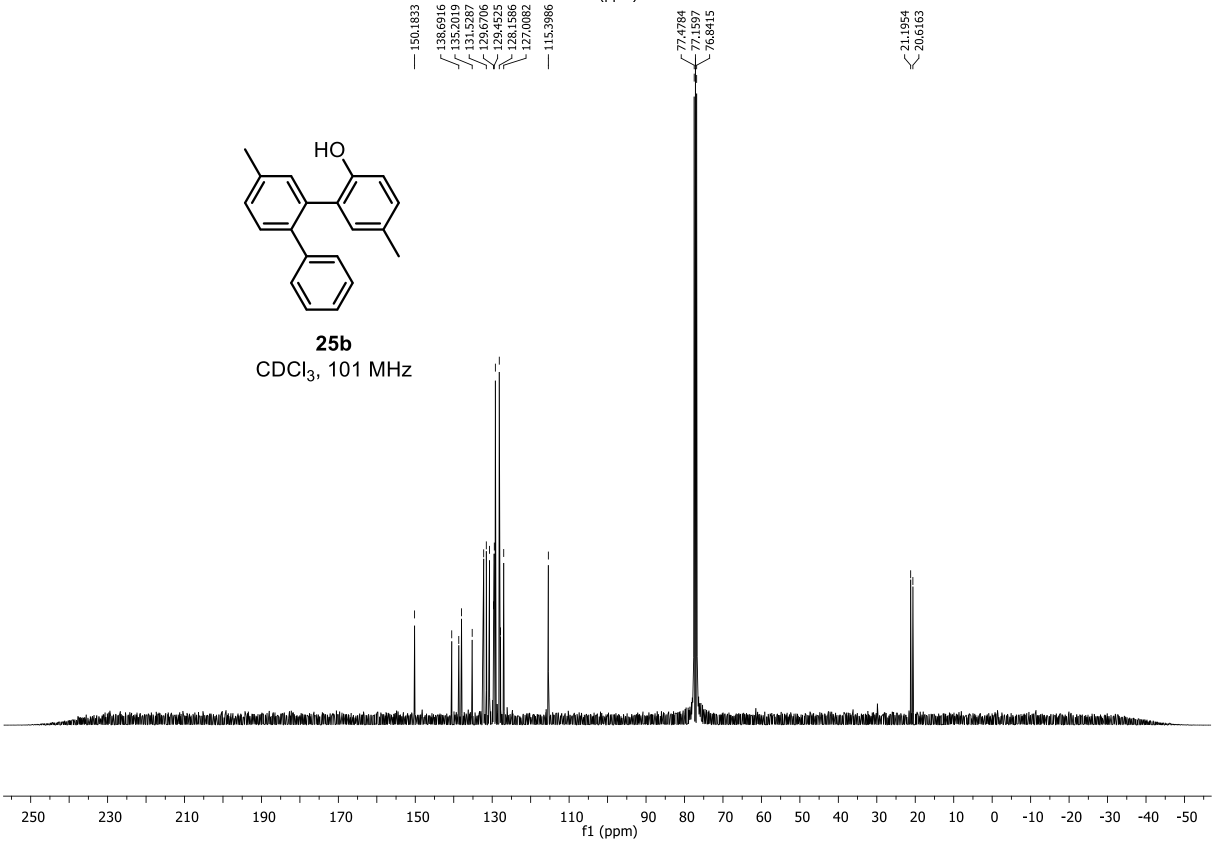

Supplementary Figure 109. NMR spectra of 25b.

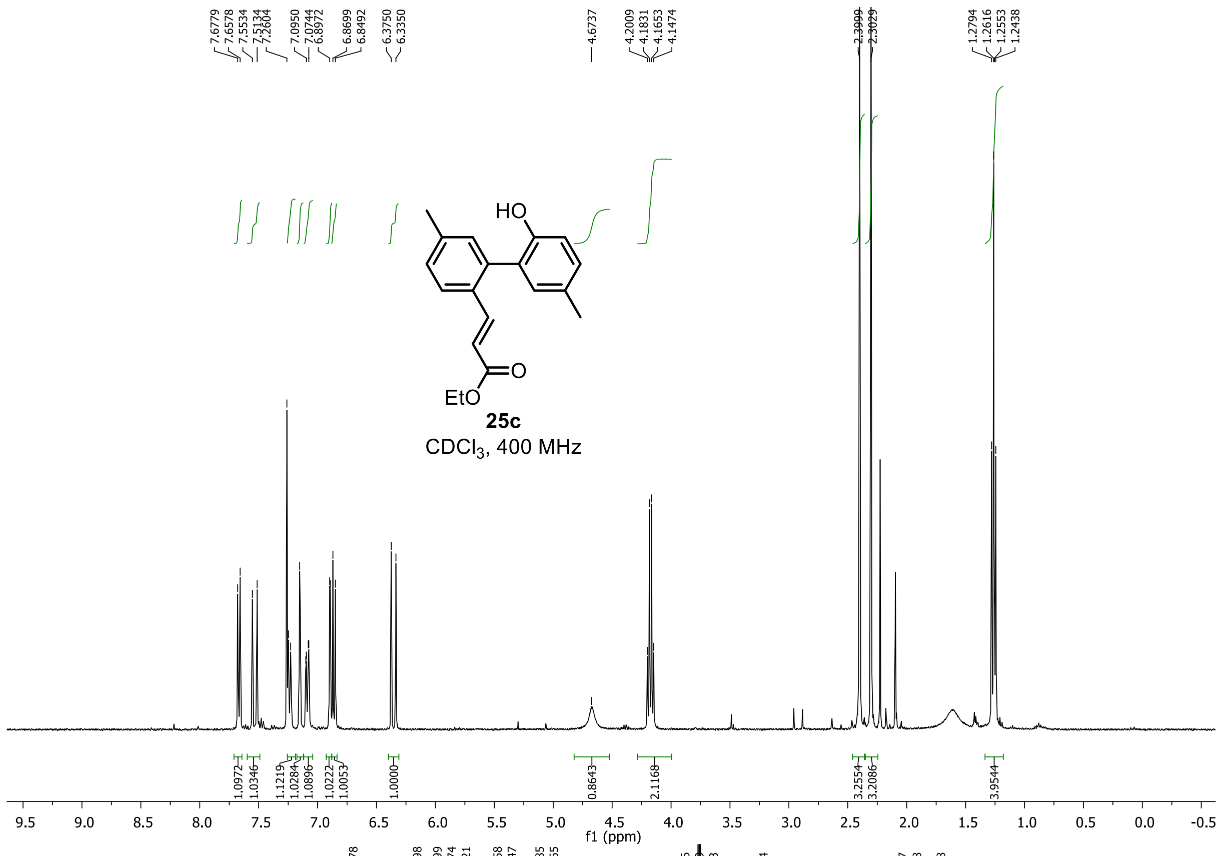

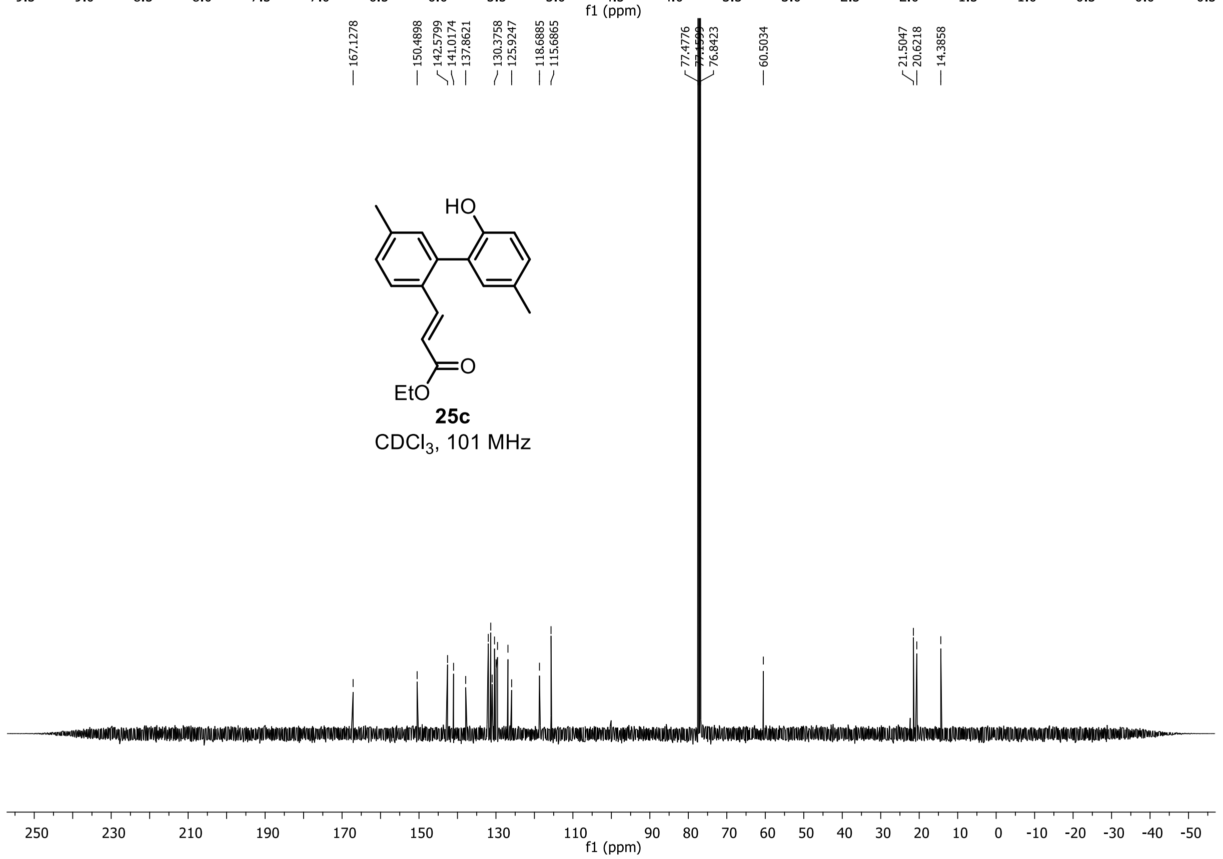

Supplementary Figure 110. NMR spectra of 25c.

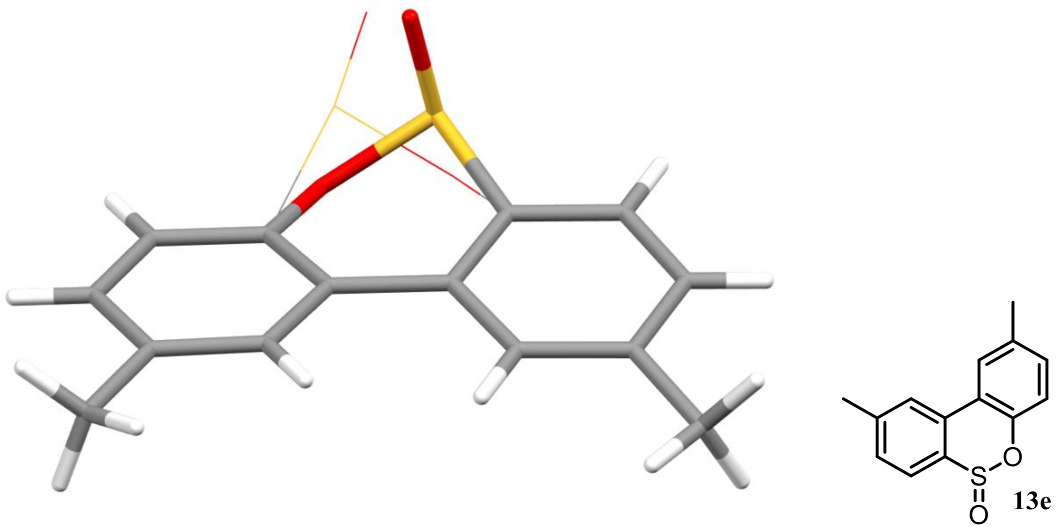

Supplementary Figure 111. Single crystal X-ray diffraction analysis of 13e. Please note that the structure itself present some disorder, main part is drawn in stick representation, minor disordered part is shown as thin lines (major and minor parts are each mirror image) ratio major/minor is about 0.8/0.2.

Supplementary References

1. Kuehm-Caubère, C., Adach-Becker, S., Fort, Y. & Caubère, P. Expeditious and efficient syntheses of pure 4-methyl and 4,6-disubstituted dibenzothiophenes. *Tetrahedron* **52**, 9087-9092 (1996).
2. Kawabata, K., Takeguchi, M. & Goto, H. Optical activity of heteroaromatic conjugated polymer films prepared by asymmetric electrochemical polymerization in cholesteric liquid crystals: structural function for chiral induction. *Macromolecules* **46**, 2078-2091 (2013).
3. Tedjamulia, M. L., Tominaga, Y. & Castle, R. N. The synthesis of all of the dimethyl dibenzothiophenes and monoethyl dibenzothiophenes. *J. Heterocyclic. Chem.* **20**, 1485-1495 (1983).
4. Yang, W. et al. Improvement of color purity in blue-emitting polyfluorene by copolymerization with dibenzothiophene. *J. Mater. Chem.* **13**, 1351-1355 (2003).
5. Patpi, S. R. et al. Design, synthesis, and structure-activity correlations of novel dibenzo[*b*,*d*]furan, dibenzo[*b*,*d*]thiophene, and *N*-methylcarbazole clubbed 1,2,3-triazoles as potent inhibitors of mycobacterium tuberculosis. *J. Med. Chem.* **55**, 3911-3922 (2012).
6. Boberg, F., Bruns, W. & Mußhoff, D. Schwefel verbindngen des erdöls xviii. Dialkyldibenzothiophene. *Phosphorus, Sulfur, and Silicon* **79**, 113-121 (1993).
7. Korang, J., Grither, W. R. & McCulla, R. D. Photodeoxygenation of dibenzothiophene S-oxide derivatives in aqueous media. *J. Am. Chem. Soc.* **132**, 4466-4476 (2010).
8. Louillat, M.-L., Biafora, A., Legros, F. & Patureau, F. W. Ruthenium-catalyzed cross-dehydrogenative ortho-*N*-carbazolation of diarylamines: versatile access to unsymmetrical diamines. *Angew. Chem. Int. Ed.* **53**, 3505-3509 (2014).
9. Che, R., Wu, Z.-Q., Li, Z.-K., Xiang, H.-F. & Zhou, X.-G. Synthesis of dibenzothiophenes by Pd-catalyzed dual C-H activation from diaryl sulfides. *Chem. Eur. J.* **20**, 7258-7261 (2014).
10. Kawanishi, Y. & Waka, K. Photosensitive composition, compound for use in the photosensitive composition, and pattern-forming method using the photosensitive composition. *US 2008/081288 A1* (2008).
11. Gregory, D. D., Wan, Z.& Jenks, W. S. Photodeoxygenation of dibenzothiophene sulfoxide: evidence for a unimolecular S-O cleavage mechanism. *J. Am. Chem. Soc.* **119**, 94-102 (1997).
12. Schoumacker, S., Hamelin, O., Téti, S., Pécaut, J. & Fontecave, M. Activation of oxaziridines by Lewis acids: Application in enantioselective sulfoxidation. *J. Org. Chem.* **70**, 301-308 (2005).
13. Bharadwaj, S. K., Sharma, S. N., Hussain, S. & Chaudhuri, M. K. Chemoselective sulfoxidation by H_2O_2 or HNO_3 using a phosphate impregnated titania catalyst. *Tetrahedron Lett.* **50**, 3767-3771 (2009).
14. Zhang, M., Welch, B. K., Hasanagic, M., Fritz, A. & McCulla, R. D. Determination of sulfoxide bond dissociation enthalpies of dibenzothiophene S-oxide derivatives with computational methods. *J. Phys. Org. Chem.* **27**, 630-639 (2014).

15. Sun, Z.-C., She, Y.-B., Zhou, Y., Song, X.-F. & Li, K. Synthesis, characterization and spectral properties of substituted tetraphenylporphyrin iron chloride complexes. *Molecules* **16**, 2960-2970 (2011).
16. Wang, L.-Z. et al. A green process for oxidation of p-nitrotoluene catalyzed by metalloporphyrins under mild conditions.*Org. Process. Res. Dev.***10**, 757-761 (2006).
17. Ormond, A. B. & Freeman, H. S. Effects of substituents on the photophysical properties of symmetrical porphyrins. *Dyes and Pigments*, **96**, 440-448 (2013).
18. Crich, D., Hutton, T. K. & Ranganathan, K. Is there a homolytic substitution chemistry (S_H2) of sulfones? *J. Org. Chem.* **70**, 7672-7678 (2005).
19. Yang, Q., Ma, S., Li, J., Xiao, F. & Xiong, H. A water-compatible, highly active and reusable PEG-coated mesoporous silica-supported palladium complex and its application in Suzuki coupling reactions. *Chem. Commun.* 2495-2497 (2006).
20. Squires, T. G. et al. Preparation, characterization, and flash vacuum pyrolysis of dibenz [*c*,*e*][1,2] oxathiin 6-oxide (biphenylene sultine). *J. Org. Chem.* **46**, 2373-2376 (1981).
21. Duan, S., Xu, Y., Zhang, X. & Fan, X. Synthesis of 2, 2′-biphenols through direct C(sp^2)-H hydroxylation of [1,1′-biphenyl]-2-ols. *Chem. Commun.* **52**, 10529-10532 (2016).
22. Kohn, W. Nobel Lecture: Electronic structure of matter-wave functions and density functionals. *Rev. Mod. Phys.***71**, 1253-1266 (1999).
23. Frisch, M. J.et. al. In Gaussian, Inc.Wallingford CT. [Online] (2009).
24. Lee, C., Yang, W.& Parr, R. G. Development of the Colle-Salvetti correlation-energy formula into a functional of the electron density. *Phys Rev B: Condens Matter* **37**, 785-789 (1988).
25. Dolg, M., Stoll, H., Savin, A. & Preuss, H. Energy-adjusted pseudopotentials for the rare earth elements. *Theoretica Chimica Acta***75**, 173-194 (1989).
26. McLean, A. D. & Chandler, G. S. Contracted Gaussian basis sets for molecular calculations. I. Second row atoms, Z=11-18. *J. Chem. Phys.***72**, 5639-5648 (1980).
27. Cances, E., Mennucci, B. & Tomasi, J. A new integral equation formalism for the polarizable continuum model: Theoretical background and applications to isotropic and anisotropic dielectrics. *J. Chem. Phys.***107**, 3032-3041 (1997).
