# Supplementary material for: Post-functionalization of dibenzothiophene to functionalized biphenyls via a photoinduced thia-Baeyer-Villiger oxidation
Source: Nat Commun. 2020 Feb 14;11:914. doi: 10.1038/s41467-020-14522-7 (PMC7021910; doi:10.1038/s41467-020-14522-7)
Supplement: Supplementary file 2 — Description of Additional Supplementary Files [file 41467_2020_14522_MOESM2_ESM.pdf]

Description of Additional Supplementary Files

File Name: Supplementary Data 1

Description: Coordinates and energies of DFT-computed stationary points.
