# Supplementary material for: Post-functionalization of dibenzothiophene to functionalized biphenyls via a photoinduced thia-Baeyer-Villiger oxidation
Source: Nat Commun. 2020 Feb 14;11:914. doi: 10.1038/s41467-020-14522-7 (PMC7021910; doi:10.1038/s41467-020-14522-7)
Supplement: Supplementary file 3 — Supplementary Data 1 [file 41467_2020_14522_MOESM3_ESM.pdf]

The coordinate information for the molecules in Supplementary Figure 15

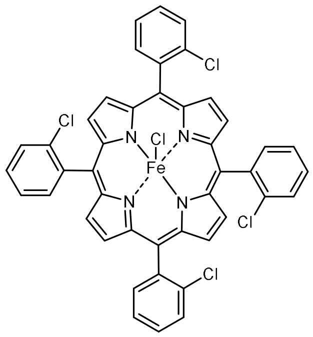

[TCPPFe]Cl G = -4334.685603 Hartree

N	0.00042200	-1.89850300	-0.57391700
C	1.09658000	-2.67339500	-0.89786600
C	-1.11364500	-2.66830200	-0.84420400
C	2.43855600	-2.28127300	-0.79033100
C	0.65741000	-3.95842100	-1.38138000
C	-2.44727500	-2.26605700	-0.68314800
C	-0.70437500	-3.95597300	-1.34627400
N	0.04224400	1.93585300	0.79675200
C	2.86965600	-1.01973900	-0.35572200
C	-2.85160600	-1.00256100	-0.22896700
C	1.15545100	2.71809300	1.03164000
C	-1.05488400	2.73017700	1.06494200
N	2.05464400	0.01382900	0.06240300
C	4.24948100	-0.60798700	-0.28890700
N	-2.01279900	0.02772900	0.14789600
C	-4.22456500	-0.57936900	-0.11040600
C	2.48859500	2.32240300	0.85202800
C	0.74495600	4.03015700	1.46545100
C	-2.39713500	2.34462800	0.93902300
C	-0.61703400	4.03846500	1.48342200
C	2.89262600	1.06102400	0.39152600
C	4.26374800	0.67322400	0.17328500

C	-2.82868100	1.08546600	0.49768300
C	-4.21035000	0.70804700	0.33413400
C	-5.42243700	5.31691000	1.76697200
C	-4.99026600	4.47594300	2.79264800
C	-4.01293200	3.51629200	2.52594400
Cl	-3.49217600	2.47290600	3.84976800
C	-3.44754100	3.36984900	1.24993800
C	-3.90192700	4.23081800	0.23765500
C	-4.87776700	5.19522500	0.48720200
C	5.58039400	5.16206700	1.87599200
C	5.07575900	5.13907000	0.57563400
C	4.07705400	4.22244000	0.24511200
Cl	3.46514900	4.22421300	-1.41029300
C	3.56092600	3.31725800	1.18540300
C	4.08807800	3.36417800	2.48617600
C	5.08637100	4.27396400	2.83330400
C	5.45715500	-5.10088800	-2.05848300
C	4.89002600	-4.21047400	-2.97212300
C	3.91663000	-3.30666600	-2.54713200
C	3.48744800	-3.26816100	-1.21051900
C	4.07513300	-4.17556500	-0.31563300
Cl	3.58635100	-4.18906800	1.37937100
C	5.05034400	-5.08611300	-0.72421200
C	-5.53539000	-5.15859600	-1.55940500
C	-5.00111300	-5.05731900	-0.27352800
C	-4.00486500	-4.11830100	-0.00814000
C	-3.51935200	-3.26312800	-1.01070400
C	-4.07543700	-3.38838700	-2.29315500
Cl	-3.51792900	-2.34797600	-3.60520700

C	-5.07282600	-4.32233100	-2.57572100
H	1.30900900	-4.75438400	-1.71193400
H	-1.37500200	-4.75022200	-1.64094800
H	5.09525300	-1.21998200	-0.56731600
H	-5.08570700	-1.18786800	-0.34610400
H	1.41464800	4.84054700	1.71486100
H	-1.26986800	4.85754000	1.74848700
H	5.12347900	1.30439600	0.34597100
H	-5.05761400	1.34967800	0.52865300
H	-6.18307300	6.06397200	1.97298700
H	-5.40434600	4.55993900	3.79159700
H	-3.47565500	4.13224400	-0.75643600
H	-5.21009300	5.84730100	-0.31470800
H	6.35775000	5.87463200	2.13499100
H	5.44997300	5.82343300	-0.17807700
H	3.70121600	2.67125800	3.22766500
H	5.47548200	4.28787400	3.84675000
H	6.21587600	-5.80893900	-2.37804200
H	5.20290700	-4.21796100	-4.01170600
H	3.47295700	-2.61209700	-3.25452700
H	5.48218200	-5.77254900	-0.00393100
H	-6.31168500	-5.88585500	-1.77752100
H	-5.35724400	-5.70578500	0.52104300
H	-3.58637600	-4.03598000	0.99069800
H	-5.47904000	-4.39016500	-3.57910400
Fe	0.03034900	-0.13645900	0.54098400
Cl	0.07582800	-0.90084200	2.69974800

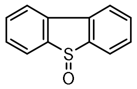

DBTO G = -935.343299 Hartree

C	0.81298300	-0.02070200	0.73562600
C	1.89793600	0.08106300	1.61106000
C	1.66528500	0.09533400	2.98903100
C	0.36697000	0.00109900	3.50205900
C	-0.72561300	-0.12340000	2.63672200
C	-0.48224100	-0.12965300	1.26954700
S	-1.76021600	-0.34968900	0.00000000
O	-2.75440500	0.80630000	0.00000000
C	-0.48224100	-0.12965300	-1.26954700
C	0.81298300	-0.02070200	-0.73562600
C	1.89793600	0.08106300	-1.61106000
C	1.66528500	0.09533400	-2.98903100
C	0.36697000	0.00109900	-3.50205900
C	-0.72561300	-0.12340000	-2.63672200
H	2.91199100	0.15477600	1.22918700
H	2.50649700	0.18191100	3.67049600
H	0.20495900	0.01999700	4.57549300
H	-1.73600800	-0.20681200	3.02653500
H	2.91199100	0.15477600	-1.22918700
H	2.50649700	0.18191100	-3.67049600
H	0.20495900	0.01999700	-4.57549300
H	-1.73600800	-0.20681200	-3.02653500

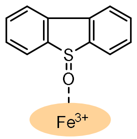

I G = -5269.999726 Hartree

N	-0.51300400	-1.93909200	-0.73959400
C	-1.76700700	-2.50101100	-0.64404200

C	0.39389800	-2.97271300	-0.81497500
C	-2.98565800	-1.80007600	-0.57434700
C	-1.64216700	-3.94012100	-0.64707600
C	1.79022300	-2.84118600	-0.93096600
C	-0.31411300	-4.22973700	-0.75591800
N	0.33768500	2.08822400	-1.00759100
C	-3.12664000	-0.39931200	-0.61351300
C	2.49036700	-1.62236200	-1.00859100
C	-0.57302500	3.11697500	-0.97715200
C	1.59046700	2.64813600	-1.08917800
N	-2.10593700	0.50868600	-0.75961500
C	-4.38154300	0.31086800	-0.50548600
N	1.92574600	-0.36901500	-1.01268700
C	3.92871300	-1.49428000	-1.07809200
C	-1.96904000	2.98122900	-0.85776800
C	0.13266000	4.37597700	-1.05569600
C	2.80924500	1.94514700	-1.12839600
C	1.46374700	4.08773100	-1.12089500
C	-2.66664100	1.76397600	-0.74954400
C	-4.09857800	1.64168400	-0.59319600
C	2.95039500	0.54512300	-1.08586400
C	4.21166500	-0.16038100	-1.12113600
C	6.44529400	4.28422900	-1.20547700
C	5.84350000	3.91515900	-2.40859000
C	4.66957300	3.16076200	-2.38448800
Cl	3.94298700	2.71285400	-3.92903300
C	4.06980500	2.75854400	-1.18106200
C	4.69948400	3.14536900	0.01351900
C	5.87306600	3.89876500	0.00831800

C	-4.31242100	6.61441600	-0.92465600
C	-3.85012300	6.11998900	0.29515200
C	-3.09509500	4.94655900	0.31642200
Cl	-2.53116000	4.35458800	1.88102800
C	-2.78267800	4.24241400	-0.85696500
C	-3.26302400	4.76480100	-2.06903400
C	-4.01897400	5.93616000	-2.10916700
C	-6.58189100	-4.15063600	-0.07511800
C	-5.78053100	-3.83557800	1.02375800
C	-4.62332100	-3.07859000	0.84365400
C	-4.23655000	-2.61584900	-0.42521400
C	-5.06002400	-2.95497300	-1.51088000
Cl	-4.63420100	-2.43694600	-3.14309900
C	-6.22166000	-3.71131700	-1.34910400
C	4.10799500	-6.47614500	-1.27302700
C	3.67828100	-5.79465500	-2.41328200
C	2.93284500	-4.62354000	-2.28179500
C	2.59727400	-4.10349000	-1.02051800
C	3.04592200	-4.81047200	0.10595700
Cl	2.67846700	-4.21890900	1.72809200
C	3.79254600	-5.98416300	-0.00648200
Fe	-0.10109200	0.06961900	-1.04568500
Cl	-0.28073300	-0.06313800	-3.42487500
C	-0.23967600	-0.08191700	4.82599600
C	-0.98337400	0.19690000	5.97558600
C	-2.37928700	0.21185100	5.90005200
C	-3.04055300	-0.05287300	4.69573300
C	-2.31026400	-0.35370600	3.54026600
C	-0.92452600	-0.35986200	3.63083300

S	0.18956500	-0.76573400	2.27037000
O	0.08847700	0.35991700	1.20531000
C	1.60349000	-0.42271900	3.34027000
C	1.22098000	-0.11787200	4.65808700
C	2.21756200	0.11677200	5.60880200
C	3.55941600	0.06164600	5.22025300
C	3.91767400	-0.23275400	3.90023800
C	2.93207900	-0.49101600	2.94023100
H	-2.46490200	-4.63753800	-0.58111700
H	0.14566000	-5.20699100	-0.79555200
H	-5.35050100	-0.15021300	-0.37651700
H	4.62884300	-2.31748700	-1.08821900
H	-0.33096300	5.35218200	-1.05439200
H	2.28692800	4.78533800	-1.18163800
H	-4.79437300	2.46758800	-0.55160700
H	5.18580500	0.30464800	-1.17283900
H	7.35872200	4.87126300	-1.22237000
H	6.27706000	4.20691100	-3.35908800
H	4.25145900	2.84238000	0.95545300
H	6.33694500	4.18288000	0.94800500
H	-4.90009500	7.52739500	-0.94350900
H	-4.07062600	6.63605800	1.22338900
H	-3.03207400	4.23445800	-2.98832300
H	-4.37626500	6.31607000	-3.06148400
H	-7.48550300	-4.73922400	0.05222900
H	-6.05353800	-4.17696500	2.01765000
H	-3.99706500	-2.83503400	1.69697300
H	-6.83119100	-3.95326900	-2.21307900
H	4.68885900	-7.38924400	-1.36275700

H	3.92098800	-6.17224600	-3.40190100
H	2.59546800	-4.09146200	-3.16652200
H	4.12137200	-6.50145400	0.88839200
H	-0.48611500	0.40666600	6.91771000
H	-2.95798900	0.43473500	6.79141400
H	-4.12503000	-0.03012000	4.65510800
H	-2.81485800	-0.56393900	2.60238500
H	1.95683300	0.34506800	6.63779300
H	4.33471000	0.25038900	5.95674600
H	4.96501800	-0.26852400	3.61706400
H	3.20364500	-0.73373700	1.91774600

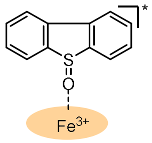

II **G = -5269.949077 Hartree**

N	-0.46901100	-1.96525700	-0.73136300
C	-1.72426900	-2.54063400	-0.64294200
C	0.44270300	-2.99456000	-0.82470000
C	-2.93488400	-1.85010500	-0.54936500
C	-1.58789900	-3.98187500	-0.68231300
C	1.83304900	-2.84923600	-0.93338900
C	-0.26152200	-4.25959300	-0.79779500
N	0.35846900	2.10265700	-1.01039100
C	-3.10168600	-0.42030300	-0.54220500
C	2.55204600	-1.60930800	-1.00346500
C	-0.55876900	3.12756700	-0.95899600
C	1.60718600	2.67607400	-1.14306300
N	-2.08347500	0.48983400	-0.70941400
C	-4.33408600	0.26290400	-0.37183600
N	1.96838300	-0.36052700	-1.05596900

C	3.96575300	-1.47601300	-1.02440000
C	-1.94036100	2.97985800	-0.78208200
C	0.13521000	4.39406300	-1.08567800
C	2.81916700	1.98388000	-1.19980600
C	1.46152900	4.11755400	-1.20147800
C	-2.65558600	1.74221300	-0.65289500
C	-4.05438600	1.62024300	-0.44606500
C	2.99218900	0.55514500	-1.12007300
C	4.24093400	-0.11744300	-1.09649900
C	6.44360000	4.32108700	-1.41994300
C	5.91794400	3.76060600	-2.58447800
C	4.74532700	3.00816000	-2.51388400
Cl	4.10181000	2.33166300	-4.01049100
C	4.07193400	2.79789400	-1.30061200
C	4.62267900	3.37671000	-0.14518400
C	5.79511900	4.13014200	-0.19851200
C	-4.32896200	6.57675000	-0.71641400
C	-3.82045800	6.06999000	0.47980400
C	-3.04874500	4.90785400	0.45712500
Cl	-2.42235600	4.29528200	1.98914500
C	-2.76453600	4.23011200	-0.73796300
C	-3.29190400	4.76132500	-1.92616600
C	-4.06485900	5.92224600	-1.92108600
C	-6.53270100	-4.18743400	-0.05490800
C	-5.70829300	-3.91334200	1.03778000
C	-4.54680000	-3.16203300	0.86029900
C	-4.18214000	-2.66598500	-0.40243600
C	-5.02914100	-2.96003700	-1.48233400
Cl	-4.62241800	-2.39570700	-3.10312600

C	-6.19311100	-3.71217000	-1.32193500
C	4.20109000	-6.44094700	-1.31715900
C	3.89979700	-5.66037600	-2.43524000
C	3.13978100	-4.50128400	-2.28814900
C	2.65458800	-4.09675200	-1.03243800
C	2.98051400	-4.89749100	0.07316500
Cl	2.44275200	-4.44019000	1.69098900
C	3.74345600	-6.05890600	-0.05632200
Fe	-0.06745000	0.06577900	-0.99942000
Cl	-0.30333400	-0.06057100	-3.39173000
C	-0.36381200	0.02417800	4.77269000
C	-1.12183000	0.41351500	5.87955200
C	-2.50416800	0.57152500	5.74003000
C	-3.13870900	0.33991200	4.51483500
C	-2.39714500	-0.07051800	3.40075600
C	-1.02465000	-0.21629400	3.55572700
S	0.09714500	-0.77990500	2.26208300
O	0.17922700	0.33566200	1.17680000
C	1.49242100	-0.56396400	3.38310300
C	1.09040400	-0.17399100	4.67231400
C	2.06721000	-0.01627400	5.65832900
C	3.40952900	-0.23228400	5.33138500
C	3.78718800	-0.61041300	4.03847800
C	2.81977800	-0.79161600	3.04298700
H	-2.40658000	-4.68538900	-0.63349700
H	0.20414600	-5.23256900	-0.86282700
H	-5.29820200	-0.20088000	-0.21915800
H	4.67771100	-2.28811800	-0.99017100
H	-0.33804400	5.36556000	-1.08276900

H	2.27445800	4.82100200	-1.31030500
H	-4.75455200	2.43910000	-0.36253100
H	5.21336600	0.35270800	-1.12850900
H	7.35646400	4.90670700	-1.47329400
H	6.40754000	3.90624100	-3.54135200
H	4.11658000	3.22377100	0.80350700
H	6.19860700	4.56529500	0.71069000
H	-4.92958100	7.48128700	-0.70137100
H	-4.01726700	6.56813000	1.42303900
H	-3.08481300	4.24878300	-2.86102500
H	-4.45826500	6.31245500	-2.85477200
H	-7.43863300	-4.77253500	0.07164400
H	-5.96597200	-4.28291000	2.02562100
H	-3.90415500	-2.94723000	1.70909900
H	-6.82014300	-3.92350200	-2.18140300
H	4.79360800	-7.34507200	-1.41987800
H	4.25454100	-5.95161600	-3.41915900
H	2.90404400	-3.89145100	-3.15523100
H	3.97574800	-6.65092600	0.82231800
H	-0.64631700	0.59734800	6.83807900
H	-3.09315500	0.88026000	6.59841500
H	-4.21216500	0.47359800	4.42534200
H	-2.88173100	-0.25806600	2.44728600
H	1.79224600	0.27588100	6.66720300
H	4.17004700	-0.10377800	6.09570000
H	4.83481300	-0.77070800	3.80386400
H	3.10301500	-1.09690800	2.04038600

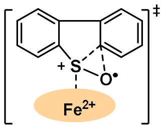

III **G = -5269.924071 Hartree**

N	-0.61906300	-2.11954800	-0.73962100
C	-1.88475400	-2.66095500	-0.69840700
C	0.26658800	-3.16928500	-0.84458100
C	-3.08913100	-1.93916300	-0.62177700
C	-1.79038500	-4.10060500	-0.76642000
C	1.66654300	-3.06098600	-0.92464900
C	-0.46698100	-4.41298100	-0.86160600
N	0.31375700	1.89189300	-0.69192300
C	-3.19673400	-0.53782600	-0.56509500
C	2.38781700	-1.85463200	-0.89032500
C	-0.57263500	2.93809100	-0.58956400
C	1.57910200	2.43027300	-0.70388500
N	-2.14988100	0.35536000	-0.60053900
C	-4.44036400	0.18896800	-0.46351600
N	1.84392000	-0.59110700	-0.78179600
C	3.82465300	-1.74844800	-0.97057000
C	-1.97240100	2.82647900	-0.51616300
C	0.16112200	4.18205700	-0.54318400
C	2.78258400	1.70706800	-0.78347100
C	1.48578500	3.86934500	-0.61322500
C	-2.69197300	1.61898700	-0.52362100
C	-4.12979800	1.51599400	-0.43941400
C	2.89151100	0.30529000	-0.81765800
C	4.13452000	-0.42078400	-0.92364000
C	6.45993500	3.98189600	-0.82622900
C	5.78958200	3.73856100	-2.02511600
C	4.60342100	3.00359900	-2.00992500

Cl	3.79280800	2.71297000	-3.55032700
C	4.05848900	2.49713400	-0.81924000
C	4.75697000	2.75705600	0.37116600
C	5.94356300	3.49006500	0.37390700
C	-4.23580600	6.50604500	-0.35735600
C	-3.88845100	5.86371600	0.83127100
C	-3.15869700	4.67530000	0.78024300
Cl	-2.73576500	3.89683600	2.30635500
C	-2.76063400	4.10103800	-0.43728800
C	-3.12483800	4.77090900	-1.61674300
C	-3.85362800	5.95955600	-1.58391500
C	-6.76719100	-4.20881700	-0.47261300
C	-6.07858100	-3.89720200	0.70106900
C	-4.89283900	-3.16583700	0.63626900
C	-4.36562000	-2.72836700	-0.58962900
C	-5.08065600	-3.05824700	-1.75208200
Cl	-4.48452900	-2.54956700	-3.33319600
C	-6.26856000	-3.78908200	-1.70595400
C	3.91433600	-6.69890600	-1.56900200
C	3.47559600	-5.92967100	-2.64834300
C	2.75217400	-4.76027300	-2.41750300
C	2.44921300	-4.32785000	-1.11541400
C	2.90493000	-5.12224400	-0.05200100
Cl	2.57082400	-4.65142200	1.61602200
C	3.62926600	-6.29620100	-0.26440800
Fe	-0.16587600	-0.10366300	-0.95886200
Cl	-0.24003500	-0.05860300	-3.30972300
C	-0.04770100	0.71950200	4.07205100
C	-0.94847200	1.26340300	5.00818300

C	-2.25740400	0.80630500	5.08082300
C	-2.68796400	-0.22589300	4.22317300
C	-1.84665000	-0.77069500	3.26194400
C	-0.54387500	-0.26003300	3.17727200
S	0.99041200	-1.09075000	2.31164100
O	-0.02700600	-0.04576000	1.46707200
C	2.06709900	-0.00891600	3.17962000
C	1.38833300	0.92102000	4.01023700
C	2.16393600	1.84815300	4.72656900
C	3.55245900	1.80433100	4.63877600
C	4.20657900	0.84929200	3.84082100
C	3.46294200	-0.06216200	3.09563000
H	-2.62908400	-4.78173700	-0.74542800
H	-0.02485400	-5.39645200	-0.93353700
H	-5.42211500	-0.25982400	-0.41319500
H	4.50731600	-2.58199200	-1.05410500
H	-0.28184200	5.16431900	-0.46144400
H	2.32609100	4.54861700	-0.59601900
H	-4.81130700	2.35169200	-0.36852000
H	5.11665600	0.02824400	-0.96216400
H	7.38288600	4.55400000	-0.83606700
H	6.17973000	4.11226600	-2.96567900
H	4.35380200	2.37273500	1.30331600
H	6.46110600	3.67563500	1.31024000
H	-4.80350500	7.43097300	-0.31913800
H	-4.17760400	6.27753600	1.79133000
H	-2.82595000	4.34211400	-2.56882700
H	-4.12116100	6.45486900	-2.51230700
H	-7.69140600	-4.77763700	-0.43474800

H	-6.46154000	-4.22095700	1.66407700
H	-4.35658200	-2.92101800	1.54831600
H	-6.79239900	-4.02434600	-2.62615300
H	4.47810700	-7.61189600	-1.73572800
H	3.69400800	-6.23759100	-3.66632600
H	2.40797000	-4.15937800	-3.25424200
H	3.96415800	-6.88342000	0.58384000
H	-0.59850200	2.02898600	5.69544000
H	-2.94101400	1.22430700	5.81267400
H	-3.70118600	-0.60960100	4.30703700
H	-2.18177100	-1.54858100	2.58602000
H	1.68425600	2.58956400	5.35820800
H	4.14245600	2.52072800	5.20314600
H	5.29084600	0.83078000	3.79388800
H	3.95035000	-0.78743700	2.45170100

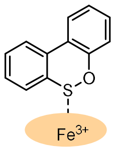

IV **G = -5270.016611 Hartree**

N	-0.50828600	-2.06665800	-1.09561900
C	-1.75344400	-2.65440300	-0.99409300
C	0.40244900	-3.09900400	-1.19961300
C	-2.97415300	-1.97554100	-0.86908600
C	-1.61784700	-4.08920200	-1.03337600
C	1.79418600	-2.95814600	-1.29983200
C	-0.29006600	-4.36300100	-1.16562500
N	0.31403200	1.91932000	-1.02470900
C	-3.11242500	-0.58074100	-0.81632600
C	2.47547400	-1.73231700	-1.29108700
C	-0.59691300	2.95195000	-0.92385000

C	1.56453500	2.50427800	-1.05296600
N	-2.08520500	0.33748500	-0.90505100
C	-4.36845700	0.10993300	-0.66065400
N	1.89109200	-0.48344200	-1.22347000
C	3.91037700	-1.59571300	-1.32560300
C	-1.98928700	2.81210300	-0.83374500
C	0.10027500	4.21343300	-0.89300700
C	2.78751400	1.82484100	-1.14667700
C	1.43197200	3.93734800	-0.96744200
C	-2.66839400	1.58528700	-0.81164700
C	-4.09518800	1.44456600	-0.66259400
C	2.92497500	0.43066900	-1.21173600
C	4.18746700	-0.26310600	-1.27175600
C	6.40607500	4.18515600	-1.04609800
C	5.82839900	3.86946400	-2.27598300
C	4.65931400	3.10823400	-2.30807500
Cl	3.95948500	2.73118400	-3.88339800
C	4.04252200	2.64708900	-1.13483500
C	4.64624200	2.98110100	0.08845200
C	5.81473800	3.74077400	0.13799100
C	-4.35102200	6.43009800	-0.73287900
C	-3.91839000	5.86271100	0.46575400
C	-3.15751700	4.69319500	0.43548300
Cl	-2.63274200	4.00597700	1.97243400
C	-2.81110500	4.06616700	-0.77139000
C	-3.26165700	4.65986900	-1.96178500
C	-4.02299200	5.82816900	-1.94912900
C	-6.55600400	-4.35733200	-0.45228500
C	-5.81334700	-3.98975600	0.67137800

C	-4.65945100	-3.22148700	0.51953900
C	-4.21992800	-2.80266600	-0.74681600
C	-4.98624600	-3.18897800	-1.85745900
Cl	-4.49563900	-2.70620500	-3.48198800
C	-6.14334600	-3.95726600	-1.72337100
C	4.14659400	-6.54932900	-1.79651400
C	3.69815200	-5.83237800	-2.90740900
C	2.94055800	-4.67571900	-2.72675000
C	2.61285000	-4.20738100	-1.44380900
C	3.07871300	-4.94849000	-0.34675100
Cl	2.71409300	-4.42199200	1.29734700
C	3.83759800	-6.10819300	-0.50973500
Fe	-0.13270500	-0.05555600	-1.51079500
Cl	-0.30663500	0.01607600	-3.79783100
H	-2.43445000	-4.79327100	-0.96401100
H	0.18193100	-5.33305600	-1.22655800
H	-5.33226500	-0.36756400	-0.55805200
H	4.61221600	-2.41577700	-1.37359800
H	-0.37053500	5.18271600	-0.81414800
H	2.25399700	4.63844700	-0.95932000
H	-4.79366400	2.26285600	-0.56243800
H	5.15851300	0.21061600	-1.26627500
H	7.31587700	4.77736100	-1.01904600
H	6.27644800	4.20825200	-3.20385200
H	4.18461900	2.63319100	1.00811800
H	6.26012500	3.98368100	1.09794400
H	-4.94301100	7.34016600	-0.71059600
H	-4.16632000	6.31921600	1.41792600
H	-3.00359900	4.18889000	-2.90583500

H	-4.35724300	6.26498900	-2.88518500
H	-7.45614300	-4.95530500	-0.34600700
H	-6.12947500	-4.29922100	1.66287200
H	-4.07833400	-2.93485800	1.39122000
H	-6.70984800	-4.23644200	-2.60519000
H	4.73707900	-7.45154300	-1.92489300
H	3.93582300	-6.17088500	-3.91123100
H	2.58896100	-4.11523400	-3.58809500
H	4.18046700	-6.65452300	0.36228700
C	-0.24156100	1.20050800	4.38763900
C	-1.07512200	1.74235900	5.38106200
C	-2.44439800	1.47916700	5.40382700
C	-3.01529000	0.65681300	4.42864500
C	-2.20947000	0.08227500	3.44446800
C	-0.84264500	0.34842700	3.43872600
S	1.40364800	-0.85050400	2.93101200
O	-0.08955100	-0.19478600	2.40279400
C	2.08143700	0.63393400	3.62702300
C	1.20320600	1.51235300	4.30119300
C	1.75407600	2.67935700	4.85466300
C	3.12557600	2.93007000	4.79819400
C	3.97974900	2.03092300	4.15549500
C	3.45272300	0.88937400	3.54716400
H	-0.64281800	2.37101500	6.15330400
H	-3.06250300	1.91149800	6.18488000
H	-4.08092900	0.44781400	4.44132700
H	-2.62292500	-0.56976100	2.68158500
H	1.10174900	3.40427700	5.33117800
H	3.52388800	3.83512500	5.24712600

H	5.04708900	2.22518500	4.10599700
H	4.10161100	0.20140200	3.01293200

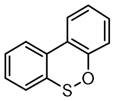
IV’ **G = -935.359302 Hartree**

C	-0.77511900	-0.55449000	-0.02902700
C	-1.55291200	-1.68093600	-0.34599400
C	-2.94597600	-1.63225400	-0.32334100
C	-3.59912700	-0.44429200	0.01405800
C	-2.85493700	0.69956000	0.30580000
C	-1.46442000	0.63753400	0.27284500
S	0.56623200	2.10900600	-0.39833200
O	-0.76629100	1.78579700	0.63163000
C	1.45383000	0.59499800	-0.12431900
C	0.70414300	-0.59411300	0.01984400
C	1.41617300	-1.78523100	0.23140000
C	2.81126900	-1.80385000	0.23413700
C	3.53121000	-0.62094400	0.05687000
C	2.84976400	0.58721700	-0.10047300
H	-1.05890100	-2.60654400	-0.62458400
H	-3.51949300	-2.51971200	-0.57345600
H	-4.68409700	-0.39983000	0.03322600
H	-3.33390400	1.64064400	0.55651200
H	0.87324700	-2.70941600	0.40278900
H	3.33430900	-2.74300800	0.38846300
H	4.61706500	-0.63074600	0.06737900
H	3.39900700	1.51893900	-0.20086300

DBTO-TS-IV **G = -935.268345 Hartree**

C	0.67112500	0.71099400	0.11307200
C	1.50736800	1.84289500	0.08860300

C	2.87362500	1.71094600	-0.11525700
C	3.42865400	0.43036600	-0.31764300
C	2.65237400	-0.71839000	-0.27001500
C	1.27997800	-0.56685100	-0.00517500
S	-0.07755400	-1.86280400	-0.25304900
O	0.70971500	-1.79985500	1.21593400
C	-1.31336000	-0.61206700	-0.16446700
C	-0.77881300	0.67445100	0.10498400
C	-1.68219000	1.74118100	0.24968700
C	-3.04696800	1.51784900	0.09173600
C	-3.55220700	0.24147100	-0.20977500
C	-2.68228700	-0.83814900	-0.33419200
H	1.06482000	2.82883700	0.20599800
H	3.50999200	2.58961200	-0.14287800
H	4.49195400	0.33107600	-0.52052500
H	3.08443800	-1.70268400	-0.41063200
H	-1.31839100	2.73966400	0.47324000
H	-3.73611000	2.35056700	0.20093800
H	-4.62085700	0.09413300	-0.32996600
H	-3.05650200	-1.83568000	-0.54420800
